# Supplementary material for: Feasibility randomised multicentre, double-blind, double-dummy controlled trial of anakinra, an interleukin-1 receptor antagonist versus intramuscular methylprednisolone for acute gout attacks in patients with chronic kidney disease (ASGARD): protocol study
Source: BMJ Open. 2017 Sep 5;7(9):e017121. doi: 10.1136/bmjopen-2017-017121 (PMC5588981; doi:10.1136/bmjopen-2017-017121)
Supplement: Supplementary file 2 [file bmjopen-2017-017121supp002.pdf]

PARTICIPANT INFORMATION SHEET (VERSION 1.1)

Centre Number: **Study Number:**

ASGARD: THE STUDY OF ANAKINRA VERSUS STEROIDS FOR THE TREATMENT OF ACUTE GOUT ATTACKS IN PEOPLE WITH KIDNEY DISEASE

Invitation to participate in the above project

We would like to invite you to take part in our research project. Before you decide, we would like you to understand why the research is being done and what it would involve for you. One of our team will go through the information sheet with you and answer any questions you have. We'd suggest this should take about 30 minutes. Talk to others about the study if you wish and ask us if there is anything that is not clear.

Part 1 tells you the purpose of this study and what will happen to you if you take part. Part 2 gives you more details information about the conduct of the study.

Part 1

What is the purpose of the project?

Gout attacks affect approximately 1.3 million people with kidney disease in the UK. We do not know the safest and best way to treat an attack of gout in these patients. We want to compare the safest treatment currently available, steroids, with a new treatment that is being increasingly used called Anakinra. Anakinra stops the action of a chemical called interleukin-1, which plays an important role in the acute gout attack, and we think it might be a better alternative to steroid treatment. This project is looking at a small number of people and will provide information to plan a larger study. We hope the larger study will then go on to tell us which treatment may be better, safer and more cost-effective.

Why have I been invited?

You are being asked to participate in this clinical research project because you have acute gout and kidney disease.

Do I have to take part?

No, it is up to you to decide whether or not to join the project. We will describe the project and go through this information sheet. If you agree to take part, we will ask you to sign a consent form. You are free to withdraw at any time, without giving a reason. This would not affect the standard of care you receive.

What will happen to me if I take part?

If you agree to take part in the project, a member of the project team may undertake some additional blood tests (about two teaspoonful) to make sure it is suitable for you to be involved with the project. If this is ok, then you will be asked to fill a consent form to proceed. This project lasts for 8 weeks but most of your involvement will be for the first 7 days.

PARTICIPANT INFORMATION SHEET (VERSION 1.1)

Centre Number: **Study Number:**

This project compares two different treatments. We put people into two groups and give each group a different treatment. The results are then compared to see if one is better. To try and make sure the groups are the same to start with, each participant is put into a group by chance (randomly). The chance of being on a particular type of treatment in 50:50. This is a “double-blind trial,” so neither you nor your doctor will know in which treatment group you are in (although, if your doctor needs to find out he/she can do so).

We will undertake an assessment on the first day (Day 1) before you receive the treatment. This will take about an hour and consists of clinical questions and examination, blood tests and you will be asked to complete some questionnaires. You will also be issued a diary for you to assess your pain and you will be asked to complete some questionnaires to assess how the pain is affecting your ability to go about your day.

You will then be given the two injections, one in the buttock, and one under the skin in your tummy or thigh. The injection into your buttock would have been part of your usual care anyway.

You will also be issued a further 4 injections to give under the skin daily over the next 4 days. We will teach you or someone who lives with you to give these injections. If we cannot do this, we can organise a nurse to visit your home to do this. You will be given a small bin to dispose of your empty syringes safely and return after 7 days.

An outline of your visits and assessments following your enrolment is shown in the figure below.

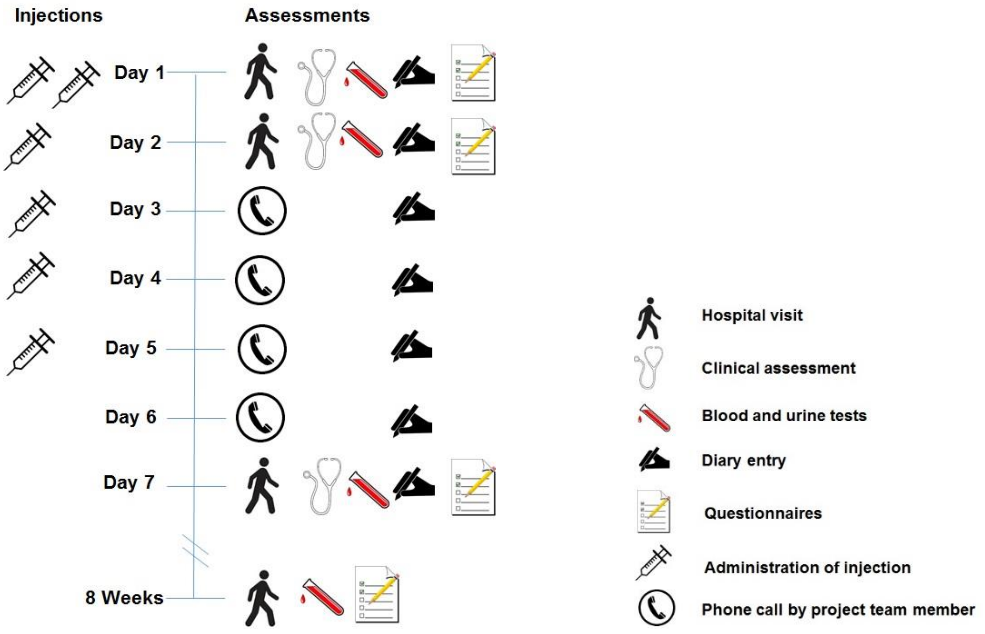

You will then have to attend hospital on the second and seventh day for about an hour. We will call you on the days you don’t attend hospital to check that you have had the treatment

and filled your diary. There is a final visit and assessment after 8 weeks for blood tests and questionnaires, one additional questionnaire will look at all the NHS services you have used over the last eight weeks. You may also be asked to have an interview to give your feelings on being part of this project, you do not need to do this if you don't want to.

Your contact information will be stored at your local hospital so that we can contact you about the study, any results of the project and any future studies that we may plan.

You will have blood tests (about two tablespoons) and urine samples (a small cupful) on the second day, the seventh day and after eight weeks. Some of these samples will be tested straight away in the local NHS laboratory, while some will be processed, stored and analysed at the end of the study at Southend Hospital or specialised laboratories linked to the project. With your consent, a tiny sample of your blood plasma, serum, urine and DNA will be stored in a clinical laboratory at Southend University Hospital under the custodianship of Dr Gowrie Balasubramaniam (Chief Investigator).

Expenses and payments

You will not be paid for taking part in the study. We can pay for travel expenses on the days you need to attend hospital.

What will I have to do?

You will have to receive additional injections for five days and make assessments for the week. You will be issued with a diary to make the assessments, this should take less than five minutes a day. We will call you to make sure you have done this. You will also have to attend hospital for a clinical assessment and to fill some questionnaires on the second day, the seventh day and at eight weeks. This visit will probably take approximately forty-five minutes, with forty minutes for the questionnaires and five minutes for the assessment and blood tests.

A small number of participants (12) will be asked to undertake an interview session when you attend at eight weeks. This can take up to an additional hour. We will ask you about your experience of participating in the project and your answers will be recorded using an audio recording device. We will specifically choose participants to give a broad representation of people based on age and gender. The questions you will be asked will be related to your feelings on what you had to do as part of this project, we will also ask if you feel we could do things differently. This will help improve our future research. You can decide not to undertake this particular aspect of the project if you wish.

What are the alternatives for treatment?

Two other alternative treatments are available. NSAIDS are the best treatment for gout but they can be harmful in people with kidney disease. Colchicine is also a good treatment but can cause severe diarrhoea and other serious complications, as colchicine can build up in the body with kidney failure.

What are the possible disadvantages, risks and side-effects?

Anakinra is a new treatment that works by reducing your immune reaction and there can be complications associated with this. In one study where Anakinra was used to help treat people with Rheumatoid Arthritis, there was a risk (<2%) of picking up an infection (like a chest infection) and a lowering of the white blood cell count. These participants were on other treatments that weakened their immune reaction and treatment with Anakinra lasted for many months. We do not anticipate these problems as we are not involving people on other treatments that affect the immune system and treatment is only for 5 days.

We will make sure that you don't have a serious infection and that your blood counts are normal before you are involved with the project. You will be monitored to make sure everything is going well and we will also check your blood counts.

You will be receiving injections under the skin for five days. Some people have had reactions where they inject their skin for many months but we do not feel this will be a problem for the five days you will receive the injection. We will have a look at the injection site when you visit the hospital.

We will ask that you refrain from using an additional or rescue pain killer for four hours prior to a pain assessment, this is to obtain a more accurate and fair comparison of the two treatments. You would normally have to wait for four hours in between doses of pain killers anyway, so you can minimise any discomfort by doing the assessment just before a dose. You do not have to do the assessments at the exact time every day but at roughly the same sort of time i.e. late morning or afternoon.

What are the possible benefits of taking part?

You will not be given a treatment that we already know may be harmful for you. You will be looked after by a specialist and you will be given contact details to use if there are any problems. If this study is successful then it would help develop clear guidelines to help doctors treat people with kidney disease and gout, something we know is currently not being done well.

There is also some suggestion that using this novel treatment may be beneficial for other conditions.

Will taking part in the study affect your usual care?

The only alteration to your treatment is that you will be asked to refrain from taking additional or rescue painkillers for four hours prior to doing an assessment of your joint. You would normally wait for four hours in between doses of painkillers and we would suggest that you do the assessment just before your next dose. We will not alter any of your other medications or interfere with your other treatments in any way. You will continue to be seen by your GP and by any hospital clinics that you usually attend.

What happens when the research study stops?

The medication is only used as part of the study. Any future gout attacks you have will be treated as before. You will not know what exact treatment you have had as part of the study.

What if there is a problem?

Any complaint about the way you have been dealt with during the study or any possible harm you might suffer will be addressed. The detailed information on this is given in Part 2.

Will my taking part in this study be kept confidential?

Yes, we will follow ethical and legal practice and all the information about you will be handled in confidence. The details are included in part 2.

This completes Part 1 of the information sheet.

Part 2 of the information sheet

If the information in Part 1 has interested you and you are considering participation please read the additional information in Part 2 before making any decision.

What if relevant new information becomes available?

Sometimes we get new information about the treatment being studied. If this happens your research doctor will tell you and discuss whether you should continue in the study. If you decide not to carry on, your research doctor will make arrangements for your care to continue. If you decide to continue in the study, he/she may ask you to sign an agreement outlining the discussion.

What will happen if I don't want to carry on with the study?

You can withdraw from treatment at any time but keep in contact with us to let us know your progress. Information collected may still be used. Any stored blood samples that can still be identified as yours can be destroyed if you so wish.

What if there is a problem?

If you have a concern about any aspect of this study, you should ask to speak to the researchers who will do their best to answer your questions. If you remain unhappy and wish

to complain formally, you can do this using the Complaints Officer for __________________

hospital on: ______________. Details can be obtained from_________________________.

In the event something goes wrong and you are harmed during the project, and this is due to someone's negligence then you may have grounds for a legal action for compensation

against ____________________ hospital but you may have to pay your legal costs. The normal National Health Service complaints mechanisms will still be available to you.

Will my taking part in this study be kept confidential?

If you join the study, some parts of your medical records and the data collected for the study will be looked at by authorised persons from the organisers of the research. They may also be looked at by authorised people to check that the study is being carried out correctly. All will have a duty of confidentiality to you as the research participant and we will do our best to meet this duty.

Your research data will be stored using a unique study code which is non-identifiable, but can be linked back to your original records. All written information will be kept in a locked filing cabinet in a locked room. Any web-based data will be stored in a secure password protected central database at the Anglia Ruskin Clinical Trials Unit. Some of the data will be

analysed at the University of East Anglia and the University of Essex. Only individuals directly involved with the study will have access to this information. All data will be under the custodianship of Dr Gowrie Balasubramaniam. Information may be retained for future studies but this will be after the appropriate ethical approvals. It will be retained for fifteen years and be disposed of securely after this period.

Will my GP know about this project?

We will inform your GP of your participation with your permission and we will also inform him/her of any clinical results, and of any new medical problems we might find as a result of your participation in the project.

What will happen to any samples I give?

Blood and urine samples taken for this study will be processed at the local centre and transferred for storage to Southend University Hospital. The samples with be labelled with a unique study code which will not identify you, but can be linked back to your original records. The samples can be stored for up to fifteen years and some will be sent to other laboratories both in the UK and within the European Union for further analysis. They may be used for future research that cannot yet be specified but this will be done after the appropriate ethical approvals. Samples will be under the custodianship of Dr Gowrie Balasubramaniam.

Will any genetic tests be done?

We will test for changes in the structure of the DNA. There will be **no** DNA genetic testing per se that may reveal significant results such as a family genetic condition etc.

What will happen to the results of the research study?

The results will be examined by the researcher at the end of the study and a report will be produced. We will share our results with the funding body, the National Institute for Health Research. We may publish the study in a scientific journal and present the results at scientific meetings. You will not be identified in any of the reports, presentations or publications. We hope to use the results to undertake a larger, more definitive study.

A summary of the findings of the study can be made available to you if you wish from the research team or the principle investigator.

Who is organising and funding the research?

The research is being organised by Southend University Hospital. It is being funded by the National Institute of Health Research.

Who has reviewed the study?

ASGARD

PARTICIPANT INFORMATION SHEET (VERSION 1.1)

Centre Number: **Study Number:**

All research in the NHS is looked at by independent group of people, called a Research Ethics Committee, to protect your interests. This study has been reviewed and given a favourable opinion by The London Research Ethics Committee.

What if I become unwell during the study?

If during the study you become unwell or are concerned, as well as the usual services provided by the NHS, you can also contact a member of the study team on this number during normal working hours on <PI/Research member contact number>.

You can also contact <a research member of the team or the principle investigator out-of-hours> via the hospital switch board on this number <centre switch board number>.

If you are unwell, and need urgent advice or assistance do not delay in seeking further advice or treatment as usual through the NHS services.

Thank you for taking the time to read this information sheet and considering to take part in this study. If you would like more information or want to ask questions about the study please contact the study team.

For further information contact:

Research Nurse

Principal Investigator

<Contact details>
