# Supplementary material for: Feasibility randomised multicentre, double-blind, double-dummy controlled trial of anakinra, an interleukin-1 receptor antagonist versus intramuscular methylprednisolone for acute gout attacks in patients with chronic kidney disease (ASGARD): protocol study
Source: BMJ Open. 2017 Sep 5;7(9):e017121. doi: 10.1136/bmjopen-2017-017121 (PMC5588981; doi:10.1136/bmjopen-2017-017121)
Supplement: Supplementary file 3 [file bmjopen-2017-017121supp003.pdf]

CONSENT FORM (VERSION 1.2)

Centre Number: **Study Number:**

Participation Id. no. for this trial:

ASGARD STUDY: How does a new treatment called Anakinra, an Interleukin-1 inhibitor, compare with the currently available best treatment, Steroids, for the treatment of gout attacks in people with kidney disease

Name of Researcher: Study reference:

This form must be completed and signed by the research participant in the presence of Principle Investigator or someone from the research team designated by the Principle Investigator

Please initial box

1. I confirm that I have read and understood the information sheet dated ………………….. (Version……….). I have had the opportunity to consider the information, ask questions and have had these answered satisfactorily. ☐

2. I understand that my participation is entirely voluntary and that I am free to withdraw at any time without giving any reason, without my medical or legal rights being affected. ☐

3. I understand that relevant sections of my medical notes and data collected will be looked at by individuals who are part of the study team (including staff from Southend University Hospital, Anglia Ruskin University, University of East Anglia and University of Essex), from regulatory authorities or from the NHS Trust, where it is relevant to my taking part in this research. I give permission for these individuals to have access to my records. ☐

4. I understand, and I agree that my identifiable routine blood tests will be stored within the NHS clinical system and will be available to doctors looking after me in the future. ☐

5. I understand that my participation in the study is voluntary and that I am free to leave the study at any time without having to give a reason and that this will not affect my medical care in any way. ☐

6. I understand that I will be asked to refrain from using additional / rescue pain killers for four hours (normal time in-between doses) prior to an assessment, this may mean that I make a slight adjustment to perform an assessment before I take the next dose. ☐

7. I agree that if I withdraw or I am withdrawn from the study that data already collected can be retained and included in the data analysis. ☐

8. I understand that I will be required to give blood samples as part of this study. ☐

CONSENT FORM (VERSION 1.2)

Centre Number: **Study Number:**

Participation Id. no. for this trial:

9. I understand and I agree that the samples I provide will be gifted by myself for use in future research. They will be transferred to specialist laboratories and analysed by members of the study team or stored (link-anonymised) for up to fifteen years and can be used for future, as yet unspecified, medical research into health, illness and medical treatment. This research will be subject to proper scientific and ethical review. (Optional) ☐

10. I agree, if required, to undertake an interview and give permission for audio recordings that will be stored for a small qualitative aspect of this study. (Optional) ☐

11. I agree that my contact information can be made available to the research team as part of the study assessments, for any new findings that may become relevant during the course of the study and for future studies that I might be interested in. ☐

12. I give consent for storage and studies on my DNA. I understand that this is **not** genetic testing per se that may reveal significant results such as a family genetic condition. (Optional) ☐

13. I agree that samples can be exported to countries in the European Union for further analysis. The collaborators / recipient country will ensure that the material has been handled properly and that the required standards of that collaborating institution / country have been met. (Optional) ☐

14. I agree to my GP being informed of my participation in the study. ☐

15. I agree to take part in the above study. ☐

Name of Patient	Date	Signature

Name of Person taking consent	Date	Signature

When completed: 1 for participant 1 for researcher site file; 1 (original) to be kept in medical notes.
