# Supplementary material for: Feasibility randomised multicentre, double-blind, double-dummy controlled trial of anakinra, an interleukin-1 receptor antagonist versus intramuscular methylprednisolone for acute gout attacks in patients with chronic kidney disease (ASGARD): protocol study
Source: BMJ Open. 2017 Sep 5;7(9):e017121. doi: 10.1136/bmjopen-2017-017121 (PMC5588981; doi:10.1136/bmjopen-2017-017121)
Supplement: Author's manuscript [file bmjopen-2017-017121.draft_revisions.pdf]

BMJ Open is committed to open peer review. As part of this commitment we make the peer review history of every article we publish publicly available.

When an article is published we post the peer reviewers' comments and the authors' responses online. We also post the versions of the paper that were used during peer review. These are the versions that the peer review comments apply to.

The versions of the paper that follow are the versions that were submitted during the peer review process. They are not the versions of record or the final published versions. They should not be cited or distributed as the published version of this manuscript.

BMJ Open is an open access journal and the full, final, typeset and author-corrected version of record of the manuscript is available on our site with no access controls, subscription charges or pay-per-view fees (http://bmjopen.bmj.com).

If you have any questions on BMJ Open's open peer review process please email editorial.bmjopen@bmj.com

BMJ Open

Study protocol for a feasibility randomised multi-centre, double-blind, double-dummy controlled trial of Anakinra, an interleukin-1 receptor antagonist versus intramuscular methylprednisolone for acute gout attacks in patients with chronic kidney disease: ASGARD

Journal:	*BMJ Open*
Manuscript ID	bmjopen-2017-017121
Article Type:	Protocol
Date Submitted by the Author:	13-Apr-2017
Complete List of Authors:	Balasubramaniam, Gowrie; Southend University Hospital NHS Foundation Trust, Department of Renal Medicine<br>Parker, Trisha; Anglia Ruskin University, Clinical Trials Unit<br>Turner, David; University of East Anglia, Public Health and Primary Care<br>Parker, Mike; Postgraduate Medical Institute, Anglia Ruskin University,<br>Scales, Jonathan; University of Essex, School of Health and Humanities<br>Harnett, Patrick; Southend University Hospital NHS Foundation Trust, Department of Renal Medicine<br>Harrison, Michael; Anglia Ruskin University, Clinical Trials Unit<br>Ahmed, Khalid; Princess Alexandra Hospital NHS Trust<br>Bhagat, Sweta; West Suffolk Hospitals NHS Trust<br>Marianayagam, Thiraupathy; East and North Hertfordshire NHS Trust<br>Pitzalis, Costantino; William Harvey Research Institute, Experimental Medicine and Rheumatology<br>Mallen, Christian; Keele University, Arthritis Research UK Primary Care Centre<br>Roddy, Edward; Keele University, Research Institute for Primary Care and Health Sciences; Keele University<br>Almond, Mike; Southend University Hospital NHS Foundation Trust, Department of Renal Medicine<br>Dasgupta, Bhaskar; Southend Hospital NHS Trust, Rheumatology
**Primary Subject Heading**:	Rheumatology
Secondary Subject Heading:	Renal medicine, Research methods
Keywords:	Acute gout, Chronic kidney disease, Feasibility, Randomised controlled trial, Anakinra, Intramuscular methylprednisolone

SCHOLARONE™
Manuscripts

Study protocol for a feasibility randomised multi-centre, double-blind, double-dummy controlled trial of Anakinra, an interleukin-1 receptor antagonist versus intramuscular methylprednisolone for acute gout attacks in patients with chronic kidney disease: ASGARD

Gowrie Balasubramaniam[1]*, Trisha Parker[2], David Turner[3], Mike Parker[2], Jonathan Scales[4], Patrick Harnett[1], Michael Harrison[2], Khalid Ahmed[5], Sweta Bhagat[6], Thiraupathy Marianayagam[7], Costantino Pitzalis[8], Christian Mallen[9], Edward Roddy[9], Mike Almond[1], Bhaskar Dasgupta[1]

Author affiliations

[1]Southend University Hospital NHS Foundation Trust, Prittlewell Chase, Southend, Essex, SS0 0RY

[2]Anglia Ruskin University, Post Graduate Medical Institute Clinical Trials Unit, Bishops Hall Lane, Chelmsford, CM1 1SQ

[3]Norwich Medical School, University of East Anglia, Norwich, NR4 7TJ

[4]School of Health and Human Sciences, University of Essex, Wivenhoe Park, Colchester, CO4 3SQ

[5]The Princess Alexandria Hospital NHS Trust, Hamstel Road, Harlow, Essex, CM20 1QX

[6]West Suffolk Hospital NHS Foundation Trust, Hardwick Lane, Bury St Edmunds, Suffolk, IP33 2QZ

[7]Lister Hospital, East and North Herfordshire NHS Trust, Corey Mills Lane, Stevenage, Hertfordshire, SG1 4AB

[8]Centre for Experimental Medicine and Rheumatology, William Harvey Research Institute, Barts and the London, Charterhouse Square, London EC1M 6BQ

[9]Primary Care and Health Sciences, Keele University, Keele, Staffordshire, ST5 5BG

*Corresponding author: gowrie@doctors.net.uk

Protocol version 1.4.

Word count 5436

Figures 2, tables 2

Conflict of interest: none

For peer review only - http://bmjopen.bmj.com/site/about/guidelines.xhtml

Abstract

Introduction: Acute gout occurs in people with chronic kidney disease, who are commonly older people with co-morbidities such as hypertension, heart disease and diabetes. Potentially harmful treatments are administered to these vulnerable patients due to a lack of clear evidence. Newly available treatment that targets a key inflammatory pathway in acute gout attacks provides an opportunity to undertake the first ever trial specifically looking treating people with kidney disease. This paper describes the protocol for a feasibility randomised controlled trial comparing Anakinra, a novel interleikin-1 antagonist versus steroids in people with chronic kidney disease (ASGARD).

Methods and Analysis: ASGARD is a two-parallel group double-blind, double dummy multicentre randomised controlled trial comparing Anakinra 100mg, an interleukin-1 antagonist, subcutaneous for five days against intramuscular methylprednisolone 120mg. The primary objective is to assess the feasibility of the trial design and procedures for a definitive randomised controlled trial. The specific aims are: 1) test recruitment and retention rates, and willingness to be randomised; 2) test eligibility criteria; 3) Collect and analyse outcome data to inform sample and power calculations for a trial of efficacy; 4) collect economic data to inform a future economic evaluation estimating costs of treatment; 5) assess capacity of the project to scale up to a national multi-centre trial. We will also gather qualitative insights from participants. It aims to recruit 32 patients with a 1:1 randomisation.

Information from this feasibility study will help design a definitive trial and provide general information in designing acute gout studies.

Ethics and Dissemination: The London-Central Ethics Committee approved the protocol. The results will be disseminated in peer-reviewed journals and at scientific conferences.

Registration details: EudraCT No. 2015-001787-19, NCT / Clinicalstrials.gov No. NCT02578394, WHO Universal Trials Reference No. U1111-1175-1977. NIHR Grant PB-PG-0614-34090.

For peer review only

For peer review only - http://bmjopen.bmj.com/site/about/guidelines.xhtml

Strengths and limitations of this study

Assessing the feasibility of undertaking a definitive robustly designed double-blind, double dummy study.

Qualitative aspects to current study design will help future study be more patient-orientated.

Current study is not designed to find differences in outcomes.

For peer review only

Introduction

Background and rationale

Chronic kidney disease (CKD) affects 5% of the UK population (1) and 40% of patients with CKD 3 and 4 have chronic gout (2), suggesting that 1.32 million people, predominantly older patients, have CKD and gout in the UK. The overall incidence of acute gout attacks has been estimated to be approximately 2 per 1000 person-years (3,4). The prevalence of gout is increasing due to increasing prevalence of co-morbid conditions that are associated with hyperuricaemia such as hypertension, obesity, metabolic syndrome, diabetes and chronic kidney disease (4,5).

In a UK study, approximately 89.4% of patients were treated for their acute attack with non-steroidal anti-inflammatory agents (NSAIDS) which are well known to worsen kidney failure (5). Secondary care data from the US showed that up to 50% of acute gout patients had CKD and 40% were given contra-indicated medications (6). This is similar to our experience where a review of in-patient acute gout care showed half of all patients with acute gout and kidney disease were given potentially harmful medications. Mismanagement of acute gout attacks in patient with kidney disease occurs as there is no firm evidence-base for treating acute gout attacks in people with kidney disease (7).

The lack of evidence in the literature for the use of conventional agents in patients with CKD is primarily because patients with CKD are excluded as NSAIDS are used as an active comparator in randomised controlled trials. Guidelines continue to suggest using colchicine with in patients with renal disease even with its toxicity profile and poor evidence base (8,9,10). We now have an opportunity to conduct a trial using two agents that would not be expected have adverse effects on renal function.

Treatment options for patient with acute gout attacks and CKD

For peer review only - http://bmjopen.bmj.com/site/about/guidelines.xhtml

NSAIDS have the largest evidence base for use in acute gout but are contra-indicated in CKD as they can cause an acute kidney injury. They can also worsen heart failure, hypertension, liver failure and cause gastro-intestinal bleeding (7,11). Colchicine is an alternative anti-inflammatory agent, it has a narrow therapeutic index and a low dose regime is advocated following findings in the AGREE trial. Even though this study excluded patients with moderate to severe kidney disease (glomerular filtration rate < 60 mls/m^3), 23% of patients on the low dose treatment still experienced diarrhoea (12). Diarrhoea is more common in patients with CKD, they are also more likely to have drug accumulation and serious side effects from toxicity such as bone marrow failure, rhabdomyolysis, pancreatitis and myopathy (7).

Glucocorticoids are an effective treatment with some evidence for use in CKD patients from older trials. Triamcinolone, a long-acting synthetic corticosteroid, was shown to be as effective as adrenocorticotrophic hormone, a hormone that increases endogenous cortisol secretion (11). A more recent double-blind randomised controlled study (13) showed that Prednisolone 35mg for 5 days was not inferior to Naproxen for acute gout attacks, although patients with moderate to severe renal impairment were excluded.

Interleukin-1 inhibitors

Interleukin-1 (IL-1) signalling has been shown to play a key role in gout associated inflammation (14,15) and targeted therapy is now available. Two agents of IL-1 have been used in randomised controlled trials. Rilonocept, an IL-1 decoy receptor, has been shown to be efficacious in reducing acute flares during the initiation of urate lowering therapy (16,17) but no added benefit was obtained for the treatment of acute gout when it was used in addition to an NSAID (18). Canakinumab, a monoclonal antibody against IL-1 beta showed efficacy preventing acute flares during the initiation of urate lowering therapy (19), and for acute gout in patients with difficult disease or contra-indication to colchicine or NSAIDs (20,21,22). It was associated with increased adverse events after a single injection (23) which was felt in part due to its prolonged action which can be for many weeks (24). This study will use anakinra (Kineret), an IL-1 receptor antagonist (IL-1 RA) that competes with IL-1beta for its receptor (IL-1R). A pilot study of its use in gout showed prompt resolution of symptoms in patients treated with anakinra (25), including in three patients with renal impairment. There have been further reports of the use of anakinra in difficult to treat cases showing good efficacy, with most patients having a good response within twenty-four hours (26-31).

Anakinra has a good pharmacokinetic profile with a shorter duration of action, accumulation can occur in severe renal failure (32) and this had been linked to an increased risk of

For peer review only - http://bmjopen.bmj.com/site/about/guidelines.xhtml

infection in one case report (31). The dose of a daily 100mg has been used in studies looking at treatment of Rheumatoid Arthritis (usually between 50mg to 150mg) and it was well tolerated for up to three years (33-37). The licensed use of Anakinra is for Cryopryin Associated Periodic Syndrome where dosing is 3mg – 4mg per kg in severe disease (doses up to 800mg) (38). The rationale for the duration of treatment use of 100mg s/c for 5 days come from emerging data where Anakinra has been used to treat acute gout with good efficacy and in patients with CKD without too many adverse events (25-29). Some reports vary between 3 to 5 days but most use 5 days' duration of treatment to ensure adequate response in all patients, including our own experience (30). We have intentionally avoided using Anakinra in patients with severe renal failure (GFR < 30mls/min/1.73m^2) although it has been used safely in patient on haemodialysis using an alternate regime (39) and we have some experience of this in our centre (30). Anakinra is much cheaper compared to Canakinumab (£131.15 vs. £9927.90 for a course of treatment), which has been given a license in the UK for use in difficult to treat gout.

Safety experience stem mainly from studies in Rheumatoid Arthritis where patients were on concurrent immunosuppressive treatment and the duration of treatment was prolonged (up to six months). A slightly higher risk of infection (2%) and neutropenia was noted. We do not anticipate these issues as the treatment time for acute gout will be short, five days versus many months, and we feel the risk of infection should be much less. We are excluding people with concurrent immunosuppressive treatment and will exclude people with serious infection. There is some data of the use of anakinra in the setting of an active infection from the first clinical trial where it was used to treat people with severe septicaemia and those who received Anakinra were not worse off (40). There are also reports of the use of Anakinra for acute gout for hospitalised in-patients with active infections and multiple co-morbidities (27-29). There was one case report of neutropenia in one patient with a kidney transplant with severe renal impairment (31). Blood counts will be checked at the end of treatment at day 5 and participants will also be assessed for infection face-to-face on day 2, day 7 and 8 weeks, and by telephone contact on day 3, day 4 and day 5. Participants will be asked to stop treatment if serious infection develops.

Methylprednisolone

Methylprednisolone acetate is one of the most commonly used long-acting intra-muscular agent in the UK, we have shown this in our region (41), and it is equivalent to Triamcinolone in other joint conditions (42).

We are using methylprednisolone 120mg given by intramuscular injection which is as efficacious as oral steroids with a lower cumulative dose (120mg vs.175mg) in other

For peer review only - http://bmjopen.bmj.com/site/about/guidelines.xhtml

For peer review only

rheumatology conditions, and this is equivalent to the dose of oral prednisolone used in the trial by Janssens et al. (13) (35 mg for 5 days). This well designed double-blind randomised controlled study showed that Prednisolone 35mg for 5 days was not inferior to Naproxen. The rate of side-effects even with short-term steroid treatment in acute gout is unknown. Our study may help establish steroids, especially intramuscular methylprednisolone which has limited evidence base, as a potential safe, cheap, and readily available treatment for acute gout in patients with renal disease. If the initial results show equivalent efficacy between the two treatment arms, then we could go on to establish steroids as the standard treatments and make considerable savings.

Our use is only as a one-off injection (as is commonly done in routine practice) and we do not anticipate long-term complications. Short term side-effects we may encounter are uncontrolled blood sugars in diabetics and worsening odeama in people with heart failure and kidney failure. The latter should occur less with methylprednisolone which is associated with less mineralocorticoid effect than other steroids (e.g. prednisolone). The placebo equivalent for the methylprednisolone will be lipofundin, a lipid emulsion commonly used in parenteral nutrition but has been use as a placebo agent in other trial using steroids.

Objectives

Primary objective

The primary objective of ASGARD is to assess the feasibility of the trial design and procedures for a definitive randomised controlled trial. The specific aims are: 1) test recruitment and retention rates, and willingness to be randomised; 2) test eligibility criteria; 3) Collect and analyse outcome data to inform sample and power calculations for a trial of efficacy; 4) collect economic data to inform a future economic evaluation estimating costs of treatment; 5) assess capacity of the project to scale up to a national multi-centre trial. We will also gather qualitative insights from participants about their experience from being in the feasibility trial to help design the subsequent definitive trial.

Secondary objectives

Information will be obtained to help with the design of the subsequent study including testing of proposed primary and secondary outcomes described below.

Trial Design

ASGARD is a two-parallel group double-blind, double dummy multicentre randomised controlled trial with a 1:1 allocation ratio comparing Anakinra 100mg subcutaneous for five

For peer review only - http://bmjopen.bmj.com/site/about/guidelines.xhtml

days with a one-off methylprednisolone (Depo-Medrone) 120mg intramuscular injection (Figure 1).

Methods

Study setting

ASGARD aims to recruit from centres in the East of England region. Recruitment will be from patients who present to primary and secondary care with acute gout and chronic kidney disease, this will also include in-patient who develop an acute gout attack. Known patients with moderate kidney disease will also be primed to contact the research team by working with local primary care providers to send out letters of invitation. Recruitment will occur in the secondary care setting with treatment occurring on an out-patient basis, or can occur in secondary care if symptoms warrant admission or if the participant is already an in-patient and develops an acute gout attack.

Eligibility criteria

Table / Box 1

Table / Box 2

Baseline Assessments

Baseline assessment will include basic medical observations, routine bloods, and basic clinical examination. The 2015 American College of Rheumatology/ European League Against Rheumatism Collaborative Initiative will also be used at baseline assessment (43).

Interventions

Eligible participants will undergo 1:1 block randomisation to one of two treatment arms; anakinra 100mg s/c daily for five days and one-off placebo methylprednisolone (lipofundin MCT) intramuscular (gluteal) or one-off intramuscular methylprednisolone (Depo-Medrone) and placebo anakinra s/c for five days. The study drug and its placebo will be provided by SOBI in its commercially available form. lipofundin will be purchased by the co-ordinating centre pharmacy by the usual route.

For anakinra and its equivalent placebo, participants will be issued a pharmacy box with individual syringes in syringe holders, and a sharps bin. Each syringe will be labelled day 1 (already administered) up to day 5. Day 1 treatment will be administered after baseline assessment and investigations.

For peer review only - http://bmjopen.bmj.com/site/about/guidelines.xhtml

The participant will be taught to self-administer the sub-cutaneous injection. Participant will then attend on day 2 for assessment of self-injection and subsequently will undertake self-injection themselves on day 3, 4 and 5. Participants unable to self-inject will receive their injections from a carer, family member, or district nurse.

Intramuscular methylprednisolone or lipofundin syringes will be prepared by a member of the research team not involved with the trial assessments (i.e. un-blinded research nurse).

Modifications

Injection sites will be assessed visually on day 2 and day 7, and by telephone contact from day 3 to day 6. Blood tests will be taken on day 2 and day 7 to check for neutropenia and any signs of infection. Treatment will be stopped in the event of any adverse reaction during subcutaneous injections, oral prednisolone 35mg po for five days will be used as rescue treatment if symptoms of acute attack persist. Side-effects to methylprednisolone such as worsening odeama and uncontrolled diabetes (if diabetic) will be monitored and reported as adverse events.

Adherence

Participants will be contacted everyday up to from day 3 to day 7, to remind them of injection up to day 5 and of diary entry up to day 7 (except over the weekend). Syringe holders will be accounted for and the number of syringes returned in the sharps box will be counted through the lid (not strictly) ay day 7 before permanently locked by the trial team for disposal.

Concomitant care

Rescue medications

Participants can take analgesia listed in the protocol as non-investigational medicinal product (co-dydramol, codeine phosphate, tramadol, and paracetamol) for relief as required, usage will be recorded. If participants get another flare during treatment, this will be recorded and a coarse of oral prednisolone 35mg po od for 5 days will be used for rescue treatment. Low dose colchicine 500micrograms po bd could be used in addition. Use of other immune modifying treatment or NSAIDS is prohibited.

Outcomes

This is a feasibility study. It is not powered to look for inference. Study processes will be analysed. Recruitment and retention rates and willingness of patients to be randomised will be calculated. The proportion of patients who did not meet eligibility criteria will be examined.

For peer review only - http://bmjopen.bmj.com/site/about/guidelines.xhtml

For peer review only

Adherence and compliance rates and qualitative feedback will be examined. Economic data on healthcare resource use and health-related quality of life will be collected. Safety outcome measures will be reported as mandated in a clinical trial of an investigational medicinal product protocol.

Primary endpoint/outcome

Feasibility of undertaking a definitive multicentre, double-blind, double dummy randomised controlled trial to obtain clear guidance on the safe management of acute gout attacks in patients with chronic kidney disease.

Information from proposed outcome measures for the larger study will be sought. Proposed primary outcome measures of effectiveness consist of resolution of pain i.e. time to 50% reduction and complete resolution of pain in self-assessed pain intensity in the joint most affected at baseline measure on the VAS (0-100mm) and 5-point Likert scale from baseline to 7 days' post randomisation (Day 1, Day 2, Day 3, Day 4, Day 5, Day 6, Day 7) using composite time points kept to 24 hour intervals as close as possible.

Proposed secondary outcome measures will consist of patient reported outcome measure (Day 1, Day 2, Day 3, Day 4, Day 5, Day 6 and Day 7), physician assessment of joint tenderness and swelling (Day 1, Day 2, and Day 7), assessment of activity limitation and quality of life: HAQ-DI and SF-36 at Day 1, Day 7 and 8 weeks; EQ-5D-5L at Day 1, Day 2, Day 7 and 8 weeks; LEFS at Day 1, Day 2, Day 7. Time to take rescue medication and a limited assessment of healthcare resource will be made.

Blood and urine tests renal on Day 1, Day 2, Day 7 and 8 weeks, consisting of FBC, U+Es, eGFR, glucose, CRP, serum uric acid and spot urine uric acid.

Participant timeline

Figure 2.

Sample size

This is a feasibility study and we will aim to recruit at least 16 patients in each arm of the study. The sample size was obtained from a recommendation based on the feasibility of running a parallel group design. The study will aim to enrol for 15 months.

Recruitment

Recruitment will be from secondary care, either when potential participants present with acute gout attack or if in-patients develop acute gout attack. Trial centres will be encouraged

For peer review only - http://bmjopen.bmj.com/site/about/guidelines.xhtml

to work with primary care centres to identify potential participants and give letter of invitation. We will use ethically approved advertising material in the community.

Our hospital serves a population of 350,000 and had an average of 47 patients per year over the last four years with approximately 50% of patients having chronic kidney disease (i.e. 23 patients per year). Enrolment is for 15 months from 6 centres, we will only recruit patients in working hours i.e. 9am – 5pm on Mondays to Thursdays, it would be logistically difficult to avoid delays to treatment if participants present out of hours and face-to-face assessments cannot be done at weekends due to lack of research infrastructure in most small to medium sized hospital. All hospitals should be able to have pharmacy support to provide preparation and recording of treatment administered.

Potential participants would hopefully be fully aware of study if a letter of invitation was already sent to them. Otherwise, potential participants can have up to twenty-four hours to decide if they wish to take part in the study. Analgesia that is listed can be used if required; however, baseline assessments can only occur four hours after administration of last dose, i.e. before the administration of the next dose. Treatment cannot be withheld during this period and exclusion criteria will be applied if duration of attack is longer than accepted or if contra-indicatory medication is administered.

Allocation

Sequence generation

Enrolled patients will undergo 1:1 randomisation in blocks of four. Randomisation will be undertaken using an Internet-based randomisation system within ARCTU. The ARCTU uses the TENALEA system provided by the Trans European Network for Clinical Trial Service.

Concealment mechanism

Once a patient has consented to take part in the trial, the designated staff will log in to the TENALEA web page to confirm eligibility and a random allocation will be sent to pharmacy. All investigators involved with the trial will be blinded to treatment. Patients will not be informed of their assigned treatment during the study. Pharmacy will keep a record of allocated treatment arms in the event emergency un-blinding is required.

Implementation

Anakinra and its placebo will be ready made by the manufacturer. Intramuscular methylprednisolone and its placebo (lipofundin) will be prepared by a research team member not involved with the study.

For peer review only - http://bmjopen.bmj.com/site/about/guidelines.xhtml

Study labels will be provided to preserve blinding. Anakinra and its equivalent placebo will be provided by the manufacturer, one allocation is known, pharmacy will use label stating "Anakinra or placebo" using standard operating procedure. Five individual syringes labelled 1 to 5 will be placed individual transparent syringe holders and packed for dispensing with a local pharmacy label. For intramuscular methylprednisolone (depo-Medrone) and lipofundin, a member of the study team not involved with the trial will draw the treatment and label with a study label stating "Depomedrone or placebo," using a standard operating procedure.

Blinding

All trial investigators will be blinded to the intervention. Pharmacy will keep a record of treatments administrated. All trial participants, care providers and outcome assessors will be blinded to the treatment. Each treatment has its equivalent placebo to ensure blinding in maintained throughout study.

Emergency Un-blinding

The treatment code for a participant can be broken by any clinician either directly or via contact of the PI, allocation lists are available to the site pharmacy will provide 24-hour cover. Failing that, the central pharmacy can access allocation list. Where possible the local investigator should aim to discuss the need for un-blinding with the co-ordinating investigator and blind should be endeavoured to be preserved to relevant research staff (data collection, analysis, and interpretation). The co-ordinating investigator is responsible for pharmacovigilance management and reporting.

METHODS: DATA COLLECTION, MANAGEMENT, ANALYSIS

Data collection methods

Data collection will be by electronic clinical research forms (eCRF), these can be printed out if required for convenience of data collection (and act as source document) and then data inputted onto eCRF. Data can also be inputted direct onto eCRF and the completed eCRF can be printed out to act as source documents.

Training plans

Study investigators must be aware of the classification criteria for gout. For the purposes of this study, each principal investigator will be asked to go through a presentation that will be available online and asked to attend study days where concerns will be raised.

Quality control of data and results

For peer review only - http://bmjopen.bmj.com/site/about/guidelines.xhtml

Entered data will continuously reviewed by the data manager and any gaps in data will be fed back to the centre trial team. Routine blood results will be taken from the NHS system. Samples for additional testing will be transported as per standard operating protocol to the co-ordinating centre.

Participant Retention and Withdrawal

Enrolled participants will be contacted daily (weekdays) by the study team for a week. Participants may withdraw from the study for any reason at any time. The investigator may also withdraw participants from the study to protect their safety and / or if they are unwilling or unable to comply with required study procedures after discussion with the chief investigator. Participants who ask to stop study treatment, become pregnant or develop a serious infection (necessitating intravenous anti-biotic treatment and hospital admission) should continue to attend all follow-up study. Participants who withdraw their consent at any point will be asked of their wish regarding the use of data collected up to the point of withdrawal. Participants who do not attend follow-up assessment after site staff have attempted to contact the patient at least twice, e.g. by telephone may be considered for withdrawal, the data already collected for them may be used and therefore needs returning in the usual manner. The PI responsible for a patient may choose to withdraw a patient from a trial for appropriate medical reasons, be they individual adverse events or new information gained about a treatment.

Data management

Information from the patient diary and functional assessments will be entered onto an electronic CRF. Data will be entered as it is possible to do so i.e. day 1, day 2, day 7 and 2 months. Referential data rules, valid values and range checks will be support by the data entry software. The data manager will review the data being sent at regular intervals and report back to the centre if there is any discrepancy. All forms, hard drives and storage devices related to the study will be kept in locked cabinets. Access to the study data will be restricted; a password system will be used to control access. Data will be stored at MACRO database.

Study status reports

Regular updates of the study will be sent to the principle investigators via email and will be posted on the study website.

Statistical methods

For peer review only

The sample size will be too small for estimates to have adequate precision, and some methods will not be technically possible for some patterns of missing values. To make use of data obtained, comparison of groups with the use of time-to-event ("survival") analysis will be undertaken where relevant. Comparison of continuous outcome measures will be undertaken with repeated measures analysis of variance with permutation tests. Two-sample comparisons of means will be by the two-sample permutation test. The association between the intervention and categorical variables will be tested using Fisher's exact test.

Economic analysis

Economic evaluation

Formal economic evaluation looking at the incremental cost-effectiveness of Anakinra compared to the comparator is not planned as this is a feasibility study. We will collect relevant data on resource use (costs) and health-related quality of life to inform subsequent large scale study. Key drivers of costs, resource allocation and scalability will be determined.

Qualitative Analysis

Twelve participants who give their specific informed consent for participation in the qualitative study will selected for an interview around their last eight-week follow-up. The interview will cover areas such as how participants became involved with the trial, the information that was provided for them, the process of obtaining informed consent and any areas that felt was not adequately covered or could have been undertaken differently.

Data monitoring

The Trial Steering Committee (TSC) will consist of the CI, trial co-ordinator, funding co-applicants and two members of patient group will bring independent representation. The TSG will take on some aspects of pharmacovigilance. There will be no data monitoring committee as this is a small feasibility study with a short duration and short follow-up, and pharmacovigilance is a component of the study. The Trial Management Group (TMG) will consist of the CI, co-CI, funding co-applicants, representative of the clinical trials units including trial co-ordinator, data manager and statistician. They should meet every two months to ensure all practical details of the trial are progressing well and adequate targets are met. The patient group consists of patients (and family) from Southend University hospital with chronic kidney disease and suffer from acute gout attacks.

Interim analysis

For peer review only - http://bmjopen.bmj.com/site/about/guidelines.xhtml

There will be no interim analysis as this is a feasibility study, the study procedures will be regularly reviewed at the trial steering committee.

Harms

Reporting of adverse events will start at the point of consent and reporting for adverse reaction starts at first IMP dose until 7 days' post treatment. At each contact with the subject during the treatment period, the investigator must seek information on adverse events by specific questioning and, as appropriate, by examination. All clearly related signs, symptoms, and abnormal diagnostic procedures should be recorded using the NCI CTCAE v4.0 event terms and grading. The clinical course of each event should be followed until resolution or stabilisation.

Reporting will follow local operating procedure and study protocol with oversight from the co-ordinating centre. Non-serious adverse events or reactions will be recorded in the study file with follow-up; and reported for review by the co-ordinating investigator within a month. All SAEs* / SUSARs must be recorded on the SAE form / AECRF Form and faxed to the Sponsor within 24 hours of the research staff becoming aware of the event. Any change of condition or other follow-up information should be faxed to the Sponsor as soon as it is available or at least within 24 hours of the information becoming available. Events will be followed up until the event has resolved or a final outcome has been reached. All SAEs assigned by the PI or co-ordinating investigator as both suspected to be related to IMP-treatment and unexpected will be classified as SUSARs and will be subject to expedited reporting to the REC and Medicines and Healthcare Products Regulatory Agency (MHRA). The CI will complete the CIOMS form and if warranted, an investigator alert may be issued, to inform all investigators involved in any study with the same drug (or therapy) that this serious adverse event has been reported.

Potential events/reactions reported with Anakinra consist of neutropenia, infection and less serious ones consist of injection site reactions such as erythema, pruritus and rash. Potential events/reactions with steroid treatment consist of worsening hypertension, blood sugar reading/uncontrolled diabetes and clinical signs of fluid retention. Pre-existing conditions should not be reported as AE unless the condition worsens by at least CTCAE grade during trial. The condition must be reported in the pre-treatment section of the eCRF, if symptomatic at the time of entry, or under concurrent medical conditions if asymptomatic. Given the potential of clinical events related to underlying co-morbid disease burden of potential participants, events that are recognised and expected complications of the condition are exempt from normal reporting procedure, unless they are of an unexpected severity. Common expected events in these patients may consist of worsening complications

For peer review only - http://bmjopen.bmj.com/site/about/guidelines.xhtml

of chronic kidney disease, heart failure, hypertension, diabetes complications, underlying cardiac disease with complications such as ACS, arrhythmia, hypotension, tachycardia and worsening oedema. Other exceptions to (S) AE or (S) AR reporting consist of routine treatment or monitoring of the studied indication not associated with any deterioration in condition, associated with any deterioration in condition, e.g. pre-planned hip replacement operation which does not lead to further complications, any admission to hospital or other institution for general care where there was no deterioration in condition, treatment on an emergency, outpatient basis for an event not fulfilling any of the definitions of serious as given above and not resulting in hospital admission, any death or hospitalisation due to fall or fracture, any death or hospitalisation due to exacerbation of an existing medical co-morbid condition

Auditing

The study will be subject to monitoring, inspection and audit by Anglia Ruskin Clinical Trials Unit, Southend University Hospital NHS Foundation Trust acting as the Sponsor, and other regulatory bodies to ensure adherence to Good Clinical Practice (GCP).

Ethics and Dissemination

The study will be conducted in accordance with the principles of Good Clinical Practice. A favourable ethical opinion was obtained from the London-Central Research Ethics Committee, reference number 15/LO/1922. Results will be published in peer-reviewed journal and disseminated at international conferences.

Protocol amendments

Protocol amendments to the protocol or study documents will be submitted to the REC and can only be implemented once approval has been obtained. Amendments to the CTA or documents that supported the original CTA application will be notified to the MHRA. Amendments will be tracked in the protocol appendix and the version of the protocol will be updated.

Consent

A clinician in the research team will obtain informed consent from potentially eligible participants. Patients who are unable to consent for themselves for suitability for the trial will not be approached as obtaining patient reported outcome measures will not be practical. If verbal translation is needed, this should be via a hospital interpreter or a personal interpreter. Telephone interpretation services are not acceptable and written material will not

For peer review only - http://bmjopen.bmj.com/site/about/guidelines.xhtml

For peer review only

be provided in various languages for this feasibility study. This study utilises questionnaire surveys that have not been validated in different languages. There is also a qualitative element to the study where lack of required language skill may prove to be difficult. Time is limited for potential participants to be considering participation and any delays in seeking consent and translation services that may prolong treatment of the patient in acute pain have also to be considered.

Written material consisting of participant information leaflet and consent documentation will be approved by the Research Ethics Committee and will be compliant with GCP, local regulatory and legal requirement. This is a feasibility study and the costs to undertake centrally commissioned translated documents may be too high. This may be something to consider for the subsequent definitive study.

There will be opportunity for the participant to ask questions to the PI or a member of the research team. Potential participants can have up to twenty-four hours to consider the information and their participation. However, treatment cannot be withheld during this period and exclusion criteria will be applied if duration of attack is longer than accepted or if contra-indicatory medication is administered.

Ancillary studies

There will be a tertiary / exploratory study where patient’s serum and urine will be stored for future analysis at the end of the study. Testing will be an extension of routine laboratory testing to look for markers of inflammation in an exploratory setting.

Confidentiality

All investigators and trial site staff must comply with the requirements of the Data Protection Act 1998 with regards to the collection, storage, processing, and disclosure of personal information and will uphold the Act’s core principles. Information with regards to study patients will be kept confidential and managed in accordance with the Data Protection Act, NHS Caldicott Guardian, The Research Governance Framework for Health and Social Care and Research Ethics Committee Approval.

Data will be stored for five years before being destroyed. The Chief Investigator in the data custodian.

Declaration of interest

The co-ordinating investigator has not obtained any personal grants, a investigator sponsored study grant of £10,000 and provision of Anakinra and its equivalent placebo is

For peer review only - http://bmjopen.bmj.com/site/about/guidelines.xhtml

For peer review only

being provided for the study by the manufacturer's. This fund will be used to cover any predominantly pharmacy and logistics costs.

Access to data

Only the data manager will have access to data, and co-ordinate access to relevant researchers. The health economist will have access to health economic data set and qualitative researcher will have access of patient demographics to obtain a broad sample.

Ancillary and post-trial care

Acute gout attack episodes will be treated as part of this trial. If participants get another acute attack of gout, they will not be eligible to entry into the study. The participating team could consider using information with regards to treatment to avoid and available treatment options, but no information will be made available prior to the end of the trial.

Dissemination policy: trial results and authorship

The trial report will be used for publications and presentation at scientific meetings. All publications and presentations will be reviewed by the TMG. Authorship will be determined per internationally agreed criteria for authorship. Funding bodies will be declared in publications. The full report will be made available directly from the chief investigator or from published material. Supplemental material linked with publication will consist of the trial protocol.

For peer review only

For peer review only - http://bmjopen.bmj.com/site/about/guidelines.xhtml

Contributorship statement

GB conceived the idea, designed the study, wrote the protocol and the manuscript. BD helped develop the design and the protocol. MA and ER helped refine the design, protocol and the manuscript. JS designed the qualitative aspect of the study. MP undertook the statistical aspect of study design. MT designed the economic evaluation aspect of the study. MH and TP helped developed the logistical aspects of trial involving the trial unit. CM, PH, KA, TM, SB, CP helped refine the study. All authors approved the final manuscript.

Competing interests

There are no competing interests.

Funding

This trial is being funded by the National Institute of Health Research under the Research for Patient Benefit Programme. Grant number PB-PG-0614-34090. There is an additional investigator initiated study fund of £10,000 from SOBI to help with pharmacy logistics.

Data sharing statement

This manuscript is a study protocol and as yet no data is available.

For peer review only

For peer review only - http://bmjopen.bmj.com/site/about/guidelines.xhtml

References

1) Fraser SD, Roderick PJ, Aitken G, Roth M, Mindell JS, Moon G, et al. Chronic kidney disease, albuminuria and socioeconomic status in the Health Surveys for England 2009 and 2010. J Public Health (Oxf). 2014 Dec;36(4):577-86.

2) Juraschek SP, Kovell LC, Miller ER 3rd, Gelber AC. Association of kidney disease with prevalent gout in the United States in 1988-1994 and 2007-2010. Semin Arthritis Rheum. 2013 Jun;42(6):551-61.

3) Rothenbacher D, Primatesta P, Ferreira A, Cea-Soriano L, Rodríguez LA. Frequency and risk factors of gout flares in a large population-based cohort of incident gout. Rheumatology (Oxf). 2011 May;50(5):973-81.

4) Kuo CF, Grainge MJ, Mallen C, Zhang W, Doherty M. Rising burden of gout in the UK but continuing suboptimal management: a nationwide population study. Ann Rheum Dis. 2015 Apr;74(4):661-7.

5) Annemans L, Spaepen E, Gaskin M, Bonnemaire M, Malier V, Gilbert T, Nuki G. Gout in the UK and Germany: prevalence, comorbidities and management in general practice 2000-2005. Ann Rheum Dis. 2008 Jul;67(7):960-6.

6) Keenan RT, O'Brien WR, Lee KH, Crittenden DB, Fisher MC, Goldfarb DS, et al. Prevalence of contraindications and prescription of pharmacologic therapies for gout. Am J Med. 2011 Feb;124(2):155-63.

7) Curiel RV, Guzman NJ. Challenges associated with the management of gouty arthritis in patients with chronic kidney disease: a systematic review. Semin Arthritis Rheum. 2012 Oct;42(2):166-78.

8) Jordan KM, Cameron JS, Snaith M, Zhang W, Doherty M, Seckl J, et al. British Society for Rheumatology and British Health Professionals in Rheumatology guideline for the management of gout. British Society for Rheumatology and British Health Professionals in Rheumatology Standards, Guidelines and Audit Working Group (SGAWG). Rheumatology (Oxf). 2007 Aug;46(8):1372-4.

9) Richette P, Doherty M, Pascual E, Barskova V, Becce F, Castañeda-Sanabria J, et al. Zhang W, Doherty M, Pascual E et al. 2016 updated EULAR evidence-based recommendations for the management of gout. Ann Rheum Dis. 2017 Jan;76(1):29-42.

For peer review only

10) Khanna D, Khanna PP, Fitzgerald JD, Singh MK, Bae S, Neogi T, et al. American College of Rheumatology guidelines for management of gout. Part 2: therapy and anti-inflammatory prophylaxis of acute gouty arthritis. American College of Rheumatology. Arthritis Care Res (Hoboken). 2012 Oct;64(10):1447-61.

11) Siegel LB, Alloway JA, Nashel DJ. Comparison of adrenocorticotropic hormone and triamcinolone acetonide in the treatment of acute gouty arthritis. J Rheumatol. 1994 Jul;21(7):1325-7.

12) Terkeltaub RA, Furst DE, Bennett K, Kook KA, Crockett RS, Davis MW. High versus low dosing of oral colchicine for early acute gout flare: Twenty four-hour outcome of the first multicenter, randomized, double-blind, placebo controlled, parallel-group, dose-comparison colchicine study. Arthritis Rheum. 2010 Apr;62(4):1060-8.

13) Janssens HJ, Janssen M, van de Lisdonk EH, van Riel PL, van Weel C. Use of oral prednisolone or naproxen for the treatment of gout arthritis: a double-blind, randomised equivalence trial. Lancet. 2008 May 31;371(9627):1854-60.

14) Dinarello CA. Interleukin-1 in the pathogenesis and treatment of inflammatory diseases. Blood. 2011 Apr 7;117(14):3720-32.

15) Di Giovine FS, Malawista SE, Nuki G, Duff GW. Interleukin 1 (IL 1) as a mediator of crystal arthritis. Stimulation of T cell and synovial fibroblast mitogenesis by urate crystal-induced IL 1. J Immunol. 1987 May 15;138(10):3213-8.

16) Mitha E, Schumacher HR, Fouche L, Luo SF, Weinstein SP, Yancopoulos GD, et al. Rilonacept for gout flare prevention during initiation of uric acid-lowering therapy: results from the PRESURGE-2 international, phase 3, randomized, placebo-controlled trial. Rheumatology (Oxf). 2013 Jul;52(7):1285-92.

17) Schumacher HR Jr, Evans RR, Saag KG, Clower J, Jennings W, Weinstein SP, et al. Rilonacept (interleukin-1 trap) for prevention of gout flares during initiation of uric acid-lowering therapy: results from a phase III randomized, double blind, placebo-controlled, confirmatory efficacy study. Arthritis Care Res (Hoboken). 2012 Oct;64(10):1462-70.

18) Terkeltaub RA, Schumacher HR, Carter JD, Baraf HS, Evans RR, Wang J, at al. Rilonacept in the treatment of acute gouty arthritis: a randomized, controlled clinical trial using indomethacin as the active comparator. Arthritis Res Ther. 2013 Feb;15(1): R25.

19) Schlesinger N, Mysler E, Lin HY, De Meulemeester M, Rovensky J, Arulmani U, et al. Canakinumab reduces the risk of acute gouty arthritis flares during initiation of allopurinol

For peer review only - http://bmjopen.bmj.com/site/about/guidelines.xhtml

For peer review only

treatment: results of a double-blind, randomised study. Ann Rheum Dis. 2011 Jul;70(7):1264-71.

20) So A, De Meulemeester M, Pikhlak A, Yücel AE, Richard D, Murphy V, et al. Canakinumab for the treatment of acute flares in difficult-to-treat gouty arthritis: Results of a multicenter, phase II, dose-ranging study. Arthritis Rheum. 2010 Oct;62(10):3064-76.

21) Schlesinger N, De Meulemeester M, Pikhlak A, Yücel AE, Richard D, Murphy V, et al. Canakinumab relieves symptoms of acute flares and improves health-related quality of life in patients with difficult-to-treat Gouty Arthritis by suppressing inflammation: results of a randomized, dose-ranging study. Arthritis Res Ther. 2011 Mar 25;13(2): R53.

22) Schlesinger N, Alten RE, Bardin T, Schumacher HR, Bloch M, Gimona A, et al. Canakinumab for acute gouty arthritis in patients with limited treatment options: results from two randomised, multicentre, active-controlled, double blind trials and their initial extensions. Ann Rheum Dis. 2012 Nov;71(11):1839-48.

23) U.S. Food and Drug Administration [Internet]. Maryland, US. Arthritis Advisory Committee Meeting 2011: FDA briefing document. [cited 2016 Nov 01] Available from: http://www.fda.gov/downloads/AdvisoryCommittees/CommitteesMeetingMaterials/Drugs/ArthritisAdvisoryCommittee/UCM259596.pdf

24) Chakraborty A, Van LM, Skerjanec A, Floch D, Klein UR, Krammer G, et al. Pharmacokinetic and pharmacodynamic properties of canakinumab in patients with gouty arthritis. 2013 Dec;53(12):1240-51.

25) So A, De Smedt T, Revaz S, Tschopp J. A pilot study of IL-1 inhibition by anakinra in acute gout. Arthritis Res Ther. 2007;9(2): R28.

26) Chen K, Fields T, Mancuso CA, Bass AR, Vasanth L. Anakinra's efficacy is variable in refractory gout: report of ten cases. Semin Arthritis Rheum. 2010 Dec;40(3):210-4.

27) Ghosh P, Cho M, Rawat G, Simkin PA, Gardner GC, et al. Treatment of acute gouty arthritis in complex hospitalized patients with anakinra. Arthritis Care Res 2013 Aug;65(8):1381-4.

28) Ottaviani S, Moltó A, Ea HK, Neveu S, Gill G, Brunier L, et al. Efficacy of anakinra in gouty arthritis: a retrospective study of 40 cases. Arthritis Res Ther. 2013;15(5): R123.

29) Thueringer JT, Doll NK, Gertner E. Anakinra for the treatment of acute severe gout in critically ill patients. Semin Arthritis Rheum. 2015 Aug;45(1):81-5

For peer review only - http://bmjopen.bmj.com/site/about/guidelines.xhtml

For peer review only

30) Balasubramaniam G, Almond M, Dasgupta B. Improved renal function in diabetic patients with acute gout treated with anakinra. Kidney Int. 2015 Jul;88(1):195-6.

31) Direz G, Noël N, Guyot C, Toupance O, Salmon JH, Eschard JP. Efficacy but side effects of anakinra therapy for chronic refractory gout in a renal transplant recipient with pre-terminal chronic renal failure. Joint Bone Spine. 2012 Dec;79(6):631.

32) Yang B-B, Baughman S, Sullivan J. Pharmacokinetics of anakinra in subjects with different levels of renal function. Clin Pharmacol Ther. 2003 Jul;74(1):85-94.

33) Nuki G, Bresnihan B, Bear MB, et al. Long-term safety and maintenance of clinical improvement following treatment with anakinra (recombinant human interleukin-1 receptor antagonist) in patients with rheumatoid arthritis: extension phase of a randomized, double-blind, placebo controlled trial. Arthritis Rheum. 2002 Nov;46(11):2838-46.

34) Fleischmann RM, Schechtman J, Bennett R, Handel ML, Burmester GR, Tesser J, et al. Anakinra, a recombinant human interleukin-1 receptor antagonist (r-metHuIL-1ra), in patients with rheumatoid arthritis: A large, international, multicenter, placebo-controlled trial. Arthritis Rheum. 2003 Apr;48(4):927-34.

35) Schiff MH, DiVittorio G, Tesser J, Fleischmann R, Schechtman J, Hartman S, at al. The safety of anakinra in high-risk patients with active rheumatoid arthritis: six-month observations of patients with comorbid conditions. Arthritis Rheum. 2004 Jun;50(6):1752-60.

36) Fleischmann RM, Tesser J, Schiff MH, Schechtman J, Burmester GR, Bennett R, et al. Safety of extended treatment with anakinra in patients with rheumatoid arthritis. J Rheumatol. 2006 Feb;33(2):234-43.

37) Bresnihan B, Alvaro-Gracia JM, Cobby M, Doherty M, Domljan Z, Emery P, et al. Treatment of rheumatoid arthritis with recombinant human interleukin-1 receptor antagonist. Arthritis Rheum. 1998 Dec;41(12):2196-204.

38) European Medicines Agency [Internet]. London, UK. Annex 1 Summary of product characteristics. [cited 2016 Nov 01] Available from: http://www.ema.europa.eu/docs/en_GB/document_library/EPAR_-_Product_Information/human/000363/WC500042310.pdf

39) Hung AM, Ellis CD, Shintani A, Booker C, Ikizler TA. IL-1β receptor antagonist reduces inflammation in hemodialysis patients. J Am Soc Nephrol. 2011 Mar;22(3):437-42.

For peer review only

40) Fisher CJ Jr, Dhainaut JF, Opal SM, Pribble JP, Balk RA, Slotman GJ, et al. Recombinant human interleukin 1 receptor antagonist in the treatment of patients with sepsis syndrome. Results from a randomized, double-blind, placebo-controlled trial. Phase III rhIL-1ra Sepsis Syndrome Study Group. JAMA. 1994 Jun 15;271(23):1836-43.

41) Balasubramaniam G, Almond M, Borg F, Dasgupta B. Treatment of an Acute Gout Flare in Patients with Kidney Disease: A Questionnaire Survey. Rheumatology 2015 Apr; 54(suppl 1): i202-i203.

42) Pyne D, Ioannou Y, Mootoo R, Bhanji A. Intra-articular steroids in knee osteoarthritis: a comparative study of triamcinolone hexacetonide and methylprednisolone acetate. Clin Rheumatol. 2004 Apr;23(2):116-20.

43) Neogi T, Jansen TL, Dalbeth N, Fransen J, Schumacher HR, Berendsen D, et al. 2015 Gout Classification Criteria. An American College of Rheumatology/ European League Against Rheumatism Collaborative Initiative. Arthritis Rheum. 2015 Oct;67(10):2557-2568.

For peer review only

Tables

Box 1 ASGARD Inclusion criteria

Inclusion criteria

Patients who meet inclusion criteria will be approached by a study investigator. Consent for participation will be sought. Upon gaining consent, the investigator will go through exclusion criteria.

1. Subjects capable of giving informed consent.
2. Male or non-pregnant, non-nursing female
3. ≥ 18 years of age
4. eGFR < 60mls/min/1.73m^2 and ≥ 30mls/min/1.73m^2 as calculated using serum creatinine and modified MDRD formula or CKD-EPI, on two occasions at least two months apart with one being in the last six months. Creatinine at time of presentation can be used unless participant has an acute kidney injury as defined by serum creatinine rise by ≥ 26µmol/L within 48 hours or ≥ 1.5-fold rise from baseline value.
5. Diagnosis of acute gout arthritis as defined by the American College of Rheumatology 1977 preliminary criteria.
6. Gout attack less ≤ 36 hours
7. Baseline pain intensity greater than or equal to 50mm on the 0-100 mm VAS. In the case of multiple joints (≤ 3), the most affected joint will be assessed.

For peer review only - http://bmjopen.bmj.com/site/about/guidelines.xhtml

Box 2 ASGARD Exclusion criteria

Exclusion criteria

1. Treatment with colchicine in the last week.

2. Initiation of or change to dose of NSAIDS in the last week. NSAID use is not an exclusion criteria if taken at a stable dose for at least four weeks.

3. Initiation of or change to dose of systemic steroids in the last week. Systemic steroid use is no an exclusion criteria if taken at an equivalent dose of 10mg Prednisolone or less for the last four weeks.

4. Polyarticular gout, i.e. affecting four or more 4 joints

5. Rheumatoid arthritis, evidence/suspicion of infectious/septic arthritis, or other acute inflammatory arthritis such as systemic lupus erythematosus, mixed connective tissue disease, scleroderma, polymyositis, or significant systemic involvement secondary to rheumatoid arthritis.

6. Con-current immunosuppression/immunomodulatory treatment (Calcineurin inhibitor, anti-proliferative or biologic) therapy for other reason i.e. organ transplant.

7. Prior history or current inflammatory joint disease other than gout (e.g. rheumatoid arthritis (RA), reactive arthritis, psoriatic arthritis, seronegative spondyloarthropathy, Lyme disease).

8. Current active malignancy (with the exception of basal cell or squamous cell carcinoma of the skin, cervical intraepithelial neoplasia and non-metastatic/advanced prostate cancer).

9. Any patients with contra-indication to intramuscular injection such as coagulopathy or thrombocytopenia (Platelet count<100 x 109/L (100,000/mm^3)).

10. Abnormal liver function tests: Total bilirubin>upper limit of normal, Alanine aminotransferase (ALT) or Aspartate Aminotransferase (AST) >2 times upper limit of normal.

11. Haemoglobin < 85g/L (8.5 g/dL)

12. White blood cell (WBC) count<1.5 x 109/L (1000/mm3), absolute neutrophil count<1.5 x 10^9/L (1000/mm^3)

For peer review only - http://bmjopen.bmj.com/site/about/guidelines.xhtml

13. Evidence of serious active infection requiring intra-venous (IV) anti-biotic drug administration within last four weeks. Potential participants who were empirically treated with a course of intravenous antibiotics for a presumptive diagnosis of an infection may be eligible where the treating medical team has subsequently excluded an active infection and consequently discontinued antibiotic therapy at least four hours before randomisation. These potential participants can be discussed with the investigator where clarity is required.

14. Evidence of uncontrolled concomitant cardiovascular, nervous system, pulmonary (including obstructive pulmonary disease), hepatic, endocrine (including uncontrolled diabetes) or gastrointestinal disease. Potential participants who have active concomitant disease can only be eligible after discussion and agreement with the treating medical team.

15. Known positive hepatitis B virus surface antigen (HBsAg), hepatitis C (HCV) antibody or HIV.

16. Females of child bearing potential who are not willing to use highly effective birth control methods from the time of consent to one week after treatment discontinuation. Highly effective method of contraception (hormonal or barrier method of birth control; abstinence) consist of:

• Combined (estrogen and progestogen containing) hormonal contraception associated with inhibition of ovulation; oral, intravaginal, transdermal.

• Progestogen-only hormonal contraception associated with inhibition of ovulation; oral, injectable, implantable.

• Intrauterine device (IUD) or intrauterine hormone-releasing system (IUS)

• Bilateral tubal occlusion or vasectomised partner

• Sexual abstinence, defined as true abstinence when it is in line with the preferred and usual lifestyle of the participant. Periodic abstinence (e.g. calendar, ovulation, sympthothermal, post-ovulation) and withdrawal methods are not acceptable methods of contraception.

17. Men who are sexually active with a female partner who are not willing to use highly effective birth control methods from the time of consent to one week after treatment discontinuation.

18. Females of childbearing potential must have a negative pregnancy test (highly

For peer review only

For peer review only - http://bmjopen.bmj.com/site/about/guidelines.xhtml

sensitive urine or serum pregnancy test after a confirmed menstrual period) within 7 days prior to treatment initiation. Subjects are considered not of child bearing potential if they are surgically sterile (i.e. they have undergone a hysterectomy, bilateral tubal ligation, or bilateral oophorectomy) or they are postmenopausal.

19. Females must not be breastfeeding.

20. Patients who have had treatment as part of this trial cannot have repeat treatment for another flare as part of the trial.

21. Patients with allergies to excipients of IMPs: citric acid, anhydrous, sodium chloride, disodium edetate dehydrate, polysorbate 80, sodium hydroxide. Hypersensitivity to E. Coli derived proteins, egg proteins and soy proteins. Patients with a latex allergy are also not eligible as the inner needle cover of the pre-filled syringe contains dry natural rubber (a derivative of latex).

For peer review only

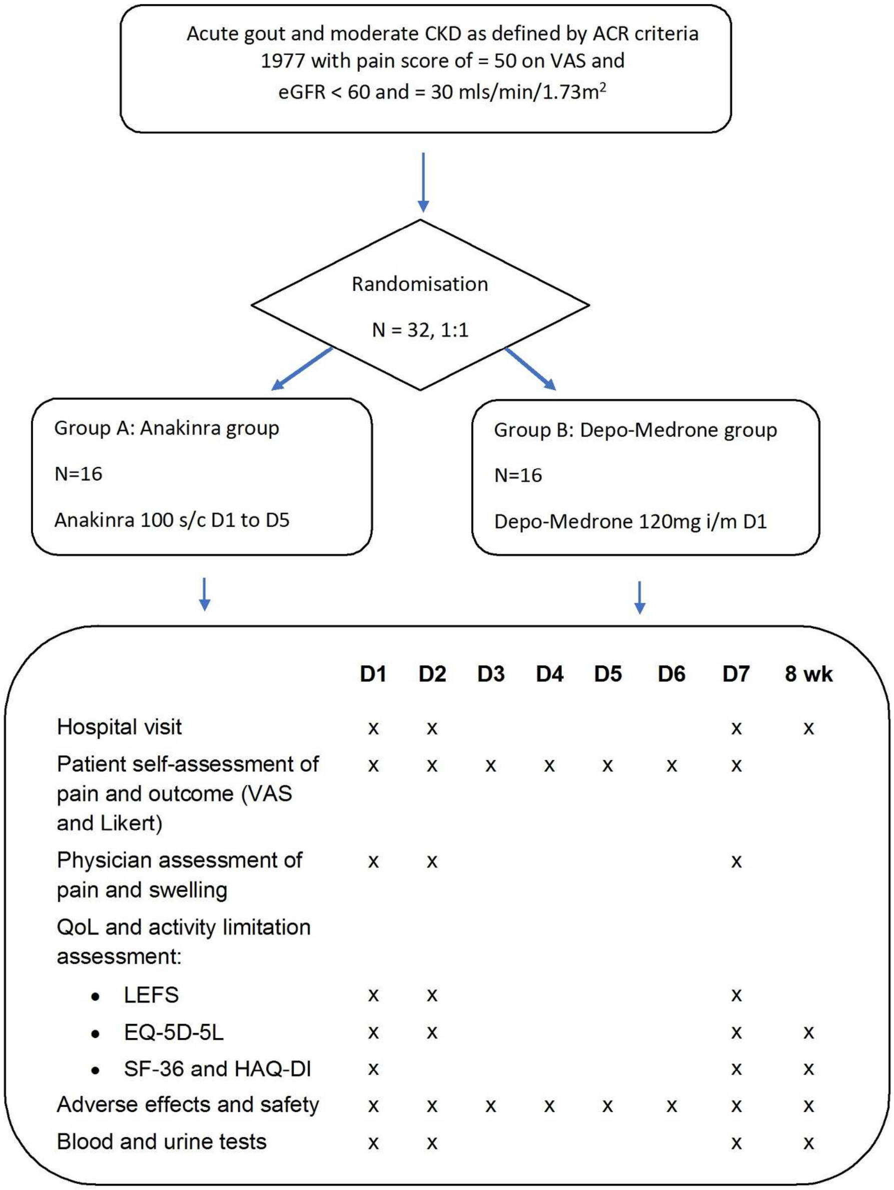

Figure 1. Trial flowchart

234x316mm (192 x 192 DPI)

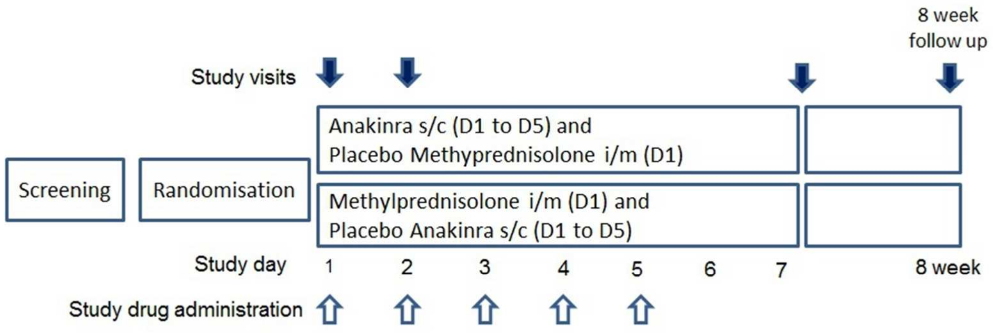

Figure 2. Participant timeline

118x66mm (192 x 192 DPI)

For peer review only - http://bmjopen.bmj.com/site/about/guidelines.xhtml

Health Research Authority

CTIMP Protocol

- **This protocol has regard for the HRA guidance and order of content**

For peer review only

Version 1.4 February 2017

For peer review only - http://bmjopen.bmj.com/site/about/guidelines.xhtml

Health Research Authority

Anakinra vs. Steroids for the Treatment of Gout Attacks in Renal Patients (ASGARD): A Feasibility Study

EudraCT no. 2015-001787-19

A study to determine the feasibility of undertaking a definitive randomised multi-centre, double-blind, double-dummy controlled study of a novel agent Anakinra, an IL-1 receptor antagonist vs. intramuscular methylprednisolone (Depo-Medrone) for acute gout attacks in patients with moderate chronic kidney disease

For peer review only

For peer review only - http://bmjopen.bmj.com/site/about/guidelines.xhtml

NHS
Health Research Authority

Anakinra vs. Steroids for the Treatment of Gout Attacks in Renal Patients (ASGARD): A Feasibility Study

EudraCT no. 2015-001787-19

Anakinra vs. Steroids for the treatment of Gout Attacks in Patients with Renal Disease (ASGARD): A Feasibility study

Version 1.4

01 February 2017

For peer review only

RESEARCH REFERENCE NUMBERS

TRIAL REGISTRY NUMBER AND DATE

IRAS Number: 160630

EudraCT Number: 2015-001787-19

US NCT / Clinical trials.gov Number: NCT02578394

WHO Universal Trials Reference Number: U1111-1175-1977

SPONSORS Number: P0850

FUNDERS Number: NIHR GRANT PB-PG-0614-34090

Health Research Authority

Anakinra vs. Steroids for the Treatment of Gout Attacks in Renal Patients (ASGARD): A Feasibility Study

EudraCT no. 2015-001787-19

SPONSOR

SOUTHEND UNIVERSITY HOSPTAL NHS FOUNDATION TRUST

CONTACT ADDRESS:

PRITTLEWELL CHASE, WESTCLIFF-ON-SEA, SOUTHEND, ESSEX SS0 0RY

For peer review only

For peer review only - http://bmjopen.bmj.com/site/about/guidelines.xhtml

Health Research Authority

Anakinra vs. Steroids for the Treatment of Gout Attacks in Renal Patients (ASGARD): A Feasibility Study

EudraCT no. 2015-001787-19

SIGNATURE PAGE

The undersigned confirm that the following protocol has been agreed and accepted and that the Chief Investigator agrees to conduct the trial in compliance with the approved protocol and will adhere to the principles outlined in the Medicines for Human Use (Clinical Trials) Regulations 2004 (SI 2004/1031), amended regulations (SI 2006/1928) and any subsequent amendments of the clinical trial regulations, GCP guidelines, the Sponsor's SOPs, and other regulatory requirements as amended.

I agree to ensure that the confidential information contained in this document will not be used for any other purpose other than the evaluation or conduct of the clinical investigation without the prior written consent of the Sponsor

I also confirm that I will make the findings of the study publicly available through publication or other dissemination tools without any unnecessary delay and that an honest accurate and transparent account of the study will be given; and that any discrepancies from the study as planned in this protocol will be explained.

For and on behalf of the Study Sponsor:

Signature: .... Date: ....../....../......

Name (please print):

....

Position:

....

Chief Investigator:

Signature: .... Date: ....../....../......

Name: (please print):

....

Statistician:

For peer review only - http://bmjopen.bmj.com/site/about/guidelines.xhtml

NHS
Health Research Authority

Anakinra vs. Steroids for the Treatment of Gout Attacks in Renal Patients (ASGARD): A Feasibility Study

EudraCT no. 2015-001787-19

Signature:
…………………………………………………………………………………………

Name: (please print):

…………………………………………………………………………………………

Position:
…………………………………………………………………………………………

KEY TRIAL CONTACTS

Chief Investigator	Gowrie Balasubramaniam<br>Consultant Nephrologist and General Physician<br>Southend University Hospital, Westcliff-on-Sea<br>Essex SS0 0RY<br>Phone: +44 7843563739 (mobile); 01702 435555<br>E-mail: gowrie@doctors.net.uk<br>g.balasubramaniam@southend.nhs.uk<br>Fax: 01702 508595
Trial Co-ordinator	Arit Udoh<br>Anglia Ruskin University<br>Post Graduate Medical Institute Clinical Trials Unit<br>Bishops Hall Lane, Chelmsford<br>Essex CM1 1SQ<br>Phone: 0845 196 4936<br>E-mail: trisha.parker@anglia.ac.uk
Sponsor	Craig MacKerness<br>Research and Development Manager<br>Southend University Hospital, Westcliff-on-Sea<br>Essex SS0 0RY<br>E-mail: Craig.Mackerness@southend.nhs.uk<br>Phone: 01702 385089<br>Fax: 01702 508595
Funder(s)	National Institute of Healthcare Research<br>Central Commissioning Facility

For peer review only - http://bmjopen.bmj.com/site/about/guidelines.xhtml

Health Research Authority

Anakinra vs. Steroids for the Treatment of Gout Attacks in Renal Patients (ASGARD): A Feasibility Study

EudraCT no. 2015-001787-19

	Grange House<br>15 Church Street<br>Twickenham TW1 3NL<br>Phone: 020 8843 8057<br>Email: rfpb@nihr-ccf.org.uk
Clinical Trials Unit	Anglia Ruskin University<br>Post Graduate Medical Institute Clinical Trials Unit<br>Bishops Hall Lane, Chelmsford<br>Essex CM1 1SQ<br>Phone: 0845 196 4936
Key Protocol Contributors	Gowrie Balasubramaniam<br>Consultant Nephrologist and General Physician<br>Southend University Hospital, Westcliff-on-Sea<br>Essex SS0 0RY<br>Phone: +44 7843563739 (mobile); 01702 435555 (work)<br>E-mail: gowrie@doctors.net.uk<br>g.balasubramaniam@southend.nhs.uk<br>Fax: 01702 508595<br><br>Bhaskar Dasgupta<br>Professor of Rheumatology<br>Southend University Hospital, Westcliff-on-Sea<br>Essex SS0 0RY<br>Phone: 01702 385856<br>E-mail: bhaskar.dasgupta@southend.nhs.uk<br>Fax: 01702 508595
Statistician	Jufen Zhang<br>Medical Statistician<br>Anglia Ruskin University<br>Post Graduate Medical Institute<br>Faculty of Health and Social Care<br>Bishops Hall Lane, Chelmsford<br>Essex CM1 1SQ<br>Phone: 0845 196 4936

For peer review only - http://bmjopen.bmj.com/site/about/guidelines.xhtml

Health Research Authority

Anakinra vs. Steroids for the Treatment of Gout Attacks in Renal Patients (ASGARD): A Feasibility Study

EudraCT no. 2015-001787-19

	E-mail: Jufen.Zhang@anglia.ac.uk
Health Economist	David Turner<br>Health Economist<br>Norwich Medical School<br>University of East Anglia<br>Norwich Research Park<br>Norwich, NR4 7TJ<br>Phone: 01603 593960<br>E-mail: David.A.Turner@uea.ac.uk
Trials pharmacists	Amiirah Ropun, Inisa Idress and Laura Wilson<br>Trial Pharmacists<br>Southend University Hospital, Westcliff-on-Sea<br>Essex SS0 0RY<br>Phone: 01702 435555<br>E-mail: Amiirah.Ropun@southend.nhs.uk; Inisa.Idress@southend.nhs.uk; Laura.Wilson@southend.nhs.uk<br>Fax: 01702 508595
Committees	ASGARD Steering Committee<br>Southend University Hospital, Westcliff-on-Sea<br>Essex SS0 0RY<br>Phone: 01702 385856<br>E-mail: asgardsteering@hotmail.com<br>Fax: 01702 508595<br>ASGARD Trial Management Committee<br>c/o Dr G Balasubramaniam<br>Southend University Hospital, Westcliff-on-Sea<br>Essex SS0 0RY<br>Phone: 01702 435555<br><br>ASGARD Patient Committee<br>Southend University Hospital, Westcliff-on-Sea<br>Essex SS0 0RY<br>Phone: 01702 435555<br>E-mail: asgardsteering@hotmail.com

For peer review only - http://bmjopen.bmj.com/site/about/guidelines.xhtml

Health Research Authority

Anakinra vs. Steroids for the Treatment of Gout Attacks in Renal Patients (ASGARD): A Feasibility Study

EudraCT no. 2015-001787-19

For peer review only

For peer review only - http://bmjopen.bmj.com/site/about/guidelines.xhtml

Anakinra vs. Steroids for the Treatment of Gout Attacks in Renal Patients (ASGARD): A Feasibility Study

EudraCT no. 2015-001787-19

TRIAL SUMMARY

Trial Title	A Multi-Centre, Randomised Controlled Trial of Anakinra vs. Steroids for Gout Attacks in patients with Moderate Renal Disease (ASGARD): a Feasibility Study	
Short title	Anakinra vs. Steroids for Gout Attacks in Patients with Kidney Disease (ASGARD): A Feasibility Study	
Internal reference no.	P0850	
Clinical Phase	Feasibility Study	
Trial Design	Multi-centre, Randomised Controlled Trail	
Trial Participants	Patients with Acute Gout Attacks and Moderate Kidney Disease (eGFR < 60 to ≥ 30)	
Planned Sample Size and accrual rate	32. 2 patients per month across all sites. Large centres should be able to recruit one case per months and smaller centre may recruit less frequently.	
Administration Schedule	Group A (Experimental)<br><br>Anakinra 100mg s/c injection D1 to D5 and placebo i/m methylprednisolone (Lipofundin) injection at D1	Group B (Active comparator)<br><br>Intramuscular methylprednisolone (Depo-Medrone) 120mg i/m injection at D1 and placebo Anakinra s/c D1 to D5
Treatment duration	5 days (D1 to D5)	
Follow up duration	8 weeks	
Planned Trial Period	22 months	
	Objectives	Outcome Measures
Primary	Feasibility for larger definitive trial and safety	Test recruitment, retention rates, willingness to be randomised, eligibility criteria, adherence / compliance rates, economic data, and scalability. Safety and quantitative assessment of study involvement.

For peer review only

For peer review only - http://bmjopen.bmj.com/site/about/guidelines.xhtml

Secondary	Proposed measures to comparing effectiveness of treatment arms	Time to 50% reduction and complete resolution of pain (self-assessment) on VAS (0-100mm) and 5-point Likert scale (D1 to D7)<br><br>Physician assessment of joint tenderness and swelling (D1, D2, and D7)<br><br>Patient reported outcome measure (D1 to D7).<br><br>Patient assessment of activity limitation and quality of life: HAQ-DI and SF-36 (D1, D7 and 8 weeks);<br><br>EQ-5D-5L at D1, D2, D7 and 8 weeks;<br><br>LEFS at D1, D2, D7.<br><br>Time to take rescue medication<br><br>Change in renal function by eGFR, CRP, uric acid and urine uric acid (D1, D2 , D7, week 8)<br><br>Healthcare resource use at 8 weeks.
Tertiary (exploratory)	Inflammatory changes in blood and urine	Changes in blood and urine inflammatory changes markers. Epigenetic changes with treatment (D1, D2, and D7, week 8).
Investigational Medicinal Product(s)	Anakinra, Interleukin-1 receptor antagonist<br><br>Intramuscular methylprednisolone (Depo-Medrone)<br><br>Lipofundin MCT/LCT 10% (placebo for Intramuscular methylprednisolone)	
Formulation, Dose, Route of Administration	Anakinra: Pre-filled syringes 0.67mLs, 100mg s/c for 5 days	

For peer review only - http://bmjopen.bmj.com/site/about/guidelines.xhtml

NHS Health Research Authority

Anakinra vs. Steroids for the Treatment of Gout Attacks in Renal Patients (ASGARD): A Feasibility Study

EudraCT no. 2015-001787-19

	Intramuscular methylprednisolone acetate (Depo-Medrone): 3mL injection containing 120mg/3mL drawn from 3mL vial.<br><br>Lipofundin MCT/LCT 10%: 3mL injection drawn from 100mL bottle for i/m injection

FUNDING AND SUPPORT IN KIND

**FUNDER(S)**<br><br>(Names and contact details of ALL organisations providing funding and/or support in kind for this trial)	**FINANCIAL AND NON FINANCIALSUPPORT GIVEN**
NIHR	£157,591.00
SOBI	Provision of Anakinra and equivalent placebo<br><br>£10,000

For peer review only

Health Research Authority

Anakinra vs. Steroids for the Treatment of Gout Attacks in Renal Patients (ASGARD): A Feasibility Study

EudraCT no. 2015-001787-19

ROLE OF STUDY SPONSOR AND FUNDER

The sponsor of this clinical trial is Southend University Hospital Trust. The named representative of the sponsor is the head of Research and Development. The sponsor is responsible for:

- Providing the investigators with information needed to conduct the trial properly, access to resources and support to deliver the research as proposed.
- Ensure monitoring of the trial, in conjunction with chief-investigator, the trial co-ordinator and the trial steering committee.
- Ensure that the investigation is conducted in accordance with Health Research Authority and the framework of research governance of the Department of Health and Good Medical Practice.

The sponsor has to be satisfied there is agreement on appropriate arrangement to:

- Record, report and review significant developments as the research proceeds, particularly those which outline the safety of individuals at risk; and to
- Approve any modifications to the design, obtain any regulatory authority required, implement them, and make them known.
- Patient withdrawal

The sponsor will undertake pharmacovigilance for this study. The sponsor will monitor adverse event monitoring and report at the end of the study back to the manufacturer. This is part of the clinical trial and will be published as an outcome as well.

Indemnity will be part of NHS indemnity of local centres. This is a phase 3 trial and a comparison of two treatments.

The sponsor has control over the final decision regarding the trial design, conduct, data analysis and interpretation, manuscript writing, and dissemination of results.

Role of the Funder

The funder has provided finance for the project. Reports will be made at regular intervals from the steering committee for the funder. The funder will also monitor and assess value for money for the project. Funding was approved after competitive, peer reviewed process.

For peer review only - http://bmjopen.bmj.com/site/about/guidelines.xhtml

ROLES AND RESPONSIBILITIES OF TRIAL MANAGEMENT COMMITEES/GROUPS & INDIVIDUALS

Trial Steering Committee

The Trial Steering Committee (TSC) will consist of the CI, trial co-ordinator and funding co-applicants. Members of patient group will bring independent representation. The TSG will take on some aspects of pharmacovigilance as there will be no data monitoring committee as part of this feasibility study.

Trial Management Group

The Trial Management Group (TMG) will consist of the CI, co-CI, funding co-applicants, representative of the clinical trials units including trial co-ordinator, data manager and statistician. They should meet every two months two to ensure all practical details of the trial are progressing well and adequate targets are met.

Data Monitoring Committee

This is a small feasibility study with a short duration and short follow-up. Pharmacovigilance is a component of the study but costs of running a data monitoring committee have to offset against the benefit in such a small feasibility trial, and also the minimal expected risk from previous published data where Anakinra has been used in patients with rheumatoid arthritis. Some roles of a data monitoring committee will be incorporated into the remit of the TMG. If a subsequent larger study is planned, then a data monitoring committee will be incorporated into the study.

ASGARD Patient group

The patient group consists of patients (and family) from Southend University hospital that suffer from chronic kidney disease and acute gout attacks.

For peer review only - http://bmjopen.bmj.com/site/about/guidelines.xhtml

Health Research Authority

Anakinra vs. Steroids for the Treatment of Gout Attacks in Renal Patients (ASGARD): A Feasibility Study

EudraCT no. 2015-001787-19

Protocol contributors

This study was instigated due to increasing difficulty in managing the described patient population in routine clinical practice of the chief investigator. The protocol was mainly written by the chief investigator with Professor Dasgupta, who will act as co-chief investigator. The co-investigators, Drs Almond and Harnett gave input on study design. Michael Parker is the trial statistician and has contributed to relevant aspects of the protocol. David Turner is the Health Economist and has contributed to relevant aspects of the trial protocol. The clinical trial unit, including the director, data manager and the trial co-ordinator have given input into practical aspects of the trial design.

The gout study patient group has had an input in the design of the trial and members will continue to be part of the steering committee

The initial version of the protocol was rejected by the funding panel. Comments from reviewers were incorporated into the revised protocol. The funder has no control over any final decisions regarding the trial.

KEY WORDS:

Acute, Gout, Kidney, Intramuscular Methylprednisolone, Interleukin-1, Randomised-Controlled Trial

For peer review only - http://bmjopen.bmj.com/site/about/guidelines.xhtml

Health Research Authority

Anakinra vs. Steroids for the Treatment of Gout Attacks in Renal Patients (ASGARD): A Feasibility Study

EudraCT no. 2015-001787-19

LIST of CONTENTS

GENERAL INFORMATION	Page No.
TITLE PAGE	2
RESEARCH REFERENCE NUMBERS	3
SIGNATURE PAGE	5
KEY TRIAL CONTACTS	6
TRIAL SUMMARY	10
FUNDING	11
ROLE OF SPONSOR AND FUNDER	12
ROLES & RESPONSIBILITIES OF TRIAL MANAGEMENT COMMITTEES, GROUPS AND INDIVIDUALS	13
LIST of CONTENTS	15
LIST OF ABBREVIATIONS	17
TRIAL FLOW CHART	19
**SECTION**	
1. BACKGROUND	22
2. RATIONALE	24
3. OBJECTIVES AND OUTCOME MEASURES/ENDPOINTS	26
4. TRIAL DESIGN	30
5. STUDY SETTING	30
6. ELIGIBILITY CRITERIA	30
7. TRIAL PROCEDURES	33
8. TRIAL MEDICATION	44

For peer review only - http://bmjopen.bmj.com/site/about/guidelines.xhtml

Health Research Authority

Anakinra vs. Steroids for the Treatment of Gout Attacks in Renal Patients (ASGARD): A Feasibility Study

EudraCT no. 2015-001787-19

9. PHARMACOVIGILANCE	60
10. STATISTICS AND DATA ANALYSIS	68
11. DATA HANDLING	72
12. MONITORING, AUDIT & INSPECTION	75
13. ETHICAL AND TRIAL ADMINISTRATION	77
14. DISSEMINATION POLICY	81
15. REFERENCES	82
16. APPENDICIES	85

For peer review only - http://bmjopen.bmj.com/site/about/guidelines.xhtml

LIST OF ABBREVIATIONS

Define all unusual or 'technical' terms related to the trial. Add or delete as appropriate to your trial. Maintain alphabetical order for ease of reference.

AE	Adverse Event
AR	Adverse Reaction
CA	Competent Authority
CI	Chief Investigator
CKD	Chronic Kidney Disease
CRF	Case Report Form
CRO	Contract Research Organisation
CTA	Clinical Trial Authorisation
CTIMP	Clinical Trial of Investigational Medicinal Product
DMC	Data Monitoring Committee
DSUR	Development Safety Update Report
EC	European Commission
EMEA	European Medicines Agency
EQ-5D-5L	EuroQol-5 Dimension-5 Level
EU	European Union
EUCTD	European Clinical Trials Directive
EudraCT	European Clinical Trials Database
EudraVIGILANCE	European database for Pharmacovigilance
GC	Glucocorticoid
GCP	Good Clinical Practice
GMP	Good Manufacturing Practice
HAQ-DI	Health Assessment Questionnaire Disability Index
IB	Investigator Brochure
ICF	Informed Consent Form
ICH	International Conference on Harmonisation of technical requirements for registration of pharmaceuticals for human use.
IDMC	Independent Data Monitoring Committee
IL-1	Interleukin-1
IL-1 RA	Interleukin-1 Receptor Antagonist
IMP	Investigational Medicinal Product
IMPD	Investigational Medicinal Product Dossier
ISF	Investigator Site File

For peer review only

For peer review only - http://bmjopen.bmj.com/site/about/guidelines.xhtml

ISRCTN	International Standard Randomised Controlled Trials Number
LEFS	Lower Extremity Functional Scale
MA	Marketing Authorisation
MHRA	Medicines and Healthcare products Regulatory Agency
MS	Member State
NHS R&D	National Health Service Research & Development
NIMP	Non-Investigational Medicinal Product
PI	Principal Investigator
PIC	Participant Identification Centre
PIS	Participant Information Sheet
QA	Quality Assurance
QC	Quality Control
QoL	Quality of Life
QP	Qualified Person
RCT	Randomised Control Trial
REC	Research Ethics Committee
SAE	Serious Adverse Event
SAR	Serious Adverse Reaction
SDV	Source Data Verification
SF 36	Short Form (36) Health Survey
SOP	Standard Operating Procedure
SmPC	Summary of Product Characteristics
SSI	Site Specific Information
SUSAR	Suspected Unexpected Serious Adverse Reaction
TMG	Trial Management Group
TSC	Trial Steering Committee
TMF	Trial Master File
VAS	Visual Analogue Scale

For peer review only - http://bmjopen.bmj.com/site/about/guidelines.xhtml

Health Research Authority

Anakinra vs. Steroids for the Treatment of Gout Attacks in Renal Patients (ASGARD): A Feasibility Study

EudraCT no. 2015-001787-19

TRIAL FLOW CHART

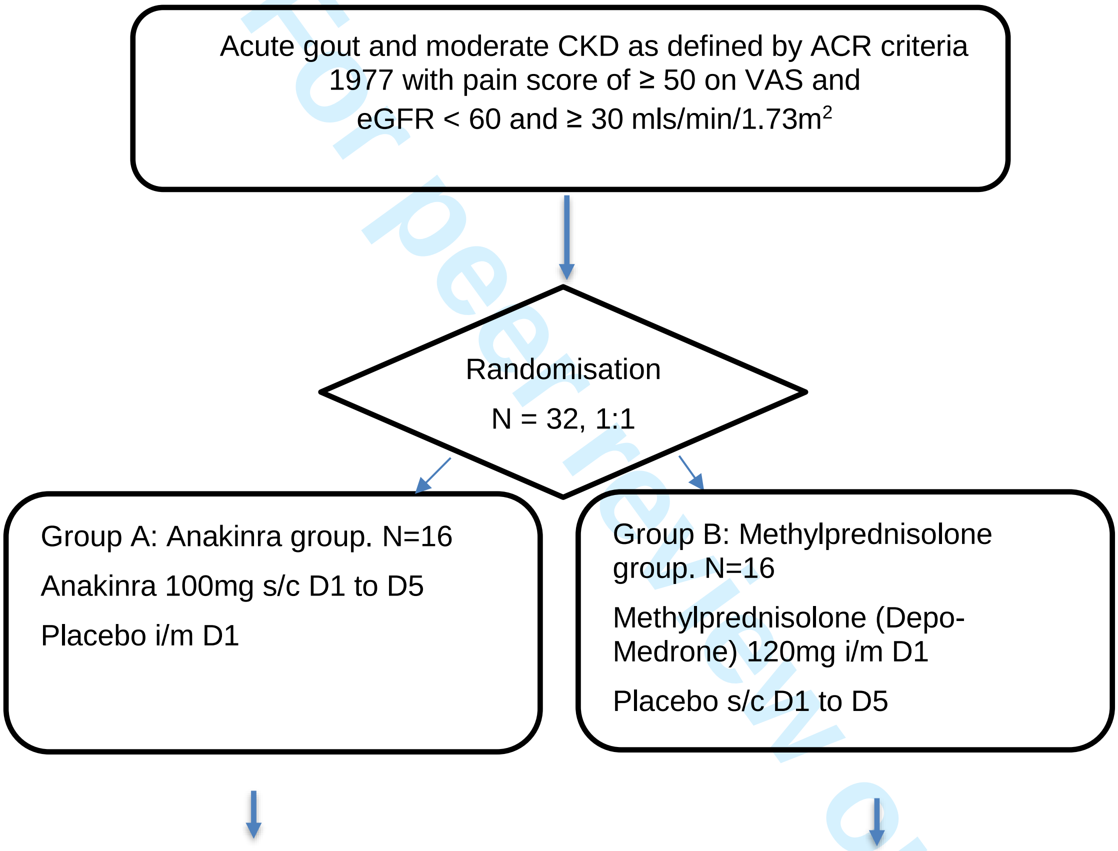

For peer review only

For peer review only - http://bmjopen.bmj.com/site/about/guidelines.xhtml

	D1	D2	D3	D4	D5	D6	D7	8 wk
Hospital visit	x	x					x	x
Patient self-assessment of pain and outcome (VAS and Likert)	x	x	x	x	x	x	x	
Physician assessment of pain and swelling	x	x					x	
QoL and activity limitation assessment:								
• LEFS	x	x					x	
• EQ-5D-5L	x	x					x	x
• SF-36 and HAQ-DI	x						x	x
Adverse effects and safety	x	x	x	x	x	x	x	x
Blood and urine tests	x	x					x	x
Interview, N=12								x
[illegible]								

SCHEDULE OF PROCEDURES

Procedures	Screening	Baseline (pre-treatment) Day 1	Day 2	Day 3	Day 4	Day 5	Day 6	Day 7	8 weeks
		Treatment phase							
			Follow up						
Informed Consent	X								
Demographics	X								
Medical History	X								
Physical examination	X								
Vital signs	X								
Blood and urine tests		X	X					X	X

For peer review only - http://bmjopen.bmj.com/site/about/guidelines.xhtml

NHS
Health Research Authority

Anakinra vs. Steroids for the Treatment of Gout Attacks in Renal Patients (ASGARD): A Feasibility Study

EudraCT no. 2015-001787-19

Concomitant medications	X	X	X	X	X	X	X	X	
Eligibility assessment	X								
Randomisation		X							
Dispensing of trial drug		X							
Drug administration		X	X	X	X	X			
Patient reported pain and outcome assessments (diary entry)		X	X	X	X	X	X	X	
Physician assessment of joint		X	X					X	
Patient assessment of activity limitation and quality of life using:									
• LEFS		X	X					X	
• EQ-5D-5L		X	X					X	X
• HAQ-DI and SF-36		X						X	X
Compliance Assessments			X	X	X	X	X	X	
Checking patient diary entry for time of injection and pain assessment (by phone or face to face)			X	X	X	X	X	X	
Diary return								X	
Check injection sites		X	X					X	

For peer review only

For peer review only - http://bmjopen.bmj.com/site/about/guidelines.xhtml

NHS
Health Research Authority

Anakinra vs. Steroids for the Treatment of Gout Attacks in Renal Patients (ASGARD): A Feasibility Study

EudraCT no. 2015-001787-19

Check returned syringes								X	
Questionnaire return (face to face or by mail)									X
Adverse events assessments (face to face or by phone)		X	X	X	X	X	X	X	X
Physician's withdrawal checklist									
Interview (12 patients)									X
Healthcare Resource use data									X

For peer review only

STUDY PROTOCOL

A study to determine the feasibility of undertaking a definitive randomised multi-centre, double-blind, double-dummy controlled study of a novel agent Anakinra, an IL-1 receptor antagonist vs. intramuscular methylprednisolone (Depo-Medrone) for acute gout attacks in patients with moderate chronic kidney disease.

1 BACKGROUND

Chronic Kidney Disease and Acute Gout

Chronic kidney disease (CKD) affects 5% of the UK population[1] and 40% of patients with CKD 3 and 4 have chronic gout[2], suggesting that 1.32 million people, predominantly older patients, have CKD and gout in the UK.

In a UK study, approximately 89.4% of patients were treated for their acute flare with non-steroidal anti-inflammatory agents (NSAIDS) which are well known to worsen kidney failure[3]. Data from secondary care showed that up to 50% of acute gout patients had CKD and 40% were given contra-indicated medications[4]. This is similar to our experience where an audit of acute gout showed half of

For peer review only - http://bmjopen.bmj.com/site/about/guidelines.xhtml

Health Research Authority

Anakinra vs. Steroids for the Treatment of Gout Attacks in Renal Patients (ASGARD): A Feasibility Study

EudraCT no. 2015-001787-19

all patients with acute gout and kidney disease were given potentially harmful medications. Mismanagement of acute gout attacks in patient with kidney disease occurs as there is no firm evidence-base[5].

Acute Gout

The incidence of acute gout attacks is 2.68 per 1000 person-years[6]. The prevalence of gout is increasing due to increasing prevalence of co-morbid conditions that are associated with hyperuricaemia such as hypertension, obesity, metabolic syndrome, diabetes and chronic kidney disease.

The lack of evidence in the literature for the use of conventional agents in patients with CKD is primarily because patients with CKD are excluded as NSAIDS are used as an active comparator in randomised controlled trials. Our survey showed that most rheumatologists in our region felt guidance in treating CKD patients is lacking (BRS poster 2015). Some guidelines continue to suggest using NSAIDS and colchicine with caution in patients with kidney disease even with a poor evidence base[7,8,9].

NSAIDS have the largest evidence base for use in acute gout but are contra-indicated in CKD. They can also worsen heart failure, hypertension, and liver failure and cause gastro-intestinal bleeding[5,10]. Colchicine is an alternative anti-inflammatory agent but all clinical trials excluded patients with moderate-to-severe CKD. It has a narrow therapeutic index, even though lower doses of colchicine can be efficacious (AGREE trial), 20% of patients still experienced diarrhoea[11]. Diarrhoea is common in patients with CKD and they are also more likely to have drug accumulation and serious side effects from toxicity such as bone marrow failure, rhabdomyolysis, pancreatitis and myopathy[5].

Glucocorticoids are an effective treatment with some evidence for use in CKD patients from older trials[12]. Triamcinolone, a long-acting synthetic corticosteroid, was shown to be as effective as adrenocorticotrophic hormone (ACTH), a hormone that increases endogenous cortisol secretion[10]. Triamcinolone is commonly used as the active comparator agent in contemporary acute gout studies world-wide.

We are using intramuscular methylprednisolone (depo-medrone) as it is the most commonly used long-acting intra-muscular agent in the UK (suggested by reviewer of initial application and from our survey). It is equivalent to Triamcinolone in other inflammatory joint conditions[13] and is the preferred UK treatment as evidence by our regional survey of Rheumatologists (presented at BRS 2015). It has been shown to be useful in the initial phase of treating rheumatoid arthritis[14] but there were concerns about long-term side-effects[15]. Intramuscular methylprednisolone is as efficacious as oral steroids with a lower cumulative dose (120mg vs.175mg) in other rheumatology conditions, and would be equivalent to the dose used in the Janssens paper[12]. This well design double-blind randomised controlled study showed that Prednisolone 35mg for 5 days was not inferior to Naproxen, although, this study recruited from primary care and excluded patients with impaired renal function (mean participant GFR was 78). Intramuscular methylprednisolone is also associated with less mineralocorticoid effect reducing the likelihood of salt and water retention in heart failure and renal failure.

The IMPs

- Anakinra (Kineret)

For peer review only - http://bmjopen.bmj.com/site/about/guidelines.xhtml

Health Research Authority

Anakinra vs. Steroids for the Treatment of Gout Attacks in Renal Patients (ASGARD): A Feasibility Study

EudraCT no. 2015-001787-19

The investigational medical product is Anakinra (Kineret), an IL-1 receptor antagonist (IL-1 RA) that competes with IL-1beta for its receptor (IL-1R) and thereby preventing its action[16]. It is a recombinant, non-glycosylated form of naturally occurring human IL-1RA. It comes in 1mL pre-filled syringes containing 100mg of Anakinra in solution containing sodium citrate, sodium chloride and disodium EDTA and polysorbate in water for injection.

A pilot study of its use in gout showed prompt resolution of symptoms in patient's treated with anakinra[17], including in three patients with renal impairment. There have been further reports of the use of Anakinra in difficult to treat cases[18-22]. All these studies show good efficacy with the majority of patients having a good response within 24 hours. There was one case report of neutropenia in one patient with a kidney transplant with severe renal impairment.[23]

Anakinra has a lot of evidence in the use of rheumatoid arthritis as a biological response modifier. Large, multi-national placebo-controlled trials did not show a significantly increased risk of infection or major adverse events[24-26]. It was well tolerated in the long-term, even up to three years[27]. Anakinra has also been shown to be beneficial in studies looking at diabetes[28] and acute heart failure[29].

Anakinra has US FDA approval for use in rheumatoid arthritis. It is currently not recommended by NICE (February 2009, NICE) for rheumatoid arthritis except in a controlled clinical study. Anakinra is currently not licenced for use in the treatment of acute gout.

We want to explore the use of Anakinra for acute gout in patients with moderate renal impairment in comparison to the current standard therapy of steroids. We feel that IL-1RA inhibition provides a very specific treatment option with less side-effect. This is a timely study that aims to establish a firm evidence base for the management of a relatively common condition in a high risk group that is associated with considerable morbidity and costs.

- Intramuscular methylprednisolone acetate (Depo-Medrone)

Intramuscular methylprednisolone acetate is the commonest depot steroid formulation used in the UK. Intramuscular methylprednisolone 120mg provides the equivalent dose of steroid used in a study comparing steroids vs. NSAIDS (i.e. 35 mg for 5 days). It is a CTIMP in this study as, even though it is commonly used, it has not been used in a clinical study. The rate of side-effects even with short-term steroid treatment in acute gout is also unknown.

- Lipofundin MCT/LCT (intramuscular methylprednisolone placebo)

Lipofundin is a lipid emulsion for injection that is routinely used to provide nutritional support of calories and essential fatty acids. Depot steroid injection has to be prepared in a lipid emulsion, similar to Lipofundin. It has been used as an equivalent placebo to Triamcinolone in contemporary studies comparing Canakinumab, another IL-1 inhibitor, vs. Triamcinolone[30]. It has an important role in the design of the study in that it maintains a double-blind aspect of the design. This would not be possible is 0.9 Saline was used. It is being used as a CTIMP after discussion with the MHRA as it is not normally used for this purpose.

2 RATIONALE

This project was initially conceived because nephrologists still see patients who have chronic kidney disease or acute kidney injury in patients with gout due to treatment with NSAIDS. The other

For peer review only - http://bmjopen.bmj.com/site/about/guidelines.xhtml

Health Research Authority

Anakinra vs. Steroids for the Treatment of Gout Attacks in Renal Patients (ASGARD): A Feasibility Study

EudraCT no. 2015-001787-19

potentially harmful agent that is widely used without any evidence base in patients with chronic kidney disease is colchicine. These patients usually develop diarrhoea, which is an early sign of toxicity. The patient group felt strongly that protecting renal function from further injury should be a priority for any clinician. This reduces the increased mortality and risk of dialysis associated with deterioration in renal function. IL-1 signalling has been shown to play a key role in gout associated inflammation[31] and targeted therapy is now available with the increasing numbers if IL-1 inhibitors.

Two such inhibitors of IL-1 have been used in randomised controlled trials against the only other evidence-based treatment option of steroids. Although they showed efficacy, FDA approval was not granted due to safety concerns with these agents.[32] Rilonocept, an IL-1 decoy receptor, has been shown to be efficacious in reducing acute flares during the initiation of urate lowering therapy[33,34] but no added benefit was obtained for the treatment of acute gout when it was used in addition to an NSAID[35]. Canakinumab, a monoclonal antibody against IL-1 beta showed efficacy preventing acute flares during the initiation of urate lowering therapy, and for acute gout in patients with difficult disease or contra-indication to colchicine or NSAIDs[30,36,37,38]. The adverse event rate was considered too high by the FDA to approve its use for acute gout attacks.

Anakinra is a recombinant human interleukin-1 receptor antagonist with proven efficacy in acute gout. It is increasingly being used in patients with co-morbidities along with patients with other conditions such as diabetes and heart disease. Anakinra also offer advantages over other agents due to:

- Pharmacokinetics: Anakinra has been used in patients with severe renal disease with a modified regime, including patients on haemodialysis[39]. The prolonged action of Canakinumab with its irreversibility increases the risk of serious adverse events[40].
- Safety: Anakinra has excellent long-term safety profile from previous studies in Rheumatoid arthritis which consisted of regimes requiring daily injections for up to three years in patients with multiple co-morbidities[41].
- Cost: Anakinra 5 injections £131.15 vs. Canakinumab £9927.90.

The use of intramuscular methylprednisolone will also help establish some data for the use of corticosteroids for acute gout in patients with renal disease. If the initial results show equivalent efficacy between the two treatment arms, then we could go on to establish steroids as the standard treatments and making considerable savings.

In summary, there is poor evidence for the treatment of acute gout in patients with CKD. This is not an ideal situation as the number of patients with CKD is increasing, and a substantial proportion of patients with gout have CKD. Glucocorticoids, even with their poor evidence base, form the mainstay of treatment for acute gout in patients with kidney disease. With a safe, novel agent now available, we feel this is a timely study exploring a novel specific treatment option for an increasing group of patients where no routinely safe option is available. We hope to go on to undertake a definitive study that would enable guidelines to be updated for this specific patient group. A double-blind, double dummy study will add experimental rigour to the study and will help add to much needed overall evidence base.

2.1 Assessment and Management of risk

Anakinra has been used to try and treat people with Rheumatoid Arthritis, where a lot of patients were already on treatments that would suppress their immune system. The treatment was for many months (up to six months) and there was a slightly higher risk of infection (2%) and neutropenia in people who had also been on Anakinra.

For peer review only - http://bmjopen.bmj.com/site/about/guidelines.xhtml

Another agent called Canakinumab, that works similarly to Anakinra but is very long acting and lasts in the body for many weeks, has also been shown to have a higher risk of infection and neutropenia when this agent was used to treat gout. It was felt that this risk did not weigh up against the benefit of the treatment and so it is advised to use with caution and not readily recommended for the treatment of gout attacks.

Anakinra may be in the best position for treating gout attacks. The treatment time will be short, five days versus many months, and we feel the risk of infection should be much less. We know from the studies in people with Rheumatoid Arthritis, it was well tolerated by people. It also doesn't last in the body for many days so we do not anticipate the same complications as with Canakinumab. If a participant develops an infection or neutropenia, we can stop treatment and it should come out of the system within days, unlike Canakinumab which can last for weeks.

Anakinra has already been shown to be effective for treating gout, even when used in patients with multiple medical problems and active infections. Anakinra was first used in a clinical setting in people with severe septicaemia and those who received Anakinra were not worse off.

To minimise this potential risk, we are excluding patients with serious active infections requiring hospital admission and intra-venous (IV) anti-biotic drug administration. We are also excluding people with an active viral infection and people who may be receiving other treatment that can compromise their immune system. We are also excluding people with low white blood cell counts at the start of the study.

The initial use of Anakinra was in sepsis and it was not associated with a higher risk of mortality. Our protocol will exclude patients with uncontrolled disease including infections. We will check bloods at the end of the course of 5 days treatment to ensure that blood counts are stable. Patients will also be assessed at Day 2, Day 7 and 8 weeks, patients will be asked to stop treatment if serious infection develops. There will be daily contact by phone on weekdays for the duration of treatment.

As with any treatment for acute gout, there is always a risk that a flare will occur at the end of treatment. This has been described in a few patients from the literature. Concurrent treatment with prophylaxis is an area of on-going study in itself, especially in patients with renal disease who are prone to reactions to allopurinol. Guidance on when to start appropriate prophylaxis use is lacking. Some studies suggest prolonged courses of low dose colchicine which, risks accumulation and some suggest starting allopurinol. We will obtain data on flares after treatment from the 8-week follow-up and possible plan a subsequent study looking at long-term prophylaxis.

We have intentionally avoided using Anakinra in patients with severe renal failure (eGFR < 30mls/min/1.73m^2) due to concerns about accumulation in pharmacokinetic studies. We are taking caution even though it has been used safely in these patients and we have some experience in our centre. It has also been described in patients on haemodialysis using an alternate day regime where no adverse complications were noted.

All the above risks are slightly higher that the risks of standard medical care. This also has to be offset against the risk associated with standard care of steroids which can cause uncontrolled diabetes, worsening salt and water retention in heart failure and kidney disease and a possible infection risk as well. Furthermore, we are aware that most patients even in secondary care are given agents that pose harm to them. Evidence suggests that Anakinra may be beneficial in patients with diabetes, heart failure and possibly in patients with diabetic kidney disease as well.

2.2 Rationale for dose and dose regimen

For peer review only - http://bmjopen.bmj.com/site/about/guidelines.xhtml

The rationale for the use of 100mg s/c for 5 days come from emerging data where Anakinra has been used to treat acute gout. Some reports vary between 3 to 5 days but most use 5 days' duration of treatment to ensure adequate response in all patients[17-23]. This includes our own experience[23] where we have used 5 days' duration of treatment to have a standard approach to all patients, exclude patients with severe kidney disease (eGFR <30 – an alternate day injection regime is used).

The 100mg dose in part reflects experience from the use on Anakinra in studies to treat Rheumatoid Arthritis where most studies used a dose on 100mg but some studies used a range of 50mg to 100mg. Dosing in Cryopryin Associated Periodic Syndrome can be up to 3mg – 4mg per kg in severe disease (doses up to 800mg). We cannot use too high a dose due to the risk of accumulation in patients with chronic kidney disease. 100mg has been used in the patients with good efficacy and in patients with CKD without too many adverse events[17-23].

3 OBJECTIVES AND OUTCOME MEASURES/ENDPOINTS

3.1 Primary objective

The aim of this study is to determine the feasibility of running a phase III double-blind, double-dummy randomised controlled trial comparing intramuscular methylprednisolone (Depo-Medrone) 120mg vs. Anakinra 100mg subcutaneous injection for 5 days for the treatment of acute gout attacks in patients with chronic kidney disease as defined by a eGFR < 60mls/min/1.73m^2 and ≥ 30mls/min/1.73m^2.

The population to be studied are patients with moderate kidney disease and acute gout. There is increasing prevalence of these patients in the population due to an older population and the presence of con-current disease such as diabetes, atherosclerotic disease and heart disease.

The intervention is the treatment for the acute gout attack, which will be Anakinra. This is a target specific agent, in 100mg pre-filled injection for 5 days.

There is not standard comparison per se as evidence is lacking in this patient group for this condition but steroids have the best evidence and the commonest agent prescribed in the UK is intramuscular methylprednisolone acetate (Depo-Medrone).

The primary outcome of interest is feasibility to undertake a definitive randomised controlled trial and safety outcome assessment. Proposed outcome measure for subsequent definitive studies will look at effectiveness of treatment with regards to resolution of symptoms.

The time to assess outcome from a resolution of symptoms would be for 7 days with one final assessment at 8 weeks.

3.2 Secondary objectives

We will test the feasibility of using our primary outcomes measures of effectiveness from day 1 to day 7. These domains are suggested by the OMERACT guidelines, an international panel of experts and patients looking at outcome measures in gout. A significant component of the larger study will be a health economic evaluation to compare the incremental cost-effectiveness of Anakinra to the comparator treatment, steroids. Blood and urine tests for resolution monitoring of full blood count, kidney function and markers of inflammation. Complications such as infection, uncontrolled blood sugars and salt and water retention will be monitored.

3.3 Outcome measures/endpoints

For peer review only - http://bmjopen.bmj.com/site/about/guidelines.xhtml

Health Research Authority

Anakinra vs. Steroids for the Treatment of Gout Attacks in Renal Patients (ASGARD): A Feasibility Study

EudraCT no. 2015-001787-19

This is a feasibility study. It is not powered to look for inference. Study process will be analysed. Recruitment and retention rates, and willingness of patients to be randomised will be calculated. Proportion of patients who did not meet eligibility criteria will be looked at. Adherence and compliance rates, and qualitative feedback will be examined. Economic data on healthcare resource use and health-related quality of life will be collected to inform the design of a subsequent larger definitive study. Key drivers of costs, resource allocation and scalability will also be determined. A significant component of the larger study will be a health economic evaluation to compare the incremental cost-effectiveness of Anakinra to the comparator treatment. Safety outcome measures will be reported as mandated in a CTIMP protocol.

Criteria for success

- Recruit approximately one subject every two weeks - target of 32
- At least 70% of eligible patients can be recruited
- At least 85% of all recruited patients complete 5 out of 7 patient reported pain outcome (proposed primary outcome measure for subsequent study)
- No more than 10% treatment crossover, or failure as defined by the use of additional agents such colchicine or further doses of steroids.

3.4 Primary endpoint/outcome

Feasibility of undertaking a definitive multicentre, double-blind, double dummy randomised controlled trial to obtain clear guidance on the safe management of acute gout attacks in patients with chronic kidney disease.

3.5 Secondary endpoints/outcomes

Proposed primary outcome measures of effectiveness consist of resolution of pain i.e. time to 50% reduction and complete resolution of pain in self-assessed pain intensity in the joint most affected at baseline measure on the VAS (0-100mm) and 5-point Likert scale from baseline to 7 days post randomisation (Day 1, Day 2, Day 3, Day 4, Day 5, Day 6, Day 7) using composite time points kept to intervals 24 hour intervals as close as possible.

Proposed secondary outcome measures:
Patient reported outcome measure (Day 1, Day 2, Day 3, Day 4, Day 5, Day 6 and Day 7)
Physician assessment of joint tenderness and swelling (Day 1, Day 2, and Day 7)
Assessment of activity limitation and quality of life: HAQ-DI and SF-36 at Day 1, Day 7 and 8 weeks; EQ-5D-5L at Day 1, Day 2, Day 7 and 8 weeks;
LEFS at Day 1, Day 2, Day 7.
Time to take rescue medication
Healthcare resource use.
Blood and urine tests renal on Day 1, Day 2, Day 7 and 8 weeks, consisting of FBC, U+Es, eGFR, glucose, CRP, serum uric acid and spot urine uric acid.
Qualitative assessment of participant’s experience at end of participation i.e. 8 weeks.

3.6 Exploratory endpoints/outcomes

For peer review only - http://bmjopen.bmj.com/site/about/guidelines.xhtml

NHS
Health Research Authority

Anakinra vs. Steroids for the Treatment of Gout Attacks in Renal Patients (ASGARD): A Feasibility Study

EudraCT no. 2015-001787-19

Measurement of inflammatory markers in blood and urine including. Serum and urine samples obtained will be stored for laboratory analysis at a later date. Changes in epigenetic DNA with both treatments.

Objectives	Outcome Measures	Time point(s) of evaluation of this outcome measure (if applicable)
**Primary Objectives**		
Feasibility of undertaking a definitive multicentre randomised controlled double blind, double dummy study<br><br>Safety and adverse event		End of study.<br><br>8 week post randomisation.
**Secondary Objectives**		
Proposed primary outcome measures for subsequent study looking at effectiveness of treatment with regards resolution of pain.	Time to 50% reduction and complete resolution of pain in self-assessed pain intensity in the joint most affected at baseline measure on the VAS (0-100mm) and 5-point Likert scale on patient diary.	At baseline (D1) to 7 days post randomisation (D1, D2, D3, D4, D5, D6, D7).
Proposed secondary outcome measures for subsequent study looking at effectiveness of treatment with regards to patients reported outcome measure and physician clinical assessment.<br><br>Patient assessment of activity limitation and quality of life.	Patient reported outcome measure on 5-point Likert scale on patient diary.<br><br>Physician assessment of joint tenderness and swelling on 5-point Likert scale.<br><br>• LEFS<br><br>• EQ-5D-5L	At baseline (D1) to 7 days post randomisation (D1, D2, D3, D4, D5, D6, D7).<br><br>At baseline (D1) and D2, D7.<br><br>At baseline (D1), D2, D7 and 8 weeks.

For peer review only - http://bmjopen.bmj.com/site/about/guidelines.xhtml

Health Research Authority

	• HAQ-DI and SF-36	At baseline (D1), D7, and 8 weeks.
Use of rescue medication	Time to take pre-specified rescue medication or treatment failure.	At baseline (D1) to 7 days post randomisation (D1, D2, D3, D4, D5, D6, D7).
Health economic evaluation to compare the two treatment arms.	Healthcare Resource use data	8 weeks
Qualitative assessment of study involvement (12 participants)	Interview	8 weeks
Monitoring of blood counts, kidney function, glucose, uric acid and markers of inflammation.	Blood and urine test for Change in FBC, renal function by eGFR, CRP, glucose, uric acid and urine uric acid.	D1, D2, D7 and 8 weeks.
**Tertiary/Exploratory Objectives**		
Changes in inflammatory blood and urine markers, including epigenetic changes in circulating blood cells.	Inflammatory cytokines e.g. IL-1alpha, IL-6, TNF-alpha, TGF-beta) from stored serum, plasma and urine analysed at end of clinical trial. Changes in epigenetic DNA with treatment.	D1, D2, D7 and 8 weeks.

For peer review only - http://bmjopen.bmj.com/site/about/guidelines.xhtml

Health Research Authority

Anakinra vs. Steroids for the Treatment of Gout Attacks in Renal Patients (ASGARD): A Feasibility Study

EudraCT no. 2015-001787-19

4 TRIAL DESIGN

This study is a multi-centre, double-blind, double dummy randomised controlled trial in which patients will be randomised into two arms of treatment, Group A (Experimental arm) will be given Anakinra and placebo intramuscular methylprednisolone (Lipofundin), Group B (Active comparator) will be given intramuscular methylprednisolone (Depo-Medrone) and placebo Anakinra. It is a feasibility study and not powered for statistical inference. It is designed to gather safety information and feasibility of conducting a full-scale trial.

5 STUDY SETTING

This is a multi-centre randomised controlled trial based in centres in the East of England region. Each site will have a nominated principle investigator. Recruitment will be from patients who present with acute gout and chronic kidney disease in secondary care, or are already in secondary care.

Patients will be in pain from acute gout so recruitment will be in the acute setting, either from Accident and Emergency or the Acute/ Surgical Medical Unit. Patients already in hospital who develop acute gout attacks can also be approached for recruitment. Known patients with moderate kidney disease and difficult to treat acute gout will also be primed to contact the research team. We will use primary care sites as participant identification centres to help recruit potential participants before an acute attack by sending letters of invitation.

The majority of patients with acute gout flare can be easily treated. Recruiting from primary care was criticised in two studies[11,12]. We want to recruit patients who present to secondary care either because of co-morbidities, disease severity or functional disability affecting patient function.

We would suggest that primary care practitioners refer patients to secondary care who have co-morbidities, history of refractory disease or severely affecting function. A good interface between the rheumatologists (PI) and the acute services would be ideal.

The intervention will be co-ordinated by the trial team, once patients consent to randomisation, patients will receive treatment that would have been co-ordinated by the trial team. The pharmacy team will prepare the medication for administration and keep a record of medication allocation. This will be blinded to the trial team, treatment will be administered by the healthcare staff looking after the patient. The patient will be blinded throughout the study and even after the end of study.

All hospitals should be able to have pharmacy support to provide preparation and recording of treatment administered.

6 ELIGIBILITY CRITERIA

6.1 Inclusion criteria

For peer review only - http://bmjopen.bmj.com/site/about/guidelines.xhtml

Health Research Authority

Patients who meet inclusion criteria will be approached by a study investigator. Consent for participation will be sought. Upon gaining consent, the investigator will go through exclusion criteria.

1. Subjects capable of giving informed consent.
2. Male or non-pregnant, non-nursing female
3. ≥ 18 years of age
4. eGFR < 60mls/min/1.73m^2 and ≥ 30mls/min/1.73m^2 as calculated using serum creatinine and modified MDRD formula or CKD-EPI, on two occasions at least two months apart with one being in the last six months. Creatinine at time of presentation can be used unless participant has an acute kidney injury as defined by serum creatinine rise by ≥ 26µmol/L within 48 hours or ≥ 1.5 fold rise from baseline value.
5. Diagnosis of gout arthritis as defined by the American College of Rheumatology 1977 preliminary criteria.
6. Gout attack less ≤ 36 hours
7. Baseline pain intensity greater than or equal to 50mm on the 0-100 mm VAS. In the case of multiple joints (≤ 3), the most affected joint will be assessed.

6.2 Exclusion criteria

1. Treatment with Colchicine in the last week.
2. Initiation of or change to dose of NSAIDS in the last week. NSAID use is not an exclusion criterial if taken at a stable dose for at least four weeks.
3. Initiation of or change to dose of systemic steroid in the last week. Systemic steroid use is not an exclusion criteria if taken at an equivalent dose of 10mg Prednisolone or less for the last four weeks.
4. Polyarticular gout, i.e. affecting four or more 4 joints
5. Rheumatoid arthritis, evidence/suspicion of infectious/septic arthritis, or other acute inflammatory arthritis such as systemic lupus erythematosus, mixed connective tissue disease, scleroderma, polymyositis, or significant systemic involvement secondary to rheumatoid arthritis.
6. Con-current immunosuppression/immunomodulatory treatment (Calcineurin inhibitor, anti-proliferative or biologic) therapy for other reason i.e. organ transplant.
7. Prior history or current inflammatory joint disease other than gout (e.g. rheumatoid arthritis (RA), reactive arthritis, psoriatic arthritis, seronegative spondyloarthropathy, Lyme disease).
8. Current active malignancy (with the exception of basal cell or squamous cell carcinoma of the skin, cervical intraepithelial neoplasia and non-metastatic/advanced prostate cancer).
9. Any patients with contra-indication to intramuscular injection such as coagulopathy or thrombocytopenia (Platelet count<100 x 10^9/L (100,000/mm^3)).
10. Abnormal liver function tests: Total bilirubin>upper limit of normal, Alanine aminotransferase (ALT) or Aspartate Aminotransferase (AST) >2 times upper limit of normal.

For peer review only - http://bmjopen.bmj.com/site/about/guidelines.xhtml

Health Research Authority

Anakinra vs. Steroids for the Treatment of Gout Attacks in Renal Patients (ASGARD): A Feasibility Study

EudraCT no. 2015-001787-19

11. Haemoglobin < 85g/L (8.5 g/dL)

12. White blood cell (WBC) count<1.5 x 10^9/L (1000/mm^3), absolute neutrophil count<1.5 x 10^9/L (1000/mm^3)

13. Evidence of active infection requiring intra-venous (IV) anti-biotic drug administration within last four weeks. Potential participants who were empirically treated with a course of intravenous antibiotics for a presumptive diagnosis of an infection may be eligible where the treating medical care team has subsequently excluded an active infection and consequently discontinued antibiotic therapy at least four hours before randomisation. These potential participants can be discussed with the investigator where clarity is required.

14. Evidence of uncontrolled concomitant cardiovascular, nervous system, pulmonary (including obstructive pulmonary disease), hepatic, endocrine (including uncontrolled diabetes) or gastrointestinal disease. Potential participants who have active concomitant disease can only be eligible after discussion and agreement with the treating medical team.

15. Known positive hepatitis B virus surface antigen (HBsAg), hepatitis C (HCV) antibody or HIV.

16. Females of child bearing potential who are not willing to use highly effective birth control methods from the time of consent to one week after treatment discontinuation. Highly effective method of contraception (hormonal or barrier method of birth control; abstinence) consist of:

    - Combined (estrogen and progestogen containing) hormonal contraception associated with inhibition of ovulation; oral, intravaginal, transdermal.
    - Progestogen-only hormonal contraception associated with inhibition of ovulation; oral, injectable, implantable.
    - Intrauterine device (IUD) or intrauterine hormone-releasing system (IUS)
    - Bilateral tubal occlusion or vasectomised partner
    - Sexual abstinence, defined as true abstinence when it is in line with the preferred and usual lifestyle of the participant. Periodic abstinence (e.g. calendar, ovulation, sympthothermal, post-ovulation) and withdrawal methods are not acceptable methods of contraception.

17. Men who are sexually active with a female partner who are not willing to use highly effective birth control methods from the time of consent to one week after treatment discontinuation.

18. Females of childbearing potential must have a negative pregnancy test (highly sensitive urine or serum pregnancy test after a confirmed menstrual period) within 7 days prior to treatment initiation. Subjects are considered not of child bearing potential if they are surgically sterile (i.e. they have undergone a hysterectomy, bilateral tubal ligation, or bilateral oophorectomy) or they are postmenopausal.

19. Females must not be breastfeeding.

20. Patients who have had treatment as part of this trial cannot have repeat treatment for another flare as part of the trial.

For peer review only

For peer review only - http://bmjopen.bmj.com/site/about/guidelines.xhtml

21. Patients with allergies to excipients of IMPs: citric acid, anhydrous, sodium chloride, disodium edetate dehydrate, polysorbate 80, sodium hydroxide. Hypersensitivity to E. Coli derived proteins, egg proteins and soy proteins. Patients with a latex allergy are also not eligible as the inner needle cover of the pre-filled syringe contains dry natural rubber (a derivative of latex).

For peer review only

7 TRIAL PROCEDURES

7.1 Recruitment

Patient who present to secondary care with acute swollen joints with a diagnosis of acute gout will be approached for recruitment. Patients already in hospital as in-patients will also be eligible for screening. Primary care practitioners will be encouraged to send difficult to manage cases such as patients with gout that effects their function and complex treatment (i.e. previous history of difficult to treat gout), as per our local CCG guidelines. This may either be to acute secondary care services or rheumatology out-patients depending on the local practice of the centre. Known patients with moderate CKD and a history of acute gout can be primed to contact the research team either via the general practitioner or the acute services, or directly. We will use primary care sites as participant identification centres to help recruit potential participants before an acute attack by sending letters of invitation. This will help minimise any delays to treatment as part of the study during an acute attack.

Initial diagnosis may be made by any clinician, the trial team based at that centre screen patients using standardised criteria for diagnosis as per eligibility criteria (used by historical and current trials of acute gout). A pre-specified list of analgesic that would least likely impact on the outcome can be used for pragmatic reasons prior to screening to avoid delaying relieving patient discomfort. Patients who present out of hours may not be approached for recruitment as to not avoid delays in treatment.

Information will be sought on patients who are screened and for participants who are not randomised as per Consolidated Standard of Reporting Trials (CONSORT) guidance.

7.1.1 Patient identification

The patient will be initially identified by a nurse practitioner or clinician in the participating acute trusts. Patients with hot joints can present to the emergency room, acute medical take, surgical take (orthopaedics), directly to rheumatologists or in-patients who develop acute gout, which is a common presentation. Patients will only be identified on clinical presentation to medical services i.e. the clinical team in charge of the patient's care. We will use primary care sites as participant identification centres to help identify potential participants before an acute attack by sending letters of invitation.

7.1.2 Pre-Screening

Trial posters will be displayed in primary care and community care settings, directing potential participants to their nearest research site and contact person. The same posters may be used to advertise the study via hospital clinic waiting areas, in-patient wards, internet and social media.

For peer review only - http://bmjopen.bmj.com/site/about/guidelines.xhtml

Potential participants will undergo an initial pre-screening process for basic eligibility; inclusion and exclusion. This will include a review of renal function and information on previous testing for gout diagnosis such as uric acid, and findings of imaging and joint fluid analysis.

This will help avoid ineligible participants undergoing a baseline assessment and consent process who can be discounted as being ineligible early on. These patients would still receive treatment by the research team for their joint condition / presentation. Potentially eligible patients will also be assessed sooner and if randomised, receive allocated treatment sooner for their painful presentation.

7.1.3 Screening

The clinical presentation of an acute hot swollen joint due to a diagnosis of gout should prompt referral by the nurse practitioner or clinician for screening if the patient is willing to be approached. Tests may be undertaken by the clinical team responsible for the patients care to confirm or refute the diagnosis as part of on-going standard care for the patient. All patients must freely give their informed consent before any trial-related procedures may be conducted. These will consist of:

- Gaining informed consent from the patient
- Recording demographic details: gender, date-of-birth, ethnic origin, previous medical history (diabetes, cardiovascular disease, respiratory disease, smoking details, and alcohol intake).
- Concomitant medications
- History of Hepatitis B, C, HIV
- History of malignancy
- Additional blood tests may consist of clotting (could be added onto initial request if possible), liver function tests, full blood count and renal function

7.2 Consent

The Principal Investigator (PI) retains overall responsibility for the informed consent of participants at their site and must ensure that any person delegated responsibility to participate in the informed consent process is duly authorised, trained and competent to participate according to the ethically approved protocol, principles of Good Clinical Practice (GCP) and Declaration of Helsinki. If delegation of consent is acceptable then details of above should be provided for the delegated individual. A clinician can only obtain consent.

Informed consent will be obtained prior to the participant undergoing procedures that are specifically for the purposes of the trial and are out-with standard routine care at the participating site (including the collection of identifiable participant data). The right of a participant to refuse participation without giving reasons will be respected. The participant will remain free to withdraw at any time from the trial without giving reasons and without prejudicing his/her further treatment and must be provided with a contact point where he/she may obtain further information about the trial.

For peer review only - http://bmjopen.bmj.com/site/about/guidelines.xhtml

If a participant is required to re-consent or if new information is required to be provided to a participant it will be the responsibility of the PI to ensure this is done in a timely manner.

Potential participants should be able to give consent and a person is assumed to have mental capacity to make a decision unless it is shown to be absent.

Assessment of capacity for participants to give consent themselves will consist of a person who:

- Understand the purpose and nature of the research
- Understand what the research involves, its benefits (or lack of benefits), risks and burdens
- Understand the alternatives to taking part
- Be able to retain the information long enough to make an effective decision.
- Be able to make a free choice
- Be capable of making this particular decision at the time it needs to be made (though their capacity may fluctuate, and they may be capable of making some decisions but not others depending on their complexity)
- Where participants are capable of consenting for themselves but are particularly susceptible to coercion, it is important to explain how their interests will be protected

Patients who are unable to consent for themselves for suitability for the trial will not be approached as obtaining patient reported outcome measures will not be practical. If verbal translation is needed, this should be via a hospital interpreter or a personal interpreter. Telephone interpretation services are not acceptable and written material will not be provided in various languages for the purpose of this feasibility study. This study utilises questionnaire surveys that have not been validated in different languages. There is also a qualitative element to the study where lack of required language skill may prove to be difficult. Time is limited for potential participants to be considering participation and any delays in seeking consent and translation services that may prolong treatment of the patient in acute pain have also to be considered.

Written material consisting of participant information leaflet and consent documentation will be approved by the Research Ethics Committee and will be in compliance with GCP, local regulatory and legal requirement. This is a feasibility study and the costs to undertake centrally commissioned translated documents may be too high. This may be something to consider for the subsequent definitive study.

There will be opportunity for the participant to ask questions to the PI or a member of the research team. The patient will be given 24 hours to consider the information and consider their participation. However, treatment cannot be withheld during this period and exclusion criteria will be applied if duration of attack is longer than accepted or if contra-indicatory medication is administered.

A copy of the ICF and PIS will be given to the participant, with the original ICF filed in the medical notes and a copy of the ICF in the ISF.

Consent will be specifically obtained for:

- Permission to have access to relevant sections of medical records by study team members, including the members from the coordinating center (ARCTU), the primary centre (Southend Hospital), and regulatory authorities

- Identifiable routine blood tests which will be stored within the NHS clinical system and will be available for doctors in the future if required for future care within the NHS system

For peer review only - http://bmjopen.bmj.com/site/about/guidelines.xhtml

- Contact information to be made available to the research team in the site file for ongoing assessment and monitoring as part of the study. Information will also be used to update participant of any new findings that may become relevant during the course of the study. This may include safety information resulting in significant changes to the study risk: benefit assessment, the protocol, PIS and/or the ICF, will be reviewed and updated as necessary. Participants will be given a copy of the revised consent form. They will then be asked to re-consent if they choose to continue the study.
- Permission for audio interviews to be recorded for the qualitative component for the study.
- Permission to retain contact details for long-term follow-up with regards to use of subsequent urate-lowering therapy and complications, and to pass on information regarding details of future research.

If a participant who has consented but later becomes incapacitated, the original consent endures the loss of capacity but patient may be withdrawn if patient reported outcome data cannot be obtained.

7.2.1 Additional consent provisions for collection and use of participant data and biological specimens in ancillary studies, if applicable

There will be a tertiary / exploratory study where patient's serum and urine will be stored for future analysis at the end of the study. Testing will be an extension of routine laboratory testing to look for markers of inflammation in an exploratory setting.

Consent will be obtained specifically for the use of DNA, although it will be made clear that the research is concerned with inflammatory changes in epigenetic DNA and subsequent changes with treatment in gout. Patients will have the option of not contributing to this study as part of the consent procedure, and also withdrawal at a subsequent date.

Permission will be obtained specifically for storage of samples up to 15 years and to be used for future, as yet unspecified, medical research into health, illness and medical treatment. This research will be subject to proper scientific and ethical review.

Some samples will be exported to countries in the European Union for further analysis. Participants will be consented specifically for this and exported material will be handled, stored, transported and disposed in accordance with the consent given. The collaborators / recipient country will ensure that the material has been handled properly and that the required standards of that collaborating institution / country have been met.

7.3 The randomisation scheme

Enrolled patients will undergo 1:1 block randomisation by ARCTU, to either Anakinra (Experimental arm) or intramuscular methylprednisolone (active comparator arm). Randomisation will be undertaken using an Internet-based randomisation system within ARCTU. The ARCTU uses the TENALEA system provided by the Trans European Network for Clinical Trial Service. The system stores the pre-determined sequence of randomisation.

This list is not available to the investigator or ARCTU staff. Once a patient has consented to take part in the trial, the designated staff will login to a web page after entering information required to check eligibility

For peer review only - http://bmjopen.bmj.com/site/about/guidelines.xhtml

and they will be randomly allocated to either arm. Allocation will be sent to pharmacy who will then dispense allocated treatment. All investigators involved with the trial will be blinded to treatment. Patients will not be informed of their assigned treatment during the study. Pharmacy will keep a record of allocated treatment arms in the event emergency un-blinding is required. This will be reported to the central pharmacy to keep up stocks of relevant medication as well.

7.3.1 Method of implementing the allocation sequence

A web based randomisation/treatment allocation system will be used. Once allocated to a treatment arm, the pharmacy department will co-ordinate the treatments. A treatment and appropriate placebo will be generated by pharmacy, this will be concealed to the trial team and healthcare team involved in administering treatment. The pharmacy will keep a record of all treatments given.

Anakinra and its placebo will be ready made by the manufacturer. Intramuscular methylprednisolone and its placebo (Lipofundin) will be prepared by a research team member or healthcare team member not involved with the study and be administered by healthcare team caring for the patient, and not involved with the trial. Patients who present out-of-hours where implementation of randomisation may delay treatment should not be approached for study. Randomisation codes will be available from pharmacy in the event of an emergency or if out-of-hours access is required.

Participants will be randomised to one of two treatment arms, Group A and Group B:

- Group A: Anakinra 100mg s/c Day 1 to Day 5 and placebo intramuscular methylprednisolone (Lipofundin) 120mg i/m Day 1
- Group B: Intramuscular methylprednisolone (Depo-Medrone) 120mg i/m Day 1 and placebo Anakinra 100mg s/c Da 1 to Day 5

7.4 Blinding

All trial investigators will be blinded to the intervention. Pharmacy will keep a record of treatments administrated. All trial participants, care providers and outcome assessors will be blinded to the treatment. Each treatment has its equivalent placebo to ensure blinding in maintained throughout study.

7.5 Un-blinding

The study code should only be broken for valid medical or safety reasons e.g. in the case of a severe adverse event where it is necessary for the investigator or treating health care professional to know which treatment the patient is receiving before the participant can be treated.

The treatment code for the patient can be broken in the reporting of a SUSAR. However, the blind should be maintained, where possible and appropriate, for staff that are involved in data analysis and interpretation. It is the allocated responsibility of the CI by the sponsor for pharmacovigilance management and reporting. In this instance, an allocated un-blinded individual (s), with no involvement in data management of the study should be responsible for the un-blinding event. The un-blinding of single cases by the PI/CI in the course of a clinical trial should only be performed if necessary for the safety of the trial participant.

We do not expect CTIMPs to be associated with high morbidity and mortality, and feel that most events will be related to the underlying risk inherent in the patient group. This study is not powered as a comparison of efficacy / effectiveness and systemic un-blinding could be considered in the event of SUSARs / SAEs if the PI and sponsor feel that it may be related to trial treatment and is clearly

For peer review only - http://bmjopen.bmj.com/site/about/guidelines.xhtml

necessary for the appropriate medical management of the participant. In all cases the Investigator would be expected to evaluate the causality and expectedness of SAEs as though the participant was receiving the active medication.

The case should be assessed for seriousness, expectedness and causal relationship as if it was the tested IMP that caused the reaction. If the case appears to be a SUSAR then it should be un-blinded and the following considered:

- If the administered product is the tested IMP, the case would be reported as a SUSAR to the MHRA/ appropriate Main Research Ethics Committee/IMP provider (if applicable) within the timelines outlined in safety reporting section.
- If the administered product is a comparator with a marketing authorisation, the adverse reaction should be reassessed for expectedness according to the study protocol. If the adverse reaction is unexpected then the SUSAR should be reported; otherwise it is an expected serious adverse reaction which still requires reporting to the sponsor/IMP provider (if applicable) within 24 hours.

Emergency un-blinding/code breaks can be undertaken by the PI or medical team responsible for the care for the patients from the pharmacy department which will have a record of all treatment allocations for the patient at the respective centre. This is available out-of-hours. The investigator is not required to discuss un-blinding if he or she feels that emergent un-blinding is necessary.

The pharmacy representative who is responsible for code breaks at ______________________________

is ______________________________________.

Failing this, the hub pharmacy centre will keep a record of participant trial number and allocation.

If the person requiring the un-blinding is a member of the Investigating team then a request to the holder of the code break envelope/list, or their delegate will be made and the un-blinded information obtained. If the person requiring the un-blinding is not the CI/PI then that health care professional will notify the Investigating team that an un-blinding is required for a trial subject and an assessment to un-blind should be made in consultation with the clinical and research teams. On receipt of the treatment allocation details the CI/PI or treating health care professional will continue to deal with the participant's medical emergency as appropriate.

Details of patients who have pre-mature un-blinding, including outcomes, will still be used for analysis. The CI/PI documents the breaking of the code and the reasons for doing so on the CRF/data collection tool, in the site file and medical notes. It will also be documented at the end of the study in any final study report and/or statistical report. The CI/Investigating team will notify the Sponsor in writing as soon as possible following the code break detailing the necessity of the code break. The CI/PI will also notify the relevant authorities. The written information will be disseminated to the TSC.

7.6 Baseline data / Pre-treatment Evaluations (Day 1)

This assessment should not occur within four hours of having taken an analgesic such as codeine, tramadol or paracetamol. The PI or trial staff (research nurse, research fellow) will undertake an assessment of the joint using a pre-specified scoring system.

- Vital signs (height, weight, general clinical assessment)

For peer review only - http://bmjopen.bmj.com/site/about/guidelines.xhtml

- Medical history specifically history of ischaemia heart disease, heart failure, type 2 or type 1 diabetes, peripheral vascular disease, cerebrovascular disease.
- Information in relation to new ACR/EULAR 2015 criteria

  Previous episode(s)
  - Pattern of previous joint(s) / bursa(e) involvement during previous symptomatic episodes
    - Not ankle, midfoot, or first MTP joint (or involved as part of polyarticular presentation)
    - Ankle *or* midfoot (as part of monoarticular or oligoarticular episode **without** involvement of first MTP)
    - Involvement of first MTP (as part of monoarticular or oligoarticular episode)
  - Characteristic of previous symptomatic episode(s)
    - Erythema overlying affected joint (patient- reported or physician – observed)
    - Can't bear to touch or pressure to affected joint
    - Great difficulty in walking or inability to use affected joint
  - Time course of previous episode(s);

    Number of previous episodes with 2 ≥ of (irrespective of anti-inflammatory treatment):
    - Time to maximal pain < 24 hours
    - Resolution of symptoms ≤ 14 days
    - Complete resolution (to baseline level) between symptomatic episodes
  - Previous serum urate level > 4 weeks from start of episodes or inter-critical period

    (Record highest value)
  - Previous synovial fluid analysis of symptomatic joint
    - MSU negative or positive
  - Previous imaging evidence of urate deposition in symptomatic joint or bursa: ultrasound evidence of double-contour sign *or* dual energy CT demonstrating urate deposition

  Current episode
  - Was joint aspiration undertaken, was MSU negative or positive
  - Pattern of joint(s) / bursa(e) involvement
  - Was imaging undertaken?
    - Imaging evidence of urate deposition in symptomatic joint or bursa: ultrasound evidence of double-contour sign *or* DECT demonstrating urate deposition
    - Imaging evidence of gout-related joint damage: conventional radiography of the hands and/or feet demonstrates at least 1 erosion
- Baseline renal function defined as CKD3; calculated using serum creatinine and modified MDRD formula or CKD-EPI, on two occasions at least two months apart with (one being in the last six months), if previous results not available use creatinine at time of hospital admission. Creatinine at time of presentation can be used unless participant has an acute kidney injury as defined by serum creatinine rise by ≥ 26μmol/L within 48 hours or ≥ 1.5 fold rise from baseline value.

For peer review only - http://bmjopen.bmj.com/site/about/guidelines.xhtml

- Blood and urine samples for routine testing within the NHS clinical system.
- Pain score using Likert and VAS and baseline outcome score on patient issued diary
- Clinical assessment of joint using standardised scoring assessment
- Quality of life questionnaires: SF-36, HAD-DI, EuroQol-5D, LEFS.
- Additional blood tests (one vial/one teaspoonful) and urine (one universal) for further study.

7.7 Trial assessments

This assessment should not occur within four hours of having taken an analgesic such as codeine, tramadol or paracetamol. The PI or trial staff (research nurse, research fellow) will undertake an assessment of the joint using a pre-specified scoring system.

First dose will be given on Day 1.

7.7.1 Evaluations after treatment

- Treatment Day 2, 3, 4, 5, 6, 7
- Pain score on diary card (VAS and Likert) - Day 2, 3, 4, 5, 6, 7
- Patient reported outcome measure of response to treatment (Likert) - Day 2, 3, 4, 5, 6, 7 Participants can be contacted on Friday and Monday as a reminder for weekend or compliance check respectively.
- Hospital visit for physician / study member assessment of joint tenderness and swelling (Day 1, Day 2 and Day 7)
- Assessment of activity limitation and quality of life using:
  - LEFS at Day 1, Day 2, Day 7.
  - EQ-5D-5L at Day 1, Day 2, Day 7 and 8 weeks.
  - HAQ-DI and SF-36 at Day 1, Day 7 and 8 weeks
- Blood and urine tests on Day 1, Day 2 Day 7, and Week 8 consisting of FBC, U+Es, eGFR, glucose, CRP, serum uric acid and spot urine uric acid. Additional bloods tests for exploratory tests.
- Last assessment at 8 weeks: resource use data and interview (12 patients) for qualitative assessment of participant's experience.

- If more than one joint is effected, then the worst effected joint is assessed.
- Participants will have their diary card entry checked; diary card entry will also note time of injection administration. On non-hospital attendance days, trial team will contact participants to administration of injection and entry into diary card. Participants should make self-assessments at time of diary card entry late morning or afternoon.
- Compliance with injections will be checked by asking participants to return used syringes back to trial team and this will be recorded. An inspection of the injection site will also be made. Arrangements can be made to collect non-puncture containers if patients who are not able to deliver it themselves.
- If a participant is not able to self-inject, then a carer could administer the injection or district nurses will be organised to administer injection.
- Patients will then attend hospital at 8 weeks to undertake last questionnaires. Diary cards will be checked on each hospital visit and be returned by the patient on Day 7.

For peer review only - http://bmjopen.bmj.com/site/about/guidelines.xhtml

Health Research Authority

- Patients who cannot attend hospital due to physical/medical reasons can be contacted by phone for administration of dose and diary card entry. Final assessment questionnaires at 8 weeks can be sent by the patient by mail, if patient is not able to attend for hospital visits.
- Patients will be asked at 8 weeks if any events that may have occurred for pharmacovigilance and also to undertake a telephone questionnaire of resource use. Patients may (12 out of 32) be asked to have a qualitative assessment of their experience of being involved with the trial.
- Blood and urine tests will be undertaken at Day 1 (pre-dose), Day 2, Day 7 and week 8, during hospital visits. Sample for the routine clinical assessments, including FBC, U+Es, CRP, urine uric acid / serum uric acid will be processed by the local centre. Samples for the exploratory study (serum and urine) will be processed and stored at the local centre. Samples will be stored using an anonymised unique identifier, once batched up to the end of study, samples will be sent to Southend University Hospital for storage and subsequent analysis of inflammatory markers.

7.8 Long term follow-up assessments

The final follow-up for the participant will be at 8 weeks. 8-week follow-up will consist for looking for any events possible related to treatment toxicity as part of CTIMP reporting. Patients will be asked to return completed questionnaires. Resource use data will be used to assess healthcare use for economic analysis.

Patients who give consent for further follow-up will be contacted for further research and their details stored for this purpose.

This is a short study and non-adherence and “loss to follow-up” will hopefully be minimal. Measure to improve adherence including using telephone calls to prompt treatment administration and pain assessments. The long-term 8-week follow-up can be conducted by telephone if required with participants sending in questionnaires by post.

7.9 Qualitative assessments – Nested studies

A qualitative assessment will be undertaken at the end of the study which will be undertaken as an interview with regards to obtaining participation experience. This was suggested by the funding body. Twelve out of the thirty-two patients will be randomly selected for qualitative assessment of participation experience.

Consent to obtain unlinked anonymised data with contact information for the qualitative aspect of the study at the 8-week time point will be explicitly obtained. This will be stored in the site file. Consent will be explicitly obtained for audio interviews to be recorded. Pseudonymised data will be used for recording purposes and data obtained will be stored in a locked cabinet with secure access only to Dr Jon Scales, University of Essex.

7.10 Withdrawal criteria

The investigator will make reasonable effort to keep each patient on the study for completeness sake. If the investigator removes a patient from the study, or if a patient declines further participation, prior to interventions

7.10.1 Patients lost

- **Withdrawn**

For peer review only - http://bmjopen.bmj.com/site/about/guidelines.xhtml

Patients who withdraw their consent at any point in the study fall into one of two categories. Site staff should ascertain which category a patient wishes to be in at the point of withdrawal;

- those who allow their data (collected up to the point of withdrawal) to be used

OR

- those who do not allow the use of any of their data collected prior to withdrawal

- **Lost to follow-up**

Patients who do not attend follow-up assessment after site staff have attempted to contact the patient at least twice, e.g. by telephone.

As these patients have not withdrawn their consent, the data already collected for them may be used and therefore needs returning in the usual manner.

These patients have not withdrawn their consent and therefore the data collected for them may be used.

7.10.2 Patients not lost

Patients that fall into one or more of the following categories are not considered as withdrawn or lost from the study. They should continue to attend all follow-up study assessments as per protocol, unless they subsequently fall into one of the categories listed in Section 7.1;

- Patients who ask to stop study treatment
- Pregnancy
- Serious infection i.e. necessitating intravenous anti-biotic treatment and hospital admission.
- Any event which in the judgement of the PI makes further study treatment inadvisable
- SAE requiring discontinuation of treatment

It is always within the remit of the physician responsible for a patient to withdraw a patient from a trial for appropriate medical reasons, be they individual adverse events or new information gained about a treatment. If any further adverse data regarding the IMP come to light patients will be withdrawn from the study.

7.10.3 Withdrawal documentation

All withdrawals will be monitored by the sponsor and a record will be kept. We will have a formal withdrawal document in place.

7.11 Storage and analysis of samples

Blood and urine samples will be obtained at baseline (day 1), and at hospital visits at day 2, day 7 and at 8 weeks. Patients should not require much further testing as these blood tests are routinely undertaken to assess disease response anyway.

Serum sample will be obtained using 6mL red top blood sample containers / vacutainers (a tablespoonful). The sample will be spun down at 1500g for 15 minutes. Using a plastic pipette, three X 2mLs aliquots will be stored in cryotubes. Serum will be stored at -80°C (+/- 10) freezer in the local centre.

For peer review only - http://bmjopen.bmj.com/site/about/guidelines.xhtml

Health Research Authority

Anakinra vs. Steroids for the Treatment of Gout Attacks in Renal Patients (ASGARD): A Feasibility Study

EudraCT no. 2015-001787-19

Plasma sample will be obtained by using a 6ml EDTA (pink top) and spun down at 1500g for 15minutes. Using a plastic pipette, three X 2mLs aliquots will be stored in cryotubes. Serum will be stored at -80ºC (+/- 10) freezer in the local centre. The cell pellet will be stored at -80ºC for further use.

Urine samples (early morning if possible) will be obtained. Patients will be given collection pots and be asked to bring sample with them at assessment visits. If -80ºC storage is not possible at the local centre, samples can be stored at -20ºC for a month and arrangements will be made for transfer to the primary centre for storage.

All research blood and urine samples (link-anonymised) will be processed at each site (each site will have a Sample Analysis and Chain of Custody Plans in the Study Operations Manual / Standard Operating Protocol). Samples will be transported to Southend Hospital at the end of study under the custodianship of the Chief Investigator.

Analysis will be done at the end of the study. Serum, plasma and urine samples will then be analysed at using validated scientific methods to quantify inflammatory markers. Inflammatory changes in the cell epigenetics will also be examined.

Consents, storage and use of samples will be underpinned by the code of practice outlined by the Human Tissue Act and Medical Research Council guidance. All human samples will be treated as donated gifts and all research will be conducted with respect and transparency.

A system of coding will be used to pseudonymise the samples obtained. Only the chief investigator will have access to the codes which will be stored in secure storage (password protected) in a locked room. The chief investigator will only have access to the secure database that would permit identification of a sample with a trial number, and subsequent identification of the participant whose material is being used. The research material will not be identifiable to the researcher and researchers, including the principle investigator, cannot link the sample to the participant. The samples will be under the custodianship of Gowrie Balasubramaniam, Chief Investigator.

Participants will be consented specifically for storage of samples up to 15 years and to be used for future, as yet unspecified, medical research into health, illness and medical treatment. This research will be subject to proper scientific and ethical review.

Some samples will be exported to countries in the European Union for further analysis. Participants will be consented specifically for this and exported material will be handled, stored, transported and disposed in accordance with the consent given. The collaborators / recipient country will ensure that the material has been handled properly and that the required standards of that collaborating institution / country have been met.

Consent will be obtained specifically for the use of DNA, although it will be made clear that the research is concerned with inflammatory changes in epigenetic DNA and subsequent changes with treatment in gout. There will be no DNA genetic testing per se that may reveal significant results such as a family genetic condition etc. We will make it at the outset of the consent that this is the case. The DNA is extracted from cells, which would otherwise be discarded if we were just using serum or plasma samples. This maximises use of samples.

For peer review only - http://bmjopen.bmj.com/site/about/guidelines.xhtml

7.12 End of trial

The recruitment period will be stopped after 19 months (15 months in duration after 4 month start-up phase) or if the trial is stopped for another reason by the steering committee, funding body or regulatory body.

The active treatment phase will be completed five days after the last participant is randomised.

The end of the trial in after the completion of final assessment, 8 weeks after the last patient is randomised.

All data available for the patient at the time of discontinuation of follow-up must be recorded in the eCRF. All reasons for discontinuation of treatment must be documented. In terminating the study, the sponsor (SUGH) and the Investigators will ensure that adequate consideration is given to the protection of the patient's interest.

The REC which gave the favourable opinion will be notified of the trial conclusion in writing. A declaration of end of a clinical trial of investigational medicinal products will be sent to the MHRA. This will be done within 90 days of the end of study as defined by the last visit / data item of the last participant in the trial. Formal notification template from the HRA website will be used: http://www.hra.nhs.uk/research-community/end-of-study-and-beyond/notifying-the-end-of-study/.

7.13 Rescue medications

Participants can take analgesia listed in the protocol as non-investigational medicinal product (co-dydramol, codeine phosphate, tramadol, and paracetamol) for relief as required, usage will be recorded. If participants get another flare during treatment, this will be recorded and a coarse of oral prednisolone 35mg po od for 5 days will be used for rescue treatment. Low dose colchicine 500micrograms po bd could be used in addition. Use of other immune modifying treatment or NSAIDS is prohibited.

For peer review only - http://bmjopen.bmj.com/site/about/guidelines.xhtml

8 TRIAL MEDICATION

There are three investigational trial medications, Anakinra, an Interleukin-1 inhibitor, intramuscular methylprednisolone acetate and its placebo, Lipofundin.

Anakinra has already marketed in the UK for the treatment of inflammatory conditions. It has been used in the treatment of acute gout we are undertaking a systematic assessment compared to the current standard of care in the UK. Hence, this falls under the remit as an Investigational Medicinal Product. Contemporary trials that use a similar mode of action have been reported with reasonable safety outcomes so some class evidence is available.

Intramuscular methylprednisolone acetate (Depo-Medrone) is the preferred depot steroid injection in the UK. It has been proven in acute inflammatory joint conditions but there are no trial looking specifically at acute gout attacks. There is a huge body of evidence with drugs using a similar mode of action and it is currently the safest option to treat acute gout attacks in patients with kidney disease. Intramuscular methylprednisolone acetate (Depo-Medrone), per se, has not been used in a trial looking at acute gout and so it falls under the remit as an investigation medicinal product.

Lipofundin MCT/LCT 10% is a milky white, sterile emulsion commonly used to provide fat and essential fatty acid for par-enteral feeding. It has been used as a placebo to depot steroid injections, including in contemporary studies looking at Canakinumab against Triamcinolone. No adverse events were reported. Injectable lipid emulsions have been used for more than forty years and the components in Lipofundin MCT/LCT are made from egg protein and soya-bean protein and these agents are commonly used carriers for non-aqueous, oil soluble drugs.

8.1.1 IMP: Anakinra

Anakinra (Kineret®) is an IL-1 receptor antagonist that is similar to the endogenous IL-1 RA that negatively regulates inflammation. There are other ways of inhibiting IL-1 action but Kineret is the only recombinant, non-glycosylated protein that differs slightly from the native form by the addition of a single methionine residue at its amino terminus (153 amino acids, MW 17.3kd). It will be supplied free of charge by the manufacturer, Swedish Orphan Biovitium.

Licensed drug name: Kineret®

8.1.2 Legal status of the drug

Anakinra has US FDA approval for use in Rheumatoid Arthritis and Cryopyrin-Associated Periodic Syndrome (CAPS). It is currently not recommended by NICE (February 2009, NICE) for RA except in a controlled clinical study or in difficult to treat cases which has not responded to methotrexate alone; it is not recommended for routine management. The Scottish Medicines Consortium has advised that Anakinra is not recommended for the treatment of rheumatoid arthritis within Scotland. Anakinra is currently not licenced for use in the treatment of acute gout.

8.1.3 Summary of Product Characteristics (SmPC)

Available from www.kineretx.com/fileadmin/user/pdfs/Kineret_Full_Prescribing_Information.pdf

Issue date: 12/2012

For peer review only - http://bmjopen.bmj.com/site/about/guidelines.xhtml

Health Research Authority

Anakinra vs. Steroids for the Treatment of Gout Attacks in Renal Patients (ASGARD): A Feasibility Study

EudraCT no. 2015-001787-19

The manufacture's recommend Kineret not be used in patients with hypersensitivity to E-Coli derived proteins or any components of the product. Administration should be discontinued if a patient develops a serious infection and should not be initiated in a patient with active infection. Safety of the product in immunosuppressed or patients with chronic infections has not been evaluated. The recommendations are derived from RA trials with a prolonged duration of treatment for chronic inflammation. Our study only requires five days treatment and cohorts have been published for acute gout attacks in patients with active infection. Furthermore, the initial human use of Anakinra was in sepsis syndrome where patients were given a loading dose of 100mg followed by an infusion or 1-2mg/kg per hour did not show a worse outcome (Fisher et al. 1994).

Pharmacodynamics and pharmacokinetics

Bioavailability after an injection is 95% and maximum plasma concentrations were reached after 3-7 hours after subcutaneous administration. The terminal half-life is between 4-6 hours and clearance was dependent on creatinine clearance and weight. Plasma clearance falls to 70-75% in patients with severe or end-stage kidney disease (eGFR < 30 mL/min/m^2) (Yang et al, 2003). Anakinra has been used in patients on dialysis on an alternate day regime for 14 weeks (Hung et al, 2011).

8.1.4 Drug storage and supply

The investigational product will be supplied by the manufacturer free of charge. It will be stocked by the co-ordinating trial site / sponsor (SUFT) and distributed to other sites for storage. The equivalent placebo will also be provided by the manufacturer of Anakinra and handled as the investigational product.

The IMP is being provided for use in the trial but hospital can purchase IMP for used on their patients. Anakinra and its equivalent placebo does not require re-constitution.

Dosage form: It is supplied as a pre-filled glass syringe with 27 gauge needles as a sterile, clear, colourless-to-white, preservative free solution for daily subcutaneous (SC) injection. Each pre-filled syringe contains 0.67mLs (100mg) Anakinra in a solution (pH 6.5) containing EDTA (0.12mg), Sodium Chloride (5.48mg), Sodium Citrate (1.29mg), and polysorbate 80 (0.70mg) in water for injection.

Packaging and storage: Kineret is dispensed in a 4X7 syringe dispensing pack containing 28 syringes and also a 1X7 syringe dispensing pack. Kineret should be stored in the refrigerator at 2-8 °C degree Celsius (36 to 46 Fahrenheit-US). Shelf life is 3 years. It should be protected from light.

Disposal: After use patients will place syringe, without re-sheathing, into a puncture-resistant container (sharp bin). This will be returned to the trial site on day 7 and the number of used needles will be counted. Syringes will be disposed as per normal hospital policy on disposal of sharps.

8.1.5 Preparation and labelling of Investigational Medicinal Product

The drug will be supplied in a pre-filled syringe. It will have an equivalent placebo.

8.1.6 Dosage schedules

At recruitment and randomisation, participants enrolled into the Anakinra arm will have a D1 SC injection of 100mg Anakinra. This will continue daily for 5 days in total (i.e. D1 to D5). Patients in the steroid arm for investigation product will have similar regime with the equivalent placebo.

The glass syringes are pre-filled to 100mg.

For peer review only - http://bmjopen.bmj.com/site/about/guidelines.xhtml

8.1.7 Dosage modifications

There will be no dosage modification.

8.1.8 Drug Accountability

Southend University Hospital will act as a hub pharmacy and receive supply of Anakinra and its equivalent placebo from the manufacturer. Stand-alone pharmacies will receive an agreed number of packs depending on the expected recruitment rate for the size of the centre.

Documentation

Accurate records of all drug shipments, medication dispensed, and all drug returned, must be maintained. The inventory record must be available for inspection by the sponsor but information that may lead to un-blinding will be minimised. The site PI or CI does not destroy any unused drug unless directed to by the sponsor. Any destroyed drug must be destroyed according to hospital procedures and properly accounted for. At the conclusion of the study, the overall numbers of the drug shipped to the centre, and the number destroyed or returned, will be provided by the pharmacy, and an account given of any discrepancy.

Dispensing procedure

Once a research team has undertaken randomisation for a patient after recruitment, they will inform pharmacy. Pharmacy will be given the allocation details and will prepare appropriate medication or placebo accordingly. Comparator medication/placebo will be drawn up in a clouded syringe and recorded. The allocated concealed medications will be given to research team or ward staff to administer.

Stopping rule

Patients who develop a hypersensitivity, including anaphylactic reactions and angioedema should stop treatment and seek immediate medical advice. The trial should be informed.

If patients develop sign of a systemic infection with fever and chills, this should be discussed with the medical team. If the participant develops an open sore that might pre-dispose to infection, this should be discussed with the medical team.

No toxicity has been described with short term use and in fact, Anakinra has been used in acutely unwell patients with con-current infection and no adverse long-term effects.

The dosage of the drug cannot be modified for any reason.

8.1.9 Known drug reactions and interaction with other therapies

Concurrent administration of Anakinra and Etanercept has been associated with increased risk of serious infections and neutropenia compared to Etanercept alone in RA patients. Concurrent administration with other anti-TNF therapy is not advised. This should not occur as these patients are excluded from the trial.

Due to the anti-inflammatory nature of Anakinra, agents that use the CYP450 enzymes that are suppressed during inflammation may theoretically have altered metabolism due to resolution of

For peer review only - http://bmjopen.bmj.com/site/about/guidelines.xhtml

Health Research Authority

Anakinra vs. Steroids for the Treatment of Gout Attacks in Renal Patients (ASGARD): A Feasibility Study

EudraCT no. 2015-001787-19

inflammation. Actions / levels of common drugs (Warfarin, Phenytoin) that use this pathway should ideally be monitored, however, no formal adverse events have been reported.

8.1.10 Concomitant medication

Patients should not receive concurrent immunosuppressive medication or biologics especially anti-TNF therapy.

Anakinra does has not been shown to accumulate even after prolonged (months) courses of treatment and should be cleared. The half-life 4-6 hours, patients could have other treatment after 7 days of randomisation.

Live vaccines should not be given con-currently.

Patients are not allowed Colchicine which has to be used in caution in patients with renal impairment due to risk of accumulation. Colchicine treatment will confound results and conclusion of the trial. Patients requiring analgesia can have Paracetamol, Tramadol and Codeine, but not within 4 hours of pain assessment.

8.1.11 Trial restrictions

There is inadequate information of Anakinra on pregnant and nursing mothers. Female participants of child-bearing age should continue to practice reliable means of contraception (such as physical barrier [patient and partner], contraceptive pill or patch, spermicide and barrier, or intrauterine devices) as stated in the exclusion criteria.

8.1.12 Assessment of compliance

Participants will be given diary cards at randomisation. Injection sites will be checked and patients will return syringes in a non-puncture container for empty syringes to be checked. Participants will also have visits at day 1, 2, 4, and 7 to check with compliance.

Patient will receive phone call to prompt injection and recording of diary cards to ensure feasibility trial meets criteria for success.

Documentation of injection sites and empty syringes will be undertaken by the trial investigator. Patients will be asked to bring non-puncture bins with empty syringes. Diary card will include time of injection.

Any non-compliance will be documented by the trial investigators and data will be recorded for study analysis, this is a key feature of the feasibility aspect of the trial. The percentage of noncompliance acceptable for patient to continue on the trial is <80% noncompliance equates to patient withdrawal (this includes compliance with IMP and study procedures e.g. visit window, refusal of study specific assessments). Patients who are withdrawn from the study for this reason will continue to be followed up.

Any omissions or delays will be documented in the eCRF to record the reason and information will be use by the Trial Management Committee to ensure success of the trial.

For peer review only - http://bmjopen.bmj.com/site/about/guidelines.xhtml

8.2.1 IMP: Intramuscular methylprednisolone (Depo-Medrone)

Glucocorticoids are adrenocortical steroids. Naturally occurring ones are hydrocortisone and cortisone are used for replacement in adrenocortical deficiency states.

It is indicated in a number of conditions when oral therapy is not feasible or the strength, dosage form, and route of administration of the drug lend the preparation to the treatment of the condition. It is used in all sorts of conditions for many years. It can be used for short-term administration for acute joint conditions.

It will only be used as an intra-muscular agent and so discussion will only be restricted to this. It will be used as a one off intra-muscular injection and although cautions described below generally apply to prolonged use, there is insufficient data on the short term one off use. We will monitor for any adverse events in our study.

Intramuscular methylprednisolone 40mg/ml (Methylprednisolone Acetate BP 40mg/mL) is an anti-inflammatory glucocorticoid for intramuscular, intra-articular, soft tissue or intra-lesion injection. Methylprednisolone acetate is a synthetic glucocorticoid. It has greater anti-inflammatory potency than prednisolone and less tendency than prednisolone to induce sodium and water retention.

Intramuscular methylprednisolone (Depo-Medrone) vials are intended for single use only and intra-muscular injection is used for sustained systemic effect; 40mg (1mL), 80mg (2mL) and 120ml (3mL). Dose must be individualised and depends on the condition being treated and its severity. Each mL contains methylprednisolone acetate, polyethylene glycol 3350 (29.5mg), polysorbate 80 (1.97mg), monobasic sodium phosphate (6.9mg), dibasic sodium phosphate USP (1.44mg), Benzyl alcohol as preservative 9.3mg. Sodium Chloride added to adjust tonicity.

It is manufactured by Pharmacia.

Licensed drug name: Depo-Medrone®

8.2.2 Legal status of the drug

It is a prescription only medication in the United Kingdom.

8.2.3 Summary of Product Characteristics (SmPC)

Available from http://www.medicines.org.uk/emc/medicine/3549

Issue date: 03/1989

Intramuscular methylprednisolone acetate is contra-indicated where there is known hypersensitivity to components and in systemic infection unless specific anti-infective therapy is employed

Following a single dose of intramuscular methylprednisolone, plasma cortisol levels are reduced and there is evidence of hypothalamic-pituitary-adrenal (HPA) axis suppression. This suppression lasts for a variable period of up to 4 weeks. The usual dynamic tests of HPA axis function can be used to diagnose evidence of impaired activity (e.g. Synacthen test).

Because rare instances of anaphylactic reactions have occurred in patients receiving parenteral corticosteroid therapy, appropriate precautionary measures should be taken prior to administration, especially when the patient has a history of drug allergy.

For peer review only - http://bmjopen.bmj.com/site/about/guidelines.xhtml

NHS
Health Research Authority

Anakinra vs. Steroids for the Treatment of Gout Attacks in Renal Patients (ASGARD): A Feasibility Study

EudraCT no. 2015-001787-19

Corticosteroids may mask some signs of infection, and new infections may appear during their use. Suppression of the inflammatory response and immune function increases the susceptibility to fungal, viral and bacterial infections and their severity. The clinical presentation may often be atypical and may reach an advanced stage before being recognised.

Live vaccines should not be given to individuals with impaired immune responsiveness. The antibody response to other vaccines may be diminished.

The slower rate of absorption by intramuscular administration should be recognised.

Most special precaution relate to prolonged use of glucocorticoids

Particular care is required when considering the use of systemic corticosteroids in patients with osteoporosis (post-menopausal females are particularly at risk), hypertension or congestive heart failure, existing or previous history of severe affective disorders (especially previous steroid psychosis), diabetes mellitus (or a family history of diabetes), history of tuberculosis, glaucoma (or a family history of glaucoma), previous corticosteroid-induced myopathy, liver failure or cirrhosis, renal insufficiency, epilepsy, peptic ulceration, fresh intestinal anastomoses, predisposition to thrombophlebitis , abscess or other pyogenic infections, ulcerative colitis, diverticulitis, myasthenia gravis, ocular herpes simplex, for fear of corneal perforation, hypothyroidism.

Patients and/or carers should be warned that potentially severe psychiatric adverse reactions may occur with systemic steroids. Particular care is required when considering the use of systemic corticosteroids in patients with existing or previous history of severe affective disorders in themselves or in their first degree relatives. These would include depressive or manic-depressive illness and previous steroid psychosis.

The common adverse effects of systemic corticosteroids may be associated with more serious consequences in old age.

Adverse effects include anaphylactic reaction or allergic reactions, dyspepsia, peptic ulceration with perforation and haemorrhage, abdominal distension, oesophageal ulceration, oesophageal candidiasis, acute pancreatitis, perforation of bowel. Increases in alanine transaminase (ALT, SGPT) aspartate transaminase (AST, SGOT) and alkaline phosphatase have been observed following corticosteroid treatment. These changes are usually small, not associated with any clinical syndrome and are reversible upon discontinuation.

Proximal myopathy, osteoporosis, vertebral and long bone fractures, avascular osteonecrosis, tendon rupture, aseptic necrosis, muscle weakness. Sodium and water retention, potassium loss, hypertension, hypokalaemic alkalosis, congestive heart failure in susceptible patients.

Suppression of the hypothalamo-pituitary-adrenal axis, growth suppression in infancy, childhood and adolescence, menstrual irregularity and amenorrhoea. Cushingoid facies, hirsutism, weight gain, impaired carbohydrate tolerance with increased requirement for antidiabetic therapy, negative nitrogen and calcium balance. Increased appetite.

A wide range of psychiatric reactions including affective disorders (such as irritable, euphoric, depressed and labile mood psychological dependence and suicidal thoughts), psychotic reactions (including mania, delusions, hallucinations and aggravation of schizophrenia), behavioural disturbances, irritability, anxiety, sleep disturbances, and cognitive dysfunction including confusion and

For peer review only - http://bmjopen.bmj.com/site/about/guidelines.xhtml

Health Research Authority

amnesia have been reported for all corticosteroids. Psychological effects have been reported on withdrawal of corticosteroids; the frequency is unknown.

Increased intra-ocular pressure, glaucoma, papilloedema, cataracts with possible damage to the optic nerve, corneal or scleral thinning, exacerbation of ophthalmic viral or fungal disease, exophthalmos.

Leucocytosis, hypersensitivity including anaphylaxis, thrombo-embolism, nausea, vertigo.

Pharmacodynamics and pharmacokinetics

One in-house study of eight volunteers determined the pharmacokinetics of a single 40 mg intramuscular dose of intramuscular methylprednisolone. The average of the individual peak plasma concentrations was 14.8 ± 8.6 ng/mL, the average of the individual peak times was 7.25 ± 1.04 hours, and the average area under the curve (AUC) was 1354.2 ± 424.1 ng/mL x hrs (Day 1-21).

Methylprednisolone is widely distributed into the tissues, crosses the blood-brain barrier, and is secreted in breast milk. Its apparent volume of distribution is approximately 1.4 L/kg. The plasma protein binding of methylprednisolone in humans is approximately 77%.

In humans, methylprednisolone is metabolized in the liver to inactive metabolites; the major ones are 20α-hydroxymethylprednisolone and 20β-hydroxymethylprednisolone. Metabolism in the liver occurs primarily via the CYP3A4.

Methylprednisolone, like many CYP3A4 substrates, may also be a substrate for the ATP-binding cassette (ABC) transport protein p-glycoprotein, influencing tissue distribution and interactions with other medicines.

The mean elimination half-life for total methylprednisolone is in the range of 1.8 to 5.2 hours. Total clearance is approximately 5 to 6 mL/min/kg.

No dosing adjustments are necessary in renal failure. Methylprednisolone is haemodialysable

8.2.4 Drug storage and supply

Shelf-life of the medicinal product as packaged for sale: 60 months. Intramuscular methylprednisolone acetate should not be mixed with any other fluid. Discard any remaining suspension after use. The nature of the container is a type I flint glass vial with a butyl rubber plug and metal seal. Each vial contains 1 ml, 2ml, or 3 ml of intramuscular methylprednisolone 40 mg/ml.

Disposal: After use patients will place syringe, without re-sheathing, into a puncture-resistant container (sharp bin). Syringes will be disposed as per normal hospital policy on disposal of sharps.

8.2.5 Preparation and labelling of Investigational Medicinal Product

The drug should be available from the local hospital pharmacy. It will be drawn up by a member of the research team not involved with the trial.

8.2.6 Dosage schedules

At recruitment and randomisation, participants enrolled into the intramuscular methylprednisolone arm will have a one off intramuscular methylprednisolone 120mg/3mL intra-muscular injection. Patients in the other arm for investigation product will have similar regime with the equivalent placebo.

For peer review only - http://bmjopen.bmj.com/site/about/guidelines.xhtml

Health Research Authority

Anakinra vs. Steroids for the Treatment of Gout Attacks in Renal Patients (ASGARD): A Feasibility Study

EudraCT no. 2015-001787-19

8.2.7 Dosage modifications

There will be no dosage modification.

8.2.8 Drug Accountability

Drug accountability will be by the local pharmacy.

Documentation

Accurate records of all drug shipments, medication dispensed, and all drug returned, must be maintained. The inventory record must be available for inspection by the sponsor but information that may lead to un-blinding will be minimised. The site PI or CI does not destroy any unused drug unless directed to by the sponsor. Any destroyed drug must be destroyed according to hospital procedures and properly accounted for. At the conclusion of the study, the overall numbers of the drug used at the centre, and the number destroyed or returned, will be provided by the pharmacy, and an account given of any discrepancy.

Dispensing procedure

Once a research team has undertaken randomisation for a patient after recruitment, they will inform pharmacy. Pharmacy will be given the allocation details and will prepare appropriate medication or placebo accordingly. Comparator medication/placebo will be drawn up in a clouded syringe and recorded. The allocated concealed medications will be given to research team or ward staff to administer.

Stopping rule

Patients who develop a hypersensitivity, including anaphylactic reactions and angioedema should stop treatment and seek immediate medical advice. The trial should be informed.

If patients develop sign of a systemic infection with fever and chills, this should be discussed with the medical team. If the participant develops an open sore that might pre-dispose to infection, this should be discussed with the medical team.

8.2.9 Known drug reactions and interaction with other therapies

- Drugs that induce hepatic enzymes, such as rifampicin, rifabutin, carbamazepine, phenobarbitone, phenytoin, primidone, and aminoglutethimide enhance the metabolism of corticosteroids and its therapeutic effects may be reduced.
- Drugs such as erythromycin and ketoconazole may inhibit the metabolism of corticosteroids and thus decrease their clearance.
- Steroids may reduce the effects of anticholinesterases in myasthenia gravis. The desired effects of hypoglycaemic agents (including insulin), anti-hypertensives and diuretics are antagonised by corticosteroids, and the hypokalaemic effects of acetazolamide, loop diuretics, thiazide diuretics and carbenoxolone are enhanced.
- The efficacy of coumarin anticoagulants may be enhanced by concurrent corticosteroid therapy and close monitoring of the INR or prothrombin time is required to avoid spontaneous bleeding.
- The renal clearance of salicylates is increased by corticosteroids and steroid withdrawal may result in salicylate intoxication. Salicylates and non-steroidal anti-inflammatory agents should be used cautiously in conjunction with corticosteroids in hypothrombinaemia.

For peer review only - http://bmjopen.bmj.com/site/about/guidelines.xhtml

- Steroids have been reported to interact with neuromuscular blocking agents such as pancuronium with partial reversal of the neuromuscular block.

8.2.10 Concomitant medication

Patients should not receive concurrent immunosuppressive medication. Live vaccines should not be given con-currently. Caution should be used when using medications described in section 8.2.9.

8.2.11 Trial restrictions

Steroids should be ideally avoided during pregnancy. Female participants of child-bearing age should continue to practice reliable means of contraception (such as physical barrier [patient and partner], contraceptive pill or patch, spermicide and barrier, or intrauterine devices) as stated in the exclusion criteria.

8.2.12 Assessment of compliance

Participants will be given the single dose of intra-muscular injection on Day 1.

8.3.1 IMP: Lipofundin MCT/LCT 10%

Lipofundin MCT/LCT 10% is an emulsion preparation for injection. It is composed of soya oil. Medium-chain Triglycerides, glycerol, egg lecithin, alpha tocopherol (vitamin E), sodium oleate. These agents are commonly used as carriers for fat soluble drugs. Lipofundin MCT/LCT specifically is used for the provision of calories and essential fatty acids. It has been used as a control for other intramuscular injection steroid emulsion injection in contemporary studies. It is normally used in amounts of over 100mls and the side-effects and contra-indications are for these form of administration. We are using this agent as an appropriate placebo for intramuscular methylprednisolone. This is to keep the appearance of the intramuscular methylprednisolone and its placebo the same. The volume used for injection will be 3mL, the same as the volume of intramuscular methylprednisolone. We do not anticipate any side-effects with this amount. There should not be any biological effects with this amount and there are no active agents in the preparation.

8.3.2 Legal status of the drug

Prescription only medication.

8.3.2 Summary of Product Characteristics (SmPC)

Emulsion for injection in glass bottle, contents 100mL, 250mL and 500mL.

8.3.4 Drug storage and supply

Store below 25°C and protect from freezing. If accidentally frozen, discard bottle. Unused contents must be discarded and should not be stored for later use. Do not use bottles showing evidence of phase separation.

8.3.5 Preparation and labelling of Investigational Medicinal Product

The drug will be drawn up for injection by a member of the research team who is not involved with the study.

8.3.6 Dosage schedules

For peer review only - http://bmjopen.bmj.com/site/about/guidelines.xhtml

NHS
Health Research Authority

Anakinra vs. Steroids for the Treatment of Gout Attacks in Renal Patients (ASGARD): A Feasibility Study

EudraCT no. 2015-001787-19

3mL will be injected as an intra-muscular injection on Day 1.

8.3.7 Dosage modifications

Lipofundin MCT/LCT is used as a placebo injection for the purpose of this study. It is not being used for intravenous feeding that it would normally be used for.

8.3.8 Drug Accountability

Drug accountability will be by the local pharmacy.

Documentation

Accurate records of all medication dispensed, and all drug returned, must be maintained. The inventory record must be available for inspection by the sponsor but information that may lead to un-blinding will be minimised. The site PI or CI does not destroy any unused drug unless directed to by the sponsor. Any destroyed drug must be destroyed according to hospital procedures and properly accounted for. At the conclusion of the study, the overall numbers of the drug used at the centre, and the number destroyed or returned, will be provided by the pharmacy, and an account given of any discrepancy.

Dispensing procedure

Once a research team has undertaken randomisation for a patient after recruitment, they will inform pharmacy. Pharmacy will be given the allocation details and will prepare appropriate medication or placebo accordingly. Comparator medication/placebo will be drawn up in a clouded syringe and recorded. The allocated concealed medications will be given to research team or ward staff to administer.

Stopping rule

Patients who develop a hypersensitivity, including anaphylactic reactions and angioedema should stop treatment and seek immediate medical advice. The trial should be informed.

Documentation

Accurate records of all drug shipments, medication dispensed, and all drug returned, must be maintained. The inventory record must be available for inspection by the sponsor but information that may lead to un-blinding will be minimised. The site PI or CI does not destroy any unused drug unless directed to by the sponsor. Any destroyed drug must be destroyed according to hospital procedures and properly accounted for. At the conclusion of the study, the overall numbers of the drug shipped to the centre, and the number destroyed or returned, will be provided by the pharmacy, and an account given of any discrepancy.

Dispensing procedure

Once a research team has undertaken randomisation for a patient after recruitment, they will inform pharmacy. Pharmacy will be given the allocation details and will prepare appropriate medication or placebo accordingly. Comparator medication/placebo will be drawn up in a clouded syringe and recorded. The allocated concealed medications will be given to research team or ward staff to administer.

For peer review only - http://bmjopen.bmj.com/site/about/guidelines.xhtml

8.3.9 Assessment of compliance

Participants will be given intra-muscular injection on Day 1.

8.4 Name and description of each Non-Investigational Medicinal Product (NIMP)

A pre-specified list of analgesics can be used in participants or potential participants. This will avoid inadvertent treatment of patients with potentially harmful agents such as NSAIDS or Colchicine, it would also allow patients to be given the opportunity to participate in the trial. These analgesics should not be used within four hours of project assessment of pain. Patients who are primed for the study can use these agents at home before presenting to secondary care.

8.4.1 NIMP: Paracetamol

Paracetamol is the most commonly used and widely used analgesic in the UK. It is used for the relief of mild to moderate pain. It also has antipyretic actions and can be used in febrile conditions. It can be given 500mg to 1g every four to six hourly as required. It is usually administered orally but can be administered intravenously as well.

It is contraindication in patients with known hypersensitivity to paracetamol or other constituents in the tablets. It should not be taken with other paracetamol-containing products. Immediate medical attention should be sought in the event of an overdose.

There are few interactions with other medicinal products but it is only for short-term use in this study. Metoclopramide and Domperidone may increase speed of absorption of paracetamol. Cholestyramine may reduce adsorption if given within one hour of paracetamol.

Paracetamol should be avoided in pregnancy but no adverse effects have been shown when used in the recommended dosage.

Adverse effects of Paracetamol are rare but hypersensitivity including skin rash may occur. There have been reports of blood dyscrasias including thrombocytopenia, neutropenia, pancytopenia, leukopenia and agranulocytosis but these were not necessarily causality related to Paracetamol.

Paracetamol is readily absorbed from the gastrointestinal tract. Peak plasma concentrations occur about 10 to 60 minutes after oral doses. Paracetamol is distributed into most body tissues. It crosses the placenta and is present in breast milk. Plasma-protein binding is negligible at usual therapeutic concentrations but increases with increasing concentrations. It is metabolised in the liver. A minor hydroxylated metabolite which is usually produced in very small amounts by mixed-function oxidases in the liver and which is usually detoxified by conjugation with liver glutathione may accumulate following paracetamol overdose and cause tissue damage. It is excreted in the urine, mainly as the glucuronide and sulphate conjugates. The elimination half-life varies from about 1 to 4 hours.

Shelf-life is for three years for PE tablet containers and five years for blisters and PP tablet containers. It should be stored below 25°C in a dry place. It should be protected from light.

8.4.2 NIMP: Codeine Phosphate

Codeine phosphate as an analgesic for the relief of mild to moderate pain and for acute moderate pain which is not considered to be relieved by other analgesics such as paracetamol. Codeine should be used at the lowest effective dose for the shortest period of time. This dose may be taken, up to 4 times

For peer review only - http://bmjopen.bmj.com/site/about/guidelines.xhtml

a day at intervals of not less than 6 hours. Maximum daily dose should not exceed 240mg. 30-60mg every four hours to a maximum dosage of 240mg daily.

Codeine is an analgesic with uses similar to those of morphine but it is much less potent as an analgesic and has only mild sedative effects. It is also used in the treatment of cough and diarrhoea.

Codeine is a centrally acting weak analgesic. Codeine exerts its effect through μ opioid receptors, although codeine has low affinity for these receptors, and its analgesic effect is due to its conversion to morphine. Codeine, particularly in combination with other analgesics such as paracetamol, has been shown to be effective in acute nociceptive pain.

The analgesic effect is not materially enhanced by increasing the dose to a greater level than that recommended above. Dosage should be reduced in the elderly where there is impairment of hepatic or renal function. It is only intended for oral use.

Contraindications to use of codeine phosphate include known hypersensitivity to codeine, other opioids or any of the excipients in the tablets, acute respiratory depression, severe obstructive airways disease (e.g. emphysema), asthma (should not be administered during an asthma attack), hepatic failure, head injuries or conditions where intracranial pressure is raised, acute alcoholism, risk of paralytic ileus and women during breastfeeding.

Patient with metabolic derangements such as those whom it is known they are CYP2D6 ultra-rapid metabolisers or with those rare hereditary problems of galactose intolerance, the Lapp lactase deficiency or glucose-galactose malabsorption should not take this medicine.

Codeine phosphate should be used with caution if there is a possible risk of CNS excitation or depression, convulsions, or a history of drug abuse or dependence (including alcoholism). It should be used with caution in patients with hepatic impairment, renal impairment, hypothyroidism, inflammatory bowel disease (codeine reduces peristalsis, increases tone and segmentation in the bowel and can raise colonic pressure, therefore should be used with caution in diverticulitis, acute colitis, diarrhoea associated with pseudomembranous colitis or after bowel surgery), gall bladder disease or gall stones (opioids may cause biliary contraction and should be avoid in biliary disorders), gastro-intestinal surgery, urinary tract surgery (following recent surgery patients will be more prone to urinary retention caused directly by spasm of the urethral sphincter, and via constipation caused by codeine).

It should be used with caution is patient with a history of phaeochromocytoma (opioids may stimulate catecholamine release by inducing the release of endogenous histamine), prostatic hypertrophy, adrenocortical insufficiency, e.g. Addison's disease, hypotension and shock, myasthenia gravis, reduced respiratory function or history of asthma, pregnancy and breast feeding.

Elderly patients may metabolise and eliminate opioid analgesics more slowly than younger patients Codeine is metabolised by the liver enzyme CYP2D6 into morphine, its active metabolite. If a patient has a deficiency or is completely lacking this enzyme an adequate analgesic effect will not be obtained. If the patient is an extensive or ultra-rapid metaboliser there is an increased risk of developing side effects of opioid toxicity even at commonly prescribed doses. These patients convert codeine into morphine rapidly resulting in higher than expected serum morphine levels.

General symptoms of opioid toxicity include confusion, somnolence, shallow breathing, small pupils, nausea, vomiting, constipation and lack of appetite. In severe cases this may include symptoms of circulatory and respiratory depression, which may be life-threatening and very rarely fatal.

For peer review only - http://bmjopen.bmj.com/site/about/guidelines.xhtml

Health Research Authority

Anakinra vs. Steroids for the Treatment of Gout Attacks in Renal Patients (ASGARD): A Feasibility Study

EudraCT no. 2015-001787-19

Interactions

- MAOIs (e.g. linezolid, moclobemide, selegiline) - is not recommended due to the possible risk of excitation or depression, avoid concomitant use and for 2 weeks after discontinuation of MAOI
- Alcohol – enhanced sedative and hypotensive effect, and increased risk of respiratory depression.
- Sedative antihistamines, hypnotics and anxiolytics - enhanced risk of sedative effect, and increased risk of respiratory depression
- Anticholinergics (e.g. atropine) - risk of severe constipation which may lead to paralytic ileus, and /or urinary retention
- Metoclopramide and domperidone – antagonise effect on GI activity
- Anti-diarrhoeal drugs (e.g. loperamide, kaolin) – increased risk of severe constipation.
- Anaesthetics - enhanced sedative and hypotensive effect
- Tricyclic antidepressants - enhanced sedative effect
- Antipsychotics - enhanced sedative and hypotensive effect
- Opioid antagonists e.g. buprenorphine, naltrexone, naloxone – may precipitate withdrawal symptoms
- Quinidine- reduced analgesic effect
- Antihypertensive drugs - enhanced hypotensive effect.
- Ciprofloxacin, - avoid premedication with opioids as they reduce plasma ciprofloxacin concentration.
- Ritonavir may increase plasma levels of opioid analgesics such as codeine.
- Mexiletine - delayed absorption of mexiletine.
- Cimetidine inhibits the metabolism of opioid analgesics causing increased plasma concentration of codeine.

Pregnancy and lactation

Risk benefit must be considered because opioid analgesics cross the placenta. Regular use during pregnancy may cause physical dependence in the foetus, leading to withdrawal symptoms in the neonate. During labour opioids enter the foetal circulation and may cause respiratory depression in the neonate. Respiratory malformation in neonates may be associated with exposure to codeine during pregnancy. Gastric stasis and a risk of inhalation pneumonia could occur in the mother during labour. Administration should be avoided during the late stages of labour and during the delivery of a premature infant. Codeine should not be used during breastfeeding.

Adverse effects

- Immune system disorders: (may be caused by histamine release) – including rash, urticaria, pruritus, difficulty breathing, increased sweating, redness or flushed face.
- Nervous system disorders: confusion, drowsiness, malaise, tiredness, vertigo, dizziness, changes in mood, hallucinations, CNS excitation (restlessness/excitement), convulsions, mental depression, headache, or nightmares, raised intracranial pressure, tolerance or dependence, dysphoria, hypothermia.
- Eye disorders: - miosis, blurred or double vision.
- Cardiac disorders: bradycardia, palpitations, hypotension, orthostatic hypotension, tachycardia.
- Respiratory, thoracic and mediastinal disorders: respiratory depression with larger doses.

For peer review only - http://bmjopen.bmj.com/site/about/guidelines.xhtml

- Gastrointestinal disorders: constipation (too constipating for long-term use), biliary spasm, nausea, vomiting, dry mouth.
- Musculoskeletal, connective tissue and bone density: muscle rigidity.
- Renal and urinary disorders: ureteral spasm, antidiuretic effect, urinary retention.
- Reproductive system and breast disorders: decrease in libido and potency.
- Withdrawal effects: abrupt withdrawal precipitates a withdrawal syndrome. Symptoms may include tremor, insomnia, restlessness, irritability, anxiety, depression, anorexia, nausea, vomiting, diarrhoea, sweating, lacrimation, rhinorrhoea, sneezing, yawning, piloerection, mydriasis, weakness, pyrexia, muscle cramps, dehydration, and increase in heart rate, respiratory rate and blood pressure. NOTE - tolerance diminishes rapidly after withdrawal so a previously tolerated dose may prove fatal.
- The effects in overdose will be potentiated by simultaneous ingestion of alcohol and psychotropic drugs. Central nervous system depression, including respiratory depression, may develop but is unlikely to be severe unless other sedative agents have been co-ingested, including alcohol, or the overdose is very large. The pupils may be pin-point in size; nausea and vomiting are common. Hypotension and tachycardia are possible but unlikely. Management consists of general symptomatic and supportive measures including a clear airway and monitoring of vital signs until stable. Consider activated charcoal if an adult presents within one hour of ingestion of more than 350mg.
- Give naloxone if coma or respiratory depression is present. Naloxone is a competitive antagonist and has a short half-life so large and repeated doses may be required in a seriously poisoned patient. Observe for at least four hours after ingestion, or eight hours if a sustained release preparation has been taken.

8.4.3 NIMP: Co-Codamol

Co-codamol 8/500mg tablets contain codeine phosphate (8mg) and paracetamol (500mg). The cautions should be exercised as above. The amount of codeine is less and the expected side-effect is less.

8.4.4 NIMP: Co-Drydramol

Co-drydramol consists of Paracetamol (500mg) and Dihydrocodiene Tartrate (10mg). It is used for relief of mild to moderate pain. Dihydrocodiene is a member of the codeine family of opioid analgesics metabolised is a similar manner to codeine i.e. morphine and norcodiene. The same cautions use for codeine should be applied here. Co-drydramol is not under the same restrictions as codeine phosphate and co-codamol, both of which are on the list of controlled drugs under the misuse of drugs legislation. Co-drydramol is more readily dispensed by emergency departments and acute medical units.

8.4.5 NIMP: Tramadol

Tramadol can be used for the treatment of moderate to severe pain. The dose should be adjusted to the intensity of the pain and the sensitivity of the individual patient. The lowest effective dose for analgesia should generally be selected. The capsules are taken orally, independent of meals, swallowed whole with water. The dose is 50-100mg 3-4 times daily. A total daily dose of 400mg should not be exceeded.

Tramadol is a centrally acting synthetic analgesic compound. It is a non-selective pure agonist at mu, delta and kappa opioid receptors with a higher affinity for the mu receptor. Tramadol opioid activity

For peer review only - http://bmjopen.bmj.com/site/about/guidelines.xhtml

derives from low affinity binding of the parent compound to mu-opioid receptors and higher affinity binding of the active metabolite, O-desmethyl tramadol. Compared to morphine, tramadol does not show respiratory depression when given within the analgesic dosage interval. The gastrointestinal motility is not affected. There is minimal effect on the cardiovascular system. The contribution to human analgesia of tramadol relative to the active metabolite is unknown. Tramadol has an antitussive effect. Animal studies have revealed a reduced dependence potential compared with morphine and a very slight tolerance potential.

Tramadol should under no circumstances be administered for longer than absolutely necessary. If long-term pain treatment with tramadol is necessary in view of the nature and severity of the illness, then careful and regular monitoring should be carried out (if necessary with breaks in treatment) to establish whether and to what extent further treatment is necessary.

Caution should be used in patients over the age of 75. Drug elimination may be prolonged in patients with renal impairment and a prolongation of the dosage intervals should be carefully considered according to the patient's requirements. For patients with creatinine clearance <30ml/min, the dosage interval should be increased to 12 hours. Tramadol is not recommended in patients with severe renal impairment (creatinine clearance <10ml/min). Tramadol is removed very slowly by haemodialysis or haemofiltration so post-dialysis administration to maintain analgesia is not usually necessary.

Drug elimination of tramadol may also be prolonged in patients with hepatic impairment. The usual dosage should be divided in 2, or the dosage interval should be extended to 12 hours. In these patients prolongation of the dosage intervals should be carefully considered according to the patient's requirements. In severe hepatic impairment, the product is contraindicated.

Tramadol is contraindicated in patients who previously demonstrated hypersensitivity to tramadol or any of the other ingredients in the capsule, patients who have acute intoxication with central nervous system depressants (alcohol, hypnotics, centrally acting analgesics, opioids, psychotropic drugs), patients receiving monoamine oxidase inhibitors or within two weeks of their withdrawal, patients with severe hepatic impairment, severely impaired kidney function (creatinine clearance less than 10ml/min), severe respiratory impairment and with epilepsy not controlled by adequate treatment. It should not be used in opioid-dependent patients.

It should be use with caution in patients with withdrawal symptoms, patients with a history of drug dependence and abuse, patients sensitive to opiates, patients with head injury, increased intracranial pressure, impairment of hepatic and renal function, decreased level of consciousness and in patients prone to convulsive disorder or in shock. Caution should be used in treating patients with respiratory depression, or if concomitant CNS depressant drugs are being administered.

Interactions

- Concomitant use of carbamazepine or concomitant intake of alcohol with tramadol is not recommended
- Buprenorphine and other mixed agonists-antagonists, naltrexone
- Caution with the use of psych-active medicines or substances including alcohol
- Tramadol should not be combined with MAO inhibitors. In patients treated with MAO inhibitors in the 14 days prior to the use of the opioid pethidine, life threatening interactions on the central nervous system, respiratory and cardiovascular function have been observed. The same interactions with MAO inhibitors cannot be ruled out during treatment with Tramadol.

For peer review only - http://bmjopen.bmj.com/site/about/guidelines.xhtml

Health Research Authority

Anakinra vs. Steroids for the Treatment of Gout Attacks in Renal Patients (ASGARD): A Feasibility Study

EudraCT no. 2015-001787-19

- Simultaneous or previous administration of carbamazepine (enzyme inducer) may reduce the analgesic effect and shorten the duration of action.
- The combination with mixed agonist/antagonists (e.g. buprenorphine, nalbuphine, pentazocine) and tramadol is not advisable, because the analgesic effect of a pure agonist may be theoretically reduced in such circumstances.
- Tramadol can induce convulsions and increase the potential for selective serotonin re-uptake inhibitors, (SSRIs), serotonin-norepinephrine reuptake inhibitors (SNRIs), tricyclic anti-depressants, anti-psychotics and other seizure threshold lowering medicinal products (such as bupropion, mirtazapine, tetrahydrocannabinol) to cause convulsions.
- Concomitant therapeutic use of tramadol and serotonergic drugs, such as selective serotonin reuptake inhibitors (SSRIs), serotonin-norepinephrine reuptake inhibitors (SNRIs), MAO inhibitors, tricyclic antidepressants and mirtazapine may cause serotonin toxicity. Signs of serotonin syndrome may be for example confusion, agitation, fever, sweating, ataxia, hyperreflexia, myoclonus and diarrhoea.
- Caution should be exercised during concomitant treatment with tramadol and coumarin derivatives (e.g. warfarin) due to reports of increased INR with major bleeding and ecchymosis in some patients.
- Other active substances known to inhibit CYP3A4, such as ketoconazole and erythromycin, might inhibit the metabolism of tramadol (N-demethylation) probably also the metabolism of the active O-demethylated metabolite.
- Tramadol crosses the placenta. There is inadequate evidence available on the safety of tramadol in human pregnancy. Therefore tramadol should not be used in pregnant women. Tramadol is not recommended during breast-feeding. After a single administration of tramadol it is not usually necessary to interrupt breast-feeding.

Adverse reactions

- Frequent (1/10 to 1/100)
    - Nausea and dizziness
    - Headache, somnolence
- Common (1/100 to 1/1000)
    - Changes in appetite, paraesthesia, tremor, respiratory depression, epileptiform convulsions, involuntary muscle contractions, abnormal coordination, syncope
    - Palpitation, tachycardia, postural hypotension or cardiovascular collapse) uncommon
    - Retching; gastrointestinal irritation (a feeling of pressure in the stomach, bloating), diarrhoea
    - Vomiting, constipation, dry mouth
    - Sweating
    - Fatigue
- Rare (1/1000 to 1/10000)
    - Bradycardia and increase in blood pressure rare.
    - Hallucinations, confusion, sleep disturbance, anxiety and nightmares.
    - Changes in mood (usually elation, occasionally dysphoria), changes in activity (usually suppression, occasionally increase) and changes in cognitive and sensorial capacity (e.g. decision behaviour, perception disorders).
    - Blurred vision
    - Dyspnoea
    - Dermal reactions (e.g. pruritus, rash, urticaria)
    - Micturition disorders (difficulty in passing urine, dysuria and urinary retention)

For peer review only - http://bmjopen.bmj.com/site/about/guidelines.xhtml

- Motor weakness
- Allergic reactions (e.g. dyspnoea, bronchospasm, wheezing, angioneurotic oedema) and anaphylaxis

- Very rare (<1/10000)
  - Deranged liver enzymes (isolated cases).
- Symptoms of overdose are typical of other opioid analgesics and include miosis, vomiting, cardiovascular collapse, sedation and coma, seizures and respiratory depression. Supportive measures such as maintaining the patency of the airway and maintaining cardiovascular function should be instituted; naloxone should be used to reverse respiratory depression; fits can be controlled with diazepam. In case of intoxication orally, gastrointestinal decontamination with activated charcoal or by gastric lavage is only recommended within 2 hours after tramadol intake. Gastrointestinal decontamination at a later time point may be useful in case of intoxication with exceptionally large quantities.
- Symptoms of withdrawal reactions, similar to those occurring during opiate withdrawal, may occur as follows: agitation, anxiety, nervousness, insomnia, hyperkinesia, tremor and gastrointestinal symptoms. Other symptoms that have very rarely been seen with tramadol discontinuation include: panic attacks, severe anxiety, hallucinations, paraesthesia, tinnitus and unusual CNS symptoms.

For peer review only - http://bmjopen.bmj.com/site/about/guidelines.xhtml

Health Research Authority

Anakinra vs. Steroids for the Treatment of Gout Attacks in Renal Patients (ASGARD): A Feasibility Study

EudraCT no. 2015-001787-19

9 PHARMACOVIGILANCE

9.1 Definitions

The Medicines for Human Use (Clinical Trials) Regulations 2004 (UK), as amended, provides the following definitions relating to adverse events in trials with an investigational medicinal product (table above).

Southend University Hospital will undertake all sponsor duties and pharmacovigilance. The Investigator is responsible for ensuring that all adverse events occurring during the study treatment period and 8 weeks after the first administration of study drugs (Anakinra and intramuscular methylprednisolone), must be recorded on the Adverse Event Case Report Form (AE CRF).

Term	Definition
**Adverse Event (AE)**	Any untoward medical occurrence in a participant to whom a medicinal product has been administered, including occurrences which are not necessarily caused by or related to that product.
**Adverse Reaction (AR)**	An untoward and unintended response in a participant to an investigational medicinal product which is related to any dose administered to that participant.<br><br>The phrase "response to an investigational medicinal product" means that a causal relationship between a trial medication and an AE is at least a reasonable possibility, i.e. the relationship cannot be ruled out.<br><br>All cases judged by either the reporting medically qualified professional or the Sponsor as having a reasonable suspected causal relationship to the trial medication qualify as adverse reactions.
**Serious Adverse Event (SAE)**	A serious adverse event is any untoward medical occurrence that:<br>• results in death<br>• is life-threatening<br>• requires inpatient hospitalisation or prolongation of existing hospitalisation<br>• results in persistent or significant disability/incapacity<br>• consists of a congenital anomaly or birth defect<br><br>Other 'important medical events' may also be considered serious if they jeopardise the participant or require an intervention to prevent one of the above consequences.<br><br>NOTE: The term "life-threatening" in the definition of "serious" refers to an event in which the participant was at risk of death at the time of

For peer review only - http://bmjopen.bmj.com/site/about/guidelines.xhtml

	the event; it does not refer to an event which hypothetically might have caused death if it were more severe.
**Serious Adverse Reaction (SAR)**	An adverse event that is both serious and, in the opinion of the reporting Investigator, believed with reasonable probability to be due to one of the trial treatments, based on the information provided.
**Suspected Unexpected Serious Adverse Reaction (SUSAR)**	A serious adverse reaction, the nature and severity of which is not consistent with the information about the medicinal product in question set out:<br>• in the case of a product with a marketing authorisation, in the summary of product characteristics (SmPC) for that product<br>• in the case of any other investigational medicinal product, in the investigator's brochure (IB) relating to the trial in question

NB: to avoid confusion or misunderstanding of the difference between the terms "serious" and "severe", the following note of clarification is provided: "Severe" is often used to describe intensity of a specific event, which may be of relatively minor medical significance. "Seriousness" is the regulatory definition supplied above.

9.2 Operational definitions for (S) AEs

9.2.1 Investigator Assessment

At each contact with the subject during the treatment period, the investigator must seek information on adverse events by specific questioning and, as appropriate, by examination. Information elicited should be recorded immediately in the source document and the AE CRF. All clearly related signs, symptoms, and abnormal diagnostic procedures should be recorded using the NCI CTCAE v4.0 event terms and grading. The clinical course of each event should be followed until resolution or stabilisation.

The use of Anakinra is in this trial is outside of the licensed indication. Hence, all AR are required to be reported and certain AEs will need to be reported. Previous AEs reported are neutropenia and infection. Other previously described adverse reactions include signs at the injection site such as erythema, pruritus and rash. Adverse reactions in the control arm to be examined for included high blood sugars/uncontrolled diabetes and clinical signs of fluid retention.

Investigators will make assessments on:

- Seriousness – the CI/PI responsible for the care of the patients, or in his absence an authorised medic with the research team, is responsible for assessing whether the event is serious according to the definitions given section 9.1.
- Causality – The investigator must assess causality of all serious adverse events/reactions in relation to the trial treatment according to the definition given. If the SAE is assessed as having a reasonable causal relationship, then it is defined as a SAR.

For peer review only - http://bmjopen.bmj.com/site/about/guidelines.xhtml

- Expectedness – The investigator must assess the expectedness of all SARs according to the definition given. If the SAR is unexpected, then it is a SUSAR.
- Severity –The intensity of an event should not be confused with the term “serious” which is a regulatory definition based on patient/event outcome criteria. The investigator must assess the severity of the vent according to the following terms and assessments.
    - Mild: Some discomfort noted but without disruption of daily life
    - Moderate: Discomfort enough to affect/reduce normal activity
    - Severe: Complete inability to perform daily activities and lead a normal life

9.2.2 Pre-existing Conditions

Pre-existing conditions should not be reported as AE unless the condition worsens by at least CTCAE grade during trial. The condition must be reported in the pre-treatment section of the CRF, if symptomatic at the time of entry, or under concurrent medical conditions if asymptomatic.

9.2.3 Exceptions to (S) AE or (S) AR reporting

Many clinical events are likely to occur given the co-morbid burden of the participant group. Events that are recognised and expected complications of the condition are exempt from normal reporting procedure, unless they are of an unexpected severity. These would not be considered to be SUSARs unless the severity of the event was considered to be unexpected. Expected adverse events in this patient consist of worsening:

- Complications of chronic kidney disease
- Heart failure
- Hypertension
- Diabetes complications
- Underlying cardiac disease with complications such as ACS, arrhythmia, hypotension, tachycardia and worsening oedema.

Other exceptions to (S) AE or (S) AR reporting consist of:

- Routine treatment or monitoring of the studied indication not associated with any deterioration in condition.
- Associated with any deterioration in condition, e.g. pre-planned hip replacement operation which does not lead to further complications.
- Any admission to hospital or other institution for general care where there was no deterioration in condition.
- Treatment on an emergency, outpatient basis for an event **not** fulfilling any of the definitions of serious as given above and not resulting in hospital admission.
- Any death or hospitalisation due to fall or fracture
- Any death or hospitalisation due to exacerbation of an existing medical co-morbid condition

In all cases AEs and / or laboratory abnormalities that are critical to the safety evaluation of the participant must be reported to the Sponsor; these may be volunteered by the participant, discovered by the investigator questioning or detected through physical examination, laboratory test or other investigation. Where certain AEs are not required to be reported to the Sponsor, these should still be recorded in the participant’s medical records.

9.2.4 Recording and reporting of AE or AR

For peer review only - http://bmjopen.bmj.com/site/about/guidelines.xhtml

The IMP reference documentation that is used for pharmacovigilance purposes is used to assess the causality and expectedness of events and will be checked by the Sponsor for changes on the anniversary of the CTA.

Reporting of AEs / SAEs start at the point of consent. Reporting for ARs / SARs and SUSARs start at first IMP dose. If the AE or AR is not defined as serious, it is recorded in the study file and the participant is followed up by the research team. The AE/AR is documented in the participants' medical notes (where appropriate) and AE CRF. **All reactions should be reported and will be reviewed by the sponsor/CI as this is a CTIMP trial using licensed drug outside of the licensed indication even though some class evidence is available.**

9.3 Recording and reporting of SAEs AND SUSARs

All **SAEs* / SUSARs** occurring from the time of **start of trial treatment** until 7 days post cessation of trial treatment must be recorded on the SAE form/AECRF Form and faxed to the Sponsor **within 24 hours** of the research staff becoming aware of the event. Once all resulting queries have been resolved, the Sponsor will request the original form should also be posted to the Sponsor and a copy to be retained on site.

A completed SAE/SUSAR Report Form for all events should be faxed, or scanned and e-mailed, to:

Dr G Balasubramaniam, Southend University Hospital.

E-mail: g.balasubramaniam@southend.nhs.uk

Tel: 07843563739 (mobile); 01702 435555 (work switchboard)

Fax: 01702 508595

E-mail should also be sent to arctu@anglia.ac.uk to initiate notification and follow-up monitoring.

For each **SAEs* / SUSARs*** the following information will be collected:

- full details in medical terms and case description
- event duration (start and end dates, if applicable)
- action taken
- outcome
- seriousness criteria
- causality (i.e. relatedness to trial drug / investigation), in the opinion of the investigator
- Whether the event would be considered expected or unexpected.

Any change of condition or other follow-up information should be faxed to the Sponsor as soon as it is available or at least within 24 hours of the information becoming available. Events will be followed up until the event has resolved or a final outcome has been reached.

All SAEs assigned by the PI or delegate (or following central review) as both suspected to be related to IMP-treatment and unexpected will be classified as SUSARs and will be subject to expedited reporting to the Medicines and Healthcare Products Regulatory Agency (MHRA). The

For peer review only - http://bmjopen.bmj.com/site/about/guidelines.xhtml

Sponsor will inform the MHRA, the REC and the Sponsor of SUSARs within the required expedited reporting timescales.

9.3.1 Recording and reporting of SAE

All SAEs will be recorded in the participants' notes, the CRF, the sponsor AECRF form and reported to the sponsor/ARCTU/IMP provider within 24 hours of CI or PI or co-investigators becoming aware of the event (unless SAE/SAR/SUSAR is specified as not requiring immediate reporting). Nominated co-investigators will be authorised to sign the SAE forms in the absence of the CI at the co-ordinating site or the PI at the participating sites.

A complete SAE form for all events requiring immediate reporting should be faxed, or scanned and e-mailed, to SUH. These reports should be followed by further detailed SAE/SUSAR reports until resolution of event.

9.3.2 Follow-up and Post-Study SAEs

The active monitoring period starts from the first dose of the IMP to two days after the last dose of the IMP i.e. 7 days. This is based on a half-life of 4 hours. The reporting requirement for SAEs affecting subjects applies for all events occurring up to 8 weeks after the last administration of study drugs, which coincides with last follow-up. All unresolved adverse events should be followed up by the Principle Investigator until the events are resolved.

Where a participant withdraws consent for further processing of data, this does not preclude the reporting of SARs and SUSARs which are required to continue being reported according to the protocol for regulatory purposes. The PIS should include a section explaining this to the participant.

9.3.3 Recording and reporting of SUSAR

All SUSARs that occur during the trial will be reported to the sponsor/ main REC/IMP provider/ARCTU (if applicable) within 24 hours of the CI or co-investigator becoming aware of the event. SUSARs should be reported to the sponsor/ARCTU within 24 hours as the sponsor has a legal obligation to report this to the MHRA within 7 days (for fatal or life-threatening SUSARs) or 15 days for all other SUSARs. In the case of multicentre studies, the PI or the co-investigators at the participating site must inform the CI within 24 hours of the event. The CI or co-investigators at the co-ordinating site must inform the sponsor/ARCTU immediately to allow reporting to the MHRA within the allocated timelines. The CI will need to complete the CIOMS form in conjunction with the sponsor SAE form to be sent to the MHRA by the sponsor. If warranted, an investigator alert may be issued, to inform all investigators involved in any study with the same drug (or therapy) that this serious adverse event has been reported.

The original and any subsequent follow up of Serious Adverse Event Forms and CIOMS forms (where applicable), together with the fax confirmation sheet must be kept with the TMF at the study site.

9.4 Responsibilities

- **Principal Investigator (PI)**

  Checking for AEs and ARs when participants attend for treatment / follow-up.

For peer review only - http://bmjopen.bmj.com/site/about/guidelines.xhtml

Health Research Authority

1. Using medical judgement in assigning seriousness, causality and expectedness [in Phase III and late Phase II CTIMPs] using the Reference Safety Information approved for the trial.
2. Using medical judgement in assigning seriousness and causality and providing an opinion on expectedness [in Phase I and early Phase II CTIMPs] using the Reference Safety Information approved for the trial.
3. Ensuring that all SAEs and SARs (including SUSARs) are recorded and reported to the Sponsor within 24 hours of becoming aware of the event and provide further follow-up information as soon as available. Ensuring that SAEs and SARs (including SUSARs) are chased with Sponsor if a record of receipt is not received within 2 working days of initial reporting.
4. Ensuring that AEs and ARs are recorded and reported to the Sponsor in line with the requirements of the protocol.

- **Chief Investigator (CI) / delegate or independent clinical reviewer**

1. Clinical oversight of the safety of patients participating in the trial, including an ongoing review of the risk / benefit.
2. Using medical judgement in assigning seriousness, causality and expectedness of SAEs where it has not been possible to obtain local medical assessment.
3. Using medical judgement in assigning expectedness [in Phase I and early Phase II CTIMPs].
4. Immediate review of all SUSARs.
5. Review of specific SAEs and SARs in accordance with the trial risk assessment and protocol as detailed in the Trial Monitoring Plan.
6. Assigning Medical Dictionary for Regulatory Activities (MedDRA) or Body System coding to all SAEs and SARs.
7. Preparing the clinical sections and final sign off of the Development Safety Update Report (DSUR).

- **Sponsor**

1. Central data collection and verification of AEs, ARs, SAEs, SARs and SUSARs according to the trial protocol onto a MACRO database.
2. Reporting safety information to the CI, delegate or independent clinical reviewer for the ongoing assessment of the risk / benefit according to the Trial Monitoring Plan.
3. Reporting safety information to the independent oversight committees identified for the trial (Data Monitoring Committee (DMC) and / or Trial Steering Committee (TSC)) according to the Trial Monitoring Plan.
4. Expedited reporting of SUSARs to the Competent Authority (MHRA in UK) and REC within required timelines.
5. Notifying Investigators of SUSARs that occur within the trial.
6. The un-blinding of a participant for the purpose of expedited SUSAR reporting [For double blind trials only].
7. Checking for (annually) and notifying PIs of updates to the Reference Safety Information for the trial.
8. Preparing standard tables and other relevant information for the DSUR in collaboration with the CI and ensuring timely submission to the MHRA and REC.

- **Trial Steering Committee (TSC)**

For peer review only - http://bmjopen.bmj.com/site/about/guidelines.xhtml

Health Research Authority

In accordance with the Trial Terms of Reference for the TSC, periodically reviewing safety data and liaising with the sponsor regarding safety issues. No DMC will be formed for this study as it is a small feasibility study, but it will be considered for the subsequent larger study.

9.5 Notification of deaths

Only deaths that are assessed to be caused by the IMP will be reported to the sponsor. This report will be immediate. All deaths, including deaths deemed unrelated to the IMP, if they occur earlier than expected will be reported to the sponsor. This report will be immediate.

9.6 Pregnancy reporting

- All pregnancies within the trial (either the trial participant or the participant's partner) should be reported to the Chief Investigator and the Sponsor using the relevant Pregnancy Reporting Form within 24 hours of notification
- Pregnancy is not considered an AE unless a negative or consequential outcome is recorded for the mother or child/foetus. If the outcome meets the serious criteria, this would be considered an SAE.
- Immediate reporting to the sponsor is required (within one working day of the PI/CI becoming aware of the event) using an ARCTU Pregnancy template form.
- If a subject or his/her partner becomes pregnant whilst taking part in the trial or during a stage where the foetus could have been exposed to an IMP. The CI/PI has the responsibility to ensure that the pregnancy form is completed and sent to the sponsor within the agreed timelines. The patient can continue on the study.
- The Investigator must ensure that the subject and the subject's healthcare professional are aware that follow-up information is required on the outcome of the pregnancy. If the subject leaves the area, their new healthcare professional should also be informed.
- The PI/CI also must follow up the pregnancy until delivery as well as monitoring the development of the new-born for the appropriate time after birth. Any events that occur during this time that could be considered to be a SAE must be reported to the sponsor in line with safety reporting, utilising the sponsor SAE reporting form.

9.7 Overdose

- No dose limiting toxicity has been observed during clinical trials. Patients have received my higher doses in the setting of sepsis and there was no difference in the adverse event profile.
- Dosing will be monitored as part of trial monitoring and any extra doses will be documented in the eCRF. Patients will still continue the regime as prescribed and outcome will be included in the final analysis.
- If an SAE is felt to be associated with the overdose will be fully described in the AECRF/SAE report form.

9.8 Reporting urgent safety measures

The CI may take urgent safety measures to ensure the safety and protection of the clinical trial participants from any immediate hazard to their health and safety, in accordance with Regulation 30. The measures should be taken immediately. In this instance, the approval of the Licensing Authority

For peer review only - http://bmjopen.bmj.com/site/about/guidelines.xhtml

Health Research Authority

Approval prior to implementing these safety measures is not required. However, it is the responsibility of the CI to inform the sponsor, Main Research Ethics Committee (via telephone) and the MHRA (via telephone for discussion with the medical assessor at the clinical trials unit) of this event immediately.

The CI has an obligation to inform both the MHRA and Main Ethics Committee in writing within 3 days, in the form of a substantial amendment. The sponsor must be sent a copy of the correspondence with regards to this matter.

9.9 The type and duration of the follow-up of subjects after adverse events.

All adverse events will need complete follow-up and reporting back to the ARCTU/CI/Sponsor. This will occur until the investigator feels the event or reaction has fully resolved.

8 weeks after consent and last follow-up, the Investigator should instruct each subject to report any subsequent event(s) that the subject, or the subject's general practitioner, believes might reasonably be related to participation in this study. The Investigator should notify the study sponsor of any death or adverse event occurring at any time after a subject has discontinued or terminated study participation that may be reasonably related to this study. The investigator should update the AECRF form with outcomes of all reported events.

Any SUSAR related to the IMP will need to be reported to the Sponsor irrespective of how long after IMP administration the reaction has occurred.

9.10 Development safety update reports

The Annual Safety Reports (ASR) will be sent by the CI to the sponsor, the MREC and MHRA (the date of the anniversary is the date on the "notice of acceptance letter" from the MHRA) using the ASR form. The CI will carry out a risk benefit analysis of the IMPs encompassing all events having arisen on the trial.

The CI/ARCTU will send the Annual Progress Report to the main REC using the REC template (the anniversary date is the date on the MREC "favourable opinion" letter from the MREC) and to the sponsor.

For peer review only - http://bmjopen.bmj.com/site/about/guidelines.xhtml

10 STATISTICS AND DATA ANALYSIS

10.1 Sample size calculation

This is a feasibility study and is not statistically powered to detect a difference. We will aim to recruit at least 16 patients in each arm of the study. The sample size of 32 was obtained from a recommendation based on the feasibility of running a parallel group design. The study will aim to enrol for 15 months. Our hospital had an average of 47 patients per year over the last four years with approximately 50% of patients having chronic kidney disease (i.e. 23 patients per year).

10.2 Planned recruitment rate

Enrolment is for 15 months from 6 centres, we will aim for one patient per month from larger centres and 1 every two months from smaller centres. If we assume a recruitment rate of xxx, we should meet out accrual rates. We will only recruit patients in working hours.

10.3 Statistical analysis plan

As this is a feasibility study the sample size will be too small for estimates to have adequate precision, and some methods will not be technically possible for some patterns of missing values. However, the best use will be made of the data that arises. The primary outcome measures are the time to 50% reduction and complete resolution of pain according to the visual analogue scale score and this will be used to compare the two groups in a time-to-event ("survival") analysis. Other time-to-event outcome measures will be analysed in a similar way. Outcome measures that are continuous, or can be taken to have an underlying continuity, such as physician assessment of joint tenderness and swelling, will be used to compare the two groups in a repeated measures analysis of variance with permutation tests. Two-sample comparisons of means will be by the two-sample permutation test. The association between the intervention and categorical variables will be tested using Fisher's exact test.

For peer review only

For peer review only - http://bmjopen.bmj.com/site/about/guidelines.xhtml

Anakinra vs. Steroids for the Treatment of Gout Attacks in Renal Patients (ASGARD): A Feasibility Study

EudraCT no. 2015-001787-19

10.3.1 Summary of baseline data and flow of patients

CONSORT 2010 Flow Diagram for reporting with transparency

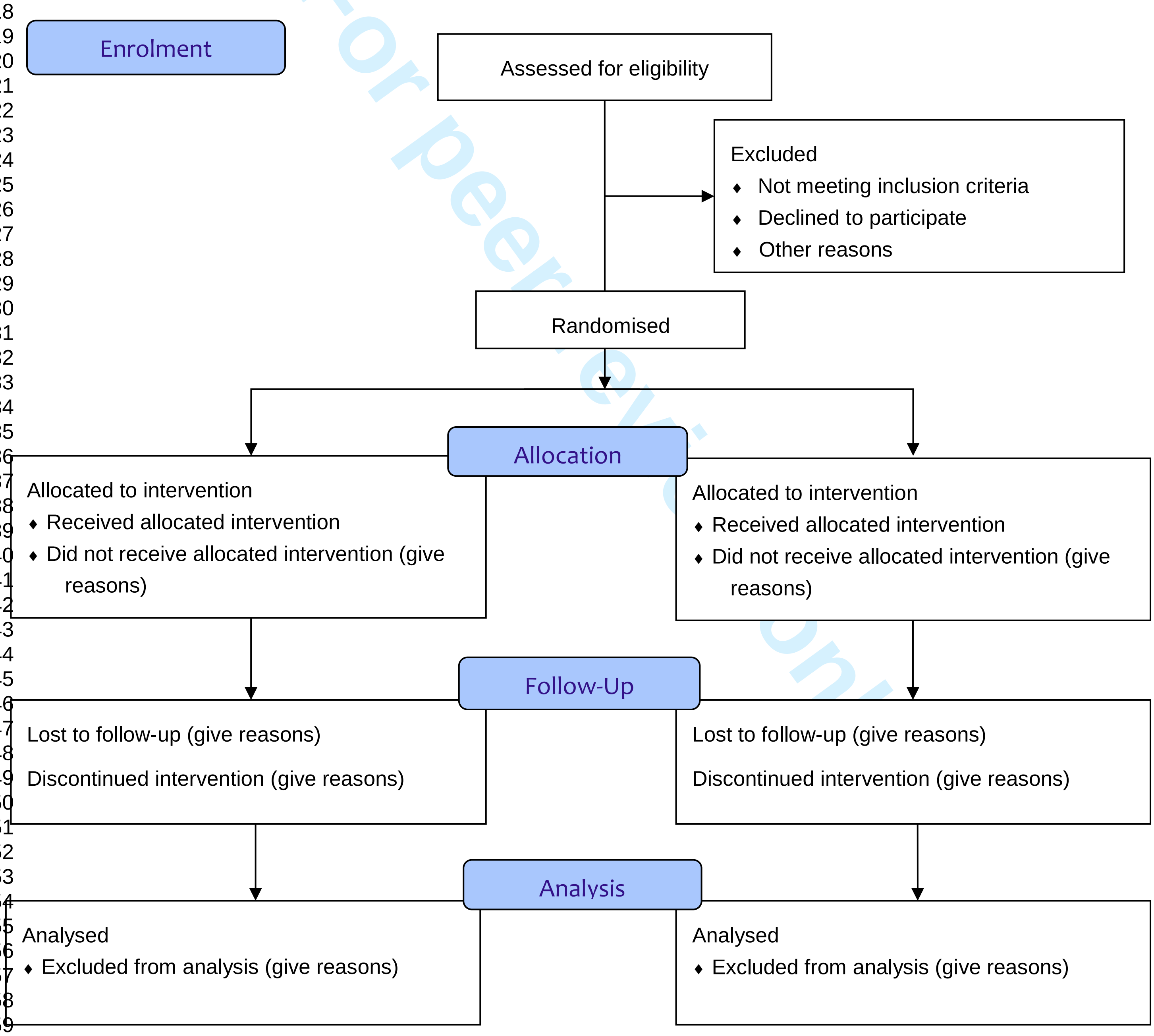

For peer review only - http://bmjopen.bmj.com/site/about/guidelines.xhtml

Health Research Authority

Anakinra vs. Steroids for the Treatment of Gout Attacks in Renal Patients (ASGARD): A Feasibility Study

EudraCT no. 2015-001787-19

For peer review only

For peer review only - http://bmjopen.bmj.com/site/about/guidelines.xhtml

10.3.2 Primary outcome analysis

- Study feasibility

  Study process will be analysed. Recruitment and retention rates, and willingness of patients to be randomised will be calculated. Proportion of patients who did not meet eligibility criteria will be looked at. Adherence and compliance rates, and qualitative feedback will be examined. Economic data on healthcare resource use and health related quality of life will be collected to inform the design of the subsequent larger study.

- Safety outcome measures with reporting as per CTIMP protocol (MHRA guidelines).

10.3.3 Secondary outcome analysis

- Proposed primary outcome measures of efficacy:
  - Time to 50% reduction and complete resolution of pain in self-assessed pain intensity in the joint most affected at baseline measure on the VAS (0-100mm) and 4-point Likert scale from baseline to 7 days post randomisation (Day 1 to Day 7)
- Patient reported outcome measure (Day 2 to Day 7)
- Physician assessment of joint tenderness and swelling (Day 1, Day 2, and Day 7)
- Time to take rescue medication
- Measurement in renal function by U+E, eGFR, CRP and urine uric acid
- Healthcare resource use.
- Patient assessment of activity limitation and quality of life: LEFS (Day 1, Day2, Day 7); EQ-5D-5L (Day 1, Day 2, Day 7 and 8 weeks); SF-36 and HAQ-DI (Day 1, Day 7 and 8 weeks).
- Changes in blood and urine inflammatory markers (Day 1, Day 2 Day 7 and 8 week).

10.4 Subgroup analyses

Not applicable

10.5 Adjusted analysis

Not applicable

10.6 Interim analysis and criteria for the premature termination of the trial

There will be no formal interim analysis. The steering committee will meet at pre-specified intervals. Patients will remain blinded, even if any adaptations are made. Analysis will be undertaken at end of study. The integrity of the trial will be protected by regular site visits and conferences (telephone, online and face-to-face meetings) with PIs.

The authority to stop or modify the trial lies with the Chief Investigator, trial steering committee, or sponsor. Although there is no formal data monitoring committee due to the small size of the study, some of the responsibilities of a DMA will be undertaken by the trial management committee as there is a pharmacovigilance aspect to this study.

10.7 Subject population

Data from all randomised population will be subjected to the study analysis, regardless of whether they received study drug and protocol adherence. This will be an intention to treat analysis.

For peer review only - http://bmjopen.bmj.com/site/about/guidelines.xhtml

Health Research Authority

Anakinra vs. Steroids for the Treatment of Gout Attacks in Renal Patients (ASGARD): A Feasibility Study

EudraCT no. 2015-001787-19

10.8 Procedure(s) to account for missing or spurious data

Processes in the protocol to minimise missing or spurious data includes reviews of patients at various time points, including at end of study.

The primary analysis is a time-to-event ("survival") analysis which accommodates missing data in its treatment of loss to follow-up. The repeated measures analysis of variance will be carried out as a linear mixed model using an algorithm that does not require balance and can accommodate missing data. For example this can be using the package lme4 for the computer program R. In this way missing values will not result in patients being dropped from an analysis. The serious issue of a possible differential dropout from the two groups will be examined carefully.

10.9 Other statistical considerations.

Not applicable

10.11 Economic evaluation

As this is a feasibility study, it is not designed or powered to show differences in effectiveness between groups and hence is unlikely to show differences in cost-effectiveness. For this reason a formal economic evaluation looking at the incremental cost-effectiveness of Anakinra compared to the comparator is not planned. Instead we will collect relevant data on resource use (costs) and health-related quality of life to inform subsequent large scale study. We will record all resources required to provide the drug treatment in both groups. This will include costs of drugs and administration and any required tests and contacts with health care practitioners. We will also record health care contacts by means of a patient administered questionnaire at the 8-week follow-up. Where possible we will also record the use of health care from the patient's notes and other sources. This will be used to access the validity of patient completed data and will inform future research design. Data collected will identify key drivers of cost in this group of individuals and will allow us to access the likely cost of providing the two drug treatments.

The performance of the EQ-5D-5L will be assessed in this group of patients. We will also calculate the SF-6D from the SF-36 questionnaire and compare the performance of this measure with that of the EQ-5D-5L.

For peer review only - http://bmjopen.bmj.com/site/about/guidelines.xhtml

11 DATA HANDLING

11.1 Data collection tools and source document identification

Source Data

The participants' clinical notes and anywhere that data is first recorded will act as source data. This will be used to obtain information for past medical history, subsequent medical conditions, hospital admissions, data on resource use, diagnostic reports and blood and urine tests. This will also include entries from study questionnaires (LEFS, EQ-5D-5L, SF-36 and HAQ-DI) and patient diaries (PROMS, VAS, and Likert scale). Entry will be made into eCRF forms and be printed out and stored at site with patient notes to form part of source data.

Source Documents

Source documents consist of:

- Case histories in participants' clinical notes, including copies of original records and signed copies of consent form.
- Pharmacy documentation of dispensed treatment
- Original documents, data and records including charts, subject's diaries, study questionnaires, subject's recordings, pharmacy records, and records kept in clinical laboratories etc.

Case report forms

Data will be collected and retained in accordance with the Data Protection Act (1988). Electronic case report forms (eCRFs) will be used via an online system (MACRO) to collect data. A validated data collection tool appropriate to the study will be used. The investigator is responsible for ensuring the accuracy, completeness, legibility and timeliness of the data reported in the CRFs.

Forms can be remotely accessed through an internet portal and study data will be entered and captured there. Data will be anonymised and patients will only be identifiable by a study number. Any inconsistencies, validation errors or inaccuracies will be reported to the CI regularly. One data collection is complete and the data has been validated, a data lock will be performed and analysis will be initiated. Only data required by the protocol will be captured in the eCRF.

CRFs as Source Documents

Data can be entered directly onto the eCRF (MACRO) by site research team, and if this is done then this would then be considered a source document. If the CRF is then transmitted to the sponsor, it is necessary for the trial site to retain a copy to ensure that the principal investigator can provide access to the source documents to a monitor, auditor, or regulatory agency.

Copied CRFs as Source Documents

Photocopies or print-outs of the CRFs may be used as source documents. Copies will be made onto coloured paper so they are easy to find in the case history and will not be mistaken for CRFs. The photocopied case report forms will indicate who collected the data (sign/initial and date) as well as the subject to whom the data applies.

For peer review only - http://bmjopen.bmj.com/site/about/guidelines.xhtml

Health Research Authority

Anakinra vs. Steroids for the Treatment of Gout Attacks in Renal Patients (ASGARD): A Feasibility Study

EudraCT no. 2015-001787-19

11.2 Data handling and record keeping

The Investigator and the study team will ensure that the Patient's identity is protected at every stage of their participation within the study. Each patient will be allocated a unique screening number by either the PI or a member of the study team before undergoing any screening procedures, this information will be kept on a screening log. Once the patient has completed screening procedures and is enrolled onto the study, the patient will be allocated a randomisation number determined by the randomisation system within the ARCTU. The data will be coded and depersonalised where the participant's identifying information is replaced by an unrelated sequence of characters. The patients initials (the first letter of their first name and the first letter of their last name) may be used as a means of pseudo-anonymising parameters. It will be securely maintained with the linking code in separate locations using encrypted digital files within password protected folders and storage media.

There will be limited access to the minimum number of individuals necessary for quality control, audit, and analysis. Identifiable data will be stored within the framework of the Caldicott principles. All stored data at the trial site, clinical trial unit and the main trial centre will be subject to review by a responsible individual from the clinical trial unit for monitoring and audit purposes. Completeness of data will be ensured by members of the research team contacting patients to ensure that allocated medication has been administered and that the main primary outcome data, patient reported pain score has been done..

The eCRF for data entry utilises MACRO. The eCRF will be developed, managed and maintained by ARCTU. Access to the eCRF will be restricted, with only authorised site-specific personnel able to make entries or amendments to their participants' data. It is the PI's/CI's responsibility to ensure completion and to review and approve all data captured in the eCRF. The PI or recruiting physician or research team will be responsible for all entries into the eCRF and will confirm (electronically) that the data are accurate and complete.

The investigator /institutions will keep records of all participating patients (sufficient information to link records e.g., CRFs, hospital records and samples), all original signed informed consent forms and copies of the CRF pages.

If any patient information needs to be sent to a third party (including correspondence/communication to central laboratories, the PI and the study team will adhere to patient pseudo-anonymous parameters. This includes the patient initials, date of birth, gender as well as the unique study ID/randomisation number. Any information that is to be collected by these third parties will utilise these coded details for any relevant documents as well as maintaining databases.

11.3 Data storage

Personal data and contact information / direct identifiers will be stored in the trial site file at the study site under the custodianship of the principle investigator for up to 15 years with participant consent. This will be available to the research / clinical team providing direct care for the patient. This will be stored with secure access in a locked cabinet. Contact information is required as the study requires daily contact with participants to ensure adherence to treatment and undertaking of assessments for data completeness. Patients will be asked for consent to keep records to contact them on a later date for update on the results and outcome of the study, and inform of further studies they could be involved with.

For peer review only - http://bmjopen.bmj.com/site/about/guidelines.xhtml

Health Research Authority

Coded data will be stored in the pharmacy of the study centre / hospital to monitor treatment allocation. A key to coded data will be held by the local pharmacy. This information will be stored in a locked cabinet with access only available to a nominated pharmacy representative identified in the pharmacy protocol.

Linked anonymised data or pseudonymised data will be entered directly using a secure web-based portal by the trial site team and stored in the research database at the Anglia Ruskin Clinical Trials Unit. Data will be stored in a locked cabinet / room with secure access. This data will be collected until the end of the study after which analysis will be undertaken by Mike Parker, Anglia Ruskin University Clinical Trials Unit statistician, to produce a report of the findings. Health economic data that will be link anonymised will be transferred under custodianship of David Turner, Norwich Medical School, University of East Anglia.

Linked anonymised data including on samples obtained will also be stored in a database with secure access only to the chief investigator and stored in a locked cabinet at the main trial site, Southend University Hospital, where samples will be stored. This will be under the custodianship of the chief investigator, Gowrie Balasubramaniam. Patients will be consented explicitly for this. Consent will be explicitly sought to keep records for 15 years with a view to use this data for future, as yet unspecified, medical research into health. Illness and medical treatment.

There is a qualitative aspect to the study that was suggested by the funding body that will require for participants to be interviewed with regards to the experience of participation. 12 out of the 32 patients will be randomly selected for qualitative assessment of participation experience. Consent will be explicitly obtained for audio interviews to be recorded. Pseudonymised data will be used for recording purposes and data obtained will be stored in a locked cabinet with secure access only to Dr Jon Scales, University of Essex.

11.4 Access to Data

Direct access will be granted to authorised representatives from the Sponsor, host institution and the regulatory authorities to permit trial-related monitoring, audits and inspections.

11.5 Archiving

During the course of research, all records are the responsibility of the Chief Investigator and will be kept in secure conditions. When the research trial is complete, records will be kept for a further 15 years as required by the Research Governance Framework and Trust Policy that the time.

For trials involving ____ Trust patients, undertaken by

_____________ staff, the approved repository for long-term storage of local records is

______________________________________ which is based at ____________________________.

Archiving will be authorised by the Sponsor following submission of the end of study report. All essential documents will be archived for a minimum of 5 years after completion of trial. Destruction of essential documents will require authorisation from the Sponsor.

The trial database will be securely stored at Anglia Ruskin Clinical Trials Unit under the ownership of the Chief Investigator.

For peer review only - http://bmjopen.bmj.com/site/about/guidelines.xhtml

Health Research Authority

Anakinra vs. Steroids for the Treatment of Gout Attacks in Renal Patients (ASGARD): A Feasibility Study

EudraCT no. 2015-001787-19

For peer review only

For peer review only - http://bmjopen.bmj.com/site/about/guidelines.xhtml

Health Research Authority

12 MONITORING, AUDIT & INSPECTION

Trial monitoring will be co-ordinated by Anglia Ruskin Clinical Trials Unit.

The purpose of monitoring is to verify that:

- The rights and well-being of the participants are protected
- Reported trial data is accurate, complete and verifiable from source documents
- The conduct of the trial is in compliance with the currently approved protocol/amendment(s), GCP and the applicable regulatory requirements.
- Part of the quality control of clinical trial and ensures ongoing quality and rigour of the study.

A Trial Monitoring Plan will be developed and agreed by the Trial Management Group (TMG) and TSC based on the trial risk assessment which may include on site monitoring

After the first site initiation visit, subsequent visits will be planned by the TMG, this will be based on the on-going assessment by the TMG and reviewing any problems that may arise from the trial data set. Monitoring might be initially conducted across all sites, and subsequently conducted using a risk based approach that focuses, for example, on sites that have the highest enrolment rates, large numbers of withdrawals, or atypical (low or high) numbers of reported adverse events.

The monitoring procedures will be outlined in a standard operating procedure to ensure uniformity and is thorough.

The processes reviewed will examine participant enrolment, consent, eligibility, and allocation to trial groups; adherence to trial interventions and policies to protect participants, including reporting of harm and completeness, accuracy, and timeliness of data collection

A member of the study team from the site will co-ordinate site visits / assessments

The site study team member will ensure:

- All required reports, notifications, applications and submissions are available and that these documents are accurate, complete, timely, legible, dated and identify the trial.
- All documents and trial supplies needed to conduct the trial properly, and to comply with the applicable regulatory requirements, are available.
- The most current up to date reference documents are available, and that all documents are available for review.
- All personnel part of the study team have adequate training, resources and facilities, including laboratories, equipment and staff, to safely and properly conduct the trial and the these remain adequate throughout the study period.
- Trial functions are not delegated to unauthorised individuals.
- Source documents and other trial records are accurate, complete and up-to-date, check the accuracy and completeness of the eCRF entries.
- Any data required by the protocol is reported accurately in the eCRF and is consistent with the source documents
- Any treatment modification, adverse events, concomitant medications and inter-current illness is reported in accordance with the protocol in the eCRFs.
- Any visits that the participant fails to make, tests not conducted and examinations not performed are reported clearly in the eCRFs.

For peer review only - http://bmjopen.bmj.com/site/about/guidelines.xhtml

Health Research Authority

Anakinra vs. Steroids for the Treatment of Gout Attacks in Renal Patients (ASGARD): A Feasibility Study

EudraCT no. 2015-001787-19

- All withdrawals and dropouts of enrolled subjects from the project are reported and explained on the eCRF
- the monitoring team are informed of any eCRF entry error, omission, or illegibility and ensure that appropriate corrections, additions or delegations are made, dated and explained (if necessary) and initialled by the Principle Investigator or an authorised individual. The monitor will not make such changes.
- Verification and collection of participant data should be performed according to data protection laws and requirements.
- All SAEs are appropriately reported within the time periods required by GCP, the protocol, the REC, clinical trial units and the applicable regulatory requirements.

With regards to the IMPs

- Ensure storage times and conditions are acceptable and that supplies are sufficient
- IMP is only supplied to subjects who are eligible, at the protocol specified dose
- Participants are provided with necessary instruction on properly using, handling, storing and returning IMPS
- The receipt, use and return of any IMP9s) at the trial sites are controlled and documented adequately.
- Disposal If unused IMP(s) complies with applicable regulatory requirement(s) and is in accordance with the sponsors of the SOP.
- Ensure that any code breaks are properly handled and documented according to the protocol and/or relevant code break SOP.
- Ensure that communication is in place in the event of any suspicion of scientific misconduct, fraud, or breach of GCP.

For peer review only - http://bmjopen.bmj.com/site/about/guidelines.xhtml

13 ETHICAL AND REGULATORY CONSIDERATIONS

13.1 Research Ethics Committee (REC) review & reports

Approval will be sought from a REC for the trial protocol, informed consent forms and other relevant documents e.g. advertisements and GP information letter. Substantial amendments that require review by REC will not be implemented until the REC grants a favourable opinion for the study. All correspondence with the REC will be retained in the Trial Master File/Investigator Site File. An annual progress report (APR) will be submitted to the REC within 30 days of the anniversary date on which the favourable opinion was given, and annually until the trial is declared ended.

It will be the Chief Investigator's responsibility to produce the annual reports as required. The Chief Investigator will notify the REC of the end of the study if the study is ended prematurely, the Chief Investigator will notify the REC, including the reasons for the premature termination.

The Chief Investigator will submit a final report with the results, including any publications/abstracts, to the REC within one year after the end of the study.

13.2 Peer review

This trial protocol was reviewed by the funder (NIHR) and internal Trust departments. It is also reviewed by the Trust Research and Development Committee.

13.3 Public and Patient Involvement

There has been public and patient involvement since the conception of this study. They have been involved in the design, and management of the research. There are members of the steering committee that are members of the patient and public group. They will help with the dissemination of the study.

13.4 Regulatory Compliance

The trial will not commence until a Clinical Trial Authorisation (CTA) is obtained from the MHRA and the protocol and trial conduct will comply with the Medicines for Human Use (Clinical Trials) Regulations 2004 and any relevant amendments.

13.5 Protocol compliance

Prospective, planned deviations or waivers to the protocol are not allowed under the UK regulations on Clinical Trials and must not be used e.g. it is not acceptable to enrol a subject if they do not meet the eligibility criteria or restrictions specified in the trial protocol. Accidental protocol deviations can happen at any time and must be adequately documented on the relevant forms and reported to the Chief Investigator and Sponsor immediately. Deviations from the protocol which are found to frequently recur are not acceptable, will require immediate action and could potentially be classified as a serious breach.

13.6 Notification of Serious Breaches to GCP and/or the protocol

A "serious breach" is a breach which is likely to effect to a significant degree –

(a) the safety or physical or mental integrity of the subjects of the trial; or
(b) the scientific value of the trial

For peer review only - http://bmjopen.bmj.com/site/about/guidelines.xhtml

Health Research Authority

Anakinra vs. Steroids for the Treatment of Gout Attacks in Renal Patients (ASGARD): A Feasibility Study

EudraCT no. 2015-001787-19

The sponsor will be notified immediately of any case where the above definition applies during the trial conduct phase. The sponsor of a clinical trial will notify the licensing authority in writing of any serious breach of:

(a) the conditions and principles of GCP in connection with that trial; or
(b) the protocol relating to that trial, as amended from time to time, within 7 days of becoming aware of that breach

13.7 Data protection and patient confidentiality

All investigators and trial site staff must comply with the requirements of the Data Protection Act 1998 with regards to the collection, storage, processing and disclosure of personal information and will uphold the Act's core principles.

The Investigator has a responsibility to ensure that patient anonymity is protected and maintained. They must also ensure that their identities are protected from any unauthorised parties. Information with regards to study patients will be kept confidential and managed in accordance with the Data Protection Act, NHS Caldicott Guardian, The Research Governance Framework for Health and Social Care and Research Ethics Committee Approval.

Data will be stored for five years before being destroyed. The Chief Investigator in the data custodian.

13.8 Financial and other competing interests for the chief investigator, PIs at each site and committee members for the overall trial management

The drug and equivalent placebo is provided by the distributor, Swedish Orphan Biovitrium (SOBI). There is a payment of £10,000 to help with additional study costs.

13.9 Indemnity

This is UK NHS-sponsored study. For NHS sponsored research HSG (96)48 reference no 2. Refers. If there is negligent harm during the clinical trial when the NHS body owes a duty of care to the person harmed, NHS indemnity covers UK NHS staff, medical academic staff with honorary contracts conducting the trial. NHS indemnity does not offer no-fault compensation and is unable to agree in advance to pay compensation for non-negligent harm. Ex-gratia payments may be considered in the case of claim. Principle investigators and their medical staff should seek indemnity against non-negligent harm for this study from their NHS trust.

13.10 Amendments

Changes made to the research after review body approval are amendments. These may be substantial or non-substantial. Amendments will be tracked in the protocol appendix and the version of the protocol will be updated.

13.10.1 Non-substantial Amendments

The sponsor may make a non-substantial amendment at any time during a trial under the Medicines for Human Use (Clinical Trials) Regulations 2004. Non-substantial amendments will be made by using the Notification of Non-Substantial/Minor Amendment(s) for NHS Studies form from the HRA website. The form will be filled by the CI ad authorised by the sponsor.

Examples of Non-substantial Amendments

For peer review only - http://bmjopen.bmj.com/site/about/guidelines.xhtml

- minor changes to the protocol or other study documentation, e.g. correcting errors, updating contact points, minor clarifications;
- updates of the investigator's brochure (unless there is a change to the risk/benefit assessment for the trial);
- changes to the chief investigator's research team changes to the research team at particular trial sites (other than appointment of a new principal investigator in a CTIMP);
- changes in funding arrangements; changes in the documentation used by the research team for recording study data;
- changes in the logistical arrangements for storing or transporting samples; inclusion of new sites and investigators in studies other than CTIMPs;
- extension of the study beyond the period specified in the application form

13.10.2 Substantial Amendments

If the sponsor wishes to make a substantial amendment to the CTA or the documents that supported the original application for the CTA, the sponsor must submit a valid notice of amendment to the licencing authority (MHRA) for consideration. If the sponsor wishes to make a substantial amendment to the REC application or the supporting documents, the sponsor must submit a valid notice of amendment to the REC for consideration. The MHRA and/or the REC will provide a response regarding the amendment within 35 days of receipt of the notice. It is the sponsor's responsibility to decide whether an amendment is substantial or non-substantial for the purposes of submission to the MHRA and/or REC.

If applicable, other specialist review bodies (e.g. CAG) need to be notified about substantial amendments in case the amendment affects their opinion of the study.

Amendments also need to be notified to NHS R&D departments of participating sites to assess whether the amendment affects the NHS permission for that site. Note that some amendments that may be considered to be non-substantial for the purposes of REC and/or MHRA may still need to be notified to NHS R&D (e.g. a change to the funding arrangements). For studies with English sites processed in NIHR CSP the amendment should be submitted in IRAS to the lead CRN, which will determine whether the amendment requires notification to English sites or may be implemented immediately (subject to REC/MHRA approval were necessary).

Examples of Substantial amendments

- changes to the design or methodology of the study, or to background information affecting its scientific value;
- changes to the procedures undertaken by participants;
- any change relating to the safety or physical or mental integrity of participants, or to the risk/benefit assessment for the study;
- significant changes to study documentation such as participant information sheets, consent forms, questionnaires, letters of invitation, letters to GPs or other clinicians, information sheets for relatives or carers;
- a change of sponsor(s) or sponsor's legal representative; appointment of a new chief investigator a change to the insurance or indemnity arrangements for the study;

For peer review only - http://bmjopen.bmj.com/site/about/guidelines.xhtml

- inclusion of a new trial site (not listed in the original application) in a CTIMP; appointment of a new principal investigator at a trial site in a CTIMP;
- temporary halt of a study to protect participants from harm, and the planned restart of a study following a temporary halt; a change to the definition of the end of the study;
- any other significant change to the protocol or the terms of the REC application

13.11 Post trial care

The treatments are for an acute flares of gout and participants will not need to be on the treatments for a long period of time. It is beyond the scope of this trial to look at the use of long-term gout prophylaxis. This is an area where evidence in people with kidney disease is lacking and we are collecting data at the 8-week point (last time point) to see if the various healthcare providers treating participants use prophylactic treatments on participants.

Use of Anakinra is not licensed for acute attacks of gout but has been used worldwide with evidence increasing building up. Continued access to this intervention need not be considered and it is available for off-label use by healthcare professionals. Intramuscular methylprednisolone is readily available and used widely.

13.12 Access to the final trial dataset

The final dataset will be available to the statistician for data analysis. Components of health economic will be made available to the health economist for economic analysis. The chief investigator and members of the steering group will have access to the full dataset. This will ensure that results are not disclosed prior to publication. Site investigators will not have access to full dataset.

For peer review only - http://bmjopen.bmj.com/site/about/guidelines.xhtml

Health Research Authority

14 DISSEMINIATION POLICY

14.1 Dissemination policy

The trial report will be used for publications and presentation at scientific meetings. Investigators have the right to publish orally or in writing the results of the study. On completion of the trial, the data will be analysed and tabulated and a Final Study Report prepared. Summaries of results will be made available to investigators for dissemination and feedback to participants.

Patient groups will also be sent summaries as their involvement with the subsequent larger study will be pertinent. Local primary care networks will also be informed.

The data will be owned by the trial steering committee, primarily by the chief investigator. Funding bodies will be declared in publications. The full report will be made available directly from the chief investigator or from published material. Supplemental material linked with publication will consist of the trial protocol.

14.2 Authorship eligibility guidelines and any intended use of professional writers

The author of the final report will be the chief investigator. Named authors of manuscripts for publications will be in accordance with the International Committee of Medical Journal Editors has defined authorship criteria for manuscripts submitted for publication.

For peer review only - http://bmjopen.bmj.com/site/about/guidelines.xhtml

Health Research Authority

Anakinra vs. Steroids for the Treatment of Gout Attacks in Renal Patients (ASGARD): A Feasibility Study

EudraCT no. 2015-001787-19

15 REFERENCES

1) Fraser SD, Roderick PJ, Aitken G et al. Chronic kidney disease, albuminuria and socioeconomic status in the Health Surveys for England 2009 and 2010. J Public Health (Oxf). 2013 Nov 25.

2) Juraschek SP, Kovell LC, Miller ER 3rd, Gelber AC. Association of kidney disease with prevalent gout in the United States in 1988-1994 and 2007-2010. Semin Arthritis Rheum. 2013 Jun;42(6):551-61.

3) Annemans L, Spaepen E, Gaskin M, Bonnemaire M, Malier V, Gilbert T, Nuki G. Gout in the UK and Germany: prevalence, comorbidities and management in general practice 2000-2005. Ann Rheum Dis. 2008 Jul;67(7):960-6.

4) Keenan RT, O'Brien WR, Lee KH et al. Prevalence of contraindications and prescription of pharmacologic therapies for gout. Am J Med. 2011 Feb;124(2):155-63.

5) Curiel RV, Guzman NJ. Challenges associated with the management of gouty arthritis in patients with chronic kidney disease: a systematic review. Semin Arthritis Rheum. 2012 Oct;42(2):166-78.

6) Rothenbacher D, Primatesta P, Ferreira A, Cea-Soriano L, Rodríguez LA. Frequency and risk factors of gout flares in a large population-based cohort of incident gout. Rheumatology (Oxford). 2011 May;50(5):973-81.

7) Jordan KM, Cameron JS, Snaith M et al. British Society for Rheumatology and British Health Professionals in Rheumatology guideline for the management of gout. British Society for Rheumatology and British Health Professionals in Rheumatology Standards, Guidelines and Audit Working Group (SGAWG). Rheumatology (Oxford). 2007 Aug;46(8):1372-4.

8) Zhang W, Doherty M, Pascual E et al. EULAR evidence based recommendations for gout. Part I: Diagnosis. Report of a task force of the Standing Committee for International Clinical Studies Including Therapeutics(ESCISIT). EULAR Standing Committee for International Clinical Studies Including Therapeutics. Ann Rheum Dis. 2006 Oct;65(10):1301-11.

9) Khanna D, Khanna PP, Fitzgerald JD et al 2012. American College of Rheumatology guidelines for management of gout. Part 2: therapy and anti-inflammatory prophylaxis of acute gouty arthritis. American College of Rheumatology. Arthritis Care Res (Hoboken). 2012 Oct;64(10):1447-61.

10) Siegel LB, Alloway JA, Nashel DJ. Comparison of adrenocorticotropic hormone and triamcinolone acetonide in the treatment of acute gouty arthritis. J Rheumatol. 1994 Jul;21(7):1325-7.

11) Terkeltaub RA, Furst DE, Bennett K, Kook KA, Crockett RS, Davis MW. High versus low dosing of oral colchicine for early acute gout flare: Twenty four-hour outcome of the first multicenter, randomized, double-blind, placebocontrolled, parallel-group, dose-comparison colchicine study. Arthritis Rheum. 2010 Apr;62(4):1060-8.

12) Janssens HJ, Janssen M, van de Lisdonk EH, van Riel PL, van Weel C. Use of oral prednisolone or naproxen for the treatment of gout arthritis: a double-blind, randomised equivalence trial. Lancet. 2008 May 31;371(9627):1854-60.

13) Pyne D, Ioannou Y, Mootoo R, Bhanji A. Intra-articular steroids in knee osteoarthritis: a comparative study of triamcinolone hexacetonide and methylprednisolone acetate. Clin Rheumatol. 2004 Apr;23(2):116-20. Epub 2004 Feb 24.

14) Corkill MM, Kirkham BW, Chikanza IC, Gibson T, Panayi GS. Intramuscular depot methylprednisolone induction of chrysotherapy in rheumatoid arthritis: a 24-week randomized controlled trial. Br J Rheumatol. 1990 Aug;29(4):274-9.

15) Choy EH, Kingsley GH, Khoshaba B, Pipitone N, Scott DL. A two year randomised controlled trial of intramuscular depot steroids in patients with established rheumatoid arthritis who have shown an incomplete response to disease modifying antirheumatic drugs. Intramuscular Methylprednisolone Study Group. Ann Rheum Dis. 2005 Sep;64(9):1288-93.

16) Dinarello CA. Interleukin-1 in the pathogenesis and treatment of inflammatory diseases. Blood. 2011 Apr 7;117(14):3720-32.

For peer review only - http://bmjopen.bmj.com/site/about/guidelines.xhtml

17) So A, De Smedt T, Revaz S, Tschopp J. A pilot study of IL-1 inhibition by anakinra in acute gout. Arthritis Res Ther. 2007;9(2):R28.
18) Chen K, Fields T, Mancuso CA, Bass AR, Vasanth L. Anakinra's efficacy is variable in refractory gout: report of ten cases. Semin Arthritis Rheum. 2010 Dec;40(3):210-4.
19) Ghosh P, Cho M, Rawat G et al. Treatment of acute gouty arthritis in complex hospitalized patients with anakinra.Arthritis Care Res (Hoboken). 2013 Aug;65(8):1381-4.
20) Ottaviani S, Moltó A, Ea HK et al. Efficacy of anakinra in gouty arthritis: a retrospective study of 40 cases. Arthritis Res Ther. 2013;15(5):R123.
21) Thueringer JT, Doll NK, Gertner E. Anakinra for the treatment of acute severe gout in critically ill patients. Semin Arthritis Rheum. 2015 Aug;45(1):81-5
22) Balasubramaniam G, Almond M, Dasgupta B. Improved renal function in diabetic patients with acute gout treated with anakinra. Kidney Int. 2015 Jul;88(1):195-6.
23) Direz G, Noël N, Guyot C, Toupance O, Salmon JH, Eschard JP. Efficacy but side effects of anakinra therapy for chronic refractory gout in a renal transplant recipient with preterminal chronic renal failure. Joint Bone Spine. 2012 Dec;79(6):631.
24) Nuki G, Bresnihan B, Bear MB et al. Long-term safety and maintenance of clinical improvement following treatment with anakinra (recombinant human interleukin-1 receptor antagonist) in patients with rheumatoid arthritis: extension phase of a randomized, double-blind, placebo controlled trial. Arthritis Rheum. 2002 Nov;46(11):2838-46.
25) Fleischmann RM, Schechtman J, Bennett R. Anakinra, a recombinant human interleukin-1 receptor antagonist (r-metHuIL-1ra), in patients with rheumatoid arthritis: A large, international, multicenter, placebo-controlled trial. Arthritis Rheum. 2003 Apr;48(4):927-34.
26) Schiff MH, DiVittorio G, Tesser J. The safety of anakinra in high-risk patients with active rheumatoid arthritis: six-month observations of patients with comorbid conditions. Arthritis Rheum. 2004 Jun;50(6):1752-60.
27) Fleischmann RM, Tesser J, Schiff MH. Safety of extended treatment with anakinra in patients with rheumatoid arthritis. J Rheumatol. 2006 Feb;33(2):234-43.
28) Larsen CM, Faulenbach M, Vaag A, Vølund A, Ehses JA, Seifert B, Mandrup-Poulsen T, Donath MY. Interleukin-1-receptor antagonist in type 2 diabetes mellitus. N Engl J Med. 2007 Apr 12;356(15):1517-26.
29) Abbate A, Kontos MC, Abouzaki NA et al. Comparative safety of interleukin-1 blockade with anakinra in patients with ST-segment elevation acute myocardial infarction (from the VCU-ART and VCU-ART2 pilot studies). Am J Cardiol. 2015 Feb 1;115(3):288-92.
30) Schlesinger N, Mysler E, Lin HY et al. Canakinumab reduces the risk of acute gouty arthritis flares during initiation of allopurinol treatment: results of a double-blind, randomised study. Ann Rheum Dis. 2011 Jul;70(7):1264-71.
31) Di Giovine FS, Malawista SE, Nuki G, Duff GW. Interleukin 1 (IL 1) as a mediator of crystal arthritis. Stimulation of T cell and synovial fibroblast mitogenesis by urate crystal-induced IL 1. J Immunol. 1987 May 15;138(10):3213-8.
32) Arthritis Advisory Committee Meeting 2011: FDA briefing committee report. http://www.fda.gov/downloads/AdvisoryCommittees/CommitteesMeetingMaterials/Drugs/Arthritis DrugsAdvisoryCommittee/UCM259596.pdf
33) Mitha E, Schumacher HR, Fouche L et al. Rilonacept for gout flare prevention during initiation of uric acid-lowering therapy: results from the PRESURGE-2 international, phase 3, randomized, placebo-controlled trial. Rheumatology (Oxford). 2013 Jul;52(7):1285-92.
34) Schumacher HR Jr, Evans RR, Saag KG et al. Rilonacept (interleukin-1 trap) for prevention of gout flares during initiation of uric acid-lowering therapy: results from a phase III randomized, double

For peer review only - http://bmjopen.bmj.com/site/about/guidelines.xhtml

Health Research Authority

Anakinra vs. Steroids for the Treatment of Gout Attacks in Renal Patients (ASGARD): A Feasibility Study

EudraCT no. 2015-001787-19

blind, placebo-controlled, confirmatory efficacy study. Arthritis Care Res (Hoboken). 2012 Oct;64(10):1462-70.
35) Terkeltaub RA, Schumacher HR, Carter JD at al. Rilonacept in the treatment of acute gouty arthritis: a randomized, controlled clinical trial using indomethacin as the active comparator. Arthritis Res Ther. 2013 Feb 1;15(1):R25.
36) So A, De Meulemeester M, Pikhlak A et al. Canakinumab for the treatment of acute flares in difficult-to-treat gouty arthritis: Results of a multicenter, phase II, dose-ranging study. Arthritis Rheum. 2010 Oct;62(10):3064-76.
37) Schlesinger N, De Meulemeester M, Pikhlak A et al. Canakinumab relieves symptoms of acute flares and improves health-related quality of life in patients with difficult-to-treat Gouty Arthritis by suppressing inflammation: results of a randomized, dose-ranging study. Arthritis Res Ther. 2011 Mar 25;13(2):R53.
38) Schlesinger N, Alten RE, Bardin T et al. Canakinumab for acute gouty arthritis in patients with limited treatment options: results from two randomised, multicentre, active-controlled, double blind trials and their initial extensions. Ann Rheum Dis. 2012 Nov;71(11):1839-48.
39) Hung AM, Ellis CD, Shintani A et al. IL-1β receptor antagonist reduces inflammation in hemodialysis patients.J Am Soc Nephrol. 2011 Mar;22(3):437-42.
40) Chakraborty A, Van LM, Skerjanec A. Pharmacokinetic and pharmacodynamic properties of canakinumab in patients with gouty arthritis. 2013 Dec;53(12):1240-51.
41) Bresnihan B, Alvaro-Gracia JM, Cobby M. Treatment of rheumatoid arthritis with recombinant human interleukin-1 receptor antagonist. Arthritis Rheum. 1998 Dec;41(12):2196-204.
42) Sicras-Mainar A, Navarro-Artieda R, Ibá˜nez-Noll J. Resource Use and Economic Impact of Patients With Gout: A Multicenter, Population-Wide Study. Reumatol Clin. 2013;9(2):94–10
43) Chandratre P, Roddy E, Clarson L, Richardson J, Hider SL, Mallen CD. Health-related quality of life in gout: a systematic review. Rheumatology (Oxford). 2013 Nov;52(11):2031-40. doi:0.1093/rheumatology/ket265.
44) Scire CA, Manara M, Cimmino MA et al. Gout impacts on function and health-related quality of life beyond associated risk factors and medical conditions: results from the KING observational study of the Italian Society for Rheumatology (SIR). Arthritis Res Ther. 2013 Aug 23;15(5):R101
45) Wu E, Patel P, Yu A et al. Disease Related and All-Cause Health Care Costs of Elderly Patients With Gout. Jour Manag Care Pharm 2008;14(2):164-75.
46) Khanna PP, Nuki G, Bardin T et al. Tophi and frequent gout flares are associated with impairments to quality of life, productivity, and increased healthcare resource use: Results from a cross-sectional survey. Health Qual Life Outcomes. 2012 Sep 22;10:117. doi: 10.1186/1477-7525-10-117.
47) Singh JA, Taylor WJ, Simon LS et al. Patient-reported outcomes in chronic gout: a report from OMERACT 10. J Rheumatol. 2011 Jul;38(7):1452-7.
48) Singh JA, Taylor WJ, Dalbeth N et al. OMERACT Endorsement of Measures of Outcome for Studies of Acute Gout. J Rheumatol. 2013 Dec 15.

For peer review only - http://bmjopen.bmj.com/site/about/guidelines.xhtml

16. APPENDICIES

16.1 Appendix 1-Risk

Risks associated with trial interventions

☐ LOW ≡ Comparable to the risk of standard medical care

× MODERATE ≡ Somewhat higher than the risk of standard medical care

☐ HIGH ≡ Markedly higher than the risk of standard medical care

Justification: Briefly justify the risk category selected and your conclusions below (where the table is completed in detail the detail need not be repeated, however a summary should be given):

There have been reports of increased risk of infections and low white cell counts in patients treated with similar agents. This may not apply with our CTIMP, Anakinra, which has good long-term safety data from use in other conditions like arthritis and septicaemia. It is a biologic and this puts it at higher risk than standard medical care.

There are also reports of injection site reactions, which would not occur with other treatments as Anakinra requires daily injections for the course of treatment.

What are the key risks related to therapeutic interventions you plan to monitor in this trial?		How will these risks be minimised?		
IMP/Intervention	Body system/Hazard	Activity	Frequency	Comments
Anakinra	Infection	Monitoring of patients and avoid recruiting patients with active infection	Throughout project, up to 8 weeks	Pharmacovigilance is an aspect of project
Anakinra	Low white cell count	Blood tests. Avoid recruiting patients with low white cell counts	Day 1, 2, 7, 8 wk	Can be reversed by stopping treatment due to its short duration of action.
Anakinra	Injection site reactions	Monitoring of injection site	Day 1, 2, 7	

For peer review only - http://bmjopen.bmj.com/site/about/guidelines.xhtml

Outline any other processes that have been put in place to mitigate risks to participant safety (e.g. DMC, independent data review, etc.)<br><br>There is a pharmacovigilance aspect to this trial which will monitor all adverse events. There is no formal DMC due to the small size of study but some of this responsibility will be undertaken by the TSC.

16.2 Appendix 2 - Study management / responsibilities

The CI will manage the Trial Management file with the Trail manager who is based at the Anglia Ruskin Clinical Trials Unit (ARCTU). The CI will also manage the investigator site file (ISF) for Southend University hospital. The CI and trial manager will set up ISF for all sites participating in this trial, this will be managed by the PIs at each of the participating sites. The ISF will contain the same sections as the TMF.

The ISF will be actively maintained by the site PI from its establishment until the trial is formally closed. The ISF and source documentation (printed eCRF) will be stored in a locked cabinet or a room in a secure area with access only authorised by study personnel.

16.2.1 Patient registration/randomisation procedure

Randomisation will be carried out by the Clinical Trials Data Manager within ARCTU. ARCTU uses the TENALEA randomisation service provided by the Trans European Network for Clinical Trial Services. This is an internet based randomisation system, which will be set up for the study by ARCTU in accordance with protocol. This will be a 1:1 block randomisation, which will only be possible if the patient meets inclusion and exclusion criteria and has consented to take part in the trial. The system stores the pre-determined sequence of randomisation; this list is visible to neither the investigator nor ARCTU. The research nurse or investigator will log onto the web browser application and enter the patient's eligibility and stratification factors into the system; the treatment allocation is then returned to the investigator and selected members of ARCTU and study team.

16.2.2 Data management

Project data collection will be managed by the Clinical Trials Unit Data Manager who will oversee recruitment and collection of data. The responsibility for data collection and entry rests with the research nurse at each respective site. ARCTU uses an online data management system called MACRO to design and manage electronic case report forms (eCRFs). ARCTU will work with Southend University Hospital study team to design and validate the data collection tools. Once a patient is enrolled, the research team can access these forms remotely through the Internet portal. All data will be in anonymised form; patients will be identifiable only by study number. Data will be remotely monitored by ARCTU and discussed at data monitoring committee meetings. Any inconsistencies, validation errors or inaccuracies will be reported to the lead investigator regularly. Data queries will be sent regularly to each study site to ensure data is accurate and being entered in a timely manner. Once data collection is complete and the data has been validated, a data lock will be performed and analysis can begin.

16.2.3 Preparation and submission of amendments

For peer review only

For peer review only - http://bmjopen.bmj.com/site/about/guidelines.xhtml

The CI along with the TSC will prepare and submit amendments. The overall process will be under the governance of the sponsor.

16.2.4 Preparation and submission of Annual Safety Report/Annual

The recruitment period is scheduled to last 15 months. The CI will prepare and submit an annual progress report to the main REC and to the sponsor as appropriate until the close of the study.

16.2.5 Data protection/confidentiality

All investigators and trial site staff must comply with the requirements of the Data Protection Act 1998 with regards to the collection, storage, processing and disclosure of personal information and will uphold the Act's core principles.

The Investigator has a responsibility to ensure that patient anonymity is protected and maintained. They must also ensure that their identities are protected from any unauthorised parties. Information with regards to study patients will be kept confidential and managed in accordance with the Data Protection Act, NHS Caldicott Guardian, The Research Governance Framework for Health and Social Care and Research Ethics Committee Approval.

Data will be stored for twenty years before being destroyed. The Chief Investigator in the data custodian.

16.2.6 Trial documentation and archiving

During the course of the research, all records are the responsibility of the Chief Investigator and will be kept in secure conditions at each designated site. When the research trial is complete, it is a requirement of the Research Governance Framework and Trust Policy that the records are kept for a further 20 years.

16.3 Appendix 3 – Authorisation of participating sites

16.3.1 Required documentation

- CVs of CI and site staff
- Protocol and any subsequent amendments
- Approved Participant Information Sheets
- Approved Consent Forms
- Indemnity documentation from sponsor
- Conditions of sponsorship from sponsor
- Final R&D Approval
- Signed site agreement
- Ethics submission and approval
- Laboratory accreditation letter, certification and normal ranges
- Delegation log
- Staff training log
- Site signature log
- Investigator's brochure
- Investigator site file

For peer review only - http://bmjopen.bmj.com/site/about/guidelines.xhtml

- Study drug accountability

16.3.2 Procedure for initiating/opening a new site

Site initiation will be managed by the trial coordinator at ARCTU and must be completed prior to any trial procedures taking place. The trial coordinator will arrange a site initiation visit, where the following information will be discussed:

- Investigator and study staff responsibilities
- Protocol review
- Investigator's brochure, including adverse events and serious adverse events
- Site recruitment action plan and recruitment expectations
- Informed consent procedures
- Source documentation and eCRF completion review
- Investigational product
- Lab procedure review
- Pharmacy procedure review
- Safety reporting
- Monitoring requirements
- Audits and Inspections
- Investigator site file

Once the trial coordinator has reviewed the necessary requirements related to the study and has received all required documentation, the site can begin trail procedures and the release of drug will be available for the participating site.

16.3.3 Principal Investigator responsibilities

The PI is responsible for managing the study and trial procedures carried out at the participating site. Prior to any study procedures taking place at the site, the PI is required to review the protocol and study documents and ensure that the site is set up and capable to handle trial procedures. The PI must attend the site initiation visit and training and participate in meetings and teleconferences related to the trial. The PI must confirm completion of training for site research staff, ensure that the ISF is accurately maintained, disseminate important safety or trial related information to the trial oversight committee and CI, and provide adequate safety reporting.

16.2 Appendix 2 - Study management / responsibilities

The CI will manage the Trial Management file with the Trail manager who is based at the Anglia Ruskin Clinical Trials Unit. The CI will also manage the investigator site file for Southend University hospital. The CI will set up ISF for all sites participating in this trial, this will be managed by the PIs at each of the participating sites. The ISF will contain the same sections as the TMF.

The ISF will be actively maintained by the site PI from its establishment until the trial is formally closed. The ISF and source documentation (printed eCRF) will be stored in a locked cabinet or a room in a secure are with access only authorised by study personnel.

For peer review only - http://bmjopen.bmj.com/site/about/guidelines.xhtml

16.4 Appendix 4 – Schedule of Procedures

SCHEDULE OF PROCEDURES

Procedures	Screening	Baseline (pre-treatment)<br>Day 1	Day 2	Day 3	Day 4	Day 5	Day 6	Day 7	8 weeks
		Treatment phase							
			Follow up						
Informed Consent	X								
Demographics	X								
Medical History	X								
Physical examination	X		X						
Vital signs	X		X						
Blood and urine tests	X	X	X					X	X
Concomitant medications	X		X	X	X	X	X	X	
Eligibility assessment	X								
Randomisation		X							
Dispensing of trial drug		X							
Drug administration		X	X	X	X	X			
Patient reported pain and outcome assessments (diary entry)		X	X	X	X	X	X	X	
Physician assessment of joint		X	X					X	

For peer review only

For peer review only - http://bmjopen.bmj.com/site/about/guidelines.xhtml

Anakinra vs. Steroids for the Treatment of Gout Attacks in Renal Patients (ASGARD): A Feasibility Study

EudraCT no. 2015-001787-19

Patient assessment of activity limitation and quality of life using:									
• LEFS		X	X					X	
• EQ-5D-5L		X	X					X	X
• HAQ-DI and SF-36		X						X	X
Compliance Assessments			X	X	X	X	X	X	
Checking patient diary entry for time of injection and pain assessment (by phone or face to face)			X	X	X	X	X	X	
Diary return								X	
Check injection sites		X	X					X	
Check returned syringes								X	
Questionnaire return (face to face or by mail)									X
Adverse events assessments (face to face or by phone)		X	X	X	X	X	X	X	X
Physician's withdrawal checklist									
Interview (12 patients)									X
Resource use data									X

For peer review only

For peer review only - http://bmjopen.bmj.com/site/about/guidelines.xhtml

Anakinra vs. Steroids for the Treatment of Gout Attacks in Renal Patients (ASGARD): A Feasibility Study

EudraCT no. 2015-001787-19

For peer review only

For peer review only - http://bmjopen.bmj.com/site/about/guidelines.xhtml

Health Research Authority

Anakinra vs. Steroids for the Treatment of Gout Attacks in Renal Patients (ASGARD): A Feasibility Study

EudraCT no. 2015-001787-19

16.5 Appendix 5 – Safety Reporting Flow Chart

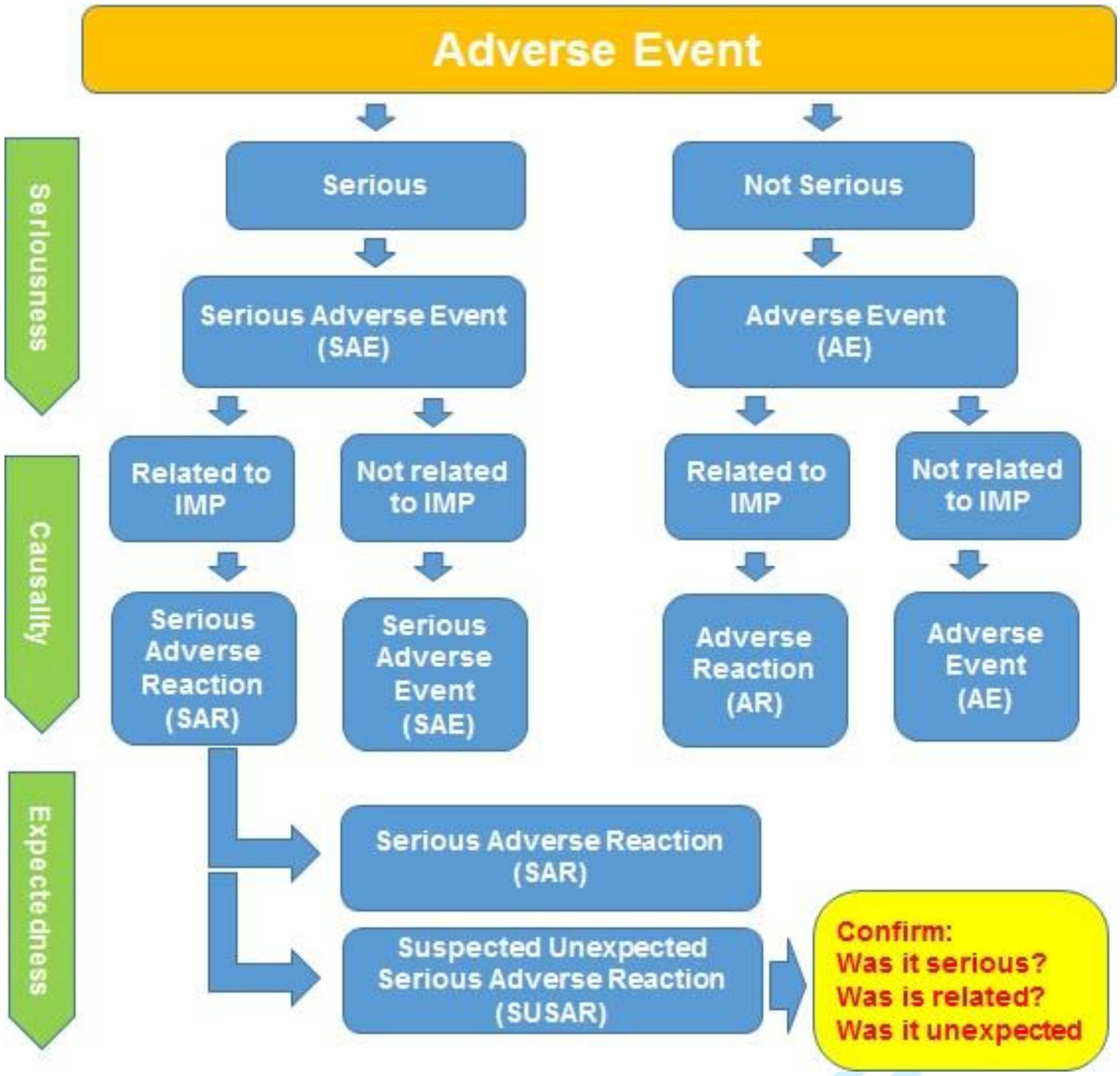

**Adverse Event (AE)**	Any untoward medical occurrence in a participant to whom a medicinal product has been administered, including occurrences which are not necessarily caused by or related to that product.
**Adverse Reaction (AR)**	An untoward and unintended response in a participant to an investigational medicinal product which is related to any dose administered to that participant.<br>The phrase "response to an investigational medicinal product" means that a causal relationship between a trial medication and an AE is at least a reasonable possibility, i.e. the relationship cannot be ruled out.<br>All cases judged by either the reporting medically qualified professional or the Sponsor as having a reasonable suspected causal relationship to the trial medication qualify as adverse reactions.
**Serious Adverse Event (SAE)**	A serious adverse event is any untoward medical occurrence that results in death; is life-threatening, requires inpatient; hospitalisation or prolongation of existing hospitalisation; results in persistent or significant disability/incapacity; or consists of a congenital anomaly or birth defect.<br>Other 'important medical events' may also be considered serious if they jeopardise the participant or require an intervention to prevent one of the above consequences.<br>NOTE: The term "life-threatening" in the definition of "serious" refers to an event in which the participant was at risk of death at the time of the event; it does not refer to an event which hypothetically might have caused death if it were more severe.
**Serious Adverse Reaction (SAR)**	An adverse event that is both serious and, in the opinion of the reporting Investigator, believed with reasonable probability to be due to one of the trial treatments, based on the information provided.

For peer review only - http://bmjopen.bmj.com/site/about/guidelines.xhtml

**Suspected Unexpected Serious Adverse Reaction (SUSAR)**	A serious adverse reaction, the nature and severity of which is not consistent with the information about the medicinal product in question set out in the case of a product with a marketing authorisation, in the summary of product characteristics (SmPC) for that product or in the case of any other investigational medicinal product, in the investigator's brochure (IB) relating to the trial in question.

16.6 Appendix 6 – Amendment History

Amendment No.	Protocol version no.	Date issued	Author(s) of changes	Details of changes made
1	1.1	17/11/15	G Balasubramaniam	Removal of day 4 assessment due to feedback from recruiting centres and outcome of TMG discussion. Week 8 visit for all patients to record health resource use and blood tests.
2	1.2	01/01/16	G Balasubramaniam	Exclusion criteria amended to be clear to exclude patients requiring intra-venous anti-biotic administration, or within two weeks of such treatment.<br><br>Abstinence from sex clarified as true abstinence i.e. non-periodic (calendar, ovulation, sympthothermal, post-ovulation methods)<br><br>Included a rationale for dose and dose duration for Anakinra (Kineret)
3	1.3	13/05/16	G Balasubramaniam	Trial pharmacist changed from Stuart Chandler to trial pharmacists: Amiirah Ropun, Inisa Idress and Laura Wilson.<br><br>Trial statistician changed from Michael Parker to Jufen Zhang.
4	1.3	01/07/16	G Balasubramaniam	Information in baseline assessment pertaining to new diagnostic gout criteria in 2015 (ACR and EULAR) taken in baseline assessment.

For peer review only - http://bmjopen.bmj.com/site/about/guidelines.xhtml

Health Research Authority

Anakinra vs. Steroids for the Treatment of Gout Attacks in Renal Patients (ASGARD): A Feasibility Study

EudraCT no. 2015-001787-19

5	1.3	01/07/16	G Balasubramaniam	Use of CKD EPI or MDRD (as per local guidelines) to calculate eGFR
6	1.3	01/07/16	G Balasubramaniam	Men of child bearing age not willing to undertake highly effective contraception during treatment period excluded
7	1.3	01/07/16	G Balasubramaniam	One further centre added Basildon and Thurrock University Hospital NHS Trust.
8	1.3	01/07/16	G Balasubramaniam	PI changed at Mid Essex NHS Trust
9	1.3	01/07/16	G Balasubramaniam	Participants can be contacted on Friday and Monday as a reminder for weekend or compliance check respectively.
10	1.3	01/07/16	G Balasubramaniam	Baseline assessments can be made after four hours as opposed to six hours if permitted form of analgesia used.
11	1.3	01/07/16	G Balasubramaniam	ARCTU email added in SAE / SUSAR section to help with monitoring.
12	1.4	01/02/17	G Balasubramaniam	Depo-Medrone changed to generic term of intramuscular methylprednisolone throughout protocol.
13	1.4	01/02/17	G Balasubramaniam	Exclusion criteria changed to specific criteria for each treatment i.e. Colchicine, Steroids and NSAIDS
14	1.4	01/02/17	G Balasubramaniam	Line in exclusion criteria 5, (7 in new version 1.4) “Con-current medication for RA like methotrexate and anti-TNF treatment has been associated with increased risk of neutropenia and infection” removed as repeats

For peer review only - http://bmjopen.bmj.com/site/about/guidelines.xhtml

Health Research Authority

Anakinra vs. Steroids for the Treatment of Gout Attacks in Renal Patients (ASGARD): A Feasibility Study

EudraCT no. 2015-001787-19

				exclusion criteria 4 (6 in version 1.4)
15	1.4	01/02/17	G Balasubramaniam	Exclusion criteria 13; potential participants who were given empirical treatment with antibiotics but infection has been excluded can be recruited.
16	1.4	01/02/17	G Balasubramaniam	Exclusion criteria 14; potential participants uncontrolled concomitant disease. The term “serious” has been removed and potential participation in the trial should be in agreement with the direct medical team.
17	1.4	01/02/17	G Balasubramaniam	Pre-screening for potential participants.
18	1.4	01/02/17	G Balasubramaniam	Use of present renal blood tests that may show an acute kidney injury will not be used to calculate baseline renal function.
19	1.5	13/03/2017	G Balasubramaniam	Trial Manager changed from Trisha Parker to Arit Udoh.
20	1.5	13/03/2017	G Balasubramaniam	Section 7.13 Rescue medication added. Rescue treatment is Prednisolone 35mg po orally, with additional low dose colchicine if needed.
21	1.5	13/03/2017	G Balasubramaniam	Typographical error with blood collection in page 41.

List details of all protocol amendments here whenever a new version of the protocol is produced.

Protocol amendments must be submitted to the Sponsor for approval prior to submission to the REC committee or MHRA.

For peer review only - http://bmjopen.bmj.com/site/about/guidelines.xhtml

NHS
Health Research Authority

Anakinra vs. Steroids for the Treatment of Gout Attacks in Renal Patients (ASGARD): A Feasibility Study

EudraCT no. 2015-001787-19

For peer review only

For peer review only - http://bmjopen.bmj.com/site/about/guidelines.xhtml

PARTICIPANT INFORMATION SHEET (VERSION 1.1)

Centre Number: Study Number:

ASGARD: THE STUDY OF ANAKINRA VERSUS STEROIDS FOR THE TREATMENT OF ACUTE GOUT ATTACKS IN PEOPLE WITH KIDNEY DISEASE

Invitation to participate in the above project

We would like to invite you to take part in our research project. Before you decide, we would like you to understand why the research is being done and what it would involve for you. One of our team will go through the information sheet with you and answer any questions you have. We'd suggest this should take about 30 minutes. Talk to others about the study if you wish and ask us if there is anything that is not clear.

Part 1 tells you the purpose of this study and what will happen to you if you take part. Part 2 gives you more details information about the conduct of the study.

Part 1

What is the purpose of the project?

Gout attacks affect approximately 1.3 million people with kidney disease in the UK. We do not know the safest and best way to treat an attack of gout in these patients. We want to compare the safest treatment currently available, steroids, with a new treatment that is being increasingly used called Anakinra. Anakinra stops the action of a chemical called interleukin-1, which plays an important role in the acute gout attack, and we think it might be a better alternative to steroid treatment. This project is looking at a small number of people and will provide information to plan a larger study. We hope the larger study will then go on to tell us which treatment may be better, safer and more cost-effective.

Why have I been invited?

You are being asked to participate in this clinical research project because you have acute gout and kidney disease.

Do I have to take part?

No, it is up to you to decide whether or not to join the project. We will describe the project and go through this information sheet. If you agree to take part, we will ask you to sign a consent form. You are free to withdraw at any time, without giving a reason. This would not affect the standard of care you receive.

What will happen to me if I take part?

If you agree to take part in the project, a member of the project team may undertake some additional blood tests (about two teaspoonful) to make sure it is suitable for you to be involved with the project. If this is ok, then you will be asked to fill a consent form to proceed. This project lasts for 8 weeks but most of your involvement will be for the first 7 days.

For peer review only - http://bmjopen.bmj.com/site/about/guidelines.xhtml

ASGARD

PARTICIPANT INFORMATION SHEET (VERSION 1.1)

Centre Number: **Study Number:**

This project compares two different treatments. We put people into two groups and give each group a different treatment. The results are then compared to see if one is better. To try and make sure the groups are the same to start with, each participant is put into a group by chance (randomly). The chance of being on a particular type of treatment in 50:50. This is a “double-blind trial,” so neither you nor your doctor will know in which treatment group you are in (although, if your doctor needs to find out he/she can do so).

We will undertake an assessment on the first day (Day 1) before you receive the treatment. This will take about an hour and consists of clinical questions and examination, blood tests and you will be asked to complete some questionnaires. You will also be issued a diary for you to assess your pain and you will be asked to complete some questionnaires to assess how the pain is affecting your ability to go about your day.

You will then be given the two injections, one in the buttock, and one under the skin in your tummy or thigh. The injection into your buttock would have been part of your usual care anyway.

You will also be issued a further 4 injections to give under the skin daily over the next 4 days. We will teach you or someone who lives with you to give these injections. If we cannot do this, we can organise a nurse to visit your home to do this. You will be given a small bin to dispose of your empty syringes safely and return after 7 days.

An outline of your visits and assessments following your enrolment is shown in the figure below.

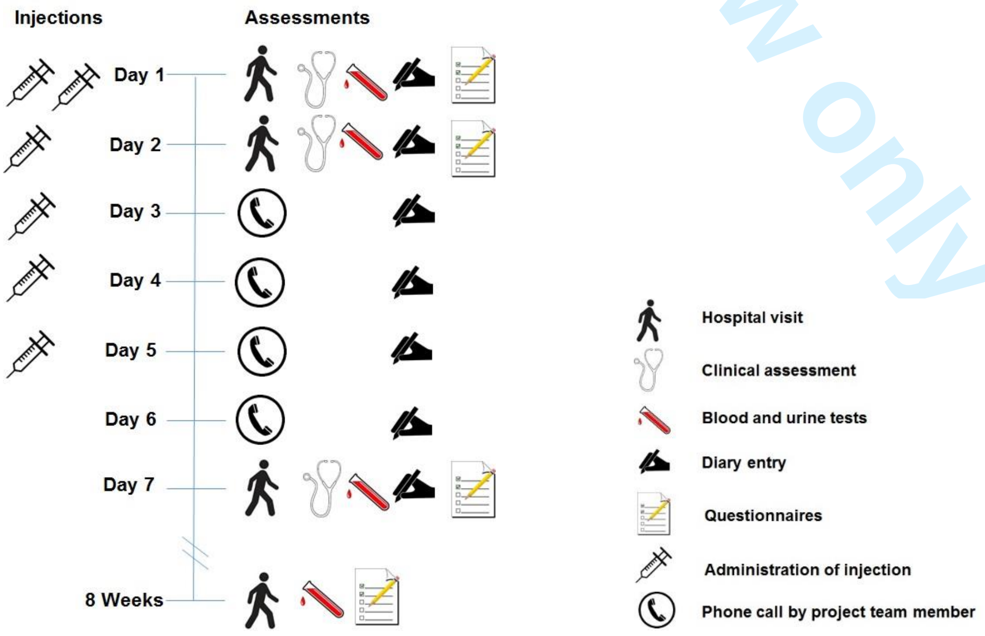

You will then have to attend hospital on the second and seventh day for about an hour. We will call you on the days you don’t attend hospital to check that you have had the treatment

For peer review only - http://bmjopen.bmj.com/site/about/guidelines.xhtml

PARTICIPANT INFORMATION SHEET (VERSION 1.1)

Centre Number: **Study Number:**

and filled your diary. There is a final visit and assessment after 8 weeks for blood tests and questionnaires, one additional questionnaire will look at all the NHS services you have used over the last eight weeks. You may also be asked to have an interview to give your feelings on being part of this project, you do not need to do this if you don't want to.

Your contact information will be stored at your local hospital so that we can contact you about the study, any results of the project and any future studies that we may plan.

You will have blood tests (about two tablespoons) and urine samples (a small cupful) on the second day, the seventh day and after eight weeks. Some of these samples will be tested straight away in the local NHS laboratory, while some will be processed, stored and analysed at the end of the study at Southend Hospital or specialised laboratories linked to the project. With your consent, a tiny sample of your blood plasma, serum, urine and DNA will be stored in a clinical laboratory at Southend University Hospital under the custodianship of Dr Gowrie Balasubramaniam (Chief Investigator).

Expenses and payments

You will not be paid for taking part in the study. We can pay for travel expenses on the days you need to attend hospital.

What will I have to do?

You will have to receive additional injections for five days and make assessments for the week. You will be issued with a diary to make the assessments, this should take less than five minutes a day. We will call you to make sure you have done this. You will also have to attend hospital for a clinical assessment and to fill some questionnaires on the second day, the seventh day and at eight weeks. This visit will probably take approximately forty-five minutes, with forty minutes for the questionnaires and five minutes for the assessment and blood tests.

A small number of participants (12) will be asked to undertake an interview session when you attend at eight weeks. This can take up to an additional hour. We will ask you about your experience of participating in the project and your answers will be recorded using an audio recording device. We will specifically choose participants to give a broad representation of people based on age and gender. The questions you will be asked will be related to your feelings on what you had to do as part of this project, we will also ask if you feel we could do things differently. This will help improve our future research. You can decide not to undertake this particular aspect of the project if you wish.

What are the alternatives for treatment?

Two other alternative treatments are available. NSAIDS are the best treatment for gout but they can be harmful in people with kidney disease. Colchicine is also a good treatment but can cause severe diarrhoea and other serious complications, as colchicine can build up in the body with kidney failure.

For peer review only - http://bmjopen.bmj.com/site/about/guidelines.xhtml

PARTICIPANT INFORMATION SHEET (VERSION 1.1)

Centre Number: **Study Number:**

What are the possible disadvantages, risks and side-effects?

Anakinra is a new treatment that works by reducing your immune reaction and there can be complications associated with this. In one study where Anakinra was used to help treat people with Rheumatoid Arthritis, there was a risk (<2%) of picking up an infection (like a chest infection) and a lowering of the white blood cell count. These participants were on other treatments that weakened their immune reaction and treatment with Anakinra lasted for many months. We do not anticipate these problems as we are not involving people on other treatments that affect the immune system and treatment is only for 5 days.

We will make sure that you don't have a serious infection and that your blood counts are normal before you are involved with the project. You will be monitored to make sure everything is going well and we will also check your blood counts.

You will be receiving injections under the skin for five days. Some people have had reactions where they inject their skin for many months but we do not feel this will be a problem for the five days you will receive the injection. We will have a look at the injection site when you visit the hospital.

We will ask that you refrain from using an additional or rescue pain killer for four hours prior to a pain assessment, this is to obtain a more accurate and fair comparison of the two treatments. You would normally have to wait for four hours in between doses of pain killers anyway, so you can minimise any discomfort by doing the assessment just before a dose. You do not have to do the assessments at the exact time every day but at roughly the same sort of time i.e. late morning or afternoon.

What are the possible benefits of taking part?

You will not be given a treatment that we already know may be harmful for you. You will be looked after by a specialist and you will be given contact details to use if there are any problems. If this study is successful then it would help develop clear guidelines to help doctors treat people with kidney disease and gout, something we know is currently not being done well.

There is also some suggestion that using this novel treatment may be beneficial for other conditions.

Will taking part in the study affect your usual care?

The only alteration to your treatment is that you will be asked to refrain from taking additional or rescue painkillers for four hours prior to doing an assessment of your joint. You would normally wait for four hours in between doses of painkillers and we would suggest that you do the assessment just before your next dose. We will not alter any of your other medications or interfere with your other treatments in any way. You will continue to be seen by your GP and by any hospital clinics that you usually attend.

For peer review only - http://bmjopen.bmj.com/site/about/guidelines.xhtml

ASGARD

PARTICIPANT INFORMATION SHEET (VERSION 1.1)

Centre Number: **Study Number:**

What happens when the research study stops?

The medication is only used as part of the study. Any future gout attacks you have will be treated as before. You will not know what exact treatment you have had as part of the study.

What if there is a problem?

Any complaint about the way you have been dealt with during the study or any possible harm you might suffer will be addressed. The detailed information on this is given in Part 2.

Will my taking part in this study be kept confidential?

Yes, we will follow ethical and legal practice and all the information about you will be handled in confidence. The details are included in part 2.

This completes Part 1 of the information sheet.

For peer review only - http://bmjopen.bmj.com/site/about/guidelines.xhtml

ASGARD

PARTICIPANT INFORMATION SHEET (VERSION 1.1)

Centre Number: **Study Number:**

Part 2 of the information sheet

If the information in Part 1 has interested you and you are considering participation please read the additional information in Part 2 before making any decision.

What if relevant new information becomes available?

Sometimes we get new information about the treatment being studied. If this happens your research doctor will tell you and discuss whether you should continue in the study. If you decide not to carry on, your research doctor will make arrangements for your care to continue. If you decide to continue in the study, he/she may ask you to sign an agreement outlining the discussion.

What will happen if I don't want to carry on with the study?

You can withdraw from treatment at any time but keep in contact with us to let us know your progress. Information collected may still be used. Any stored blood samples that can still be identified as yours can be destroyed if you so wish.

What if there is a problem?

If you have a concern about any aspect of this study, you should ask to speak to the researchers who will do their best to answer your questions. If you remain unhappy and wish

to complain formally, you can do this using the Complaints Officer for ___________________

hospital on: _______________. Details can be obtained from__________________________.

In the event something goes wrong and you are harmed during the project, and this is due to someone's negligence then you may have grounds for a legal action for compensation

against _____________________ hospital but you may have to pay your legal costs. The normal National Health Service complaints mechanisms will still be available to you.

Will my taking part in this study be kept confidential?

If you join the study, some parts of your medical records and the data collected for the study will be looked at by authorised persons from the organisers of the research. They may also be looked at by authorised people to check that the study is being carried out correctly. All will have a duty of confidentiality to you as the research participant and we will do our best to meet this duty.

Your research data will be stored using a unique study code which is non-identifiable, but can be linked back to your original records. All written information will be kept in a locked filing cabinet in a locked room. Any web-based data will be stored in a secure password protected central database at the Anglia Ruskin Clinical Trials Unit. Some of the data will be

For peer review only - http://bmjopen.bmj.com/site/about/guidelines.xhtml

PARTICIPANT INFORMATION SHEET (VERSION 1.1)

Centre Number: **Study Number:**

analysed at the University of East Anglia and the University of Essex. Only individuals directly involved with the study will have access to this information. All data will be under the custodianship of Dr Gowrie Balasubramaniam. Information may be retained for future studies but this will be after the appropriate ethical approvals. It will be retained for fifteen years and be disposed of securely after this period.

Will my GP know about this project?

We will inform your GP of your participation with your permission and we will also inform him/her of any clinical results, and of any new medical problems we might find as a result of your participation in the project.

What will happen to any samples I give?

Blood and urine samples taken for this study will be processed at the local centre and transferred for storage to Southend University Hospital. The samples with be labelled with a unique study code which will not identify you, but can be linked back to your original records. The samples can be stored for up to fifteen years and some will be sent to other laboratories both in the UK and within the European Union for further analysis. They may be used for future research that cannot yet be specified but this will be done after the appropriate ethical approvals. Samples will be under the custodianship of Dr Gowrie Balasubramaniam.

Will any genetic tests be done?

We will test for changes in the structure of the DNA. There will be **no** DNA genetic testing per se that may reveal significant results such as a family genetic condition etc.

What will happen to the results of the research study?

The results will be examined by the researcher at the end of the study and a report will be produced. We will share our results with the funding body, the National Institute for Health Research. We may publish the study in a scientific journal and present the results at scientific meetings. You will not be identified in any of the reports, presentations or publications. We hope to use the results to undertake a larger, more definitive study.

A summary of the findings of the study can be made available to you if you wish from the research team or the principle investigator.

Who is organising and funding the research?

The research is being organised by Southend University Hospital. It is being funded by the National Institute of Health Research.

Who has reviewed the study?

For peer review only - http://bmjopen.bmj.com/site/about/guidelines.xhtml

PARTICIPANT INFORMATION SHEET (VERSION 1.1)

Centre Number: **Study Number:**

All research in the NHS is looked at by independent group of people, called a Research Ethics Committee, to protect your interests. This study has been reviewed and given a favourable opinion by The London Research Ethics Committee.

What if I become unwell during the study?

If during the study you become unwell or are concerned, as well as the usual services provided by the NHS, you can also contact a member of the study team on this number during normal working hours on <PI/Research member contact number>.

You can also contact <a research member of the team or the principle investigator out-of-hours> via the hospital switch board on this number <centre switch board number>.

If you are unwell, and need urgent advice or assistance do not delay in seeking further advice or treatment as usual through the NHS services.

Thank you for taking the time to read this information sheet and considering to take part in this study. If you would like more information or want to ask questions about the study please contact the study team.

For further information contact:

Research Nurse

Principal Investigator

<Contact details>

For peer review only - http://bmjopen.bmj.com/site/about/guidelines.xhtml

ASGARD

CONSENT FORM (VERSION 1.2)

Centre Number: **Study Number:**

Participation Id. no. for this trial:

ASGARD STUDY: How does a new treatment called Anakinra, an Interleukin-1 inhibitor, compare with the currently available best treatment, Steroids, for the treatment of gout attacks in people with kidney disease

Name of Researcher: Study reference:

This form must be completed and signed by the research participant in the presence of Principle Investigator or someone from the research team designated by the Principle Investigator

Please initial box

1. I confirm that I have read and understood the information sheet dated …………………. (Version……….). I have had the opportunity to consider the information, ask questions and have had these answered satisfactorily. ☐

2. I understand that my participation is entirely voluntary and that I am free to withdraw at any time without giving any reason, without my medical or legal rights being affected. ☐

3. I understand that relevant sections of my medical notes and data collected will be looked at by individuals who are part of the study team (including staff from Southend University Hospital, Anglia Ruskin University, University of East Anglia and University of Essex), from regulatory authorities or from the NHS Trust, where it is relevant to my taking part in this research. I give permission for these individuals to have access to my records. ☐

4. I understand, and I agree that my identifiable routine blood tests will be stored within the NHS clinical system and will be available to doctors looking after me in the future. ☐

5. I understand that my participation in the study is voluntary and that I am free to leave the study at any time without having to give a reason and that this will not affect my medical care in any way. ☐

6. I understand that I will be asked to refrain from using additional / rescue pain killers for four hours (normal time in-between doses) prior to an assessment, this may mean that I make a slight adjustment to perform an assessment before I take the next dose. ☐

7. I agree that if I withdraw or I am withdrawn from the study that data already collected can be retained and included in the data analysis. ☐

8. I understand that I will be required to give blood samples as part of this study. ☐

For peer review only - http://bmjopen.bmj.com/site/about/guidelines.xhtml

CONSENT FORM (VERSION 1.2)

Centre Number: **Study Number:**

Participation Id. no. for this trial:

9. I understand and I agree that the samples I provide will be gifted by myself for use in future research. They will be transferred to specialist laboratories and analysed by members of the study team or stored (link-anonymised) for up to fifteen years and can be used for future, as yet unspecified, medical research into health, illness and medical treatment. This research will be subject to proper scientific and ethical review. (Optional)	☐
10. I agree, if required, to undertake an interview and give permission for audio recordings that will be stored for a small qualitative aspect of this study. (Optional)	☐
11. I agree that my contact information can be made available to the research team as part of the study assessments, for any new findings that may become relevant during the course of the study and for future studies that I might be interested in.	☐
12. I give consent for storage and studies on my DNA. I understand that this is **not** genetic testing per se that may reveal significant results such as a family genetic condition. (Optional)	☐
13. I agree that samples can be exported to countries in the European Union for further analysis. The collaborators / recipient country will ensure that the material has been handled properly and that the required standards of that collaborating institution / country have been met. (Optional)	☐
14. I agree to my GP being informed of my participation in the study.	☐
15. I agree to take part in the above study.	☐

Name of Patient	Date	Signature

Name of Person taking consent	Date	Signature

When completed: 1 for participant 1 for researcher site file; 1 (original) to be kept in medical notes.

For peer review only - http://bmjopen.bmj.com/site/about/guidelines.xhtml

SPIRIT 2013 Checklist: Recommended items to address in a clinical trial protocol and related documents*

Section/item	Item No	Description	Addressed on page number
**Administrative information**			
Title	1	Descriptive title identifying the study design, population, interventions, and, if applicable, trial acronym	1
Trial registration	2a	Trial identifier and registry name. If not yet registered, name of intended registry	2
	2b	All items from the World Health Organization Trial Registration Data Set	2
Protocol version	3	Date and version identifier	1____
Funding	4	Sources and types of financial, material, and other support	2_____________
Roles and responsibilities	5a	Names, affiliations, and roles of protocol contributors	1 and submission details__
	5b	Name and contact information for the trial sponsor	main protocol (supplementary material)___
	5c	Role of study sponsor and funders, if any, in study design; collection, management, analysis, and interpretation of data; writing of the report; and the decision to submit the report for publication, including whether they will have ultimate authority over any of these activities	main protocol (supplementary material)___

For peer review only - http://bmjopen.bmj.com/site/about/guidelines.xhtml

	5d	Composition, roles, and responsibilities of the coordinating centre, steering committee, endpoint adjudication committee, data management team, and other individuals or groups overseeing the trial, if applicable (see Item 21a for data monitoring committee)	main protocol (supplementary material)___
**Introduction**			
Background and rationale	6a	Description of research question and justification for undertaking the trial, including summary of relevant studies (published and unpublished) examining benefits and harms for each intervention	_3____________
	6b	Explanation for choice of comparators	_5____________
Objectives	7	Specific objectives or hypotheses	_6____________
Trial design	8	Description of trial design including type of trial (eg, parallel group, crossover, factorial, single group), allocation ratio, and framework (eg, superiority, equivalence, noninferiority, exploratory)	_6____________
**Methods: Participants, interventions, and outcomes**			
Study setting	9	Description of study settings (eg, community clinic, academic hospital) and list of countries where data will be collected. Reference to where list of study sites can be obtained	_7____________
Eligibility criteria	10	Inclusion and exclusion criteria for participants. If applicable, eligibility criteria for study centres and individuals who will perform the interventions (eg, surgeons, psychotherapists)	_7____________
Interventions	11a	Interventions for each group with sufficient detail to allow replication, including how and when they will be administered	_7____________
	11b	Criteria for discontinuing or modifying allocated interventions for a given trial participant (eg, drug dose change in response to harms, participant request, or improving/worsening disease)	_8____________
	11c	Strategies to improve adherence to intervention protocols, and any procedures for monitoring adherence (eg, drug tablet return, laboratory tests)	_8___________
	11d	Relevant concomitant care and interventions that are permitted or prohibited during the trial	_8____________

For peer review only - http://bmjopen.bmj.com/site/about/guidelines.xhtml

Outcomes	12	Primary, secondary, and other outcomes, including the specific measurement variable (eg, systolic blood pressure), analysis metric (eg, change from baseline, final value, time to event), method of aggregation (eg, median, proportion), and time point for each outcome. Explanation of the clinical relevance of chosen efficacy and harm outcomes is strongly recommended	_8___________
Participant timeline	13	Time schedule of enrolment, interventions (including any run-ins and washouts), assessments, and visits for participants. A schematic diagram is highly recommended (see Figure)	_9___________
Sample size	14	Estimated number of participants needed to achieve study objectives and how it was determined, including clinical and statistical assumptions supporting any sample size calculations	_9___________
Recruitment	15	Strategies for achieving adequate participant enrolment to reach target sample size	_9__________
**Methods: Assignment of interventions (for controlled trials)**			
Allocation:			
Sequence generation	16a	Method of generating the allocation sequence (eg, computer-generated random numbers), and list of any factors for stratification. To reduce predictability of a random sequence, details of any planned restriction (eg, blocking) should be provided in a separate document that is unavailable to those who enrol participants or assign interventions	_10____________
Allocation concealment mechanism	16b	Mechanism of implementing the allocation sequence (eg, central telephone; sequentially numbered, opaque, sealed envelopes), describing any steps to conceal the sequence until interventions are assigned	_10___________
Implementation	16c	Who will generate the allocation sequence, who will enrol participants, and who will assign participants to interventions	_11____________
Blinding (masking)	17a	Who will be blinded after assignment to interventions (eg, trial participants, care providers, outcome assessors, data analysts), and how	_11____________
	17b	If blinded, circumstances under which unblinding is permissible, and procedure for revealing a participant's allocated intervention during the trial	_11____________
**Methods: Data collection, management, and analysis**			

Data collection methods	18a	Plans for assessment and collection of outcome, baseline, and other trial data, including any related processes to promote data quality (eg, duplicate measurements, training of assessors) and a description of study instruments (eg, questionnaires, laboratory tests) along with their reliability and validity, if known. Reference to where data collection forms can be found, if not in the protocol	_11____________
	18b	Plans to promote participant retention and complete follow-up, including list of any outcome data to be collected for participants who discontinue or deviate from intervention protocols	_12____________
Data management	19	Plans for data entry, coding, security, and storage, including any related processes to promote data quality (eg, double data entry; range checks for data values). Reference to where details of data management procedures can be found, if not in the protocol	_12___________
Statistical methods	20a	Statistical methods for analysing primary and secondary outcomes. Reference to where other details of the statistical analysis plan can be found, if not in the protocol	_12___________
	20b	Methods for any additional analyses (eg, subgroup and adjusted analyses)	_13____________
	20c	Definition of analysis population relating to protocol non-adherence (eg, as randomised analysis), and any statistical methods to handle missing data (eg, multiple imputation)	_13____________
**Methods: Monitoring**			
Data monitoring	21a	Composition of data monitoring committee (DMC); summary of its role and reporting structure; statement of whether it is independent from the sponsor and competing interests; and reference to where further details about its charter can be found, if not in the protocol. Alternatively, an explanation of why a DMC is not needed	_13____________
	21b	Description of any interim analyses and stopping guidelines, including who will have access to these interim results and make the final decision to terminate the trial	_13____________
Harms	22	Plans for collecting, assessing, reporting, and managing solicited and spontaneously reported adverse events and other unintended effects of trial interventions or trial conduct	__14___________
Auditing	23	Frequency and procedures for auditing trial conduct, if any, and whether the process will be independent from investigators and the sponsor	__15___________
**Ethics and dissemination**			

For peer review only - http://bmjopen.bmj.com/site/about/guidelines.xhtml

Research ethics approval	24	Plans for seeking research ethics committee/institutional review board (REC/IRB) approval	__15___________
Protocol amendments	25	Plans for communicating important protocol modifications (eg, changes to eligibility criteria, outcomes, analyses) to relevant parties (eg, investigators, REC/IRBs, trial participants, trial registries, journals, regulators)	___15__________
Consent or assent	26a	Who will obtain informed consent or assent from potential trial participants or authorised surrogates, and how (see Item 32)	__15___________
	26b	Additional consent provisions for collection and use of participant data and biological specimens in ancillary studies, if applicable	__16___________
Confidentiality	27	How personal information about potential and enrolled participants will be collected, shared, and maintained in order to protect confidentiality before, during, and after the trial	__16___________
Declaration of interests	28	Financial and other competing interests for principal investigators for the overall trial and each study site	___16__________
Access to data	29	Statement of who will have access to the final trial dataset, and disclosure of contractual agreements that limit such access for investigators	___17__________
Ancillary and post-trial care	30	Provisions, if any, for ancillary and post-trial care, and for compensation to those who suffer harm from trial participation	___17__________
Dissemination policy	31a	Plans for investigators and sponsor to communicate trial results to participants, healthcare professionals, the public, and other relevant groups (eg, via publication, reporting in results databases, or other data sharing arrangements), including any publication restrictions	___17__________
	31b	Authorship eligibility guidelines and any intended use of professional writers	__17___________
	31c	Plans, if any, for granting public access to the full protocol, participant-level dataset, and statistical code	__17___________
**Appendices**			
Informed consent materials	32	Model consent form and other related documentation given to participants and authorised surrogates	Supplementary material_________ ____

For peer review only - http://bmjopen.bmj.com/site/about/guidelines.xhtml

Biological specimens	33	Plans for collection, laboratory evaluation, and storage of biological specimens for genetic or molecular analysis in the current trial and for future use in ancillary studies, if applicable	_16____________

*It is strongly recommended that this checklist be read in conjunction with the SPIRIT 2013 Explanation & Elaboration for important clarification on the items. Amendments to the protocol should be tracked and dated. The SPIRIT checklist is copyrighted by the SPIRIT Group under the Creative Commons "Attribution-NonCommercial-NoDerivs 3.0 Unported" license.

For peer review only

For peer review only - http://bmjopen.bmj.com/site/about/guidelines.xhtml

Study protocol for a feasibility randomised multi-centre, double-blind, double-dummy controlled trial of Anakinra, an interleukin-1 receptor antagonist versus intramuscular methylprednisolone for acute gout attacks in patients with chronic kidney disease: ASGARD

Journal:	*BMJ Open*
Manuscript ID	bmjopen-2017-017121.R1
Article Type:	Protocol
Date Submitted by the Author:	28-Jun-2017

Complete List of Authors:	Balasubramaniam, Gowrie; Southend University Hospital NHS Foundation Trust, Department of Renal Medicine<br>Parker, Trisha; Anglia Ruskin University, Clinical Trials Unit<br>Turner, David; University of East Anglia, Public Health and Primary Care<br>Parker, Mike; Postgraduate Medical Institute, Anglia Ruskin University,<br>Scales, Jonathan; University of Essex, School of Health and Humanities<br>Harnett, Patrick; Southend University Hospital NHS Foundation Trust, Department of Renal Medicine<br>Harrison, Michael; Anglia Ruskin University, Clinical Trials Unit<br>Ahmed, Khalid; Princess Alexandra Hospital NHS Trust<br>Bhagat, Sweta; West Suffolk Hospitals NHS Trust<br>Marianayagam, Thiraupathy; East and North Hertfordshire NHS Trust<br>Pitzalis, Costantino; William Harvey Research Institute, Experimental Medicine and Rheumatology<br>Mallen, Christian; Keele University, Arthritis Research UK Primary Care Centre<br>Roddy, Edward; Keele University, Research Institute for Primary Care and Health Sciences; Keele University<br>Almond, Mike; Southend University Hospital NHS Foundation Trust, Department of Renal Medicine<br>Dasgupta, Bhaskar; Southend Hospital NHS Trust, Rheumatology

**Primary Subject Heading**:	Rheumatology
Secondary Subject Heading:	Renal medicine, Research methods
Keywords:	Acute gout, Chronic kidney disease, Feasibility, Randomised controlled trial, Anakinra, Intramuscular methylprednisolone

SCHOLARONE™ Manuscripts

Study protocol for a feasibility randomised multi-centre, double-blind, double-dummy controlled trial of Anakinra, an interleukin-1 receptor antagonist versus intramuscular methylprednisolone for acute gout attacks in patients with chronic kidney disease: ASGARD

Gowrie Balasubramaniam[1]*, Trisha Parker[2], David Turner[3], Mike Parker[2], Jonathan Scales[4], Patrick Harnett[1], Michael Harrison[2], Khalid Ahmed[5], Sweta Bhagat[6], Thiraupathy Marianayagam[7], Costantino Pitzalis[8], Christian Mallen[9], Edward Roddy[9], Mike Almond[1], Bhaskar Dasgupta[1]

Author affiliations

[1]Southend University Hospital NHS Foundation Trust, Prittlewell Chase, Southend, Essex, SS0 0RY

[2]Anglia Ruskin University, Post Graduate Medical Institute Clinical Trials Unit, Bishops Hall Lane, Chelmsford, CM1 1SQ

[3]Norwich Medical School, University of East Anglia, Norwich, NR4 7TJ

[4]School of Health and Human Sciences, University of Essex, Wivenhoe Park, Colchester, CO4 3SQ

[5]The Princess Alexandria Hospital NHS Trust, Hamstel Road, Harlow, Essex, CM20 1QX

[6]West Suffolk Hospital NHS Foundation Trust, Hardwick Lane, Bury St Edmunds, Suffolk, IP33 2QZ

[7]Lister Hospital, East and North Herfordshire NHS Trust, Corey Mills Lane, Stevenage, Hertfordshire, SG1 4AB

[8]Centre for Experimental Medicine and Rheumatology, William Harvey Research Institute, Barts and the London, Charterhouse Square, London EC1M 6BQ

[9]Primary Care and Health Sciences, Keele University, Keele, Staffordshire, ST5 5BG

*Corresponding author: gowrie@doctors.net.uk

Protocol version 1.4.

Word count 5666

Figures 2, tables 2

Conflict of interest: none

For peer review only - http://bmjopen.bmj.com/site/about/guidelines.xhtml

Abstract

Introduction: Acute gout occurs in people with chronic kidney disease, who are commonly older people with co-morbidities such as hypertension, heart disease and diabetes. Potentially harmful treatments are administered to these vulnerable patients due to a lack of clear evidence. Newly available treatment that targets a key inflammatory pathway in acute gout attacks provides an opportunity to undertake the first ever trial specifically looking treating people with kidney disease. This paper describes the protocol for a feasibility randomised controlled trial comparing Anakinra, a novel interleikin-1 antagonist versus steroids in people with chronic kidney disease (ASGARD).

Methods and Analysis: ASGARD is a two-parallel group double-blind, double dummy multicentre randomised controlled trial comparing Anakinra 100mg, an interleukin-1 antagonist, subcutaneous for five days against intramuscular methylprednisolone 120mg. The primary objective is to assess the feasibility of the trial design and procedures for a definitive randomised controlled trial. The specific aims are: 1) test recruitment and retention rates, and willingness to be randomised; 2) test eligibility criteria; 3) Collect and analyse outcome data to inform sample and power calculations for a trial of efficacy; 4) collect economic data to inform a future economic evaluation estimating costs of treatment; 5) assess capacity of the project to scale up to a national multi-centre trial. We will also gather qualitative insights from participants. It aims to recruit 32 patients with a 1:1 randomisation.

Information from this feasibility study will help design a definitive trial and provide general information in designing acute gout studies.

Ethics and Dissemination: The London-Central Ethics Committee approved the protocol. The results will be disseminated in peer-reviewed journals and at scientific conferences.

Registration details: EudraCT No. 2015-001787-19, NCT / Clinicalstrials.gov No. NCT02578394, WHO Universal Trials Reference No. U1111-1175-1977. NIHR Grant PB-PG-0614-34090.

For peer review only

For peer review only - http://bmjopen.bmj.com/site/about/guidelines.xhtml

Strengths and limitations of this study

Assessing the feasibility of undertaking a definitive robustly designed double-blind, double dummy study.

Qualitative aspects to current study design will help future study be more patient-orientated.

Current study is not designed to find differences in outcomes.

For peer review only

Introduction

Background and rationale

Chronic kidney disease (CKD) affects 5% of the UK population (1) and 40% of patients with CKD 3 and 4 have chronic gout (2), suggesting that 1.32 million people, predominantly older patients, have CKD and gout in the UK. The overall incidence of acute gout attacks has been estimated to be approximately 2 per 1000 person-years (3,4). The prevalence of gout is increasing due to increasing prevalence of co-morbid conditions that are associated with hyperuricaemia such as hypertension, obesity, metabolic syndrome, diabetes and chronic kidney disease (4,5).

In a UK study, approximately 89.4% of patients were treated for their acute attack with non-steroidal anti-inflammatory agents (NSAIDS) which are well known to worsen kidney failure (5). Secondary care data from the US showed that up to 50% of acute gout patients had CKD and 40% were given contra-indicated medications (6). This is similar to our experience where a review of in-patient acute gout care showed half of all patients with acute gout and kidney disease were given potentially harmful medications. Mismanagement of acute gout attacks in patient with kidney disease occurs as there is no firm evidence-base for treating acute gout attacks in people with kidney disease (7).

The lack of evidence in the literature for the use of conventional agents in patients with CKD is primarily because patients with CKD are excluded as NSAIDS are used as an active comparator in randomised controlled trials. Guidelines continue to suggest using colchicine with in patients with renal disease even with its toxicity profile and poor evidence base (8,9,10). We now have an opportunity to conduct a trial using two agents that would not be expected have adverse effects on renal function.

Treatment options for patient with acute gout attacks and CKD

For peer review only - http://bmjopen.bmj.com/site/about/guidelines.xhtml

NSAIDS have the largest evidence base for use in acute gout but are contra-indicated in CKD as they can cause an acute kidney injury. They can also worsen heart failure, hypertension, liver failure and cause gastro-intestinal bleeding (7,11). Colchicine is an alternative anti-inflammatory agent, it has a narrow therapeutic index and a low dose regime is advocated following findings in the AGREE trial. Even though this study excluded patients with moderate to severe kidney disease (glomerular filtration rate < 60 mls/m^3), 23% of patients on the low dose treatment still experienced diarrhoea (12). Diarrhoea is more common in patients with CKD, they are also more likely to have drug accumulation and serious side effects from toxicity such as bone marrow failure, rhabdomyolysis, pancreatitis and myopathy (7).

Glucocorticoids are an effective treatment with some evidence for use in CKD patients from older trials. Triamcinolone, a long-acting synthetic corticosteroid, was shown to be as effective as adrenocorticotrophic hormone, a hormone that increases endogenous cortisol secretion (11). A more recent double-blind randomised controlled study (13) showed that Prednisolone 35mg for 5 days was not inferior to Naproxen for acute gout attacks, although patients with moderate to severe renal impairment were excluded.

Interleukin-1 inhibitors

Interleukin-1 (IL-1) signalling has been shown to play a key role in gout associated inflammation (14,15) and targeted therapy is now available. Two agents of IL-1 have been used in randomised controlled trials. Rilonocept, an IL-1 decoy receptor, has been shown to be efficacious in reducing acute flares during the initiation of urate lowering therapy (16,17) but no added benefit was obtained for the treatment of acute gout when it was used in addition to an NSAID (18). Canakinumab, a monoclonal antibody against IL-1 beta showed efficacy preventing acute flares during the initiation of urate lowering therapy (19), and for acute gout in patients with difficult disease or contra-indication to colchicine or NSAIDs (20,21,22). It was associated with increased adverse events after a single injection (23) which was felt in part due to its prolonged action which can be for many weeks (24). This study will use anakinra (Kineret), an IL-1 receptor antagonist (IL-1 RA) that competes with IL-1beta for its receptor (IL-1R). A pilot study of its use in gout showed prompt resolution of symptoms in patients treated with anakinra (25), including in three patients with renal impairment. There have been further reports of the use of anakinra in difficult to treat cases showing good efficacy, with most patients having a good response within twenty-four hours (26-31).

Anakinra has a good pharmacokinetic profile with a shorter duration of action, accumulation can occur in severe renal failure (32) and this had been linked to an increased risk of

For peer review only - http://bmjopen.bmj.com/site/about/guidelines.xhtml

infection in one case report (31). The dose of a daily 100mg has been used in studies looking at treatment of Rheumatoid Arthritis (usually between 50mg to 150mg) and it was well tolerated for up to three years (33-37). The licensed use of Anakinra is for Cryopryin Associated Periodic Syndrome where dosing is 3mg – 4mg per kg in severe disease (doses up to 800mg) (38). The rationale for the duration of treatment use of 100mg s/c for 5 days come from emerging data where Anakinra has been used to treat acute gout with good efficacy and in patients with CKD without too many adverse events (25-29). Some reports vary between 3 to 5 days but most use 5 days' duration of treatment to ensure adequate response in all patients, including our own experience (30). We have intentionally avoided using Anakinra in patients with severe renal failure (GFR < 30mls/min/1.73m^2) although it has been used safely in patient on haemodialysis using an alternate regime (39) and we have some experience of this in our centre (30). Anakinra is much cheaper compared to Canakinumab (£131.15 vs. £9927.90 for a course of treatment), which has been given a license in the UK for use in difficult to treat gout.

Safety experience stem mainly from studies in Rheumatoid Arthritis where patients were on concurrent immunosuppressive treatment and the duration of treatment was prolonged (up to six months). A slightly higher risk of infection (2%) and neutropenia was noted. We do not anticipate these issues as the treatment time for acute gout will be short, five days versus many months, and we feel the risk of infection should be much less. We are excluding people with concurrent immunosuppressive treatment and will exclude people with serious infection. There is some data of the use of anakinra in the setting of an active infection from the first clinical trial where it was used to treat people with severe septicaemia and those who received Anakinra were not worse off (40). There are also reports of the use of Anakinra for acute gout for hospitalised in-patients with active infections and multiple co-morbidities (27-29). There was one case report of neutropenia in one patient with a kidney transplant with severe renal impairment (31). Blood counts will be checked at the end of treatment at day 5 and participants will also be assessed for infection face-to-face on day 2, day 7 and 8 weeks, and by telephone contact on day 3, day 4 and day 5. Participants will be asked to stop treatment if serious infection develops.

Methylprednisolone

Methylprednisolone acetate is one of the most commonly used long-acting intra-muscular agent in the UK, we have shown this in our region (41), and it is equivalent to Triamcinolone in other joint conditions (42).

We are using methylprednisolone 120mg given by intramuscular injection which is as efficacious as oral steroids with a lower cumulative dose (120mg vs.175mg) in other

For peer review only - http://bmjopen.bmj.com/site/about/guidelines.xhtml

rheumatology conditions, and this is equivalent to the dose of oral prednisolone used in the trial by Janssens et al. (13) (35 mg for 5 days). This well designed double-blind randomised controlled study showed that Prednisolone 35mg for 5 days was not inferior to Naproxen. The rate of side-effects even with short-term steroid treatment in acute gout is unknown. Our study may help establish steroids, especially intramuscular methylprednisolone which has limited evidence base, as a potential safe, cheap, and readily available treatment for acute gout in patients with renal disease. If the initial results show equivalent efficacy between the two treatment arms, then we could go on to establish steroids as the standard treatments and make considerable savings.

Our use is only as a one-off injection (as is commonly done in routine practice) and we do not anticipate long-term complications. Short term side-effects we may encounter are uncontrolled blood sugars in diabetics and worsening odeama in people with heart failure and kidney failure. The latter should occur less with methylprednisolone which is associated with less mineralocorticoid effect than other steroids (e.g. prednisolone). The placebo equivalent for the methylprednisolone will be lipofundin, a lipid emulsion commonly used in parenteral nutrition but has been use as a placebo agent in other trial using steroids.

Objectives

Primary objective

The primary objective of ASGARD is to assess the feasibility of the trial design and procedures for a definitive randomised controlled trial. The specific aims are: 1) test recruitment and retention rates, and willingness to be randomised; 2) test eligibility criteria; 3) Collect and analyse outcome data to inform sample and power calculations for a trial of efficacy; 4) collect economic data to inform a future economic evaluation estimating costs of treatment; 5) assess capacity of the project to scale up to a national multi-centre trial. We will also gather qualitative insights from participants about their experience from being in the feasibility trial to help design the subsequent definitive trial.

Secondary objectives

Information will be obtained to help with the design of the subsequent study including testing of proposed primary and secondary outcomes described below.

Trial Design

ASGARD is a two-parallel group double-blind, double dummy multicentre randomised controlled trial with a 1:1 allocation ratio comparing Anakinra 100mg subcutaneous for five

For peer review only - http://bmjopen.bmj.com/site/about/guidelines.xhtml

days with a one-off methylprednisolone (Depo-Medrone) 120mg intramuscular injection (Figure 1) (Supplementary material).

Methods

Study setting

ASGARD aims to recruit from centres in the East of England region. Recruitment will be from patients who present to primary and secondary care with acute gout and chronic kidney disease, this will also include in-patient who develop an acute gout attack. Known patients with moderate kidney disease will also be primed to contact the research team by working with local primary care providers to send out letters of invitation. Recruitment will occur in the secondary care setting with treatment occurring on an out-patient basis, or can occur in secondary care if symptoms warrant admission or if the participant is already an in-patient and develops an acute gout attack.

Eligibility criteria

Table / Box 1

Table / Box 2

Baseline Assessments

Baseline assessment will include basic medical observations, routine bloods, and basic clinical examination. The 2015 American College of Rheumatology/ European League Against Rheumatism Collaborative Initiative will also be used at baseline assessment (43).

Interventions

Eligible participants will undergo 1:1 block randomisation to one of two treatment arms; anakinra 100mg s/c daily for five days and one-off placebo methylprednisolone (lipofundin MCT) intramuscular (gluteal) or one-off intramuscular methylprednisolone (Depo-Medrone) and placebo anakinra s/c for five days. The study drug and its placebo will be provided by SOBI. Lipofundin will be purchased by the co-ordinating centre pharmacy by the usual route.

For anakinra and its equivalent placebo, participants will be issued a pharmacy box with individual syringes in syringe holders, and a sharps bin. Each syringe will be labelled day 1 (already administered) up to day 5. Day 1 treatment will be administered after baseline assessment and investigations.

The participant will be taught to self-administer the sub-cutaneous injection. Participant will then attend on day 2 for assessment of self-injection and subsequently will undertake self-

For peer review only - http://bmjopen.bmj.com/site/about/guidelines.xhtml

injection themselves on day 3, 4 and 5. Participants unable to self-inject will receive their injections from a carer, family member, or district nurse.

Intramuscular methylprednisolone or lipofundin syringes will be prepared by a member of the research team not involved with the trial assessments (i.e. un-blinded research nurse).

Modifications

Injection sites will be assessed visually on day 2 and day 7, and by telephone contact from day 3 to day 6. Blood tests will be taken on day 2 and day 7 to check for neutropenia and any signs of infection. Treatment will be stopped in the event of any adverse reaction during subcutaneous injections, oral prednisolone 35mg po for five days will be used as rescue treatment if symptoms of acute attack persist. Side-effects to methylprednisolone such as worsening odeama and uncontrolled diabetes (if diabetic) will be monitored and reported as adverse events.

Adherence

Participants will be contacted everyday up to from day 3 to day 7, to remind them of injection up to day 5 and of diary entry up to day 7 (except over the weekend). Syringe holders will be accounted for and the number of syringes returned in the sharps box will be counted through the lid (not strictly) ay day 7 before permanently locked by the trial team for disposal.

Concomitant care

Rescue medications

Participants can take analgesia listed in the protocol as non-investigational medicinal product (co-dydramol, codeine phosphate, tramadol, and paracetamol) for relief as required, usage will be recorded. If participants get another flare during treatment, this will be recorded and a coarse of oral prednisolone 35mg po od for 5 days will be used for rescue treatment. Low dose colchicine 500micrograms po bd could be used in addition. Use of other immune modifying treatment or NSAIDS is prohibited.

Outcomes

This is a feasibility study. It is not powered to look for inference. Study processes will be analysed. Recruitment and retention rates and willingness of patients to be randomised will be calculated. The proportion of patients who did not meet eligibility criteria will be examined. Adherence and compliance rates and qualitative feedback will be examined. Economic data on healthcare resource use and health-related quality of life will be collected. Safety

For peer review only - http://bmjopen.bmj.com/site/about/guidelines.xhtml

outcome measures will be reported as mandated in a clinical trial of an investigational medicinal product protocol.

Primary endpoint/outcome

Feasibility of undertaking a definitive multicentre, double-blind, double dummy randomised controlled trial to obtain clear guidance on the safe management of acute gout attacks in patients with chronic kidney disease.

Information from proposed outcome measures for the larger study will be sought. Proposed primary outcome measures of effectiveness consist of resolution of pain i.e. time to 50% reduction and complete resolution of pain in self-assessed pain intensity in the joint most affected at baseline measure on the VAS (0-100mm) and 5-point Likert scale from baseline to 7 days' post randomisation (Day 1, Day 2, Day 3, Day 4, Day 5, Day 6, Day 7) using composite time points kept to 24 hour intervals as close as possible.

Proposed secondary outcome measures will consist of patient reported outcome measure (Day 1, Day 2, Day 3, Day 4, Day 5, Day 6 and Day 7), physician assessment of joint tenderness and swelling (Day 1, Day 2, and Day 7), assessment of activity limitation and quality of life: HAQ-DI and SF-36 at Day 1, Day 7 and 8 weeks; EQ-5D-5L at Day 1, Day 2, Day 7 and 8 weeks; LEFS at Day 1, Day 2, Day 7. Time to take rescue medication and a limited assessment of healthcare resource will be made.

Blood and urine tests renal on Day 1, Day 2, Day 7 and 8 weeks, consisting of FBC, U+Es, eGFR, glucose, CRP, serum uric acid and spot urine uric acid.

Participant timeline

Figure 2.

Sample size

This is a feasibility study and we will aim to recruit at least 16 patients in each arm of the study. The sample size was obtained from a recommendation based on the feasibility of running a parallel group design. The study will aim to enrol for 15 months.

Recruitment

Recruitment will be from secondary care, either when potential participants present with acute gout attack or if in-patients develop acute gout attack. Trial centres will be encouraged to work with primary care centres to identify potential participants and give letter of invitation.

For peer review only

For peer review only - http://bmjopen.bmj.com/site/about/guidelines.xhtml

We will use ethically approved advertising material in the community. Potential participants will be given or sent a participant information sheet (Supplementary material).

Our hospital serves a population of 350,000 and had an average of 47 patients per year over the last four years with approximately 50% of patients having chronic kidney disease (i.e. 23 patients per year). Enrolment is for 15 months from 6 centres, we will only recruit patients in working hours i.e. 9am – 5pm on Mondays to Thursdays, it would be logistically difficult to avoid delays to treatment if participants present out of hours and face-to-face assessments cannot be done at weekends due to lack of research infrastructure in most small to medium sized hospital. All hospitals should be able to have pharmacy support to provide preparation and recording of treatment administered.

Potential participants would hopefully be fully aware of study if a letter of invitation was already sent to them. Otherwise, potential participants can have up to twenty-four hours to decide if they wish to take part in the study. Analgesia that is listed can be used if required; however, baseline assessments can only occur four hours after administration of last dose, i.e. before the administration of the next dose. Treatment cannot be withheld during this period and exclusion criteria will be applied if duration of attack is longer than accepted or if contra-indicatory medication is administered.

Allocation

Sequence generation

Enrolled patients will undergo 1:1 randomisation in blocks of four. Randomisation will be undertaken using an Internet-based randomisation system within ARCTU. The ARCTU uses the TENALEA system provided by the Trans European Network for Clinical Trial Service.

Concealment mechanism

Once a patient has consented to take part in the trial, the designated staff will log in to the TENALEA web page to confirm eligibility and a random allocation will be sent to pharmacy. All investigators involved with the trial will be blinded to treatment. Patients will not be informed of their assigned treatment during the study. Pharmacy will keep a record of allocated treatment arms in the event emergency un-blinding is required.

Implementation

Anakinra and its placebo will be supplied by the manufacturer. Intramuscular methylprednisolone and its placebo (lipofundin) will be prepared by a research team member not involved with the study.

For peer review only - http://bmjopen.bmj.com/site/about/guidelines.xhtml

Study labels will be provided to preserve blinding. Anakinra and its equivalent placebo will be provided by the manufacturer, one allocation is known, pharmacy will use label stating “Anakinra or placebo” using standard operating procedure. Five individual syringes labelled 1 to 5 will be placed individual transparent syringe holders and packed for dispensing with a local pharmacy label. For intramuscular methylprednisolone (depo-Medrone) and lipofundin, a member of the study team not involved with the trial will draw the treatment and label with a study label stating “Depomedrone or placebo,” using a standard operating procedure.

Blinding

All trial investigators will be blinded to the intervention. Pharmacy will keep a record of treatments administrated. All trial participants, care providers and outcome assessors will be blinded to the treatment. Each treatment has its equivalent placebo to ensure blinding in maintained throughout study.

Emergency Un-blinding

The treatment code for a participant can be broken by any clinician either directly or via contact of the PI, allocation lists are available to the site pharmacy will provide 24-hour cover. Failing that, the central pharmacy can access allocation list. Where possible the local investigator should aim to discuss the need for un-blinding with the co-ordinating investigator and blind should be endeavoured to be preserved to relevant research staff (data collection, analysis, and interpretation). The co-ordinating investigator is responsible for pharmacovigilance management and reporting.

METHODS: DATA COLLECTION, MANAGEMENT, ANALYSIS

Data collection methods

Data collection will be by electronic clinical research forms (eCRF), these can be printed out if required for convenience of data collection (and act as source document) and then data inputted onto eCRF. Data can also be inputted direct onto eCRF and the completed eCRF can be printed out to act as source documents.

Training plans

Study investigators must be aware of the classification criteria for gout. For the purposes of this study, each principal investigator will be asked to go through a presentation that will be available online and asked to attend study days where concerns will be raised.

Quality control of data and results

For peer review only - http://bmjopen.bmj.com/site/about/guidelines.xhtml

Entered data will continuously reviewed by the data manager and any gaps in data will be fed back to the centre trial team. Routine blood results will be taken from the NHS system. Samples for additional testing will be transported as per standard operating protocol to the co-ordinating centre.

Participant Retention and Withdrawal

Enrolled participants will be contacted daily (weekdays) by the study team for a week. Participants may withdraw from the study for any reason at any time. The investigator may also withdraw participants from the study to protect their safety and / or if they are unwilling or unable to comply with required study procedures after discussion with the chief investigator. Participants who ask to stop study treatment, become pregnant or develop a serious infection (necessitating intravenous anti-biotic treatment and hospital admission) should continue to attend all follow-up study. Participants who withdraw their consent at any point will be asked of their wish regarding the use of data collected up to the point of withdrawal. Participants who do not attend follow-up assessment after site staff have attempted to contact the patient at least twice, e.g. by telephone may be considered for withdrawal, the data already collected for them may be used and therefore needs returning in the usual manner. The PI responsible for a patient may choose to withdraw a patient from a trial for appropriate medical reasons, be they individual adverse events or new information gained about a treatment.

Data management

Information from the patient diary and functional assessments will be entered onto an electronic CRF. Data will be entered as it is possible to do so i.e. day 1, day 2, day 7 and 2 months. Referential data rules, valid values and range checks will be support by the data entry software. The data manager will review the data being sent at regular intervals and report back to the centre if there is any discrepancy. All forms, hard drives and storage devices related to the study will be kept in locked cabinets. Access to the study data will be restricted; a password system will be used to control access. Data will be stored at MACRO database.

Study status reports

Regular updates of the study will be sent to the principle investigators via email and will be posted on the study website.

Statistical methods

For peer review only - http://bmjopen.bmj.com/site/about/guidelines.xhtml

For peer review only

The sample size will be too small for estimates to have adequate precision, and some methods will not be technically possible for some patterns of missing values. To make use of data obtained, comparison of groups with the use of time-to-event ("survival") analysis will be undertaken where relevant. Comparison of continuous outcome measures will be undertaken with repeated measures analysis of variance with permutation tests. Two-sample comparisons of means will be by the two-sample permutation test. The association between the intervention and categorical variables will be tested using Fisher's exact test.

Economic analysis

Economic evaluation

Formal economic evaluation looking at the incremental cost-effectiveness of Anakinra compared to the comparator is not planned as this is a feasibility study. We will collect relevant data on resource use (costs) and health-related quality of life to inform subsequent large scale study. Key drivers of costs, resource allocation and scalability will be determined.

Qualitative Analysis

Twelve participants who give their specific informed consent for participation in the qualitative study will selected for an interview around their last eight-week follow-up. The interview will cover areas such as how participants became involved with the trial, the information that was provided for them, the process of obtaining informed consent and any areas that felt was not adequately covered or could have been undertaken differently.

Data monitoring

The Trial Steering Committee (TSC) will consist of the CI, trial co-ordinator, funding co-applicants and two members of patient group will bring independent representation. The TSG will take on some aspects of pharmacovigilance. There will be no data monitoring committee as this is a small feasibility study with a short duration and short follow-up, and pharmacovigilance is a component of the study. The Trial Management Group (TMG) will consist of the CI, co-CI, funding co-applicants, representative of the clinical trials units including trial co-ordinator, data manager and statistician. They should meet every two months to ensure all practical details of the trial are progressing well and adequate targets are met. The patient group consists of patients (and family) from Southend University hospital with chronic kidney disease and suffer from acute gout attacks.

Interim analysis

For peer review only - http://bmjopen.bmj.com/site/about/guidelines.xhtml

There will be no interim analysis as this is a feasibility study, the study procedures will be regularly reviewed at the trial steering committee.

Harms

Reporting of adverse events will start at the point of consent and reporting for adverse reaction starts at first IMP dose until 7 days' post treatment. At each contact with the subject during the treatment period, the investigator must seek information on adverse events by specific questioning and, as appropriate, by examination. All clearly related signs, symptoms, and abnormal diagnostic procedures should be recorded using the NCI CTCAE v4.0 event terms and grading. The clinical course of each event should be followed until resolution or stabilisation.

Reporting will follow local operating procedure and study protocol with oversight from the co-ordinating centre. Non-serious adverse events or reactions will be recorded in the study file with follow-up; and reported for review by the co-ordinating investigator within a month. All SAEs* / SUSARs must be recorded on the SAE form / AECRF Form and faxed to the Sponsor within 24 hours of the research staff becoming aware of the event. Any change of condition or other follow-up information should be faxed to the Sponsor as soon as it is available or at least within 24 hours of the information becoming available. Events will be followed up until the event has resolved or a final outcome has been reached. All SAEs assigned by the PI or co-ordinating investigator as both suspected to be related to IMP-treatment and unexpected will be classified as SUSARs and will be subject to expedited reporting to the REC and Medicines and Healthcare Products Regulatory Agency (MHRA). The CI will complete the CIOMS form and if warranted, an investigator alert may be issued, to inform all investigators involved in any study with the same drug (or therapy) that this serious adverse event has been reported.

Potential events/reactions reported with Anakinra consist of neutropenia, infection and less serious ones consist of injection site reactions such as erythema, pruritus and rash. Potential events/reactions with steroid treatment consist of worsening hypertension, blood sugar reading/uncontrolled diabetes and clinical signs of fluid retention. Pre-existing conditions should not be reported as AE unless the condition worsens by at least CTCAE grade during trial. The condition must be reported in the pre-treatment section of the eCRF, if symptomatic at the time of entry, or under concurrent medical conditions if asymptomatic. Given the potential of clinical events related to underlying co-morbid disease burden of potential participants, events that are recognised and expected complications of the condition are exempt from normal reporting procedure, unless they are of an unexpected severity. Common expected events in these patients may consist of worsening complications

For peer review only - http://bmjopen.bmj.com/site/about/guidelines.xhtml

of chronic kidney disease, heart failure, hypertension, diabetes complications, underlying cardiac disease with complications such as ACS, arrhythmia, hypotension, tachycardia and worsening oedema. Other exceptions to (S) AE or (S) AR reporting consist of routine treatment or monitoring of the studied indication not associated with any deterioration in condition, associated with any deterioration in condition, e.g. pre-planned hip replacement operation which does not lead to further complications, any admission to hospital or other institution for general care where there was no deterioration in condition, treatment on an emergency, outpatient basis for an event not fulfilling any of the definitions of serious as given above and not resulting in hospital admission, any death or hospitalisation due to fall or fracture, any death or hospitalisation due to exacerbation of an existing medical co-morbid condition

Auditing

The study will be subject to monitoring, inspection and audit by Anglia Ruskin Clinical Trials Unit, Southend University Hospital NHS Foundation Trust acting as the Sponsor, and other regulatory bodies to ensure adherence to Good Clinical Practice (GCP).

Ethics and Dissemination

The study will be conducted in accordance with the principles of Good Clinical Practice. A favourable ethical opinion was obtained from the London-Central Research Ethics Committee, reference number 15/LO/1922. Results will be published in peer-reviewed journal and disseminated at international conferences.

Protocol amendments

Protocol amendments to the protocol or study documents will be submitted to the REC and can only be implemented once approval has been obtained. Amendments to the CTA or documents that supported the original CTA application will be notified to the MHRA. Amendments will be tracked in the protocol appendix and the version of the protocol will be updated.

Consent

A clinician in the research team will obtain informed consent from potentially eligible participants. Patients who are unable to consent for themselves for suitability for the trial will not be approached as obtaining patient reported outcome measures will not be practical. If verbal translation is needed, this should be via a hospital interpreter or a personal interpreter. Telephone interpretation services are not acceptable and written material will not

For peer review only - http://bmjopen.bmj.com/site/about/guidelines.xhtml

For peer review only

be provided in various languages for this feasibility study. This study utilises questionnaire surveys that have not been validated in different languages. There is also a qualitative element to the study where lack of required language skill may prove to be difficult. Time is limited for potential participants to be considering participation and any delays in seeking consent and translation services that may prolong treatment of the patient in acute pain have also to be considered.

Written material consisting of participant information leaflet and consent documentation (Supplementary material) that has been approved by the Research Ethics Committee and will be compliant with GCP, local regulatory and legal requirement. This is a feasibility study and the costs to undertake centrally commissioned translated documents may be too high. This may be something to consider for the subsequent definitive study.

There will be opportunity for the participant to ask questions to the PI or a member of the research team. Potential participants can have up to twenty-four hours to consider the information and their participation. However, treatment cannot be withheld during this period and exclusion criteria will be applied if duration of attack is longer than accepted or if contra-indicatory medication is administered.

Ancillary studies

There will be a tertiary / exploratory study where patient's serum and urine will be stored for future analysis at the end of the study. Testing will be an extension of routine laboratory testing to look for markers of inflammation in an exploratory setting.

Confidentiality

All investigators and trial site staff must comply with the requirements of the Data Protection Act 1998 with regards to the collection, storage, processing, and disclosure of personal information and will uphold the Act's core principles. Information with regards to study patients will be kept confidential and managed in accordance with the Data Protection Act, NHS Caldicott Guardian, The Research Governance Framework for Health and Social Care and Research Ethics Committee Approval.

Data will be stored for five years before being destroyed. The Chief Investigator in the data custodian.

Declaration of interest

The co-ordinating investigator has not obtained any personal grants, a investigator sponsored study grant of £10,000 and provision of Anakinra and its equivalent placebo is

For peer review only - http://bmjopen.bmj.com/site/about/guidelines.xhtml

being provided for the study by the manufacturer's. This fund will be used to cover any predominantly pharmacy and logistics costs.

Access to data

Only the data manager will have access to data, and co-ordinate access to relevant researchers. The health economist will have access to health economic data set and qualitative researcher will have access of patient demographics to obtain a broad sample.

Ancillary and post-trial care

Acute gout attack episodes will be treated as part of this trial. If participants get another acute attack of gout, they will not be eligible to entry into the study. The participating team could consider using information with regards to treatment to avoid and available treatment options, but no information will be made available prior to the end of the trial.

Dissemination policy: trial results and authorship

The trial report will be used for publications and presentation at scientific meetings. All publications and presentations will be reviewed by the TMG. Authorship will be determined per internationally agreed criteria for authorship. Funding bodies will be declared in publications. The full report will be made available directly from the chief investigator or from published material. Supplemental material linked with publication will consist of the trial protocol.

For peer review only

Contributorship statement

GB conceived the idea, designed the study, wrote the protocol and the manuscript. BD helped develop the design and the protocol. MA and ER helped refine the design, protocol and the manuscript. JS designed the qualitative aspect of the study. MP undertook the statistical aspect of study design. MT designed the economic evaluation aspect of the study. MH and TP helped developed the logistical aspects of trial involving the trial unit. CM, PH, KA, TM, SB, CP helped refine the study. All authors approved the final manuscript.

Competing interests

There are no competing interests.

Funding

This paper presents independent research funded by the NIHR under its Research for Patient Benefit (RfPB) Programme (Grant Reference Number **PB-PG-0614-34090**. The views expressed are those of the author(s) and not necessarily those of the NHS, the NIHR or the Department of Health.

There is an additional investigator initiated study fund of £10,000 from SOBI to help with pharmacy logistics.

Data sharing statement

This manuscript is a study protocol and as yet no data is available.

For peer review only

References

1) Fraser SD, Roderick PJ, Aitken G, Roth M, Mindell JS, Moon G, et al. Chronic kidney disease, albuminuria and socioeconomic status in the Health Surveys for England 2009 and 2010. J Public Health (Oxf). 2014 Dec;36(4):577-86.

2) Juraschek SP, Kovell LC, Miller ER 3rd, Gelber AC. Association of kidney disease with prevalent gout in the United States in 1988-1994 and 2007-2010. Semin Arthritis Rheum. 2013 Jun;42(6):551-61.

3) Rothenbacher D, Primatesta P, Ferreira A, Cea-Soriano L, Rodríguez LA. Frequency and risk factors of gout flares in a large population-based cohort of incident gout. Rheumatology (Oxf). 2011 May;50(5):973-81.

4) Kuo CF, Grainge MJ, Mallen C, Zhang W, Doherty M. Rising burden of gout in the UK but continuing suboptimal management: a nationwide population study. Ann Rheum Dis. 2015 Apr;74(4):661-7.

5) Annemans L, Spaepen E, Gaskin M, Bonnemaire M, Malier V, Gilbert T, Nuki G. Gout in the UK and Germany: prevalence, comorbidities and management in general practice 2000-2005. Ann Rheum Dis. 2008 Jul;67(7):960-6.

6) Keenan RT, O'Brien WR, Lee KH, Crittenden DB, Fisher MC, Goldfarb DS, et al. Prevalence of contraindications and prescription of pharmacologic therapies for gout. Am J Med. 2011 Feb;124(2):155-63.

7) Curiel RV, Guzman NJ. Challenges associated with the management of gouty arthritis in patients with chronic kidney disease: a systematic review. Semin Arthritis Rheum. 2012 Oct;42(2):166-78.

8) Jordan KM, Cameron JS, Snaith M, Zhang W, Doherty M, Seckl J, et al. British Society for Rheumatology and British Health Professionals in Rheumatology guideline for the management of gout. British Society for Rheumatology and British Health Professionals in Rheumatology Standards, Guidelines and Audit Working Group (SGAWG). Rheumatology (Oxf). 2007 Aug;46(8):1372-4.

9) Richette P, Doherty M, Pascual E, Barskova V, Becce F, Castañeda-Sanabria J, et al. Zhang W, Doherty M, Pascual E et al. 2016 updated EULAR evidence-based recommendations for the management of gout. Ann Rheum Dis. 2017 Jan;76(1):29-42.

For peer review only - http://bmjopen.bmj.com/site/about/guidelines.xhtml

10) Khanna D, Khanna PP, Fitzgerald JD, Singh MK, Bae S, Neogi T, et al. American College of Rheumatology guidelines for management of gout. Part 2: therapy and anti-inflammatory prophylaxis of acute gouty arthritis. American College of Rheumatology. Arthritis Care Res (Hoboken). 2012 Oct;64(10):1447-61.

11) Siegel LB, Alloway JA, Nashel DJ. Comparison of adrenocorticotropic hormone and triamcinolone acetonide in the treatment of acute gouty arthritis. J Rheumatol. 1994 Jul;21(7):1325-7.

12) Terkeltaub RA, Furst DE, Bennett K, Kook KA, Crockett RS, Davis MW. High versus low dosing of oral colchicine for early acute gout flare: Twenty four-hour outcome of the first multicenter, randomized, double-blind, placebo controlled, parallel-group, dose-comparison colchicine study. Arthritis Rheum. 2010 Apr;62(4):1060-8.

13) Janssens HJ, Janssen M, van de Lisdonk EH, van Riel PL, van Weel C. Use of oral prednisolone or naproxen for the treatment of gout arthritis: a double-blind, randomised equivalence trial. Lancet. 2008 May 31;371(9627):1854-60.

14) Dinarello CA. Interleukin-1 in the pathogenesis and treatment of inflammatory diseases. Blood. 2011 Apr 7;117(14):3720-32.

15) Di Giovine FS, Malawista SE, Nuki G, Duff GW. Interleukin 1 (IL 1) as a mediator of crystal arthritis. Stimulation of T cell and synovial fibroblast mitogenesis by urate crystal-induced IL 1. J Immunol. 1987 May 15;138(10):3213-8.

16) Mitha E, Schumacher HR, Fouche L, Luo SF, Weinstein SP, Yancopoulos GD, et al. Rilonacept for gout flare prevention during initiation of uric acid-lowering therapy: results from the PRESURGE-2 international, phase 3, randomized, placebo-controlled trial. Rheumatology (Oxf). 2013 Jul;52(7):1285-92.

17) Schumacher HR Jr, Evans RR, Saag KG, Clower J, Jennings W, Weinstein SP, et al. Rilonacept (interleukin-1 trap) for prevention of gout flares during initiation of uric acid-lowering therapy: results from a phase III randomized, double blind, placebo-controlled, confirmatory efficacy study. Arthritis Care Res (Hoboken). 2012 Oct;64(10):1462-70.

18) Terkeltaub RA, Schumacher HR, Carter JD, Baraf HS, Evans RR, Wang J, at al. Rilonacept in the treatment of acute gouty arthritis: a randomized, controlled clinical trial using indomethacin as the active comparator. Arthritis Res Ther. 2013 Feb;15(1): R25.

19) Schlesinger N, Mysler E, Lin HY, De Meulemeester M, Rovensky J, Arulmani U, et al. Canakinumab reduces the risk of acute gouty arthritis flares during initiation of allopurinol

For peer review only

treatment: results of a double-blind, randomised study. Ann Rheum Dis. 2011 Jul;70(7):1264-71.

20) So A, De Meulemeester M, Pikhlak A, Yücel AE, Richard D, Murphy V, et al. Canakinumab for the treatment of acute flares in difficult-to-treat gouty arthritis: Results of a multicenter, phase II, dose-ranging study. Arthritis Rheum. 2010 Oct;62(10):3064-76.

21) Schlesinger N, De Meulemeester M, Pikhlak A, Yücel AE, Richard D, Murphy V, et al. Canakinumab relieves symptoms of acute flares and improves health-related quality of life in patients with difficult-to-treat Gouty Arthritis by suppressing inflammation: results of a randomized, dose-ranging study. Arthritis Res Ther. 2011 Mar 25;13(2): R53.

22) Schlesinger N, Alten RE, Bardin T, Schumacher HR, Bloch M, Gimona A, et al. Canakinumab for acute gouty arthritis in patients with limited treatment options: results from two randomised, multicentre, active-controlled, double blind trials and their initial extensions. Ann Rheum Dis. 2012 Nov;71(11):1839-48.

23) U.S. Food and Drug Administration [Internet]. Maryland, US. Arthritis Advisory Committee Meeting 2011: FDA briefing document. [cited 2016 Nov 01] Available from: http://www.fda.gov/downloads/AdvisoryCommittees/CommitteesMeetingMaterials/Drugs/ArthritisAdvisoryCommittee/UCM259596.pdf

24) Chakraborty A, Van LM, Skerjanec A, Floch D, Klein UR, Krammer G, et al. Pharmacokinetic and pharmacodynamic properties of canakinumab in patients with gouty arthritis. 2013 Dec;53(12):1240-51.

25) So A, De Smedt T, Revaz S, Tschopp J. A pilot study of IL-1 inhibition by anakinra in acute gout. Arthritis Res Ther. 2007;9(2): R28.

26) Chen K, Fields T, Mancuso CA, Bass AR, Vasanth L. Anakinra's efficacy is variable in refractory gout: report of ten cases. Semin Arthritis Rheum. 2010 Dec;40(3):210-4.

27) Ghosh P, Cho M, Rawat G, Simkin PA, Gardner GC, et al. Treatment of acute gouty arthritis in complex hospitalized patients with anakinra. Arthritis Care Res 2013 Aug;65(8):1381-4.

28) Ottaviani S, Moltó A, Ea HK, Neveu S, Gill G, Brunier L, et al. Efficacy of anakinra in gouty arthritis: a retrospective study of 40 cases. Arthritis Res Ther. 2013;15(5): R123.

29) Thueringer JT, Doll NK, Gertner E. Anakinra for the treatment of acute severe gout in critically ill patients. Semin Arthritis Rheum. 2015 Aug;45(1):81-5

For peer review only - http://bmjopen.bmj.com/site/about/guidelines.xhtml

30) Balasubramaniam G, Almond M, Dasgupta B. Improved renal function in diabetic patients with acute gout treated with anakinra. Kidney Int. 2015 Jul;88(1):195-6.

31) Direz G, Noël N, Guyot C, Toupance O, Salmon JH, Eschard JP. Efficacy but side effects of anakinra therapy for chronic refractory gout in a renal transplant recipient with pre-terminal chronic renal failure. Joint Bone Spine. 2012 Dec;79(6):631.

32) Yang B-B, Baughman S, Sullivan J. Pharmacokinetics of anakinra in subjects with different levels of renal function. Clin Pharmacol Ther. 2003 Jul;74(1):85-94.

33) Nuki G, Bresnihan B, Bear MB, et al. Long-term safety and maintenance of clinical improvement following treatment with anakinra (recombinant human interleukin-1 receptor antagonist) in patients with rheumatoid arthritis: extension phase of a randomized, double-blind, placebo controlled trial. Arthritis Rheum. 2002 Nov;46(11):2838-46.

34) Fleischmann RM, Schechtman J, Bennett R, Handel ML, Burmester GR, Tesser J, et al. Anakinra, a recombinant human interleukin-1 receptor antagonist (r-metHuIL-1ra), in patients with rheumatoid arthritis: A large, international, multicenter, placebo-controlled trial. Arthritis Rheum. 2003 Apr;48(4):927-34.

35) Schiff MH, DiVittorio G, Tesser J, Fleischmann R, Schechtman J, Hartman S, at al. The safety of anakinra in high-risk patients with active rheumatoid arthritis: six-month observations of patients with comorbid conditions. Arthritis Rheum. 2004 Jun;50(6):1752-60.

36) Fleischmann RM, Tesser J, Schiff MH, Schechtman J, Burmester GR, Bennett R, et al. Safety of extended treatment with anakinra in patients with rheumatoid arthritis. J Rheumatol. 2006 Feb;33(2):234-43.

37) Bresnihan B, Alvaro-Gracia JM, Cobby M, Doherty M, Domljan Z, Emery P, et al. Treatment of rheumatoid arthritis with recombinant human interleukin-1 receptor antagonist. Arthritis Rheum. 1998 Dec;41(12):2196-204.

38) European Medicines Agency [Internet]. London, UK. Annex 1 Summary of product characteristics. [cited 2016 Nov 01] Available from: http://www.ema.europa.eu/docs/en_GB/document_library/EPAR_-_Product_Information/human/000363/WC500042310.pdf

39) Hung AM, Ellis CD, Shintani A, Booker C, Ikizler TA. IL-1β receptor antagonist reduces inflammation in hemodialysis patients. J Am Soc Nephrol. 2011 Mar;22(3):437-42.

For peer review only - http://bmjopen.bmj.com/site/about/guidelines.xhtml

40) Fisher CJ Jr, Dhainaut JF, Opal SM, Pribble JP, Balk RA, Slotman GJ, et al. Recombinant human interleukin 1 receptor antagonist in the treatment of patients with sepsis syndrome. Results from a randomized, double-blind, placebo-controlled trial. Phase III rhIL-1ra Sepsis Syndrome Study Group. JAMA. 1994 Jun 15;271(23):1836-43.

41) Balasubramaniam G, Almond M, Borg F, Dasgupta B. Treatment of an Acute Gout Flare in Patients with Kidney Disease: A Questionnaire Survey. Rheumatology 2015 Apr; 54(suppl 1): i202-i203.

42) Pyne D, Ioannou Y, Mootoo R, Bhanji A. Intra-articular steroids in knee osteoarthritis: a comparative study of triamcinolone hexacetonide and methylprednisolone acetate. Clin Rheumatol. 2004 Apr;23(2):116-20.

43) Neogi T, Jansen TL, Dalbeth N, Fransen J, Schumacher HR, Berendsen D, et al. 2015 Gout Classification Criteria. An American College of Rheumatology/ European League Against Rheumatism Collaborative Initiative. Arthritis Rheum. 2015 Oct;67(10):2557-2568.

For peer review only

For peer review only - http://bmjopen.bmj.com/site/about/guidelines.xhtml

Tables

Box 1 ASGARD Inclusion criteria

Inclusion criteria

Patients who meet inclusion criteria will be approached by a study investigator. Consent for participation will be sought. Upon gaining consent, the investigator will go through exclusion criteria.

1. Subjects capable of giving informed consent.
2. Male or non-pregnant, non-nursing female
3. ≥ 18 years of age
4. eGFR < 60mls/min/1.73m^2 and ≥ 30mls/min/1.73m^2 as calculated using serum creatinine and modified MDRD formula or CKD-EPI, on two occasions at least two months apart with one being in the last six months. Creatinine at time of presentation can be used unless participant has an acute kidney injury as defined by serum creatinine rise by ≥ 26µmol/L within 48 hours or ≥ 1.5-fold rise from baseline value.
5. Diagnosis of acute gout arthritis as defined by the American College of Rheumatology 1977 preliminary criteria.
6. Gout attack less ≤ 36 hours
7. Baseline pain intensity greater than or equal to 50mm on the 0-100 mm VAS. In the case of multiple joints (≤ 3), the most affected joint will be assessed.

For peer review only

Box 2 ASGARD Exclusion criteria

Exclusion criteria

1. Treatment with colchicine in the last week.

2. Initiation of or change to dose of NSAIDS in the last week. NSAID use is not an exclusion criteria if taken at a stable dose for at least four weeks.

3. Initiation of or change to dose of systemic steroids in the last week. Systemic steroid use is no an exclusion criteria if taken at an equivalent dose of 10mg Prednisolone or less for the last four weeks.

4. Polyarticular gout, i.e. affecting four or more 4 joints

5. Rheumatoid arthritis, evidence/suspicion of infectious/septic arthritis, or other acute inflammatory arthritis such as systemic lupus erythematosus, mixed connective tissue disease, scleroderma, polymyositis, or significant systemic involvement secondary to rheumatoid arthritis.

6. Con-current immunosuppression/immunomodulatory treatment (Calcineurin inhibitor, anti-proliferative or biologic) therapy for other reason i.e. organ transplant.

7. Prior history or current inflammatory joint disease other than gout (e.g. rheumatoid arthritis (RA), reactive arthritis, psoriatic arthritis, seronegative spondyloarthropathy, Lyme disease).

8. Current active malignancy (with the exception of basal cell or squamous cell carcinoma of the skin, cervical intraepithelial neoplasia and non-metastatic/advanced prostate cancer).

9. Any patients with contra-indication to intramuscular injection such as coagulopathy or thrombocytopenia (Platelet count<100 x 109/L (100,000/mm^3)).

10. Abnormal liver function tests: Total bilirubin>upper limit of normal, Alanine aminotransferase (ALT) or Aspartate Aminotransferase (AST) >2 times upper limit of normal.

11. Haemoglobin < 85g/L (8.5 g/dL)

12. White blood cell (WBC) count<1.5 x 109/L (1000/mm3), absolute neutrophil count<1.5 x 10^9/L (1000/mm^3)

For peer review only - http://bmjopen.bmj.com/site/about/guidelines.xhtml

13. Evidence of serious active infection requiring intra-venous (IV) anti-biotic drug administration within last four weeks. Potential participants who were empirically treated with a course of intravenous antibiotics for a presumptive diagnosis of an infection may be eligible where the treating medical team has subsequently excluded an active infection and consequently discontinued antibiotic therapy at least four hours before randomisation. These potential participants can be discussed with the investigator where clarity is required.

14. Evidence of uncontrolled concomitant cardiovascular, nervous system, pulmonary (including obstructive pulmonary disease), hepatic, endocrine (including uncontrolled diabetes) or gastrointestinal disease. Potential participants who have active concomitant disease can only be eligible after discussion and agreement with the treating medical team.

15. Known positive hepatitis B virus surface antigen (HBsAg), hepatitis C (HCV) antibody or HIV.

16. Females of child bearing potential who are not willing to use highly effective birth control methods from the time of consent to one week after treatment discontinuation. Highly effective method of contraception (hormonal or barrier method of birth control; abstinence) consist of:

• Combined (estrogen and progestogen containing) hormonal contraception associated with inhibition of ovulation; oral, intravaginal, transdermal.

• Progestogen-only hormonal contraception associated with inhibition of ovulation; oral, injectable, implantable.

• Intrauterine device (IUD) or intrauterine hormone-releasing system (IUS)

• Bilateral tubal occlusion or vasectomised partner

• Sexual abstinence, defined as true abstinence when it is in line with the preferred and usual lifestyle of the participant. Periodic abstinence (e.g. calendar, ovulation, sympthothermal, post-ovulation) and withdrawal methods are not acceptable methods of contraception.

17. Men who are sexually active with a female partner who are not willing to use highly effective birth control methods from the time of consent to one week after treatment discontinuation.

18. Females of childbearing potential must have a negative pregnancy test (highly

For peer review only - http://bmjopen.bmj.com/site/about/guidelines.xhtml

sensitive urine or serum pregnancy test after a confirmed menstrual period) within 7 days prior to treatment initiation. Subjects are considered not of child bearing potential if they are surgically sterile (i.e. they have undergone a hysterectomy, bilateral tubal ligation, or bilateral oophorectomy) or they are postmenopausal.

19. Females must not be breastfeeding.

20. Patients who have had treatment as part of this trial cannot have repeat treatment for another flare as part of the trial.

21. Patients with allergies to excipients of IMPs: citric acid, anhydrous, sodium chloride, disodium edetate dehydrate, polysorbate 80, sodium hydroxide. Hypersensitivity to E. Coli derived proteins, egg proteins and soy proteins. Patients with a latex allergy are also not eligible as the inner needle cover of the pre-filled syringe contains dry natural rubber (a derivative of latex).

Legends

Figure 1. ASGARD study flowchart. Randomised participants will receive allocated treatment and placebo equivalent.

Figure 2. ASGARD participant timeline. Participants will be assessed by research team at Day 1 (randomisation), Day 2, Day 7 and 8 weeks.

For peer review only

For peer review only - http://bmjopen.bmj.com/site/about/guidelines.xhtml

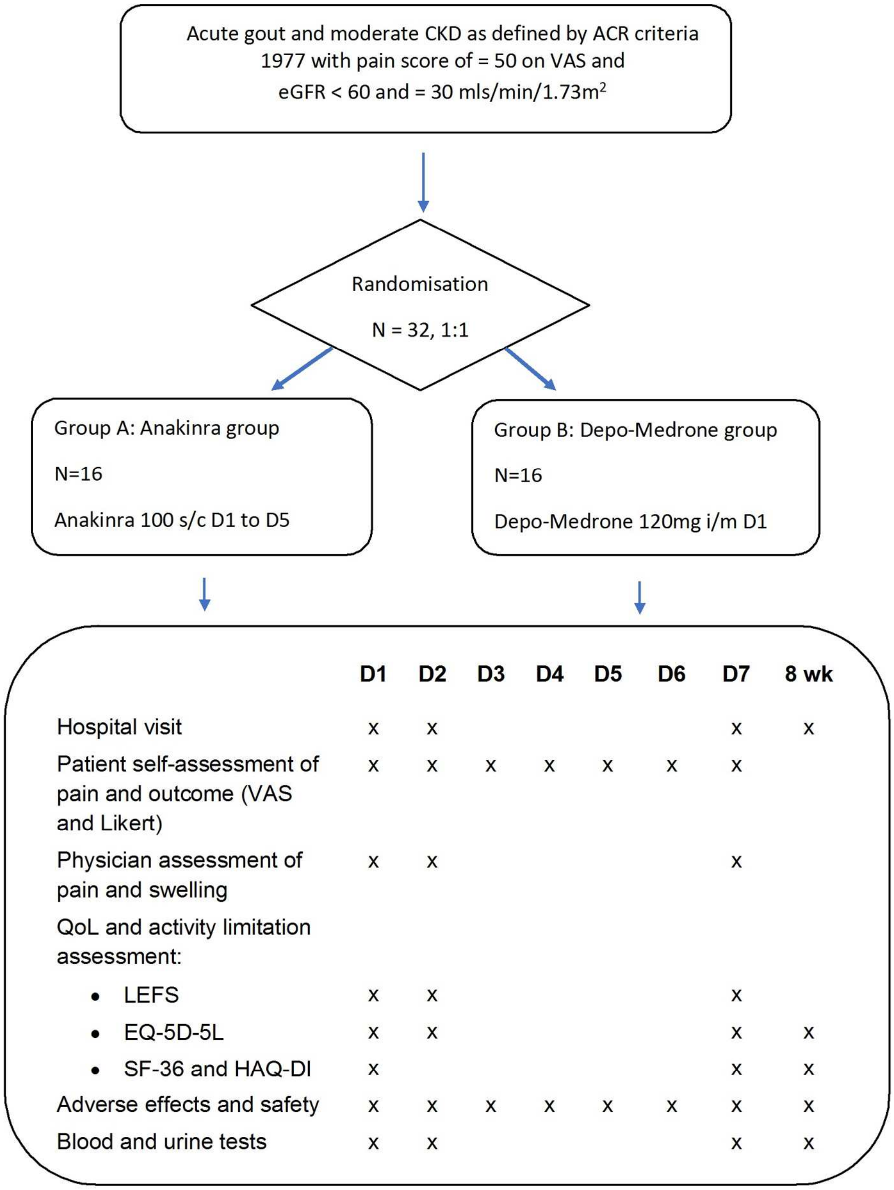

Trial flowchart

150x202mm (300 x 300 DPI)

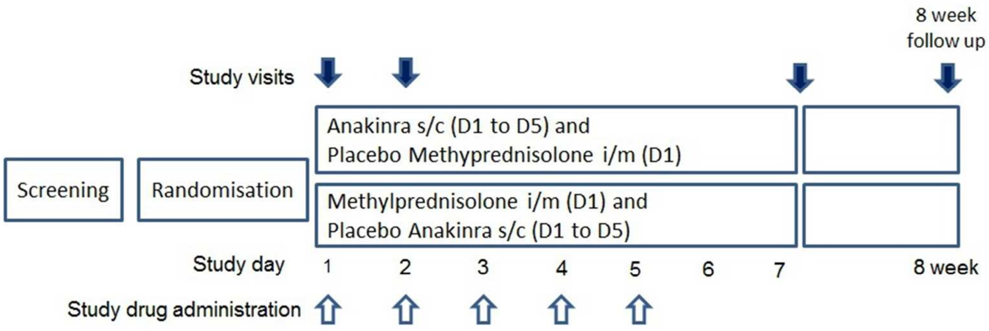

Participant timeline

76x42mm (300 x 300 DPI)

review only

Health Research Authority

CTIMP Protocol

- **This protocol has regard for the HRA guidance and order of content**

For peer review only

Version 1.4 February 2017

Health Research Authority

Anakinra vs. Steroids for the Treatment of Gout Attacks in Renal Patients (ASGARD): A Feasibility Study

EudraCT no. 2015-001787-19

A study to determine the feasibility of undertaking a definitive randomised multi-centre, double-blind, double-dummy controlled study of a novel agent Anakinra, an IL-1 receptor antagonist vs. intramuscular methylprednisolone (Depo-Medrone) for acute gout attacks in patients with moderate chronic kidney disease

For peer review only

For peer review only - http://bmjopen.bmj.com/site/about/guidelines.xhtml

Health Research Authority

Anakinra vs. Steroids for the Treatment of Gout Attacks in Renal Patients (ASGARD): A Feasibility Study

EudraCT no. 2015-001787-19

Anakinra vs. Steroids for the treatment of Gout Attacks in Patients with Renal Disease (ASGARD): A Feasibility study

Version 1.4

01 February 2017

RESEARCH REFERENCE NUMBERS

TRIAL REGISTRY NUMBER AND DATE

IRAS Number: 160630

EudraCT Number: 2015-001787-19

US NCT / Clinical trials.gov Number: NCT02578394

WHO Universal Trials Reference Number: U1111-1175-1977

SPONSORS Number: P0850

FUNDERS Number: NIHR GRANT PB-PG-0614-34090

For peer review only - http://bmjopen.bmj.com/site/about/guidelines.xhtml

Health Research Authority

Anakinra vs. Steroids for the Treatment of Gout Attacks in Renal Patients (ASGARD): A Feasibility Study

EudraCT no. 2015-001787-19

SPONSOR

SOUTHEND UNIVERSITY HOSPTAL NHS FOUNDATION TRUST

CONTACT ADDRESS:

PRITTLEWELL CHASE, WESTCLIFF-ON-SEA, SOUTHEND, ESSEX SS0 0RY

For peer review only

For peer review only - http://bmjopen.bmj.com/site/about/guidelines.xhtml

Health Research Authority

Anakinra vs. Steroids for the Treatment of Gout Attacks in Renal Patients (ASGARD): A Feasibility Study

EudraCT no. 2015-001787-19

SIGNATURE PAGE

The undersigned confirm that the following protocol has been agreed and accepted and that the Chief Investigator agrees to conduct the trial in compliance with the approved protocol and will adhere to the principles outlined in the Medicines for Human Use (Clinical Trials) Regulations 2004 (SI 2004/1031), amended regulations (SI 2006/1928) and any subsequent amendments of the clinical trial regulations, GCP guidelines, the Sponsor's SOPs, and other regulatory requirements as amended.

I agree to ensure that the confidential information contained in this document will not be used for any other purpose other than the evaluation or conduct of the clinical investigation without the prior written consent of the Sponsor

I also confirm that I will make the findings of the study publicly available through publication or other dissemination tools without any unnecessary delay and that an honest accurate and transparent account of the study will be given; and that any discrepancies from the study as planned in this protocol will be explained.

For and on behalf of the Study Sponsor:

Signature: .... Date: ....../....../......

Name (please print): ....

Position: ....

Chief Investigator:

Signature: .... Date: ....../....../......

Name: (please print): ....

Statistician:

For peer review only - http://bmjopen.bmj.com/site/about/guidelines.xhtml

NHS
Health Research Authority

Anakinra vs. Steroids for the Treatment of Gout Attacks in Renal Patients (ASGARD): A Feasibility Study

EudraCT no. 2015-001787-19

Signature:
……………………………………………………………………………………

Name: (please print):

……………………………………………………………………………………

Position:
……………………………………………………………………………………

KEY TRIAL CONTACTS

Chief Investigator	Gowrie Balasubramaniam<br>Consultant Nephrologist and General Physician<br>Southend University Hospital, Westcliff-on-Sea<br>Essex SS0 0RY<br>Phone: +44 7843563739 (mobile); 01702 435555<br>E-mail: gowrie@doctors.net.uk<br>g.balasubramaniam@southend.nhs.uk<br>Fax: 01702 508595
Trial Co-ordinator	Arit Udoh<br>Anglia Ruskin University<br>Post Graduate Medical Institute Clinical Trials Unit<br>Bishops Hall Lane, Chelmsford<br>Essex CM1 1SQ<br>Phone: 0845 196 4936<br>E-mail: trisha.parker@anglia.ac.uk
Sponsor	Craig MacKerness<br>Research and Development Manager<br>Southend University Hospital, Westcliff-on-Sea<br>Essex SS0 0RY<br>E-mail: Craig.Mackerness@southend.nhs.uk<br>Phone: 01702 385089<br>Fax: 01702 508595
Funder(s)	National Institute of Healthcare Research<br>Central Commissioning Facility

For peer review only - http://bmjopen.bmj.com/site/about/guidelines.xhtml

Health Research Authority

Anakinra vs. Steroids for the Treatment of Gout Attacks in Renal Patients (ASGARD): A Feasibility Study

EudraCT no. 2015-001787-19

	Grange House<br>15 Church Street<br>Twickenham TW1 3NL<br>Phone: 020 8843 8057<br>Email: rfpb@nihr-ccf.org.uk
Clinical Trials Unit	Anglia Ruskin University<br>Post Graduate Medical Institute Clinical Trials Unit<br>Bishops Hall Lane, Chelmsford<br>Essex CM1 1SQ<br>Phone: 0845 196 4936
Key Protocol Contributors	Gowrie Balasubramaniam<br>Consultant Nephrologist and General Physician<br>Southend University Hospital, Westcliff-on-Sea<br>Essex SS0 0RY<br>Phone: +44 7843563739 (mobile); 01702 435555 (work)<br>E-mail: gowrie@doctors.net.uk<br>g.balasubramaniam@southend.nhs.uk<br>Fax: 01702 508595<br><br>Bhaskar Dasgupta<br>Professor of Rheumatology<br>Southend University Hospital, Westcliff-on-Sea<br>Essex SS0 0RY<br>Phone: 01702 385856<br>E-mail: bhaskar.dasgupta@southend.nhs.uk<br>Fax: 01702 508595
Statistician	Jufen Zhang<br>Medical Statistician<br>Anglia Ruskin University<br>Post Graduate Medical Institute<br>Faculty of Health and Social Care<br>Bishops Hall Lane, Chelmsford<br>Essex CM1 1SQ<br>Phone: 0845 196 4936

For peer review only - http://bmjopen.bmj.com/site/about/guidelines.xhtml

Health Research Authority

Anakinra vs. Steroids for the Treatment of Gout Attacks in Renal Patients (ASGARD): A Feasibility Study

EudraCT no. 2015-001787-19

	E-mail: Jufen.Zhang@anglia.ac.uk
Health Economist	David Turner<br>Health Economist<br>Norwich Medical School<br>University of East Anglia<br>Norwich Research Park<br>Norwich, NR4 7TJ<br>Phone: 01603 593960<br>E-mail: David.A.Turner@uea.ac.uk
Trials pharmacists	Amiirah Ropun, Inisa Idress and Laura Wilson<br>Trial Pharmacists<br>Southend University Hospital, Westcliff-on-Sea<br>Essex SS0 0RY<br>Phone: 01702 435555<br>E-mail: Amiirah.Ropun@southend.nhs.uk; Inisa.Idress@southend.nhs.uk; Laura.Wilson@southend.nhs.uk<br>Fax: 01702 508595
Committees	ASGARD Steering Committee<br>Southend University Hospital, Westcliff-on-Sea<br>Essex SS0 0RY<br>Phone: 01702 385856<br>E-mail: asgardsteering@hotmail.com<br>Fax: 01702 508595<br>ASGARD Trial Management Committee<br>c/o Dr G Balasubramaniam<br>Southend University Hospital, Westcliff-on-Sea<br>Essex SS0 0RY<br>Phone: 01702 435555<br><br>ASGARD Patient Committee<br>Southend University Hospital, Westcliff-on-Sea<br>Essex SS0 0RY<br>Phone: 01702 435555<br>E-mail: asgardsteering@hotmail.com

For peer review only - http://bmjopen.bmj.com/site/about/guidelines.xhtml

Health Research Authority

Anakinra vs. Steroids for the Treatment of Gout Attacks in Renal Patients (ASGARD): A Feasibility Study

EudraCT no. 2015-001787-19

For peer review only

For peer review only - http://bmjopen.bmj.com/site/about/guidelines.xhtml

Health Research Authority

Anakinra vs. Steroids for the Treatment of Gout Attacks in Renal Patients (ASGARD): A Feasibility Study

EudraCT no. 2015-001787-19

TRIAL SUMMARY

Trial Title	A Multi-Centre, Randomised Controlled Trial of Anakinra vs. Steroids for Gout Attacks in patients with Moderate Renal Disease (ASGARD): a Feasibility Study	
Short title	Anakinra vs. Steroids for Gout Attacks in Patients with Kidney Disease (ASGARD): A Feasibility Study	
Internal reference no.	P0850	
Clinical Phase	Feasibility Study	
Trial Design	Multi-centre, Randomised Controlled Trail	
Trial Participants	Patients with Acute Gout Attacks and Moderate Kidney Disease (eGFR < 60 to ≥ 30)	
Planned Sample Size and accrual rate	32. 2 patients per month across all sites. Large centres should be able to recruit one case per months and smaller centre may recruit less frequently.	
Administration Schedule	Group A (Experimental)<br><br>Anakinra 100mg s/c injection D1 to D5 and placebo i/m methylprednisolone (Lipofundin) injection at D1	Group B (Active comparator)<br><br>Intramuscular methylprednisolone (Depo-Medrone) 120mg i/m injection at D1 and placebo Anakinra s/c D1 to D5
Treatment duration	5 days (D1 to D5)	
Follow up duration	8 weeks	
Planned Trial Period	22 months	
	Objectives	Outcome Measures
Primary	Feasibility for larger definitive trial and safety	Test recruitment, retention rates, willingness to be randomised, eligibility criteria, adherence / compliance rates, economic data, and scalability. Safety and quantitative assessment of study involvement.

For peer review only - http://bmjopen.bmj.com/site/about/guidelines.xhtml

Health Research Authority

Anakinra vs. Steroids for the Treatment of Gout Attacks in Renal Patients (ASGARD): A Feasibility Study

EudraCT no. 2015-001787-19

Secondary	Proposed measures to comparing effectiveness of treatment arms	Time to 50% reduction and complete resolution of pain (self-assessment) on VAS (0-100mm) and 5-point Likert scale (D1 to D7)<br><br>Physician assessment of joint tenderness and swelling (D1, D2, and D7)<br><br>Patient reported outcome measure (D1 to D7).<br><br>Patient assessment of activity limitation and quality of life: HAQ-DI and SF-36 (D1, D7 and 8 weeks);<br><br>EQ-5D-5L at D1, D2, D7 and 8 weeks;<br><br>LEFS at D1, D2, D7.<br><br>Time to take rescue medication<br><br>Change in renal function by eGFR, CRP, uric acid and urine uric acid (D1, D2 , D7, week 8)<br><br>Healthcare resource use at 8 weeks.
Tertiary (exploratory)	Inflammatory changes in blood and urine	Changes in blood and urine inflammatory changes markers. Epigenetic changes with treatment (D1, D2, and D7, week 8).
Investigational Medicinal Product(s)	Anakinra, Interleukin-1 receptor antagonist<br><br>Intramuscular methylprednisolone (Depo-Medrone)<br><br>Lipofundin MCT/LCT 10% (placebo for Intramuscular methylprednisolone)	
Formulation, Dose, Route of Administration	Anakinra: Pre-filled syringes 0.67mLs, 100mg s/c for 5 days	

For peer review only - http://bmjopen.bmj.com/site/about/guidelines.xhtml

Health Research Authority

Anakinra vs. Steroids for the Treatment of Gout Attacks in Renal Patients (ASGARD): A Feasibility Study

EudraCT no. 2015-001787-19

	Intramuscular methylprednisolone acetate (Depo-Medrone): 3mL injection containing 120mg/3mL drawn from 3mL vial.<br><br>Lipofundin MCT/LCT 10%: 3mL injection drawn from 100mL bottle for i/m injection

FUNDING AND SUPPORT IN KIND

**FUNDER(S)**<br><br>(Names and contact details of ALL organisations providing funding and/or support in kind for this trial)	**FINANCIAL AND NON FINANCIALSUPPORT GIVEN**
NIHR	£157,591.00
SOBI	Provision of Anakinra and equivalent placebo<br><br>£10,000

For peer review only - http://bmjopen.bmj.com/site/about/guidelines.xhtml

Health Research Authority

Anakinra vs. Steroids for the Treatment of Gout Attacks in Renal Patients (ASGARD): A Feasibility Study

EudraCT no. 2015-001787-19

ROLE OF STUDY SPONSOR AND FUNDER

The sponsor of this clinical trial is Southend University Hospital Trust. The named representative of the sponsor is the head of Research and Development. The sponsor is responsible for:

- Providing the investigators with information needed to conduct the trial properly, access to resources and support to deliver the research as proposed.
- Ensure monitoring of the trial, in conjunction with chief-investigator, the trial co-ordinator and the trial steering committee.
- Ensure that the investigation is conducted in accordance with Health Research Authority and the framework of research governance of the Department of Health and Good Medical Practice.

The sponsor has to be satisfied there is agreement on appropriate arrangement to:

- Record, report and review significant developments as the research proceeds, particularly those which outline the safety of individuals at risk; and to
- Approve any modifications to the design, obtain any regulatory authority required, implement them, and make them known.
- Patient withdrawal

The sponsor will undertake pharmacovigilance for this study. The sponsor will monitor adverse event monitoring and report at the end of the study back to the manufacturer. This is part of the clinical trial and will be published as an outcome as well.

Indemnity will be part of NHS indemnity of local centres. This is a phase 3 trial and a comparison of two treatments.

The sponsor has control over the final decision regarding the trial design, conduct, data analysis and interpretation, manuscript writing, and dissemination of results.

Role of the Funder

The funder has provided finance for the project. Reports will be made at regular intervals from the steering committee for the funder. The funder will also monitor and assess value for money for the project. Funding was approved after competitive, peer reviewed process.

For peer review only - http://bmjopen.bmj.com/site/about/guidelines.xhtml

ROLES AND RESPONSIBILITIES OF TRIAL MANAGEMENT COMMITEES/GROUPS & INDIVIDUALS

Trial Steering Committee

The Trial Steering Committee (TSC) will consist of the CI, trial co-ordinator and funding co-applicants. Members of patient group will bring independent representation. The TSG will take on some aspects of pharmacovigilance as there will be no data monitoring committee as part of this feasibility study.

Trial Management Group

The Trial Management Group (TMG) will consist of the CI, co-CI, funding co-applicants, representative of the clinical trials units including trial co-ordinator, data manager and statistician. They should meet every two months two to ensure all practical details of the trial are progressing well and adequate targets are met.

Data Monitoring Committee

This is a small feasibility study with a short duration and short follow-up. Pharmacovigilance is a component of the study but costs of running a data monitoring committee have to offset against the benefit in such a small feasibility trial, and also the minimal expected risk from previous published data where Anakinra has been used in patients with rheumatoid arthritis. Some roles of a data monitoring committee will be incorporated into the remit of the TMG. If a subsequent larger study is planned, then a data monitoring committee will be incorporated into the study.

ASGARD Patient group

The patient group consists of patients (and family) from Southend University hospital that suffer from chronic kidney disease and acute gout attacks.

For peer review only

Protocol contributors

This study was instigated due to increasing difficulty in managing the described patient population in routine clinical practice of the chief investigator. The protocol was mainly written by the chief investigator with Professor Dasgupta, who will act as co-chief investigator. The co-investigators, Drs Almond and Harnett gave input on study design. Michael Parker is the trial statistician and has contributed to relevant aspects of the protocol. David Turner is the Health Economist and has contributed to relevant aspects of the trial protocol. The clinical trial unit, including the director, data manager and the trial co-ordinator have given input into practical aspects of the trial design.

The gout study patient group has had an input in the design of the trial and members will continue to be part of the steering committee

The initial version of the protocol was rejected by the funding panel. Comments from reviewers were incorporated into the revised protocol. The funder has no control over any final decisions regarding the trial.

KEY WORDS:

Acute, Gout, Kidney, Intramuscular Methylprednisolone, Interleukin-1, Randomised-Controlled Trial

For peer review only - http://bmjopen.bmj.com/site/about/guidelines.xhtml

LIST of CONTENTS

GENERAL INFORMATION	Page No.
TITLE PAGE	2
RESEARCH REFERENCE NUMBERS	3
SIGNATURE PAGE	5
KEY TRIAL CONTACTS	6
TRIAL SUMMARY	10
FUNDING	11
ROLE OF SPONSOR AND FUNDER	12
ROLES & RESPONSIBILITIES OF TRIAL MANAGEMENT COMMITTEES, GROUPS AND INDIVIDUALS	13
LIST of CONTENTS	15
LIST OF ABBREVIATIONS	17
TRIAL FLOW CHART	19
**SECTION**	
1. BACKGROUND	22
2. RATIONALE	24
3. OBJECTIVES AND OUTCOME MEASURES/ENDPOINTS	26
4. TRIAL DESIGN	30
5. STUDY SETTING	30
6. ELIGIBILITY CRITERIA	30
7. TRIAL PROCEDURES	33
8. TRIAL MEDICATION	44

For peer review only - http://bmjopen.bmj.com/site/about/guidelines.xhtml

Health Research Authority

Anakinra vs. Steroids for the Treatment of Gout Attacks in Renal Patients (ASGARD): A Feasibility Study

EudraCT no. 2015-001787-19

9. PHARMACOVIGILANCE	60
10. STATISTICS AND DATA ANALYSIS	68
11. DATA HANDLING	72
12. MONITORING, AUDIT & INSPECTION	75
13. ETHICAL AND TRIAL ADMINISTRATION	77
14. DISSEMINATION POLICY	81
15. REFERENCES	82
16. APPENDICIES	85

For peer review only - http://bmjopen.bmj.com/site/about/guidelines.xhtml

Health Research Authority

Anakinra vs. Steroids for the Treatment of Gout Attacks in Renal Patients (ASGARD): A Feasibility Study

EudraCT no. 2015-001787-19

LIST OF ABBREVIATIONS

Define all unusual or 'technical' terms related to the trial. Add or delete as appropriate to your trial. Maintain alphabetical order for ease of reference.

AE	Adverse Event
AR	Adverse Reaction
CA	Competent Authority
CI	Chief Investigator
CKD	Chronic Kidney Disease
CRF	Case Report Form
CRO	Contract Research Organisation
CTA	Clinical Trial Authorisation
CTIMP	Clinical Trial of Investigational Medicinal Product
DMC	Data Monitoring Committee
DSUR	Development Safety Update Report
EC	European Commission
EMEA	European Medicines Agency
EQ-5D-5L	EuroQol-5 Dimension-5 Level
EU	European Union
EUCTD	European Clinical Trials Directive
EudraCT	European Clinical Trials Database
EudraVIGILANCE	European database for Pharmacovigilance
GC	Glucocorticoid
GCP	Good Clinical Practice
GMP	Good Manufacturing Practice
HAQ-DI	Health Assessment Questionnaire Disability Index
IB	Investigator Brochure
ICF	Informed Consent Form
ICH	International Conference on Harmonisation of technical requirements for registration of pharmaceuticals for human use.
IDMC	Independent Data Monitoring Committee
IL-1	Interleukin-1
IL-1 RA	Interleukin-1 Receptor Antagonist
IMP	Investigational Medicinal Product
IMPD	Investigational Medicinal Product Dossier
ISF	Investigator Site File

For peer review only - http://bmjopen.bmj.com/site/about/guidelines.xhtml

Health Research Authority

Anakinra vs. Steroids for the Treatment of Gout Attacks in Renal Patients (ASGARD): A Feasibility Study

EudraCT no. 2015-001787-19

ISRCTN	International Standard Randomised Controlled Trials Number
LEFS	Lower Extremity Functional Scale
MA	Marketing Authorisation
MHRA	Medicines and Healthcare products Regulatory Agency
MS	Member State
NHS R&D	National Health Service Research & Development
NIMP	Non-Investigational Medicinal Product
PI	Principal Investigator
PIC	Participant Identification Centre
PIS	Participant Information Sheet
QA	Quality Assurance
QC	Quality Control
QoL	Quality of Life
QP	Qualified Person
RCT	Randomised Control Trial
REC	Research Ethics Committee
SAE	Serious Adverse Event
SAR	Serious Adverse Reaction
SDV	Source Data Verification
SF 36	Short Form (36) Health Survey
SOP	Standard Operating Procedure
SmPC	Summary of Product Characteristics
SSI	Site Specific Information
SUSAR	Suspected Unexpected Serious Adverse Reaction
TMG	Trial Management Group
TSC	Trial Steering Committee
TMF	Trial Master File
VAS	Visual Analogue Scale

For peer review only

For peer review only - http://bmjopen.bmj.com/site/about/guidelines.xhtml

TRIAL FLOW CHART

Acute gout and moderate CKD as defined by ACR criteria 1977 with pain score of ≥ 50 on VAS and eGFR < 60 and ≥ 30 mls/min/1.73m^2

↓

Randomisation

N = 32, 1:1

Group A: Anakinra group. N=16

Anakinra 100mg s/c D1 to D5

Placebo i/m D1

Group B: Methylprednisolone group. N=16

Methylprednisolone (Depo-Medrone) 120mg i/m D1

Placebo s/c D1 to D5

For peer review only - http://bmjopen.bmj.com/site/about/guidelines.xhtml

Health Research Authority

Anakinra vs. Steroids for the Treatment of Gout Attacks in Renal Patients (ASGARD): A Feasibility Study

EudraCT no. 2015-001787-19

	D1	D2	D3	D4	D5	D6	D7	8 wk
Hospital visit	x	x					x	x
Patient self-assessment of pain and outcome (VAS and Likert)	x	x	x	x	x	x	x	
Physician assessment of pain and swelling	x	x					x	
QoL and activity limitation assessment:								
• LEFS	x	x					x	
• EQ-5D-5L	x	x					x	x
• SF-36 and HAQ-DI	x						x	x
Adverse effects and safety	x	x	x	x	x	x	x	x
Blood and urine tests	x	x					x	x
Interview, N=12								x
[illegible]								

SCHEDULE OF PROCEDURES

Procedures	Screening	Baseline (pre-treatment)<br>Day 1	Day 2	Day 3	Day 4	Day 5	Day 6	Day 7	8 weeks
		Treatment phase							
			Follow up						
Informed Consent	X								
Demographics	X								
Medical History	X								
Physical examination	X								
Vital signs	X								
Blood and urine tests		X	X					X	X

For peer review only - http://bmjopen.bmj.com/site/about/guidelines.xhtml

Concomitant medications	X	X	X	X	X	X	X	X	
Eligibility assessment	X								
Randomisation		X							
Dispensing of trial drug		X							
Drug administration		X	X	X	X	X			
Patient reported pain and outcome assessments (diary entry)		X	X	X	X	X	X	X	
Physician assessment of joint		X	X					X	
Patient assessment of activity limitation and quality of life using:									
• LEFS		X	X					X	
• EQ-5D-5L		X	X					X	X
• HAQ-DI and SF-36		X						X	X
Compliance Assessments			X	X	X	X	X	X	
Checking patient diary entry for time of injection and pain assessment (by phone or face to face)			X	X	X	X	X	X	
Diary return								X	
Check injection sites		X	X					X	

For peer review only

For peer review only - http://bmjopen.bmj.com/site/about/guidelines.xhtml

Check returned syringes								X	
Questionnaire return (face to face or by mail)									X
Adverse events assessments (face to face or by phone)		X	X	X	X	X	X	X	X
Physician's withdrawal checklist									
Interview (12 patients)									X
Healthcare Resource use data									X

For peer review only

STUDY PROTOCOL

A study to determine the feasibility of undertaking a definitive randomised multi-centre, double-blind, double-dummy controlled study of a novel agent Anakinra, an IL-1 receptor antagonist vs. intramuscular methylprednisolone (Depo-Medrone) for acute gout attacks in patients with moderate chronic kidney disease.

1 BACKGROUND

Chronic Kidney Disease and Acute Gout

Chronic kidney disease (CKD) affects 5% of the UK population[1] and 40% of patients with CKD 3 and 4 have chronic gout[2], suggesting that 1.32 million people, predominantly older patients, have CKD and gout in the UK.

In a UK study, approximately 89.4% of patients were treated for their acute flare with non-steroidal anti-inflammatory agents (NSAIDS) which are well known to worsen kidney failure[3]. Data from secondary care showed that up to 50% of acute gout patients had CKD and 40% were given contra-indicated medications[4]. This is similar to our experience where an audit of acute gout showed half of

Health Research Authority

Anakinra vs. Steroids for the Treatment of Gout Attacks in Renal Patients (ASGARD): A Feasibility Study

EudraCT no. 2015-001787-19

all patients with acute gout and kidney disease were given potentially harmful medications. Mismanagement of acute gout attacks in patient with kidney disease occurs as there is no firm evidence-base[5].

Acute Gout

The incidence of acute gout attacks is 2.68 per 1000 person-years[6]. The prevalence of gout is increasing due to increasing prevalence of co-morbid conditions that are associated with hyperuricaemia such as hypertension, obesity, metabolic syndrome, diabetes and chronic kidney disease.

The lack of evidence in the literature for the use of conventional agents in patients with CKD is primarily because patients with CKD are excluded as NSAIDS are used as an active comparator in randomised controlled trials. Our survey showed that most rheumatologists in our region felt guidance in treating CKD patients is lacking (BRS poster 2015). Some guidelines continue to suggest using NSAIDS and colchicine with caution in patients with kidney disease even with a poor evidence base[7,8,9].

NSAIDS have the largest evidence base for use in acute gout but are contra-indicated in CKD. They can also worsen heart failure, hypertension, and liver failure and cause gastro-intestinal bleeding[5,10]. Colchicine is an alternative anti-inflammatory agent but all clinical trials excluded patients with moderate-to-severe CKD. It has a narrow therapeutic index, even though lower doses of colchicine can be efficacious (AGREE trial), 20% of patients still experienced diarrhoea[11]. Diarrhoea is common in patients with CKD and they are also more likely to have drug accumulation and serious side effects from toxicity such as bone marrow failure, rhabdomyolysis, pancreatitis and myopathy[5].

Glucocorticoids are an effective treatment with some evidence for use in CKD patients from older trials[12]. Triamcinolone, a long-acting synthetic corticosteroid, was shown to be as effective as adrenocorticotrophic hormone (ACTH), a hormone that increases endogenous cortisol secretion[10]. Triamcinolone is commonly used as the active comparator agent in contemporary acute gout studies world-wide.

We are using intramuscular methylprednisolone (depo-medrone) as it is the most commonly used long-acting intra-muscular agent in the UK (suggested by reviewer of initial application and from our survey). It is equivalent to Triamcinolone in other inflammatory joint conditions[13] and is the preferred UK treatment as evidence by our regional survey of Rheumatologists (presented at BRS 2015). It has been shown to be useful in the initial phase of treating rheumatoid arthritis[14] but there were concerns about long-term side-effects[15]. Intramuscular methylprednisolone is as efficacious as oral steroids with a lower cumulative dose (120mg vs.175mg) in other rheumatology conditions, and would be equivalent to the dose used in the Janssens paper[12]. This well design double-blind randomised controlled study showed that Prednisolone 35mg for 5 days was not inferior to Naproxen, although, this study recruited from primary care and excluded patients with impaired renal function (mean participant GFR was 78). Intramuscular methylprednisolone is also associated with less mineralocorticoid effect reducing the likelihood of salt and water retention in heart failure and renal failure.

The IMPs

- Anakinra (Kineret)

For peer review only - http://bmjopen.bmj.com/site/about/guidelines.xhtml

The investigational medical product is Anakinra (Kineret), an IL-1 receptor antagonist (IL-1 RA) that competes with IL-1beta for its receptor (IL-1R) and thereby preventing its action[16]. It is a recombinant, non-glycosylated form of naturally occurring human IL-1RA. It comes in 1mL pre-filled syringes containing 100mg of Anakinra in solution containing sodium citrate, sodium chloride and disodium EDTA and polysorbate in water for injection.

A pilot study of its use in gout showed prompt resolution of symptoms in patient's treated with anakinra[17], including in three patients with renal impairment. There have been further reports of the use of Anakinra in difficult to treat cases[18-22]. All these studies show good efficacy with the majority of patients having a good response within 24 hours. There was one case report of neutropenia in one patient with a kidney transplant with severe renal impairment.[23]

Anakinra has a lot of evidence in the use of rheumatoid arthritis as a biological response modifier. Large, multi-national placebo-controlled trials did not show a significantly increased risk of infection or major adverse events[24-26]. It was well tolerated in the long-term, even up to three years[27]. Anakinra has also been shown to be beneficial in studies looking at diabetes[28] and acute heart failure[29].

Anakinra has US FDA approval for use in rheumatoid arthritis. It is currently not recommended by NICE (February 2009, NICE) for rheumatoid arthritis except in a controlled clinical study. Anakinra is currently not licenced for use in the treatment of acute gout.

We want to explore the use of Anakinra for acute gout in patients with moderate renal impairment in comparison to the current standard therapy of steroids. We feel that IL-1RA inhibition provides a very specific treatment option with less side-effect. This is a timely study that aims to establish a firm evidence base for the management of a relatively common condition in a high risk group that is associated with considerable morbidity and costs.

- Intramuscular methylprednisolone acetate (Depo-Medrone)

Intramuscular methylprednisolone acetate is the commonest depot steroid formulation used in the UK. Intramuscular methylprednisolone 120mg provides the equivalent dose of steroid used in a study comparing steroids vs. NSAIDS (i.e. 35 mg for 5 days). It is a CTIMP in this study as, even though it is commonly used, it has not been used in a clinical study. The rate of side-effects even with short-term steroid treatment in acute gout is also unknown.

- Lipofundin MCT/LCT (intramuscular methylprednisolone placebo)

Lipofundin is a lipid emulsion for injection that is routinely used to provide nutritional support of calories and essential fatty acids. Depot steroid injection has to be prepared in a lipid emulsion, similar to Lipofundin. It has been used as an equivalent placebo to Triamcinolone in contemporary studies comparing Canakinumab, another IL-1 inhibitor, vs. Triamcinolone[30]. It has an important role in the design of the study in that it maintains a double-blind aspect of the design. This would not be possible is 0.9 Saline was used. It is being used as a CTIMP after discussion with the MHRA as it is not normally used for this purpose.

2 RATIONALE

This project was initially conceived because nephrologists still see patients who have chronic kidney disease or acute kidney injury in patients with gout due to treatment with NSAIDS. The other

For peer review only - http://bmjopen.bmj.com/site/about/guidelines.xhtml

potentially harmful agent that is widely used without any evidence base in patients with chronic kidney disease is colchicine. These patients usually develop diarrhoea, which is an early sign of toxicity. The patient group felt strongly that protecting renal function from further injury should be a priority for any clinician. This reduces the increased mortality and risk of dialysis associated with deterioration in renal function. IL-1 signalling has been shown to play a key role in gout associated inflammation[31] and targeted therapy is now available with the increasing numbers if IL-1 inhibitors.

Two such inhibitors of IL-1 have been used in randomised controlled trials against the only other evidence-based treatment option of steroids. Although they showed efficacy, FDA approval was not granted due to safety concerns with these agents.[32] Rilonocept, an IL-1 decoy receptor, has been shown to be efficacious in reducing acute flares during the initiation of urate lowering therapy[33,34] but no added benefit was obtained for the treatment of acute gout when it was used in addition to an NSAID[35]. Canakinumab, a monoclonal antibody against IL-1 beta showed efficacy preventing acute flares during the initiation of urate lowering therapy, and for acute gout in patients with difficult disease or contra-indication to colchicine or NSAIDs[30,36,37,38]. The adverse event rate was considered too high by the FDA to approve its use for acute gout attacks.

Anakinra is a recombinant human interleukin-1 receptor antagonist with proven efficacy in acute gout. It is increasingly being used in patients with co-morbidities along with patients with other conditions such as diabetes and heart disease. Anakinra also offer advantages over other agents due to:

- Pharmacokinetics: Anakinra has been used in patients with severe renal disease with a modified regime, including patients on haemodialysis[39]. The prolonged action of Canakinumab with its irreversibility increases the risk of serious adverse events[40].
- Safety: Anakinra has excellent long-term safety profile from previous studies in Rheumatoid arthritis which consisted of regimes requiring daily injections for up to three years in patients with multiple co-morbidities[41].
- Cost: Anakinra 5 injections £131.15 vs. Canakinumab £9927.90.

The use of intramuscular methylprednisolone will also help establish some data for the use of corticosteroids for acute gout in patients with renal disease. If the initial results show equivalent efficacy between the two treatment arms, then we could go on to establish steroids as the standard treatments and making considerable savings.

In summary, there is poor evidence for the treatment of acute gout in patients with CKD. This is not an ideal situation as the number of patients with CKD is increasing, and a substantial proportion of patients with gout have CKD. Glucocorticoids, even with their poor evidence base, form the mainstay of treatment for acute gout in patients with kidney disease. With a safe, novel agent now available, we feel this is a timely study exploring a novel specific treatment option for an increasing group of patients where no routinely safe option is available. We hope to go on to undertake a definitive study that would enable guidelines to be updated for this specific patient group. A double-blind, double dummy study will add experimental rigour to the study and will help add to much needed overall evidence base.

2.1 Assessment and Management of risk

Anakinra has been used to try and treat people with Rheumatoid Arthritis, where a lot of patients were already on treatments that would suppress their immune system. The treatment was for many months (up to six months) and there was a slightly higher risk of infection (2%) and neutropenia in people who had also been on Anakinra.

For peer review only - http://bmjopen.bmj.com/site/about/guidelines.xhtml

NHS Health Research Authority

Anakinra vs. Steroids for the Treatment of Gout Attacks in Renal Patients (ASGARD): A Feasibility Study

EudraCT no. 2015-001787-19

Another agent called Canakinumab, that works similarly to Anakinra but is very long acting and lasts in the body for many weeks, has also been shown to have a higher risk of infection and neutropenia when this agent was used to treat gout. It was felt that this risk did not weigh up against the benefit of the treatment and so it is advised to use with caution and not readily recommended for the treatment of gout attacks.

Anakinra may be in the best position for treating gout attacks. The treatment time will be short, five days versus many months, and we feel the risk of infection should be much less. We know from the studies in people with Rheumatoid Arthritis, it was well tolerated by people. It also doesn't last in the body for many days so we do not anticipate the same complications as with Canakinumab. If a participant develops an infection or neutropenia, we can stop treatment and it should come out of the system within days, unlike Canakinumab which can last for weeks.

Anakinra has already been shown to be effective for treating gout, even when used in patients with multiple medical problems and active infections. Anakinra was first used in a clinical setting in people with severe septicaemia and those who received Anakinra were not worse off.

To minimise this potential risk, we are excluding patients with serious active infections requiring hospital admission and intra-venous (IV) anti-biotic drug administration. We are also excluding people with an active viral infection and people who may be receiving other treatment that can compromise their immune system. We are also excluding people with low white blood cell counts at the start of the study.

The initial use of Anakinra was in sepsis and it was not associated with a higher risk of mortality. Our protocol will exclude patients with uncontrolled disease including infections. We will check bloods at the end of the course of 5 days treatment to ensure that blood counts are stable. Patients will also be assessed at Day 2, Day 7 and 8 weeks, patients will be asked to stop treatment if serious infection develops. There will be daily contact by phone on weekdays for the duration of treatment.

As with any treatment for acute gout, there is always a risk that a flare will occur at the end of treatment. This has been described in a few patients from the literature. Concurrent treatment with prophylaxis is an area of on-going study in itself, especially in patients with renal disease who are prone to reactions to allopurinol. Guidance on when to start appropriate prophylaxis use is lacking. Some studies suggest prolonged courses of low dose colchicine which, risks accumulation and some suggest starting allopurinol. We will obtain data on flares after treatment from the 8-week follow-up and possible plan a subsequent study looking at long-term prophylaxis.

We have intentionally avoided using Anakinra in patients with severe renal failure (eGFR < 30mls/min/1.73m^2) due to concerns about accumulation in pharmacokinetic studies. We are taking caution even though it has been used safely in these patients and we have some experience in our centre. It has also been described in patients on haemodialysis using an alternate day regime where no adverse complications were noted.

All the above risks are slightly higher that the risks of standard medical care. This also has to be offset against the risk associated with standard care of steroids which can cause uncontrolled diabetes, worsening salt and water retention in heart failure and kidney disease and a possible infection risk as well. Furthermore, we are aware that most patients even in secondary care are given agents that pose harm to them. Evidence suggests that Anakinra may be beneficial in patients with diabetes, heart failure and possibly in patients with diabetic kidney disease as well.

2.2 Rationale for dose and dose regimen

For peer review only - http://bmjopen.bmj.com/site/about/guidelines.xhtml

Health Research Authority

Anakinra vs. Steroids for the Treatment of Gout Attacks in Renal Patients (ASGARD): A Feasibility Study

EudraCT no. 2015-001787-19

The rationale for the use of 100mg s/c for 5 days come from emerging data where Anakinra has been used to treat acute gout. Some reports vary between 3 to 5 days but most use 5 days' duration of treatment to ensure adequate response in all patients[17-23]. This includes our own experience[23] where we have used 5 days' duration of treatment to have a standard approach to all patients, exclude patients with severe kidney disease (eGFR <30 – an alternate day injection regime is used).

The 100mg dose in part reflects experience from the use on Anakinra in studies to treat Rheumatoid Arthritis where most studies used a dose on 100mg but some studies used a range of 50mg to 100mg. Dosing in Cryopryin Associated Periodic Syndrome can be up to 3mg – 4mg per kg in severe disease (doses up to 800mg). We cannot use too high a dose due to the risk of accumulation in patients with chronic kidney disease. 100mg has been used in the patients with good efficacy and in patients with CKD without too many adverse events[17-23].

3 OBJECTIVES AND OUTCOME MEASURES/ENDPOINTS

3.1 Primary objective

The aim of this study is to determine the feasibility of running a phase III double-blind, double-dummy randomised controlled trial comparing intramuscular methylprednisolone (Depo-Medrone) 120mg vs. Anakinra 100mg subcutaneous injection for 5 days for the treatment of acute gout attacks in patients with chronic kidney disease as defined by a eGFR < 60mls/min/1.73m^2 and ≥ 30mls/min/1.73m^2.

The population to be studied are patients with moderate kidney disease and acute gout. There is increasing prevalence of these patients in the population due to an older population and the presence of con-current disease such as diabetes, atherosclerotic disease and heart disease.

The intervention is the treatment for the acute gout attack, which will be Anakinra. This is a target specific agent, in 100mg pre-filled injection for 5 days.

There is not standard comparison per se as evidence is lacking in this patient group for this condition but steroids have the best evidence and the commonest agent prescribed in the UK is intramuscular methylprednisolone acetate (Depo-Medrone).

The primary outcome of interest is feasibility to undertake a definitive randomised controlled trial and safety outcome assessment. Proposed outcome measure for subsequent definitive studies will look at effectiveness of treatment with regards to resolution of symptoms.

The time to assess outcome from a resolution of symptoms would be for 7 days with one final assessment at 8 weeks.

3.2 Secondary objectives

We will test the feasibility of using our primary outcomes measures of effectiveness from day 1 to day 7. These domains are suggested by the OMERACT guidelines, an international panel of experts and patients looking at outcome measures in gout. A significant component of the larger study will be a health economic evaluation to compare the incremental cost-effectiveness of Anakinra to the comparator treatment, steroids. Blood and urine tests for resolution monitoring of full blood count, kidney function and markers of inflammation. Complications such as infection, uncontrolled blood sugars and salt and water retention will be monitored.

3.3 Outcome measures/endpoints

For peer review only - http://bmjopen.bmj.com/site/about/guidelines.xhtml

This is a feasibility study. It is not powered to look for inference. Study process will be analysed. Recruitment and retention rates, and willingness of patients to be randomised will be calculated. Proportion of patients who did not meet eligibility criteria will be looked at. Adherence and compliance rates, and qualitative feedback will be examined. Economic data on healthcare resource use and health-related quality of life will be collected to inform the design of a subsequent larger definitive study. Key drivers of costs, resource allocation and scalability will also be determined. A significant component of the larger study will be a health economic evaluation to compare the incremental cost-effectiveness of Anakinra to the comparator treatment. Safety outcome measures will be reported as mandated in a CTIMP protocol.

Criteria for success

- Recruit approximately one subject every two weeks - target of 32
- At least 70% of eligible patients can be recruited
- At least 85% of all recruited patients complete 5 out of 7 patient reported pain outcome (proposed primary outcome measure for subsequent study)
- No more than 10% treatment crossover, or failure as defined by the use of additional agents such colchicine or further doses of steroids.

3.4 Primary endpoint/outcome

Feasibility of undertaking a definitive multicentre, double-blind, double dummy randomised controlled trial to obtain clear guidance on the safe management of acute gout attacks in patients with chronic kidney disease.

3.5 Secondary endpoints/outcomes

Proposed primary outcome measures of effectiveness consist of resolution of pain i.e. time to 50% reduction and complete resolution of pain in self-assessed pain intensity in the joint most affected at baseline measure on the VAS (0-100mm) and 5-point Likert scale from baseline to 7 days post randomisation (Day 1, Day 2, Day 3, Day 4, Day 5, Day 6, Day 7) using composite time points kept to intervals 24 hour intervals as close as possible.

Proposed secondary outcome measures:
Patient reported outcome measure (Day 1, Day 2, Day 3, Day 4, Day 5, Day 6 and Day 7)
Physician assessment of joint tenderness and swelling (Day 1, Day 2, and Day 7)
Assessment of activity limitation and quality of life: HAQ-DI and SF-36 at Day 1, Day 7 and 8 weeks; EQ-5D-5L at Day 1, Day 2, Day 7 and 8 weeks;
LEFS at Day 1, Day 2, Day 7.
Time to take rescue medication
Healthcare resource use.
Blood and urine tests renal on Day 1, Day 2, Day 7 and 8 weeks, consisting of FBC, U+Es, eGFR, glucose, CRP, serum uric acid and spot urine uric acid.
Qualitative assessment of participant’s experience at end of participation i.e. 8 weeks.

3.6 Exploratory endpoints/outcomes

For peer review only - http://bmjopen.bmj.com/site/about/guidelines.xhtml

Health Research Authority

Anakinra vs. Steroids for the Treatment of Gout Attacks in Renal Patients (ASGARD): A Feasibility Study

EudraCT no. 2015-001787-19

Measurement of inflammatory markers in blood and urine including. Serum and urine samples obtained will be stored for laboratory analysis at a later date. Changes in epigenetic DNA with both treatments.

Objectives	Outcome Measures	Time point(s) of evaluation of this outcome measure (if applicable)
**Primary Objectives**		
Feasibility of undertaking a definitive multicentre randomised controlled double blind, double dummy study<br><br>Safety and adverse event		End of study.<br><br>8 week post randomisation.
**Secondary Objectives**		
Proposed primary outcome measures for subsequent study looking at effectiveness of treatment with regards resolution of pain.	Time to 50% reduction and complete resolution of pain in self-assessed pain intensity in the joint most affected at baseline measure on the VAS (0-100mm) and 5-point Likert scale on patient diary.	At baseline (D1) to 7 days post randomisation (D1, D2, D3, D4, D5, D6, D7).
Proposed secondary outcome measures for subsequent study looking at effectiveness of treatment with regards to patients reported outcome measure and physician clinical assessment.<br><br>Patient assessment of activity limitation and quality of life.	Patient reported outcome measure on 5-point Likert scale on patient diary.<br><br>Physician assessment of joint tenderness and swelling on 5-point Likert scale.<br><br>• LEFS<br><br>• EQ-5D-5L	At baseline (D1) to 7 days post randomisation (D1, D2, D3, D4, D5, D6, D7).<br><br>At baseline (D1) and D2, D7.<br><br>At baseline (D1), D2, D7 and 8 weeks.

For peer review only - http://bmjopen.bmj.com/site/about/guidelines.xhtml

Health Research Authority

Anakinra vs. Steroids for the Treatment of Gout Attacks in Renal Patients (ASGARD): A Feasibility Study

EudraCT no. 2015-001787-19

	• HAQ-DI and SF-36	At baseline (D1), D7, and 8 weeks.
Use of rescue medication	Time to take pre-specified rescue medication or treatment failure.	At baseline (D1) to 7 days post randomisation (D1, D2, D3, D4, D5, D6, D7).
Health economic evaluation to compare the two treatment arms.	Healthcare Resource use data	8 weeks
Qualitative assessment of study involvement (12 participants)	Interview	8 weeks
Monitoring of blood counts, kidney function, glucose, uric acid and markers of inflammation.	Blood and urine test for Change in FBC, renal function by eGFR, CRP, glucose, uric acid and urine uric acid.	D1, D2, D7 and 8 weeks.
**Tertiary/Exploratory Objectives**		
Changes in inflammatory blood and urine markers, including epigenetic changes in circulating blood cells.	Inflammatory cytokines e.g. IL-1alpha, IL-6, TNF-alpha, TGF-beta) from stored serum, plasma and urine analysed at end of clinical trial. Changes in epigenetic DNA with treatment.	D1, D2, D7 and 8 weeks.

For peer review only

For peer review only - http://bmjopen.bmj.com/site/about/guidelines.xhtml

Health Research Authority

Anakinra vs. Steroids for the Treatment of Gout Attacks in Renal Patients (ASGARD): A Feasibility Study

EudraCT no. 2015-001787-19

4 TRIAL DESIGN

This study is a multi-centre, double-blind, double dummy randomised controlled trial in which patients will be randomised into two arms of treatment, Group A (Experimental arm) will be given Anakinra and placebo intramuscular methylprednisolone (Lipofundin), Group B (Active comparator) will be given intramuscular methylprednisolone (Depo-Medrone) and placebo Anakinra. It is a feasibility study and not powered for statistical inference. It is designed to gather safety information and feasibility of conducting a full-scale trial.

5 STUDY SETTING

This is a multi-centre randomised controlled trial based in centres in the East of England region. Each site will have a nominated principle investigator. Recruitment will be from patients who present with acute gout and chronic kidney disease in secondary care, or are already in secondary care.

Patients will be in pain from acute gout so recruitment will be in the acute setting, either from Accident and Emergency or the Acute/ Surgical Medical Unit. Patients already in hospital who develop acute gout attacks can also be approached for recruitment. Known patients with moderate kidney disease and difficult to treat acute gout will also be primed to contact the research team. We will use primary care sites as participant identification centres to help recruit potential participants before an acute attack by sending letters of invitation.

The majority of patients with acute gout flare can be easily treated. Recruiting from primary care was criticised in two studies[11,12]. We want to recruit patients who present to secondary care either because of co-morbidities, disease severity or functional disability affecting patient function.

We would suggest that primary care practitioners refer patients to secondary care who have co-morbidities, history of refractory disease or severely affecting function. A good interface between the rheumatologists (PI) and the acute services would be ideal.

The intervention will be co-ordinated by the trial team, once patients consent to randomisation, patients will receive treatment that would have been co-ordinated by the trial team. The pharmacy team will prepare the medication for administration and keep a record of medication allocation. This will be blinded to the trial team, treatment will be administered by the healthcare staff looking after the patient. The patient will be blinded throughout the study and even after the end of study.

All hospitals should be able to have pharmacy support to provide preparation and recording of treatment administered.

6 ELIGIBILITY CRITERIA

6.1 Inclusion criteria

For peer review only - http://bmjopen.bmj.com/site/about/guidelines.xhtml

Health Research Authority

Patients who meet inclusion criteria will be approached by a study investigator. Consent for participation will be sought. Upon gaining consent, the investigator will go through exclusion criteria.

1. Subjects capable of giving informed consent.
2. Male or non-pregnant, non-nursing female
3. ≥ 18 years of age
4. eGFR < 60mls/min/1.73m^2 and ≥ 30mls/min/1.73m^2 as calculated using serum creatinine and modified MDRD formula or CKD-EPI, on two occasions at least two months apart with one being in the last six months. Creatinine at time of presentation can be used unless participant has an acute kidney injury as defined by serum creatinine rise by ≥ 26μmol/L within 48 hours or ≥ 1.5 fold rise from baseline value.
5. Diagnosis of gout arthritis as defined by the American College of Rheumatology 1977 preliminary criteria.
6. Gout attack less ≤ 36 hours
7. Baseline pain intensity greater than or equal to 50mm on the 0-100 mm VAS. In the case of multiple joints (≤ 3), the most affected joint will be assessed.

6.2 Exclusion criteria

1. Treatment with Colchicine in the last week.
2. Initiation of or change to dose of NSAIDS in the last week. NSAID use is not an exclusion criterial if taken at a stable dose for at least four weeks.
3. Initiation of or change to dose of systemic steroid in the last week. Systemic steroid use is not an exclusion criteria if taken at an equivalent dose of 10mg Prednisolone or less for the last four weeks.
4. Polyarticular gout, i.e. affecting four or more 4 joints
5. Rheumatoid arthritis, evidence/suspicion of infectious/septic arthritis, or other acute inflammatory arthritis such as systemic lupus erythematosus, mixed connective tissue disease, scleroderma, polymyositis, or significant systemic involvement secondary to rheumatoid arthritis.
6. Con-current immunosuppression/immunomodulatory treatment (Calcineurin inhibitor, anti-proliferative or biologic) therapy for other reason i.e. organ transplant.
7. Prior history or current inflammatory joint disease other than gout (e.g. rheumatoid arthritis (RA), reactive arthritis, psoriatic arthritis, seronegative spondyloarthropathy, Lyme disease).
8. Current active malignancy (with the exception of basal cell or squamous cell carcinoma of the skin, cervical intraepithelial neoplasia and non-metastatic/advanced prostate cancer).
9. Any patients with contra-indication to intramuscular injection such as coagulopathy or thrombocytopenia (Platelet count<100 x 10^9/L (100,000/mm^3)).
10. Abnormal liver function tests: Total bilirubin>upper limit of normal, Alanine aminotransferase (ALT) or Aspartate Aminotransferase (AST) >2 times upper limit of normal.

For peer review only - http://bmjopen.bmj.com/site/about/guidelines.xhtml

Health Research Authority

Anakinra vs. Steroids for the Treatment of Gout Attacks in Renal Patients (ASGARD): A Feasibility Study

EudraCT no. 2015-001787-19

11. Haemoglobin < 85g/L (8.5 g/dL)

12. White blood cell (WBC) count<1.5 x 10^9/L (1000/mm^3), absolute neutrophil count<1.5 x 10^9/L (1000/mm^3)

13. Evidence of active infection requiring intra-venous (IV) anti-biotic drug administration within last four weeks. Potential participants who were empirically treated with a course of intravenous antibiotics for a presumptive diagnosis of an infection may be eligible where the treating medical care team has subsequently excluded an active infection and consequently discontinued antibiotic therapy at least four hours before randomisation. These potential participants can be discussed with the investigator where clarity is required.

14. Evidence of uncontrolled concomitant cardiovascular, nervous system, pulmonary (including obstructive pulmonary disease), hepatic, endocrine (including uncontrolled diabetes) or gastrointestinal disease. Potential participants who have active concomitant disease can only be eligible after discussion and agreement with the treating medical team.

15. Known positive hepatitis B virus surface antigen (HBsAg), hepatitis C (HCV) antibody or HIV.

16. Females of child bearing potential who are not willing to use highly effective birth control methods from the time of consent to one week after treatment discontinuation. Highly effective method of contraception (hormonal or barrier method of birth control; abstinence) consist of:

    - Combined (estrogen and progestogen containing) hormonal contraception associated with inhibition of ovulation; oral, intravaginal, transdermal.
    - Progestogen-only hormonal contraception associated with inhibition of ovulation; oral, injectable, implantable.
    - Intrauterine device (IUD) or intrauterine hormone-releasing system (IUS)
    - Bilateral tubal occlusion or vasectomised partner
    - Sexual abstinence, defined as true abstinence when it is in line with the preferred and usual lifestyle of the participant. Periodic abstinence (e.g. calendar, ovulation, sympthothermal, post-ovulation) and withdrawal methods are not acceptable methods of contraception.

17. Men who are sexually active with a female partner who are not willing to use highly effective birth control methods from the time of consent to one week after treatment discontinuation.

18. Females of childbearing potential must have a negative pregnancy test (highly sensitive urine or serum pregnancy test after a confirmed menstrual period) within 7 days prior to treatment initiation. Subjects are considered not of child bearing potential if they are surgically sterile (i.e. they have undergone a hysterectomy, bilateral tubal ligation, or bilateral oophorectomy) or they are postmenopausal.

19. Females must not be breastfeeding.

20. Patients who have had treatment as part of this trial cannot have repeat treatment for another flare as part of the trial.

For peer review only - http://bmjopen.bmj.com/site/about/guidelines.xhtml

21. Patients with allergies to excipients of IMPs: citric acid, anhydrous, sodium chloride, disodium edetate dehydrate, polysorbate 80, sodium hydroxide. Hypersensitivity to E. Coli derived proteins, egg proteins and soy proteins. Patients with a latex allergy are also not eligible as the inner needle cover of the pre-filled syringe contains dry natural rubber (a derivative of latex).

7 TRIAL PROCEDURES

7.1 Recruitment

Patient who present to secondary care with acute swollen joints with a diagnosis of acute gout will be approached for recruitment. Patients already in hospital as in-patients will also be eligible for screening. Primary care practitioners will be encouraged to send difficult to manage cases such as patients with gout that effects their function and complex treatment (i.e. previous history of difficult to treat gout), as per our local CCG guidelines. This may either be to acute secondary care services or rheumatology out-patients depending on the local practice of the centre. Known patients with moderate CKD and a history of acute gout can be primed to contact the research team either via the general practitioner or the acute services, or directly. We will use primary care sites as participant identification centres to help recruit potential participants before an acute attack by sending letters of invitation. This will help minimise any delays to treatment as part of the study during an acute attack.

Initial diagnosis may be made by any clinician, the trial team based at that centre screen patients using standardised criteria for diagnosis as per eligibility criteria (used by historical and current trials of acute gout). A pre-specified list of analgesic that would least likely impact on the outcome can be used for pragmatic reasons prior to screening to avoid delaying relieving patient discomfort. Patients who present out of hours may not be approached for recruitment as to not avoid delays in treatment.

Information will be sought on patients who are screened and for participants who are not randomised as per Consolidated Standard of Reporting Trials (CONSORT) guidance.

7.1.1 Patient identification

The patient will be initially identified by a nurse practitioner or clinician in the participating acute trusts. Patients with hot joints can present to the emergency room, acute medical take, surgical take (orthopaedics), directly to rheumatologists or in-patients who develop acute gout, which is a common presentation. Patients will only be identified on clinical presentation to medical services i.e. the clinical team in charge of the patient’s care. We will use primary care sites as participant identification centres to help identify potential participants before an acute attack by sending letters of invitation.

7.1.2 Pre-Screening

Trial posters will be displayed in primary care and community care settings, directing potential participants to their nearest research site and contact person. The same posters may be used to advertise the study via hospital clinic waiting areas, in-patient wards, internet and social media.

For peer review only - http://bmjopen.bmj.com/site/about/guidelines.xhtml

Health Research Authority

Potential participants will undergo an initial pre-screening process for basic eligibility; inclusion and exclusion. This will include a review of renal function and information on previous testing for gout diagnosis such as uric acid, and findings of imaging and joint fluid analysis.

This will help avoid ineligible participants undergoing a baseline assessment and consent process who can be discounted as being ineligible early on. These patients would still receive treatment by the research team for their joint condition / presentation. Potentially eligible patients will also be assessed sooner and if randomised, receive allocated treatment sooner for their painful presentation.

7.1.3 Screening

The clinical presentation of an acute hot swollen joint due to a diagnosis of gout should prompt referral by the nurse practitioner or clinician for screening if the patient is willing to be approached. Tests may be undertaken by the clinical team responsible for the patients care to confirm or refute the diagnosis as part of on-going standard care for the patient. All patients must freely give their informed consent before any trial-related procedures may be conducted. These will consist of:

- Gaining informed consent from the patient
- Recording demographic details: gender, date-of-birth, ethnic origin, previous medical history (diabetes, cardiovascular disease, respiratory disease, smoking details, and alcohol intake).
- Concomitant medications
- History of Hepatitis B, C, HIV
- History of malignancy
- Additional blood tests may consist of clotting (could be added onto initial request if possible), liver function tests, full blood count and renal function

7.2 Consent

The Principal Investigator (PI) retains overall responsibility for the informed consent of participants at their site and must ensure that any person delegated responsibility to participate in the informed consent process is duly authorised, trained and competent to participate according to the ethically approved protocol, principles of Good Clinical Practice (GCP) and Declaration of Helsinki. If delegation of consent is acceptable then details of above should be provided for the delegated individual. A clinician can only obtain consent.

Informed consent will be obtained prior to the participant undergoing procedures that are specifically for the purposes of the trial and are out-with standard routine care at the participating site (including the collection of identifiable participant data). The right of a participant to refuse participation without giving reasons will be respected. The participant will remain free to withdraw at any time from the trial without giving reasons and without prejudicing his/her further treatment and must be provided with a contact point where he/she may obtain further information about the trial.

For peer review only - http://bmjopen.bmj.com/site/about/guidelines.xhtml

Health Research Authority

Anakinra vs. Steroids for the Treatment of Gout Attacks in Renal Patients (ASGARD): A Feasibility Study

EudraCT no. 2015-001787-19

If a participant is required to re-consent or if new information is required to be provided to a participant it will be the responsibility of the PI to ensure this is done in a timely manner.

Potential participants should be able to give consent and a person is assumed to have mental capacity to make a decision unless it is shown to be absent.

Assessment of capacity for participants to give consent themselves will consist of a person who:

- Understand the purpose and nature of the research
- Understand what the research involves, its benefits (or lack of benefits), risks and burdens
- Understand the alternatives to taking part
- Be able to retain the information long enough to make an effective decision.
- Be able to make a free choice
- Be capable of making this particular decision at the time it needs to be made (though their capacity may fluctuate, and they may be capable of making some decisions but not others depending on their complexity)
- Where participants are capable of consenting for themselves but are particularly susceptible to coercion, it is important to explain how their interests will be protected

Patients who are unable to consent for themselves for suitability for the trial will not be approached as obtaining patient reported outcome measures will not be practical. If verbal translation is needed, this should be via a hospital interpreter or a personal interpreter. Telephone interpretation services are not acceptable and written material will not be provided in various languages for the purpose of this feasibility study. This study utilises questionnaire surveys that have not been validated in different languages. There is also a qualitative element to the study where lack of required language skill may prove to be difficult. Time is limited for potential participants to be considering participation and any delays in seeking consent and translation services that may prolong treatment of the patient in acute pain have also to be considered.

Written material consisting of participant information leaflet and consent documentation will be approved by the Research Ethics Committee and will be in compliance with GCP, local regulatory and legal requirement. This is a feasibility study and the costs to undertake centrally commissioned translated documents may be too high. This may be something to consider for the subsequent definitive study.

There will be opportunity for the participant to ask questions to the PI or a member of the research team. The patient will be given 24 hours to consider the information and consider their participation. However, treatment cannot be withheld during this period and exclusion criteria will be applied if duration of attack is longer than accepted or if contra-indicatory medication is administered.

A copy of the ICF and PIS will be given to the participant, with the original ICF filed in the medical notes and a copy of the ICF in the ISF.

Consent will be specifically obtained for:

- Permission to have access to relevant sections of medical records by study team members, including the members from the coordinating center (ARCTU), the primary centre (Southend Hospital), and regulatory authorities

- Identifiable routine blood tests which will be stored within the NHS clinical system and will be available for doctors in the future if required for future care within the NHS system

For peer review only - http://bmjopen.bmj.com/site/about/guidelines.xhtml

Health Research Authority

- Contact information to be made available to the research team in the site file for ongoing assessment and monitoring as part of the study. Information will also be used to update participant of any new findings that may become relevant during the course of the study. This may include safety information resulting in significant changes to the study risk: benefit assessment, the protocol, PIS and/or the ICF, will be reviewed and updated as necessary. Participants will be given a copy of the revised consent form. They will then be asked to re-consent if they choose to continue the study.
- Permission for audio interviews to be recorded for the qualitative component for the study.
- Permission to retain contact details for long-term follow-up with regards to use of subsequent urate-lowering therapy and complications, and to pass on information regarding details of future research.

If a participant who has consented but later becomes incapacitated, the original consent endures the loss of capacity but patient may be withdrawn if patient reported outcome data cannot be obtained.

7.2.1 Additional consent provisions for collection and use of participant data and biological specimens in ancillary studies, if applicable

There will be a tertiary / exploratory study where patient's serum and urine will be stored for future analysis at the end of the study. Testing will be an extension of routine laboratory testing to look for markers of inflammation in an exploratory setting.

Consent will be obtained specifically for the use of DNA, although it will be made clear that the research is concerned with inflammatory changes in epigenetic DNA and subsequent changes with treatment in gout. Patients will have the option of not contributing to this study as part of the consent procedure, and also withdrawal at a subsequent date.

Permission will be obtained specifically for storage of samples up to 15 years and to be used for future, as yet unspecified, medical research into health, illness and medical treatment. This research will be subject to proper scientific and ethical review.

Some samples will be exported to countries in the European Union for further analysis. Participants will be consented specifically for this and exported material will be handled, stored, transported and disposed in accordance with the consent given. The collaborators / recipient country will ensure that the material has been handled properly and that the required standards of that collaborating institution / country have been met.

7.3 The randomisation scheme

Enrolled patients will undergo 1:1 block randomisation by ARCTU, to either Anakinra (Experimental arm) or intramuscular methylprednisolone (active comparator arm). Randomisation will be undertaken using an Internet-based randomisation system within ARCTU. The ARCTU uses the TENALEA system provided by the Trans European Network for Clinical Trial Service. The system stores the pre-determined sequence of randomisation.

This list is not available to the investigator or ARCTU staff. Once a patient has consented to take part in the trial, the designated staff will login to a web page after entering information required to check eligibility

For peer review only - http://bmjopen.bmj.com/site/about/guidelines.xhtml

and they will be randomly allocated to either arm. Allocation will be sent to pharmacy who will then dispense allocated treatment. All investigators involved with the trial will be blinded to treatment. Patients will not be informed of their assigned treatment during the study. Pharmacy will keep a record of allocated treatment arms in the event emergency un-blinding is required. This will be reported to the central pharmacy to keep up stocks of relevant medication as well.

7.3.1 Method of implementing the allocation sequence

A web based randomisation/treatment allocation system will be used. Once allocated to a treatment arm, the pharmacy department will co-ordinate the treatments. A treatment and appropriate placebo will be generated by pharmacy, this will be concealed to the trial team and healthcare team involved in administering treatment. The pharmacy will keep a record of all treatments given.

Anakinra and its placebo will be ready made by the manufacturer. Intramuscular methylprednisolone and its placebo (Lipofundin) will be prepared by a research team member or healthcare team member not involved with the study and be administered by healthcare team caring for the patient, and not involved with the trial. Patients who present out-of-hours where implementation of randomisation may delay treatment should not be approached for study. Randomisation codes will be available from pharmacy in the event of an emergency or if out-of-hours access is required.

Participants will be randomised to one of two treatment arms, Group A and Group B:

- Group A: Anakinra 100mg s/c Day 1 to Day 5 and placebo intramuscular methylprednisolone (Lipofundin) 120mg i/m Day 1
- Group B: Intramuscular methylprednisolone (Depo-Medrone) 120mg i/m Day 1 and placebo Anakinra 100mg s/c Da 1 to Day 5

7.4 Blinding

All trial investigators will be blinded to the intervention. Pharmacy will keep a record of treatments administrated. All trial participants, care providers and outcome assessors will be blinded to the treatment. Each treatment has its equivalent placebo to ensure blinding in maintained throughout study.

7.5 Un-blinding

The study code should only be broken for valid medical or safety reasons e.g. in the case of a severe adverse event where it is necessary for the investigator or treating health care professional to know which treatment the patient is receiving before the participant can be treated.

The treatment code for the patient can be broken in the reporting of a SUSAR. However, the blind should be maintained, where possible and appropriate, for staff that are involved in data analysis and interpretation. It is the allocated responsibility of the CI by the sponsor for pharmacovigilance management and reporting. In this instance, an allocated un-blinded individual (s), with no involvement in data management of the study should be responsible for the un-blinding event. The un-blinding of single cases by the PI/CI in the course of a clinical trial should only be performed if necessary for the safety of the trial participant.

We do not expect CTIMPs to be associated with high morbidity and mortality, and feel that most events will be related to the underlying risk inherent in the patient group. This study is not powered as a comparison of efficacy / effectiveness and systemic un-blinding could be considered in the event of SUSARs / SAEs if the PI and sponsor feel that it may be related to trial treatment and is clearly

For peer review only - http://bmjopen.bmj.com/site/about/guidelines.xhtml

necessary for the appropriate medical management of the participant. In all cases the Investigator would be expected to evaluate the causality and expectedness of SAEs as though the participant was receiving the active medication.

The case should be assessed for seriousness, expectedness and causal relationship as if it was the tested IMP that caused the reaction. If the case appears to be a SUSAR then it should be un-blinded and the following considered:

- If the administered product is the tested IMP, the case would be reported as a SUSAR to the MHRA/ appropriate Main Research Ethics Committee/IMP provider (if applicable) within the timelines outlined in safety reporting section.
- If the administered product is a comparator with a marketing authorisation, the adverse reaction should be reassessed for expectedness according to the study protocol. If the adverse reaction is unexpected then the SUSAR should be reported; otherwise it is an expected serious adverse reaction which still requires reporting to the sponsor/IMP provider (if applicable) within 24 hours.

Emergency un-blinding/code breaks can be undertaken by the PI or medical team responsible for the care for the patients from the pharmacy department which will have a record of all treatment allocations for the patient at the respective centre. This is available out-of-hours. The investigator is not required to discuss un-blinding if he or she feels that emergent un-blinding is necessary.

The pharmacy representative who is responsible for code breaks at ______________________________

is ______________________________________.

Failing this, the hub pharmacy centre will keep a record of participant trial number and allocation.

If the person requiring the un-blinding is a member of the Investigating team then a request to the holder of the code break envelope/list, or their delegate will be made and the un-blinded information obtained. If the person requiring the un-blinding is not the CI/PI then that health care professional will notify the Investigating team that an un-blinding is required for a trial subject and an assessment to un-blind should be made in consultation with the clinical and research teams. On receipt of the treatment allocation details the CI/PI or treating health care professional will continue to deal with the participant's medical emergency as appropriate.

Details of patients who have pre-mature un-blinding, including outcomes, will still be used for analysis. The CI/PI documents the breaking of the code and the reasons for doing so on the CRF/data collection tool, in the site file and medical notes. It will also be documented at the end of the study in any final study report and/or statistical report. The CI/Investigating team will notify the Sponsor in writing as soon as possible following the code break detailing the necessity of the code break. The CI/PI will also notify the relevant authorities. The written information will be disseminated to the TSC.

7.6 Baseline data / Pre-treatment Evaluations (Day 1)

This assessment should not occur within four hours of having taken an analgesic such as codeine, tramadol or paracetamol. The PI or trial staff (research nurse, research fellow) will undertake an assessment of the joint using a pre-specified scoring system.

- Vital signs (height, weight, general clinical assessment)

For peer review only - http://bmjopen.bmj.com/site/about/guidelines.xhtml

- Medical history specifically history of ischaemia heart disease, heart failure, type 2 or type 1 diabetes, peripheral vascular disease, cerebrovascular disease.
- Information in relation to new ACR/EULAR 2015 criteria

  Previous episode(s)

  - Pattern of previous joint(s) / bursa(e) involvement during previous symptomatic episodes
    - Not ankle, midfoot, or first MTP joint (or involved as part of polyarticular presentation)
    - Ankle *or* midfoot (as part of monoarticular or oligoarticular episode **without** involvement of first MTP)
    - Involvement of first MTP (as part of monoarticular or oligoarticular episode)
  - Characteristic of previous symptomatic episode(s)
    - Erythema overlying affected joint (patient- reported or physician – observed)
    - Can't bear to touch or pressure to affected joint
    - Great difficulty in walking or inability to use affected joint
  - Time course of previous episode(s);

    Number of previous episodes with 2 ≥ of (irrespective of anti-inflammatory treatment):
    - Time to maximal pain < 24 hours
    - Resolution of symptoms ≤ 14 days
    - Complete resolution (to baseline level) between symptomatic episodes
  - Previous serum urate level > 4 weeks from start of episodes or inter-critical period

    (Record highest value)
  - Previous synovial fluid analysis of symptomatic joint
    - MSU negative or positive
  - Previous imaging evidence of urate deposition in symptomatic joint or bursa: ultrasound evidence of double-contour sign *or* dual energy CT demonstrating urate deposition

  Current episode

  - Was joint aspiration undertaken, was MSU negative or positive
  - Pattern of joint(s) / bursa(e) involvement
  - Was imaging undertaken?
    - Imaging evidence of urate deposition in symptomatic joint or bursa: ultrasound evidence of double-contour sign *or* DECT demonstrating urate deposition
    - Imaging evidence of gout-related joint damage: conventional radiography of the hands and/or feet demonstrates at least 1 erosion
- Baseline renal function defined as CKD3; calculated using serum creatinine and modified MDRD formula or CKD-EPI, on two occasions at least two months apart with (one being in the last six months), if previous results not available use creatinine at time of hospital admission. Creatinine at time of presentation can be used unless participant has an acute kidney injury as defined by serum creatinine rise by ≥ 26μmol/L within 48 hours or ≥ 1.5 fold rise from baseline value.

For peer review only - http://bmjopen.bmj.com/site/about/guidelines.xhtml

- Blood and urine samples for routine testing within the NHS clinical system.
- Pain score using Likert and VAS and baseline outcome score on patient issued diary
- Clinical assessment of joint using standardised scoring assessment
- Quality of life questionnaires: SF-36, HAD-DI, EuroQol-5D, LEFS.
- Additional blood tests (one vial/one teaspoonful) and urine (one universal) for further study.

7.7 Trial assessments

This assessment should not occur within four hours of having taken an analgesic such as codeine, tramadol or paracetamol. The PI or trial staff (research nurse, research fellow) will undertake an assessment of the joint using a pre-specified scoring system.

First dose will be given on Day 1.

7.7.1 Evaluations after treatment

- Treatment Day 2, 3, 4, 5, 6, 7
- Pain score on diary card (VAS and Likert) - Day 2, 3, 4, 5, 6, 7
- Patient reported outcome measure of response to treatment (Likert) - Day 2, 3, 4, 5, 6, 7 Participants can be contacted on Friday and Monday as a reminder for weekend or compliance check respectively.
- Hospital visit for physician / study member assessment of joint tenderness and swelling (Day 1, Day 2 and Day 7)
- Assessment of activity limitation and quality of life using:
    - LEFS at Day 1, Day 2, Day 7.
    - EQ-5D-5L at Day 1, Day 2, Day 7 and 8 weeks.
    - HAQ-DI and SF-36 at Day 1, Day 7 and 8 weeks
- Blood and urine tests on Day 1, Day 2 Day 7, and Week 8 consisting of FBC, U+Es, eGFR, glucose, CRP, serum uric acid and spot urine uric acid. Additional bloods tests for exploratory tests.
- Last assessment at 8 weeks: resource use data and interview (12 patients) for qualitative assessment of participant's experience.

- If more than one joint is effected, then the worst effected joint is assessed.
- Participants will have their diary card entry checked; diary card entry will also note time of injection administration. On non-hospital attendance days, trial team will contact participants to administration of injection and entry into diary card. Participants should make self-assessments at time of diary card entry late morning or afternoon.
- Compliance with injections will be checked by asking participants to return used syringes back to trial team and this will be recorded. An inspection of the injection site will also be made. Arrangements can be made to collect non-puncture containers if patients who are not able to deliver it themselves.
- If a participant is not able to self-inject, then a carer could administer the injection or district nurses will be organised to administer injection.
- Patients will then attend hospital at 8 weeks to undertake last questionnaires. Diary cards will be checked on each hospital visit and be returned by the patient on Day 7.

For peer review only - http://bmjopen.bmj.com/site/about/guidelines.xhtml

- Patients who cannot attend hospital due to physical/medical reasons can be contacted by phone for administration of dose and diary card entry. Final assessment questionnaires at 8 weeks can be sent by the patient by mail, if patient is not able to attend for hospital visits.
- Patients will be asked at 8 weeks if any events that may have occurred for pharmacovigilance and also to undertake a telephone questionnaire of resource use. Patients may (12 out of 32) be asked to have a qualitative assessment of their experience of being involved with the trial.
- Blood and urine tests will be undertaken at Day 1 (pre-dose), Day 2, Day 7 and week 8, during hospital visits. Sample for the routine clinical assessments, including FBC, U+Es, CRP, urine uric acid / serum uric acid will be processed by the local centre. Samples for the exploratory study (serum and urine) will be processed and stored at the local centre. Samples will be stored using an anonymised unique identifier, once batched up to the end of study, samples will be sent to Southend University Hospital for storage and subsequent analysis of inflammatory markers.

7.8 Long term follow-up assessments

The final follow-up for the participant will be at 8 weeks. 8-week follow-up will consist for looking for any events possible related to treatment toxicity as part of CTIMP reporting. Patients will be asked to return completed questionnaires. Resource use data will be used to assess healthcare use for economic analysis.

Patients who give consent for further follow-up will be contacted for further research and their details stored for this purpose.

This is a short study and non-adherence and “loss to follow-up” will hopefully be minimal. Measure to improve adherence including using telephone calls to prompt treatment administration and pain assessments. The long-term 8-week follow-up can be conducted by telephone if required with participants sending in questionnaires by post.

7.9 Qualitative assessments – Nested studies

A qualitative assessment will be undertaken at the end of the study which will be undertaken as an interview with regards to obtaining participation experience. This was suggested by the funding body. Twelve out of the thirty-two patients will be randomly selected for qualitative assessment of participation experience.

Consent to obtain unlinked anonymised data with contact information for the qualitative aspect of the study at the 8-week time point will be explicitly obtained. This will be stored in the site file. Consent will be explicitly obtained for audio interviews to be recorded. Pseudonymised data will be used for recording purposes and data obtained will be stored in a locked cabinet with secure access only to Dr Jon Scales, University of Essex.

7.10 Withdrawal criteria

The investigator will make reasonable effort to keep each patient on the study for completeness sake. If the investigator removes a patient from the study, or if a patient declines further participation, prior to interventions

7.10.1 Patients lost

- **Withdrawn**

For peer review only - http://bmjopen.bmj.com/site/about/guidelines.xhtml

Health Research Authority

Patients who withdraw their consent at any point in the study fall into one of two categories. Site staff should ascertain which category a patient wishes to be in at the point of withdrawal;

- those who allow their data (collected up to the point of withdrawal) to be used

OR

- those who do not allow the use of any of their data collected prior to withdrawal

- **Lost to follow-up**

Patients who do not attend follow-up assessment after site staff have attempted to contact the patient at least twice, e.g. by telephone.

As these patients have not withdrawn their consent, the data already collected for them may be used and therefore needs returning in the usual manner.

These patients have not withdrawn their consent and therefore the data collected for them may be used.

7.10.2 Patients not lost

Patients that fall into one or more of the following categories are not considered as withdrawn or lost from the study. They should continue to attend all follow-up study assessments as per protocol, unless they subsequently fall into one of the categories listed in Section 7.1;

- Patients who ask to stop study treatment
- Pregnancy
- Serious infection i.e. necessitating intravenous anti-biotic treatment and hospital admission.
- Any event which in the judgement of the PI makes further study treatment inadvisable
- SAE requiring discontinuation of treatment

It is always within the remit of the physician responsible for a patient to withdraw a patient from a trial for appropriate medical reasons, be they individual adverse events or new information gained about a treatment. If any further adverse data regarding the IMP come to light patients will be withdrawn from the study.

7.10.3 Withdrawal documentation

All withdrawals will be monitored by the sponsor and a record will be kept. We will have a formal withdrawal document in place.

7.11 Storage and analysis of samples

Blood and urine samples will be obtained at baseline (day 1), and at hospital visits at day 2, day 7 and at 8 weeks. Patients should not require much further testing as these blood tests are routinely undertaken to assess disease response anyway.

Serum sample will be obtained using 6mL red top blood sample containers / vacutainers (a tablespoonful). The sample will be spun down at 1500g for 15 minutes. Using a plastic pipette, three X 2mLs aliquots will be stored in cryotubes. Serum will be stored at -80°C (+/- 10) freezer in the local centre.

For peer review only - http://bmjopen.bmj.com/site/about/guidelines.xhtml

Plasma sample will be obtained by using a 6ml EDTA (pink top) and spun down at 1500g for 15minutes. Using a plastic pipette, three X 2mLs aliquots will be stored in cryotubes. Serum will be stored at -80ºC (+/- 10) freezer in the local centre. The cell pellet will be stored at -80ºC for further use.

Urine samples (early morning if possible) will be obtained. Patients will be given collection pots and be asked to bring sample with them at assessment visits. If -80ºC storage is not possible at the local centre, samples can be stored at -20ºC for a month and arrangements will be made for transfer to the primary centre for storage.

All research blood and urine samples (link-anonymised) will be processed at each site (each site will have a Sample Analysis and Chain of Custody Plans in the Study Operations Manual / Standard Operating Protocol). Samples will be transported to Southend Hospital at the end of study under the custodianship of the Chief Investigator.

Analysis will be done at the end of the study. Serum, plasma and urine samples will then be analysed at using validated scientific methods to quantify inflammatory markers. Inflammatory changes in the cell epigenetics will also be examined.

Consents, storage and use of samples will be underpinned by the code of practice outlined by the Human Tissue Act and Medical Research Council guidance. All human samples will be treated as donated gifts and all research will be conducted with respect and transparency.

A system of coding will be used to pseudonymise the samples obtained. Only the chief investigator will have access to the codes which will be stored in secure storage (password protected) in a locked room. The chief investigator will only have access to the secure database that would permit identification of a sample with a trial number, and subsequent identification of the participant whose material is being used. The research material will not be identifiable to the researcher and researchers, including the principle investigator, cannot link the sample to the participant. The samples will be under the custodianship of Gowrie Balasubramaniam, Chief Investigator.

Participants will be consented specifically for storage of samples up to 15 years and to be used for future, as yet unspecified, medical research into health, illness and medical treatment. This research will be subject to proper scientific and ethical review.

Some samples will be exported to countries in the European Union for further analysis. Participants will be consented specifically for this and exported material will be handled, stored, transported and disposed in accordance with the consent given. The collaborators / recipient country will ensure that the material has been handled properly and that the required standards of that collaborating institution / country have been met.

Consent will be obtained specifically for the use of DNA, although it will be made clear that the research is concerned with inflammatory changes in epigenetic DNA and subsequent changes with treatment in gout. There will be no DNA genetic testing per se that may reveal significant results such as a family genetic condition etc. We will make it at the outset of the consent that this is the case. The DNA is extracted from cells, which would otherwise be discarded if we were just using serum or plasma samples. This maximises use of samples.

For peer review only - http://bmjopen.bmj.com/site/about/guidelines.xhtml

Health Research Authority

Anakinra vs. Steroids for the Treatment of Gout Attacks in Renal Patients (ASGARD): A Feasibility Study

EudraCT no. 2015-001787-19

7.12 End of trial

The recruitment period will be stopped after 19 months (15 months in duration after 4 month start-up phase) or if the trial is stopped for another reason by the steering committee, funding body or regulatory body.

The active treatment phase will be completed five days after the last participant is randomised.

The end of the trial in after the completion of final assessment, 8 weeks after the last patient is randomised.

All data available for the patient at the time of discontinuation of follow-up must be recorded in the eCRF. All reasons for discontinuation of treatment must be documented. In terminating the study, the sponsor (SUGH) and the Investigators will ensure that adequate consideration is given to the protection of the patient's interest.

The REC which gave the favourable opinion will be notified of the trial conclusion in writing. A declaration of end of a clinical trial of investigational medicinal products will be sent to the MHRA. This will be done within 90 days of the end of study as defined by the last visit / data item of the last participant in the trial. Formal notification template from the HRA website will be used: http://www.hra.nhs.uk/research-community/end-of-study-and-beyond/notifying-the-end-of-study/.

7.13 Rescue medications

Participants can take analgesia listed in the protocol as non-investigational medicinal product (co-dydramol, codeine phosphate, tramadol, and paracetamol) for relief as required, usage will be recorded. If participants get another flare during treatment, this will be recorded and a coarse of oral prednisolone 35mg po od for 5 days will be used for rescue treatment. Low dose colchicine 500micrograms po bd could be used in addition. Use of other immune modifying treatment or NSAIDS is prohibited.

For peer review only - http://bmjopen.bmj.com/site/about/guidelines.xhtml

8 TRIAL MEDICATION

There are three investigational trial medications, Anakinra, an Interleukin-1 inhibitor, intramuscular methylprednisolone acetate and its placebo, Lipofundin.

Anakinra has already marketed in the UK for the treatment of inflammatory conditions. It has been used in the treatment of acute gout we are undertaking a systematic assessment compared to the current standard of care in the UK. Hence, this falls under the remit as an Investigational Medicinal Product. Contemporary trials that use a similar mode of action have been reported with reasonable safety outcomes so some class evidence is available.

Intramuscular methylprednisolone acetate (Depo-Medrone) is the preferred depot steroid injection in the UK. It has been proven in acute inflammatory joint conditions but there are no trial looking specifically at acute gout attacks. There is a huge body of evidence with drugs using a similar mode of action and it is currently the safest option to treat acute gout attacks in patients with kidney disease. Intramuscular methylprednisolone acetate (Depo-Medrone), per se, has not been used in a trial looking at acute gout and so it falls under the remit as an investigation medicinal product.

Lipofundin MCT/LCT 10% is a milky white, sterile emulsion commonly used to provide fat and essential fatty acid for par-enteral feeding. It has been used as a placebo to depot steroid injections, including in contemporary studies looking at Canakinumab against Triamcinolone. No adverse events were reported. Injectable lipid emulsions have been used for more than forty years and the components in Lipofundin MCT/LCT are made from egg protein and soya-bean protein and these agents are commonly used carriers for non-aqueous, oil soluble drugs.

8.1.1 IMP: Anakinra

Anakinra (Kineret®) is an IL-1 receptor antagonist that is similar to the endogenous IL-1 RA that negatively regulates inflammation. There are other ways of inhibiting IL-1 action but Kineret is the only recombinant, non-glycosylated protein that differs slightly from the native form by the addition of a single methionine residue at its amino terminus (153 amino acids, MW 17.3kd). It will be supplied free of charge by the manufacturer, Swedish Orphan Biovitium.

Licensed drug name: Kineret®

8.1.2 Legal status of the drug

Anakinra has US FDA approval for use in Rheumatoid Arthritis and Cryopyrin-Associated Periodic Syndrome (CAPS). It is currently not recommended by NICE (February 2009, NICE) for RA except in a controlled clinical study or in difficult to treat cases which has not responded to methotrexate alone; it is not recommended for routine management. The Scottish Medicines Consortium has advised that Anakinra is not recommended for the treatment of rheumatoid arthritis within Scotland. Anakinra is currently not licenced for use in the treatment of acute gout.

8.1.3 Summary of Product Characteristics (SmPC)

Available from www.kineretx.com/fileadmin/user/pdfs/Kineret_Full_Prescribing_Information.pdf

Issue date: 12/2012

For peer review only - http://bmjopen.bmj.com/site/about/guidelines.xhtml

The manufacture's recommend Kineret not be used in patients with hypersensitivity to E-Coli derived proteins or any components of the product. Administration should be discontinued if a patient develops a serious infection and should not be initiated in a patient with active infection. Safety of the product in immunosuppressed or patients with chronic infections has not been evaluated. The recommendations are derived from RA trials with a prolonged duration of treatment for chronic inflammation. Our study only requires five days treatment and cohorts have been published for acute gout attacks in patients with active infection. Furthermore, the initial human use of Anakinra was in sepsis syndrome where patients were given a loading dose of 100mg followed by an infusion or 1-2mg/kg per hour did not show a worse outcome (Fisher et al. 1994).

Pharmacodynamics and pharmacokinetics

Bioavailability after an injection is 95% and maximum plasma concentrations were reached after 3-7 hours after subcutaneous administration. The terminal half-life is between 4-6 hours and clearance was dependent on creatinine clearance and weight. Plasma clearance falls to 70-75% in patients with severe or end-stage kidney disease (eGFR < 30 mL/min/m^2) (Yang et al, 2003). Anakinra has been used in patients on dialysis on an alternate day regime for 14 weeks (Hung et al, 2011).

8.1.4 Drug storage and supply

The investigational product will be supplied by the manufacturer free of charge. It will be stocked by the co-ordinating trial site / sponsor (SUFT) and distributed to other sites for storage. The equivalent placebo will also be provided by the manufacturer of Anakinra and handled as the investigational product.

The IMP is being provided for use in the trial but hospital can purchase IMP for used on their patients. Anakinra and its equivalent placebo does not require re-constitution.

Dosage form: It is supplied as a pre-filled glass syringe with 27 gauge needles as a sterile, clear, colourless-to-white, preservative free solution for daily subcutaneous (SC) injection. Each pre-filled syringe contains 0.67mLs (100mg) Anakinra in a solution (pH 6.5) containing EDTA (0.12mg), Sodium Chloride (5.48mg), Sodium Citrate (1.29mg), and polysorbate 80 (0.70mg) in water for injection.

Packaging and storage: Kineret is dispensed in a 4X7 syringe dispensing pack containing 28 syringes and also a 1X7 syringe dispensing pack. Kineret should be stored in the refrigerator at 2-8 °C degree Celsius (36 to 46 Fahrenheit-US). Shelf life is 3 years. It should be protected from light.

Disposal: After use patients will place syringe, without re-sheathing, into a puncture-resistant container (sharp bin). This will be returned to the trial site on day 7 and the number of used needles will be counted. Syringes will be disposed as per normal hospital policy on disposal of sharps.

8.1.5 Preparation and labelling of Investigational Medicinal Product

The drug will be supplied in a pre-filled syringe. It will have an equivalent placebo.

8.1.6 Dosage schedules

At recruitment and randomisation, participants enrolled into the Anakinra arm will have a D1 SC injection of 100mg Anakinra. This will continue daily for 5 days in total (i.e. D1 to D5). Patients in the steroid arm for investigation product will have similar regime with the equivalent placebo.

The glass syringes are pre-filled to 100mg.

For peer review only - http://bmjopen.bmj.com/site/about/guidelines.xhtml

8.1.7 Dosage modifications

There will be no dosage modification.

8.1.8 Drug Accountability

Southend University Hospital will act as a hub pharmacy and receive supply of Anakinra and its equivalent placebo from the manufacturer. Stand-alone pharmacies will receive an agreed number of packs depending on the expected recruitment rate for the size of the centre.

Documentation

Accurate records of all drug shipments, medication dispensed, and all drug returned, must be maintained. The inventory record must be available for inspection by the sponsor but information that may lead to un-blinding will be minimised. The site PI or CI does not destroy any unused drug unless directed to by the sponsor. Any destroyed drug must be destroyed according to hospital procedures and properly accounted for. At the conclusion of the study, the overall numbers of the drug shipped to the centre, and the number destroyed or returned, will be provided by the pharmacy, and an account given of any discrepancy.

Dispensing procedure

Once a research team has undertaken randomisation for a patient after recruitment, they will inform pharmacy. Pharmacy will be given the allocation details and will prepare appropriate medication or placebo accordingly. Comparator medication/placebo will be drawn up in a clouded syringe and recorded. The allocated concealed medications will be given to research team or ward staff to administer.

Stopping rule

Patients who develop a hypersensitivity, including anaphylactic reactions and angioedema should stop treatment and seek immediate medical advice. The trial should be informed.

If patients develop sign of a systemic infection with fever and chills, this should be discussed with the medical team. If the participant develops an open sore that might pre-dispose to infection, this should be discussed with the medical team.

No toxicity has been described with short term use and in fact, Anakinra has been used in acutely unwell patients with con-current infection and no adverse long-term effects.

The dosage of the drug cannot be modified for any reason.

8.1.9 Known drug reactions and interaction with other therapies

Concurrent administration of Anakinra and Etanercept has been associated with increased risk of serious infections and neutropenia compared to Etanercept alone in RA patients. Concurrent administration with other anti-TNF therapy is not advised. This should not occur as these patients are excluded from the trial.

Due to the anti-inflammatory nature of Anakinra, agents that use the CYP450 enzymes that are suppressed during inflammation may theoretically have altered metabolism due to resolution of

For peer review only - http://bmjopen.bmj.com/site/about/guidelines.xhtml

inflammation. Actions / levels of common drugs (Warfarin, Phenytoin) that use this pathway should ideally be monitored, however, no formal adverse events have been reported.

8.1.10 Concomitant medication

Patients should not receive concurrent immunosuppressive medication or biologics especially anti-TNF therapy.

Anakinra does has not been shown to accumulate even after prolonged (months) courses of treatment and should be cleared. The half-life 4-6 hours, patients could have other treatment after 7 days of randomisation.

Live vaccines should not be given con-currently.

Patients are not allowed Colchicine which has to be used in caution in patients with renal impairment due to risk of accumulation. Colchicine treatment will confound results and conclusion of the trial. Patients requiring analgesia can have Paracetamol, Tramadol and Codeine, but not within 4 hours of pain assessment.

8.1.11 Trial restrictions

There is inadequate information of Anakinra on pregnant and nursing mothers. Female participants of child-bearing age should continue to practice reliable means of contraception (such as physical barrier [patient and partner], contraceptive pill or patch, spermicide and barrier, or intrauterine devices) as stated in the exclusion criteria.

8.1.12 Assessment of compliance

Participants will be given diary cards at randomisation. Injection sites will be checked and patients will return syringes in a non-puncture container for empty syringes to be checked. Participants will also have visits at day 1, 2, 4, and 7 to check with compliance.

Patient will receive phone call to prompt injection and recording of diary cards to ensure feasibility trial meets criteria for success.

Documentation of injection sites and empty syringes will be undertaken by the trial investigator. Patients will be asked to bring non-puncture bins with empty syringes. Diary card will include time of injection.

Any non-compliance will be documented by the trial investigators and data will be recorded for study analysis, this is a key feature of the feasibility aspect of the trial. The percentage of noncompliance acceptable for patient to continue on the trial is <80% noncompliance equates to patient withdrawal (this includes compliance with IMP and study procedures e.g. visit window, refusal of study specific assessments). Patients who are withdrawn from the study for this reason will continue to be followed up.

Any omissions or delays will be documented in the eCRF to record the reason and information will be use by the Trial Management Committee to ensure success of the trial.

For peer review only - http://bmjopen.bmj.com/site/about/guidelines.xhtml

Health Research Authority

8.2.1 IMP: Intramuscular methylprednisolone (Depo-Medrone)

Glucocorticoids are adrenocortical steroids. Naturally occurring ones are hydrocortisone and cortisone are used for replacement in adrenocortical deficiency states.

It is indicated in a number of conditions when oral therapy is not feasible or the strength, dosage form, and route of administration of the drug lend the preparation to the treatment of the condition. It is used in all sorts of conditions for many years. It can be used for short-term administration for acute joint conditions.

It will only be used as an intra-muscular agent and so discussion will only be restricted to this. It will be used as a one off intra-muscular injection and although cautions described below generally apply to prolonged use, there is insufficient data on the short term one off use. We will monitor for any adverse events in our study.

Intramuscular methylprednisolone 40mg/ml (Methylprednisolone Acetate BP 40mg/mL) is an anti-inflammatory glucocorticoid for intramuscular, intra-articular, soft tissue or intra-lesion injection. Methylprednisolone acetate is a synthetic glucocorticoid. It has greater anti-inflammatory potency than prednisolone and less tendency than prednisolone to induce sodium and water retention.

Intramuscular methylprednisolone (Depo-Medrone) vials are intended for single use only and intra-muscular injection is used for sustained systemic effect; 40mg (1mL), 80mg (2mL) and 120ml (3mL). Dose must be individualised and depends on the condition being treated and its severity. Each mL contains methylprednisolone acetate, polyethylene glycol 3350 (29.5mg), polysorbate 80 (1.97mg), monobasic sodium phosphate (6.9mg), dibasic sodium phosphate USP (1.44mg), Benzyl alcohol as preservative 9.3mg. Sodium Chloride added to adjust tonicity.

It is manufactured by Pharmacia.

Licensed drug name: Depo-Medrone®

8.2.2 Legal status of the drug

It is a prescription only medication in the United Kingdom.

8.2.3 Summary of Product Characteristics (SmPC)

Available from http://www.medicines.org.uk/emc/medicine/3549

Issue date: 03/1989

Intramuscular methylprednisolone acetate is contra-indicated where there is known hypersensitivity to components and in systemic infection unless specific anti-infective therapy is employed

Following a single dose of intramuscular methylprednisolone, plasma cortisol levels are reduced and there is evidence of hypothalamic-pituitary-adrenal (HPA) axis suppression. This suppression lasts for a variable period of up to 4 weeks. The usual dynamic tests of HPA axis function can be used to diagnose evidence of impaired activity (e.g. Synacthen test).

Because rare instances of anaphylactic reactions have occurred in patients receiving parenteral corticosteroid therapy, appropriate precautionary measures should be taken prior to administration, especially when the patient has a history of drug allergy.

For peer review only - http://bmjopen.bmj.com/site/about/guidelines.xhtml

NHS Health Research Authority

Anakinra vs. Steroids for the Treatment of Gout Attacks in Renal Patients (ASGARD): A Feasibility Study

EudraCT no. 2015-001787-19

Corticosteroids may mask some signs of infection, and new infections may appear during their use. Suppression of the inflammatory response and immune function increases the susceptibility to fungal, viral and bacterial infections and their severity. The clinical presentation may often be atypical and may reach an advanced stage before being recognised.

Live vaccines should not be given to individuals with impaired immune responsiveness. The antibody response to other vaccines may be diminished.

The slower rate of absorption by intramuscular administration should be recognised.

Most special precaution relate to prolonged use of glucocorticoids

Particular care is required when considering the use of systemic corticosteroids in patients with osteoporosis (post-menopausal females are particularly at risk), hypertension or congestive heart failure, existing or previous history of severe affective disorders (especially previous steroid psychosis), diabetes mellitus (or a family history of diabetes), history of tuberculosis, glaucoma (or a family history of glaucoma), previous corticosteroid-induced myopathy, liver failure or cirrhosis, renal insufficiency, epilepsy, peptic ulceration, fresh intestinal anastomoses, predisposition to thrombophlebitis , abscess or other pyogenic infections, ulcerative colitis, diverticulitis, myasthenia gravis, ocular herpes simplex, for fear of corneal perforation, hypothyroidism.

Patients and/or carers should be warned that potentially severe psychiatric adverse reactions may occur with systemic steroids. Particular care is required when considering the use of systemic corticosteroids in patients with existing or previous history of severe affective disorders in themselves or in their first degree relatives. These would include depressive or manic-depressive illness and previous steroid psychosis.

The common adverse effects of systemic corticosteroids may be associated with more serious consequences in old age.

Adverse effects include anaphylactic reaction or allergic reactions, dyspepsia, peptic ulceration with perforation and haemorrhage, abdominal distension, oesophageal ulceration, oesophageal candidiasis, acute pancreatitis, perforation of bowel. Increases in alanine transaminase (ALT, SGPT) aspartate transaminase (AST, SGOT) and alkaline phosphatase have been observed following corticosteroid treatment. These changes are usually small, not associated with any clinical syndrome and are reversible upon discontinuation.

Proximal myopathy, osteoporosis, vertebral and long bone fractures, avascular osteonecrosis, tendon rupture, aseptic necrosis, muscle weakness. Sodium and water retention, potassium loss, hypertension, hypokalaemic alkalosis, congestive heart failure in susceptible patients.

Suppression of the hypothalamo-pituitary-adrenal axis, growth suppression in infancy, childhood and adolescence, menstrual irregularity and amenorrhoea. Cushingoid facies, hirsutism, weight gain, impaired carbohydrate tolerance with increased requirement for antidiabetic therapy, negative nitrogen and calcium balance. Increased appetite.

A wide range of psychiatric reactions including affective disorders (such as irritable, euphoric, depressed and labile mood psychological dependence and suicidal thoughts), psychotic reactions (including mania, delusions, hallucinations and aggravation of schizophrenia), behavioural disturbances, irritability, anxiety, sleep disturbances, and cognitive dysfunction including confusion and

For peer review only - http://bmjopen.bmj.com/site/about/guidelines.xhtml

Health Research Authority

Anakinra vs. Steroids for the Treatment of Gout Attacks in Renal Patients (ASGARD): A Feasibility Study

EudraCT no. 2015-001787-19

amnesia have been reported for all corticosteroids. Psychological effects have been reported on withdrawal of corticosteroids; the frequency is unknown.

Increased intra-ocular pressure, glaucoma, papilloedema, cataracts with possible damage to the optic nerve, corneal or scleral thinning, exacerbation of ophthalmic viral or fungal disease, exophthalmos.

Leucocytosis, hypersensitivity including anaphylaxis, thrombo-embolism, nausea, vertigo.

Pharmacodynamics and pharmacokinetics

One in-house study of eight volunteers determined the pharmacokinetics of a single 40 mg intramuscular dose of intramuscular methylprednisolone. The average of the individual peak plasma concentrations was 14.8 ± 8.6 ng/mL, the average of the individual peak times was 7.25 ± 1.04 hours, and the average area under the curve (AUC) was 1354.2 ± 424.1 ng/mL x hrs (Day 1-21).

Methylprednisolone is widely distributed into the tissues, crosses the blood-brain barrier, and is secreted in breast milk. Its apparent volume of distribution is approximately 1.4 L/kg. The plasma protein binding of methylprednisolone in humans is approximately 77%.

In humans, methylprednisolone is metabolized in the liver to inactive metabolites; the major ones are 20α-hydroxymethylprednisolone and 20β-hydroxymethylprednisolone. Metabolism in the liver occurs primarily via the CYP3A4.

Methylprednisolone, like many CYP3A4 substrates, may also be a substrate for the ATP-binding cassette (ABC) transport protein p-glycoprotein, influencing tissue distribution and interactions with other medicines.

The mean elimination half-life for total methylprednisolone is in the range of 1.8 to 5.2 hours. Total clearance is approximately 5 to 6 mL/min/kg.

No dosing adjustments are necessary in renal failure. Methylprednisolone is haemodialysable

8.2.4 Drug storage and supply

Shelf-life of the medicinal product as packaged for sale: 60 months. Intramuscular methylprednisolone acetate should not be mixed with any other fluid. Discard any remaining suspension after use. The nature of the container is a type I flint glass vial with a butyl rubber plug and metal seal. Each vial contains 1 ml, 2ml, or 3 ml of intramuscular methylprednisolone 40 mg/ml.

Disposal: After use patients will place syringe, without re-sheathing, into a puncture-resistant container (sharp bin). Syringes will be disposed as per normal hospital policy on disposal of sharps.

8.2.5 Preparation and labelling of Investigational Medicinal Product

The drug should be available from the local hospital pharmacy. It will be drawn up by a member of the research team not involved with the trial.

8.2.6 Dosage schedules

At recruitment and randomisation, participants enrolled into the intramuscular methylprednisolone arm will have a one off intramuscular methylprednisolone 120mg/3mL intra-muscular injection. Patients in the other arm for investigation product will have similar regime with the equivalent placebo.

For peer review only - http://bmjopen.bmj.com/site/about/guidelines.xhtml

Health Research Authority

Anakinra vs. Steroids for the Treatment of Gout Attacks in Renal Patients (ASGARD): A Feasibility Study

EudraCT no. 2015-001787-19

8.2.7 Dosage modifications

There will be no dosage modification.

8.2.8 Drug Accountability

Drug accountability will be by the local pharmacy.

Documentation

Accurate records of all drug shipments, medication dispensed, and all drug returned, must be maintained. The inventory record must be available for inspection by the sponsor but information that may lead to un-blinding will be minimised. The site PI or CI does not destroy any unused drug unless directed to by the sponsor. Any destroyed drug must be destroyed according to hospital procedures and properly accounted for. At the conclusion of the study, the overall numbers of the drug used at the centre, and the number destroyed or returned, will be provided by the pharmacy, and an account given of any discrepancy.

Dispensing procedure

Once a research team has undertaken randomisation for a patient after recruitment, they will inform pharmacy. Pharmacy will be given the allocation details and will prepare appropriate medication or placebo accordingly. Comparator medication/placebo will be drawn up in a clouded syringe and recorded. The allocated concealed medications will be given to research team or ward staff to administer.

Stopping rule

Patients who develop a hypersensitivity, including anaphylactic reactions and angioedema should stop treatment and seek immediate medical advice. The trial should be informed.

If patients develop sign of a systemic infection with fever and chills, this should be discussed with the medical team. If the participant develops an open sore that might pre-dispose to infection, this should be discussed with the medical team.

8.2.9 Known drug reactions and interaction with other therapies

- Drugs that induce hepatic enzymes, such as rifampicin, rifabutin, carbamazepine, phenobarbitone, phenytoin, primidone, and aminoglutethimide enhance the metabolism of corticosteroids and its therapeutic effects may be reduced.
- Drugs such as erythromycin and ketoconazole may inhibit the metabolism of corticosteroids and thus decrease their clearance.
- Steroids may reduce the effects of anticholinesterases in myasthenia gravis. The desired effects of hypoglycaemic agents (including insulin), anti-hypertensives and diuretics are antagonised by corticosteroids, and the hypokalaemic effects of acetazolamide, loop diuretics, thiazide diuretics and carbenoxolone are enhanced.
- The efficacy of coumarin anticoagulants may be enhanced by concurrent corticosteroid therapy and close monitoring of the INR or prothrombin time is required to avoid spontaneous bleeding.
- The renal clearance of salicylates is increased by corticosteroids and steroid withdrawal may result in salicylate intoxication. Salicylates and non-steroidal anti-inflammatory agents should be used cautiously in conjunction with corticosteroids in hypothrombinaemia.

For peer review only - http://bmjopen.bmj.com/site/about/guidelines.xhtml

- Steroids have been reported to interact with neuromuscular blocking agents such as pancuronium with partial reversal of the neuromuscular block.

8.2.10 Concomitant medication

Patients should not receive concurrent immunosuppressive medication. Live vaccines should not be given con-currently. Caution should be used when using medications described in section 8.2.9.

8.2.11 Trial restrictions

Steroids should be ideally avoided during pregnancy. Female participants of child-bearing age should continue to practice reliable means of contraception (such as physical barrier [patient and partner], contraceptive pill or patch, spermicide and barrier, or intrauterine devices) as stated in the exclusion criteria.

8.2.12 Assessment of compliance

Participants will be given the single dose of intra-muscular injection on Day 1.

8.3.1 IMP: Lipofundin MCT/LCT 10%

Lipofundin MCT/LCT 10% is an emulsion preparation for injection. It is composed of soya oil. Medium-chain Triglycerides, glycerol, egg lecithin, alpha tocopherol (vitamin E), sodium oleate. These agents are commonly used as carriers for fat soluble drugs. Lipofundin MCT/LCT specifically is used for the provision of calories and essential fatty acids. It has been used as a control for other intramuscular injection steroid emulsion injection in contemporary studies. It is normally used in amounts of over 100mls and the side-effects and contra-indications are for these form of administration. We are using this agent as an appropriate placebo for intramuscular methylprednisolone. This is to keep the appearance of the intramuscular methylprednisolone and its placebo the same. The volume used for injection will be 3mL, the same as the volume of intramuscular methylprednisolone. We do not anticipate any side-effects with this amount. There should not be any biological effects with this amount and there are no active agents in the preparation.

8.3.2 Legal status of the drug

Prescription only medication.

8.3.2 Summary of Product Characteristics (SmPC)

Emulsion for injection in glass bottle, contents 100mL, 250mL and 500mL.

8.3.4 Drug storage and supply

Store below 25°C and protect from freezing. If accidentally frozen, discard bottle. Unused contents must be discarded and should not be stored for later use. Do not use bottles showing evidence of phase separation.

8.3.5 Preparation and labelling of Investigational Medicinal Product

The drug will be drawn up for injection by a member of the research team who is not involved with the study.

8.3.6 Dosage schedules

For peer review only - http://bmjopen.bmj.com/site/about/guidelines.xhtml

Health Research Authority

Anakinra vs. Steroids for the Treatment of Gout Attacks in Renal Patients (ASGARD): A Feasibility Study

EudraCT no. 2015-001787-19

3mL will be injected as an intra-muscular injection on Day 1.

8.3.7 Dosage modifications

Lipofundin MCT/LCT is used as a placebo injection for the purpose of this study. It is not being used for intravenous feeding that it would normally be used for.

8.3.8 Drug Accountability

Drug accountability will be by the local pharmacy.

Documentation

Accurate records of all medication dispensed, and all drug returned, must be maintained. The inventory record must be available for inspection by the sponsor but information that may lead to un-blinding will be minimised. The site PI or CI does not destroy any unused drug unless directed to by the sponsor. Any destroyed drug must be destroyed according to hospital procedures and properly accounted for. At the conclusion of the study, the overall numbers of the drug used at the centre, and the number destroyed or returned, will be provided by the pharmacy, and an account given of any discrepancy.

Dispensing procedure

Once a research team has undertaken randomisation for a patient after recruitment, they will inform pharmacy. Pharmacy will be given the allocation details and will prepare appropriate medication or placebo accordingly. Comparator medication/placebo will be drawn up in a clouded syringe and recorded. The allocated concealed medications will be given to research team or ward staff to administer.

Stopping rule

Patients who develop a hypersensitivity, including anaphylactic reactions and angioedema should stop treatment and seek immediate medical advice. The trial should be informed.

Documentation

Accurate records of all drug shipments, medication dispensed, and all drug returned, must be maintained. The inventory record must be available for inspection by the sponsor but information that may lead to un-blinding will be minimised. The site PI or CI does not destroy any unused drug unless directed to by the sponsor. Any destroyed drug must be destroyed according to hospital procedures and properly accounted for. At the conclusion of the study, the overall numbers of the drug shipped to the centre, and the number destroyed or returned, will be provided by the pharmacy, and an account given of any discrepancy.

Dispensing procedure

Once a research team has undertaken randomisation for a patient after recruitment, they will inform pharmacy. Pharmacy will be given the allocation details and will prepare appropriate medication or placebo accordingly. Comparator medication/placebo will be drawn up in a clouded syringe and recorded. The allocated concealed medications will be given to research team or ward staff to administer.

For peer review only - http://bmjopen.bmj.com/site/about/guidelines.xhtml

8.3.9 Assessment of compliance

Participants will be given intra-muscular injection on Day 1.

8.4 Name and description of each Non-Investigational Medicinal Product (NIMP)

A pre-specified list of analgesics can be used in participants or potential participants. This will avoid inadvertent treatment of patients with potentially harmful agents such as NSAIDS or Colchicine, it would also allow patients to be given the opportunity to participate in the trial. These analgesics should not be used within four hours of project assessment of pain. Patients who are primed for the study can use these agents at home before presenting to secondary care.

8.4.1 NIMP: Paracetamol

Paracetamol is the most commonly used and widely used analgesic in the UK. It is used for the relief of mild to moderate pain. It also has antipyretic actions and can be used in febrile conditions. It can be given 500mg to 1g every four to six hourly as required. It is usually administered orally but can be administered intravenously as well.

It is contraindication in patients with known hypersensitivity to paracetamol or other constituents in the tablets. It should not be taken with other paracetamol-containing products. Immediate medical attention should be sought in the event of an overdose.

There are few interactions with other medicinal products but it is only for short-term use in this study. Metoclopramide and Domperidone may increase speed of absorption of paracetamol. Cholestyramine may reduce adsorption if given within one hour of paracetamol.

Paracetamol should be avoided in pregnancy but no adverse effects have been shown when used in the recommended dosage.

Adverse effects of Paracetamol are rare but hypersensitivity including skin rash may occur. There have been reports of blood dyscrasias including thrombocytopenia, neutropenia, pancytopenia, leukopenia and agranulocytosis but these were not necessarily causality related to Paracetamol.

Paracetamol is readily absorbed from the gastrointestinal tract. Peak plasma concentrations occur about 10 to 60 minutes after oral doses. Paracetamol is distributed into most body tissues. It crosses the placenta and is present in breast milk. Plasma-protein binding is negligible at usual therapeutic concentrations but increases with increasing concentrations. It is metabolised in the liver. A minor hydroxylated metabolite which is usually produced in very small amounts by mixed-function oxidases in the liver and which is usually detoxified by conjugation with liver glutathione may accumulate following paracetamol overdose and cause tissue damage. It is excreted in the urine, mainly as the glucuronide and sulphate conjugates. The elimination half-life varies from about 1 to 4 hours.

Shelf-life is for three years for PE tablet containers and five years for blisters and PP tablet containers. It should be stored below 25°C in a dry place. It should be protected from light.

8.4.2 NIMP: Codeine Phosphate

Codeine phosphate as an analgesic for the relief of mild to moderate pain and for acute moderate pain which is not considered to be relieved by other analgesics such as paracetamol. Codeine should be used at the lowest effective dose for the shortest period of time. This dose may be taken, up to 4 times

For peer review only - http://bmjopen.bmj.com/site/about/guidelines.xhtml

a day at intervals of not less than 6 hours. Maximum daily dose should not exceed 240mg. 30-60mg every four hours to a maximum dosage of 240mg daily.

Codeine is an analgesic with uses similar to those of morphine but it is much less potent as an analgesic and has only mild sedative effects. It is also used in the treatment of cough and diarrhoea.

Codeine is a centrally acting weak analgesic. Codeine exerts its effect through μ opioid receptors, although codeine has low affinity for these receptors, and its analgesic effect is due to its conversion to morphine. Codeine, particularly in combination with other analgesics such as paracetamol, has been shown to be effective in acute nociceptive pain.

The analgesic effect is not materially enhanced by increasing the dose to a greater level than that recommended above. Dosage should be reduced in the elderly where there is impairment of hepatic or renal function. It is only intended for oral use.

Contraindications to use of codeine phosphate include known hypersensitivity to codeine, other opioids or any of the excipients in the tablets, acute respiratory depression, severe obstructive airways disease (e.g. emphysema), asthma (should not be administered during an asthma attack), hepatic failure, head injuries or conditions where intracranial pressure is raised, acute alcoholism, risk of paralytic ileus and women during breastfeeding.

Patient with metabolic derangements such as those whom it is known they are CYP2D6 ultra-rapid metabolisers or with those rare hereditary problems of galactose intolerance, the Lapp lactase deficiency or glucose-galactose malabsorption should not take this medicine.

Codeine phosphate should be used with caution if there is a possible risk of CNS excitation or depression, convulsions, or a history of drug abuse or dependence (including alcoholism). It should be used with caution in patients with hepatic impairment, renal impairment, hypothyroidism, inflammatory bowel disease (codeine reduces peristalsis, increases tone and segmentation in the bowel and can raise colonic pressure, therefore should be used with caution in diverticulitis, acute colitis, diarrhoea associated with pseudomembranous colitis or after bowel surgery), gall bladder disease or gall stones (opioids may cause biliary contraction and should be avoid in biliary disorders), gastro-intestinal surgery, urinary tract surgery (following recent surgery patients will be more prone to urinary retention caused directly by spasm of the urethral sphincter, and via constipation caused by codeine).

It should be used with caution is patient with a history of phaeochromocytoma (opioids may stimulate catecholamine release by inducing the release of endogenous histamine), prostatic hypertrophy, adrenocortical insufficiency, e.g. Addison's disease, hypotension and shock, myasthenia gravis, reduced respiratory function or history of asthma, pregnancy and breast feeding.

Elderly patients may metabolise and eliminate opioid analgesics more slowly than younger patients Codeine is metabolised by the liver enzyme CYP2D6 into morphine, its active metabolite. If a patient has a deficiency or is completely lacking this enzyme an adequate analgesic effect will not be obtained. If the patient is an extensive or ultra-rapid metaboliser there is an increased risk of developing side effects of opioid toxicity even at commonly prescribed doses. These patients convert codeine into morphine rapidly resulting in higher than expected serum morphine levels.

General symptoms of opioid toxicity include confusion, somnolence, shallow breathing, small pupils, nausea, vomiting, constipation and lack of appetite. In severe cases this may include symptoms of circulatory and respiratory depression, which may be life-threatening and very rarely fatal.

For peer review only - http://bmjopen.bmj.com/site/about/guidelines.xhtml

Interactions

- MAOIs (e.g. linezolid, moclobemide, selegiline) - is not recommended due to the possible risk of excitation or depression, avoid concomitant use and for 2 weeks after discontinuation of MAOI
- Alcohol – enhanced sedative and hypotensive effect, and increased risk of respiratory depression.
- Sedative antihistamines, hypnotics and anxiolytics - enhanced risk of sedative effect, and increased risk of respiratory depression
- Anticholinergics (e.g. atropine) - risk of severe constipation which may lead to paralytic ileus, and /or urinary retention
- Metoclopramide and domperidone – antagonise effect on GI activity
- Anti-diarrhoeal drugs (e.g. loperamide, kaolin) – increased risk of severe constipation.
- Anaesthetics - enhanced sedative and hypotensive effect
- Tricyclic antidepressants - enhanced sedative effect
- Antipsychotics - enhanced sedative and hypotensive effect
- Opioid antagonists e.g. buprenorphine, naltrexone, naloxone – may precipitate withdrawal symptoms
- Quinidine- reduced analgesic effect
- Antihypertensive drugs - enhanced hypotensive effect.
- Ciprofloxacin, - avoid premedication with opioids as they reduce plasma ciprofloxacin concentration.
- Ritonavir may increase plasma levels of opioid analgesics such as codeine.
- Mexiletine - delayed absorption of mexiletine.
- Cimetidine inhibits the metabolism of opioid analgesics causing increased plasma concentration of codeine.

Pregnancy and lactation

Risk benefit must be considered because opioid analgesics cross the placenta. Regular use during pregnancy may cause physical dependence in the foetus, leading to withdrawal symptoms in the neonate. During labour opioids enter the foetal circulation and may cause respiratory depression in the neonate. Respiratory malformation in neonates may be associated with exposure to codeine during pregnancy. Gastric stasis and a risk of inhalation pneumonia could occur in the mother during labour. Administration should be avoided during the late stages of labour and during the delivery of a premature infant. Codeine should not be used during breastfeeding.

Adverse effects

- Immune system disorders: (may be caused by histamine release) – including rash, urticaria, pruritus, difficulty breathing, increased sweating, redness or flushed face.
- Nervous system disorders: confusion, drowsiness, malaise, tiredness, vertigo, dizziness, changes in mood, hallucinations, CNS excitation (restlessness/excitement), convulsions, mental depression, headache, or nightmares, raised intracranial pressure, tolerance or dependence, dysphoria, hypothermia.
- Eye disorders: - miosis, blurred or double vision.
- Cardiac disorders: bradycardia, palpitations, hypotension, orthostatic hypotension, tachycardia.
- Respiratory, thoracic and mediastinal disorders: respiratory depression with larger doses.

For peer review only - http://bmjopen.bmj.com/site/about/guidelines.xhtml

- Gastrointestinal disorders: constipation (too constipating for long-term use), biliary spasm, nausea, vomiting, dry mouth.
- Musculoskeletal, connective tissue and bone density: muscle rigidity.
- Renal and urinary disorders: ureteral spasm, antidiuretic effect, urinary retention.
- Reproductive system and breast disorders: decrease in libido and potency.
- Withdrawal effects: abrupt withdrawal precipitates a withdrawal syndrome. Symptoms may include tremor, insomnia, restlessness, irritability, anxiety, depression, anorexia, nausea, vomiting, diarrhoea, sweating, lacrimation, rhinorrhoea, sneezing, yawning, piloerection, mydriasis, weakness, pyrexia, muscle cramps, dehydration, and increase in heart rate, respiratory rate and blood pressure. NOTE - tolerance diminishes rapidly after withdrawal so a previously tolerated dose may prove fatal.
- The effects in overdose will be potentiated by simultaneous ingestion of alcohol and psychotropic drugs. Central nervous system depression, including respiratory depression, may develop but is unlikely to be severe unless other sedative agents have been co-ingested, including alcohol, or the overdose is very large. The pupils may be pin-point in size; nausea and vomiting are common. Hypotension and tachycardia are possible but unlikely. Management consists of general symptomatic and supportive measures including a clear airway and monitoring of vital signs until stable. Consider activated charcoal if an adult presents within one hour of ingestion of more than 350mg.
- Give naloxone if coma or respiratory depression is present. Naloxone is a competitive antagonist and has a short half-life so large and repeated doses may be required in a seriously poisoned patient. Observe for at least four hours after ingestion, or eight hours if a sustained release preparation has been taken.

8.4.3 NIMP: Co-Codamol

Co-codamol 8/500mg tablets contain codeine phosphate (8mg) and paracetamol (500mg). The cautions should be exercised as above. The amount of codeine is less and the expected side-effect is less.

8.4.4 NIMP: Co-Drydramol

Co-drydramol consists of Paracetamol (500mg) and Dihydrocodiene Tartrate (10mg). It is used for relief of mild to moderate pain. Dihydrocodiene is a member of the codeine family of opioid analgesics metabolised is a similar manner to codeine i.e. morphine and norcodiene. The same cautions use for codeine should be applied here. Co-drydramol is not under the same restrictions as codeine phosphate and co-codamol, both of which are on the list of controlled drugs under the misuse of drugs legislation. Co-drydramol is more readily dispensed by emergency departments and acute medical units.

8.4.5 NIMP: Tramadol

Tramadol can be used for the treatment of moderate to severe pain. The dose should be adjusted to the intensity of the pain and the sensitivity of the individual patient. The lowest effective dose for analgesia should generally be selected. The capsules are taken orally, independent of meals, swallowed whole with water. The dose is 50-100mg 3-4 times daily. A total daily dose of 400mg should not be exceeded.

Tramadol is a centrally acting synthetic analgesic compound. It is a non-selective pure agonist at mu, delta and kappa opioid receptors with a higher affinity for the mu receptor. Tramadol opioid activity

For peer review only - http://bmjopen.bmj.com/site/about/guidelines.xhtml

derives from low affinity binding of the parent compound to mu-opioid receptors and higher affinity binding of the active metabolite, O-desmethyl tramadol. Compared to morphine, tramadol does not show respiratory depression when given within the analgesic dosage interval. The gastrointestinal motility is not affected. There is minimal effect on the cardiovascular system. The contribution to human analgesia of tramadol relative to the active metabolite is unknown. Tramadol has an antitussive effect. Animal studies have revealed a reduced dependence potential compared with morphine and a very slight tolerance potential.

Tramadol should under no circumstances be administered for longer than absolutely necessary. If long-term pain treatment with tramadol is necessary in view of the nature and severity of the illness, then careful and regular monitoring should be carried out (if necessary with breaks in treatment) to establish whether and to what extent further treatment is necessary.

Caution should be used in patients over the age of 75. Drug elimination may be prolonged in patients with renal impairment and a prolongation of the dosage intervals should be carefully considered according to the patient's requirements. For patients with creatinine clearance <30ml/min, the dosage interval should be increased to 12 hours. Tramadol is not recommended in patients with severe renal impairment (creatinine clearance <10ml/min). Tramadol is removed very slowly by haemodialysis or haemofiltration so post-dialysis administration to maintain analgesia is not usually necessary.

Drug elimination of tramadol may also be prolonged in patients with hepatic impairment. The usual dosage should be divided in 2, or the dosage interval should be extended to 12 hours. In these patients prolongation of the dosage intervals should be carefully considered according to the patient's requirements. In severe hepatic impairment, the product is contraindicated.

Tramadol is contraindicated in patients who previously demonstrated hypersensitivity to tramadol or any of the other ingredients in the capsule, patients who have acute intoxication with central nervous system depressants (alcohol, hypnotics, centrally acting analgesics, opioids, psychotropic drugs), patients receiving monoamine oxidase inhibitors or within two weeks of their withdrawal, patients with severe hepatic impairment, severely impaired kidney function (creatinine clearance less than 10ml/min), severe respiratory impairment and with epilepsy not controlled by adequate treatment. It should not be used in opioid-dependent patients.

It should be use with caution in patients with withdrawal symptoms, patients with a history of drug dependence and abuse, patients sensitive to opiates, patients with head injury, increased intracranial pressure, impairment of hepatic and renal function, decreased level of consciousness and in patients prone to convulsive disorder or in shock. Caution should be used in treating patients with respiratory depression, or if concomitant CNS depressant drugs are being administered.

Interactions

- Concomitant use of carbamazepine or concomitant intake of alcohol with tramadol is not recommended
- Buprenorphine and other mixed agonists-antagonists, naltrexone
- Caution with the use of psych-active medicines or substances including alcohol
- Tramadol should not be combined with MAO inhibitors. In patients treated with MAO inhibitors in the 14 days prior to the use of the opioid pethidine, life threatening interactions on the central nervous system, respiratory and cardiovascular function have been observed. The same interactions with MAO inhibitors cannot be ruled out during treatment with Tramadol.

For peer review only - http://bmjopen.bmj.com/site/about/guidelines.xhtml

Health Research Authority

Anakinra vs. Steroids for the Treatment of Gout Attacks in Renal Patients (ASGARD): A Feasibility Study

EudraCT no. 2015-001787-19

- Simultaneous or previous administration of carbamazepine (enzyme inducer) may reduce the analgesic effect and shorten the duration of action.
- The combination with mixed agonist/antagonists (e.g. buprenorphine, nalbuphine, pentazocine) and tramadol is not advisable, because the analgesic effect of a pure agonist may be theoretically reduced in such circumstances.
- Tramadol can induce convulsions and increase the potential for selective serotonin re-uptake inhibitors, (SSRIs), serotonin-norepinephrine reuptake inhibitors (SNRIs), tricyclic anti-depressants, anti-psychotics and other seizure threshold lowering medicinal products (such as bupropion, mirtazapine, tetrahydrocannabinol) to cause convulsions.
- Concomitant therapeutic use of tramadol and serotonergic drugs, such as selective serotonin reuptake inhibitors (SSRIs), serotonin-norepinephrine reuptake inhibitors (SNRIs), MAO inhibitors, tricyclic antidepressants and mirtazapine may cause serotonin toxicity. Signs of serotonin syndrome may be for example confusion, agitation, fever, sweating, ataxia, hyperreflexia, myoclonus and diarrhoea.
- Caution should be exercised during concomitant treatment with tramadol and coumarin derivatives (e.g. warfarin) due to reports of increased INR with major bleeding and ecchymosis in some patients.
- Other active substances known to inhibit CYP3A4, such as ketoconazole and erythromycin, might inhibit the metabolism of tramadol (N-demethylation) probably also the metabolism of the active O-demethylated metabolite.
- Tramadol crosses the placenta. There is inadequate evidence available on the safety of tramadol in human pregnancy. Therefore tramadol should not be used in pregnant women. Tramadol is not recommended during breast-feeding. After a single administration of tramadol it is not usually necessary to interrupt breast-feeding.

Adverse reactions

- Frequent (1/10 to 1/100)
  - Nausea and dizziness
  - Headache, somnolence
- Common (1/100 to 1/1000)
  - Changes in appetite, paraesthesia, tremor, respiratory depression, epileptiform convulsions, involuntary muscle contractions, abnormal coordination, syncope
  - Palpitation, tachycardia, postural hypotension or cardiovascular collapse) uncommon
  - Retching; gastrointestinal irritation (a feeling of pressure in the stomach, bloating), diarrhoea
  - Vomiting, constipation, dry mouth
  - Sweating
  - Fatigue
- Rare (1/1000 to 1/10000)
  - Bradycardia and increase in blood pressure rare.
  - Hallucinations, confusion, sleep disturbance, anxiety and nightmares.
  - Changes in mood (usually elation, occasionally dysphoria), changes in activity (usually suppression, occasionally increase) and changes in cognitive and sensorial capacity (e.g. decision behaviour, perception disorders).
  - Blurred vision
  - Dyspnoea
  - Dermal reactions (e.g. pruritus, rash, urticaria)
  - Micturition disorders (difficulty in passing urine, dysuria and urinary retention)

For peer review only - http://bmjopen.bmj.com/site/about/guidelines.xhtml

    - Motor weakness
    - Allergic reactions (e.g. dyspnoea, bronchospasm, wheezing, angioneurotic oedema) and anaphylaxis
- Very rare (<1/10000)
    - Deranged liver enzymes (isolated cases).
- Symptoms of overdose are typical of other opioid analgesics and include miosis, vomiting, cardiovascular collapse, sedation and coma, seizures and respiratory depression. Supportive measures such as maintaining the patency of the airway and maintaining cardiovascular function should be instituted; naloxone should be used to reverse respiratory depression; fits can be controlled with diazepam. In case of intoxication orally, gastrointestinal decontamination with activated charcoal or by gastric lavage is only recommended within 2 hours after tramadol intake. Gastrointestinal decontamination at a later time point may be useful in case of intoxication with exceptionally large quantities.
- Symptoms of withdrawal reactions, similar to those occurring during opiate withdrawal, may occur as follows: agitation, anxiety, nervousness, insomnia, hyperkinesia, tremor and gastrointestinal symptoms. Other symptoms that have very rarely been seen with tramadol discontinuation include: panic attacks, severe anxiety, hallucinations, paraesthesia, tinnitus and unusual CNS symptoms.

For peer review only

For peer review only - http://bmjopen.bmj.com/site/about/guidelines.xhtml

9 PHARMACOVIGILANCE

9.1 Definitions

The Medicines for Human Use (Clinical Trials) Regulations 2004 (UK), as amended, provides the following definitions relating to adverse events in trials with an investigational medicinal product (table above).

Southend University Hospital will undertake all sponsor duties and pharmacovigilance. The Investigator is responsible for ensuring that all adverse events occurring during the study treatment period and 8 weeks after the first administration of study drugs (Anakinra and intramuscular methylprednisolone), must be recorded on the Adverse Event Case Report Form (AE CRF).

Term	Definition
**Adverse Event (AE)**	Any untoward medical occurrence in a participant to whom a medicinal product has been administered, including occurrences which are not necessarily caused by or related to that product.
**Adverse Reaction (AR)**	An untoward and unintended response in a participant to an investigational medicinal product which is related to any dose administered to that participant.<br><br>The phrase "response to an investigational medicinal product" means that a causal relationship between a trial medication and an AE is at least a reasonable possibility, i.e. the relationship cannot be ruled out.<br><br>All cases judged by either the reporting medically qualified professional or the Sponsor as having a reasonable suspected causal relationship to the trial medication qualify as adverse reactions.
**Serious Adverse Event (SAE)**	A serious adverse event is any untoward medical occurrence that:<br>• results in death<br>• is life-threatening<br>• requires inpatient hospitalisation or prolongation of existing hospitalisation<br>• results in persistent or significant disability/incapacity<br>• consists of a congenital anomaly or birth defect<br><br>Other 'important medical events' may also be considered serious if they jeopardise the participant or require an intervention to prevent one of the above consequences.<br><br>NOTE: The term "life-threatening" in the definition of "serious" refers to an event in which the participant was at risk of death at the time of

For peer review only - http://bmjopen.bmj.com/site/about/guidelines.xhtml

Health Research Authority

	the event; it does not refer to an event which hypothetically might have caused death if it were more severe.
**Serious Adverse Reaction (SAR)**	An adverse event that is both serious and, in the opinion of the reporting Investigator, believed with reasonable probability to be due to one of the trial treatments, based on the information provided.
**Suspected Unexpected Serious Adverse Reaction (SUSAR)**	A serious adverse reaction, the nature and severity of which is not consistent with the information about the medicinal product in question set out:<br>• in the case of a product with a marketing authorisation, in the summary of product characteristics (SmPC) for that product<br>• in the case of any other investigational medicinal product, in the investigator's brochure (IB) relating to the trial in question

NB: to avoid confusion or misunderstanding of the difference between the terms "serious" and "severe", the following note of clarification is provided: "Severe" is often used to describe intensity of a specific event, which may be of relatively minor medical significance. "Seriousness" is the regulatory definition supplied above.

9.2 Operational definitions for (S) AEs

9.2.1 Investigator Assessment

At each contact with the subject during the treatment period, the investigator must seek information on adverse events by specific questioning and, as appropriate, by examination. Information elicited should be recorded immediately in the source document and the AE CRF. All clearly related signs, symptoms, and abnormal diagnostic procedures should be recorded using the NCI CTCAE v4.0 event terms and grading. The clinical course of each event should be followed until resolution or stabilisation.

The use of Anakinra is in this trial is outside of the licensed indication. Hence, all AR are required to be reported and certain AEs will need to be reported. Previous AEs reported are neutropenia and infection. Other previously described adverse reactions include signs at the injection site such as erythema, pruritus and rash. Adverse reactions in the control arm to be examined for included high blood sugars/uncontrolled diabetes and clinical signs of fluid retention.

Investigators will make assessments on:

- Seriousness – the CI/PI responsible for the care of the patients, or in his absence an authorised medic with the research team, is responsible for assessing whether the event is serious according to the definitions given section 9.1.
- Causality – The investigator must assess causality of all serious adverse events/reactions in relation to the trial treatment according to the definition given. If the SAE is assessed as having a reasonable causal relationship, then it is defined as a SAR.

For peer review only - http://bmjopen.bmj.com/site/about/guidelines.xhtml

- Expectedness – The investigator must assess the expectedness of all SARs according to the definition given. If the SAR is unexpected, then it is a SUSAR.
- Severity –The intensity of an event should not be confused with the term "serious" which is a regulatory definition based on patient/event outcome criteria. The investigator must assess the severity of the vent according to the following terms and assessments.
  - Mild: Some discomfort noted but without disruption of daily life
  - Moderate: Discomfort enough to affect/reduce normal activity
  - Severe: Complete inability to perform daily activities and lead a normal life

9.2.2 Pre-existing Conditions

Pre-existing conditions should not be reported as AE unless the condition worsens by at least CTCAE grade during trial. The condition must be reported in the pre-treatment section of the CRF, if symptomatic at the time of entry, or under concurrent medical conditions if asymptomatic.

9.2.3 Exceptions to (S) AE or (S) AR reporting

Many clinical events are likely to occur given the co-morbid burden of the participant group. Events that are recognised and expected complications of the condition are exempt from normal reporting procedure, unless they are of an unexpected severity. These would not be considered to be SUSARs unless the severity of the event was considered to be unexpected. Expected adverse events in this patient consist of worsening:

- Complications of chronic kidney disease
- Heart failure
- Hypertension
- Diabetes complications
- Underlying cardiac disease with complications such as ACS, arrhythmia, hypotension, tachycardia and worsening oedema.

Other exceptions to (S) AE or (S) AR reporting consist of:

- Routine treatment or monitoring of the studied indication not associated with any deterioration in condition.
- Associated with any deterioration in condition, e.g. pre-planned hip replacement operation which does not lead to further complications.
- Any admission to hospital or other institution for general care where there was no deterioration in condition.
- Treatment on an emergency, outpatient basis for an event **not** fulfilling any of the definitions of serious as given above and not resulting in hospital admission.
- Any death or hospitalisation due to fall or fracture
- Any death or hospitalisation due to exacerbation of an existing medical co-morbid condition

In all cases AEs and / or laboratory abnormalities that are critical to the safety evaluation of the participant must be reported to the Sponsor; these may be volunteered by the participant, discovered by the investigator questioning or detected through physical examination, laboratory test or other investigation. Where certain AEs are not required to be reported to the Sponsor, these should still be recorded in the participant's medical records.

9.2.4 Recording and reporting of AE or AR

For peer review only - http://bmjopen.bmj.com/site/about/guidelines.xhtml

The IMP reference documentation that is used for pharmacovigilance purposes is used to assess the causality and expectedness of events and will be checked by the Sponsor for changes on the anniversary of the CTA.

Reporting of AEs / SAEs start at the point of consent. Reporting for ARs / SARs and SUSARs start at first IMP dose. If the AE or AR is not defined as serious, it is recorded in the study file and the participant is followed up by the research team. The AE/AR is documented in the participants' medical notes (where appropriate) and AE CRF. **All reactions should be reported and will be reviewed by the sponsor/CI as this is a CTIMP trial using licensed drug outside of the licensed indication even though some class evidence is available.**

9.3 Recording and reporting of SAEs AND SUSARs

All **SAEs* / SUSARs** occurring from the time of **start of trial treatment** until 7 days post cessation of trial treatment must be recorded on the SAE form/AECRF Form and faxed to the Sponsor **within 24 hours** of the research staff becoming aware of the event. Once all resulting queries have been resolved, the Sponsor will request the original form should also be posted to the Sponsor and a copy to be retained on site.

A completed SAE/SUSAR Report Form for all events should be faxed, or scanned and e-mailed, to:

Dr G Balasubramaniam, Southend University Hospital.

E-mail: g.balasubramaniam@southend.nhs.uk

Tel: 07843563739 (mobile); 01702 435555 (work switchboard)

Fax: 01702 508595

E-mail should also be sent to arctu@anglia.ac.uk to initiate notification and follow-up monitoring.

For each **SAEs* / SUSARs*** the following information will be collected:

- full details in medical terms and case description
- event duration (start and end dates, if applicable)
- action taken
- outcome
- seriousness criteria
- causality (i.e. relatedness to trial drug / investigation), in the opinion of the investigator
- Whether the event would be considered expected or unexpected.

Any change of condition or other follow-up information should be faxed to the Sponsor as soon as it is available or at least within 24 hours of the information becoming available. Events will be followed up until the event has resolved or a final outcome has been reached.

All SAEs assigned by the PI or delegate (or following central review) as both suspected to be related to IMP-treatment and unexpected will be classified as SUSARs and will be subject to expedited reporting to the Medicines and Healthcare Products Regulatory Agency (MHRA). The

For peer review only - http://bmjopen.bmj.com/site/about/guidelines.xhtml

Sponsor will inform the MHRA, the REC and the Sponsor of SUSARs within the required expedited reporting timescales.

9.3.1 Recording and reporting of SAE

All SAEs will be recorded in the participants' notes, the CRF, the sponsor AECRF form and reported to the sponsor/ARCTU/IMP provider within 24 hours of CI or PI or co-investigators becoming aware of the event (unless SAE/SAR/SUSAR is specified as not requiring immediate reporting). Nominated co-investigators will be authorised to sign the SAE forms in the absence of the CI at the co-ordinating site or the PI at the participating sites.

A complete SAE form for all events requiring immediate reporting should be faxed, or scanned and e-mailed, to SUH. These reports should be followed by further detailed SAE/SUSAR reports until resolution of event.

9.3.2 Follow-up and Post-Study SAEs

The active monitoring period starts from the first dose of the IMP to two days after the last dose of the IMP i.e. 7 days. This is based on a half-life of 4 hours. The reporting requirement for SAEs affecting subjects applies for all events occurring up to 8 weeks after the last administration of study drugs, which coincides with last follow-up. All unresolved adverse events should be followed up by the Principle Investigator until the events are resolved.

Where a participant withdraws consent for further processing of data, this does not preclude the reporting of SARs and SUSARs which are required to continue being reported according to the protocol for regulatory purposes. The PIS should include a section explaining this to the participant.

9.3.3 Recording and reporting of SUSAR

All SUSARs that occur during the trial will be reported to the sponsor/ main REC/IMP provider/ARCTU (if applicable) within 24 hours of the CI or co-investigator becoming aware of the event. SUSARs should be reported to the sponsor/ARCTU within 24 hours as the sponsor has a legal obligation to report this to the MHRA within 7 days (for fatal or life-threatening SUSARs) or 15 days for all other SUSARs. In the case of multicentre studies, the PI or the co-investigators at the participating site must inform the CI within 24 hours of the event. The CI or co-investigators at the co-ordinating site must inform the sponsor/ARCTU immediately to allow reporting to the MHRA within the allocated timelines. The CI will need to complete the CIOMS form in conjunction with the sponsor SAE form to be sent to the MHRA by the sponsor. If warranted, an investigator alert may be issued, to inform all investigators involved in any study with the same drug (or therapy) that this serious adverse event has been reported.

The original and any subsequent follow up of Serious Adverse Event Forms and CIOMS forms (where applicable), together with the fax confirmation sheet must be kept with the TMF at the study site.

9.4 Responsibilities

- **Principal Investigator (PI)**

  Checking for AEs and ARs when participants attend for treatment / follow-up.

For peer review only - http://bmjopen.bmj.com/site/about/guidelines.xhtml

1. Using medical judgement in assigning seriousness, causality and expectedness [in Phase III and late Phase II CTIMPs] using the Reference Safety Information approved for the trial.
2. Using medical judgement in assigning seriousness and causality and providing an opinion on expectedness [in Phase I and early Phase II CTIMPs] using the Reference Safety Information approved for the trial.
3. Ensuring that all SAEs and SARs (including SUSARs) are recorded and reported to the Sponsor within 24 hours of becoming aware of the event and provide further follow-up information as soon as available. Ensuring that SAEs and SARs (including SUSARs) are chased with Sponsor if a record of receipt is not received within 2 working days of initial reporting.
4. Ensuring that AEs and ARs are recorded and reported to the Sponsor in line with the requirements of the protocol.

- **Chief Investigator (CI) / delegate or independent clinical reviewer**

1. Clinical oversight of the safety of patients participating in the trial, including an ongoing review of the risk / benefit.
2. Using medical judgement in assigning seriousness, causality and expectedness of SAEs where it has not been possible to obtain local medical assessment.
3. Using medical judgement in assigning expectedness [in Phase I and early Phase II CTIMPs].
4. Immediate review of all SUSARs.
5. Review of specific SAEs and SARs in accordance with the trial risk assessment and protocol as detailed in the Trial Monitoring Plan.
6. Assigning Medical Dictionary for Regulatory Activities (MedDRA) or Body System coding to all SAEs and SARs.
7. Preparing the clinical sections and final sign off of the Development Safety Update Report (DSUR).

- **Sponsor**

1. Central data collection and verification of AEs, ARs, SAEs, SARs and SUSARs according to the trial protocol onto a MACRO database.
2. Reporting safety information to the CI, delegate or independent clinical reviewer for the ongoing assessment of the risk / benefit according to the Trial Monitoring Plan.
3. Reporting safety information to the independent oversight committees identified for the trial (Data Monitoring Committee (DMC) and / or Trial Steering Committee (TSC)) according to the Trial Monitoring Plan.
4. Expedited reporting of SUSARs to the Competent Authority (MHRA in UK) and REC within required timelines.
5. Notifying Investigators of SUSARs that occur within the trial.
6. The un-blinding of a participant for the purpose of expedited SUSAR reporting [For double blind trials only].
7. Checking for (annually) and notifying PIs of updates to the Reference Safety Information for the trial.
8. Preparing standard tables and other relevant information for the DSUR in collaboration with the CI and ensuring timely submission to the MHRA and REC.

- **Trial Steering Committee (TSC)**

For peer review only - http://bmjopen.bmj.com/site/about/guidelines.xhtml

Health Research Authority

Anakinra vs. Steroids for the Treatment of Gout Attacks in Renal Patients (ASGARD): A Feasibility Study

EudraCT no. 2015-001787-19

In accordance with the Trial Terms of Reference for the TSC, periodically reviewing safety data and liaising with the sponsor regarding safety issues. No DMC will be formed for this study as it is a small feasibility study, but it will be considered for the subsequent larger study.

9.5 Notification of deaths

Only deaths that are assessed to be caused by the IMP will be reported to the sponsor. This report will be immediate. All deaths, including deaths deemed unrelated to the IMP, if they occur earlier than expected will be reported to the sponsor. This report will be immediate.

9.6 Pregnancy reporting

- All pregnancies within the trial (either the trial participant or the participant's partner) should be reported to the Chief Investigator and the Sponsor using the relevant Pregnancy Reporting Form within 24 hours of notification
- Pregnancy is not considered an AE unless a negative or consequential outcome is recorded for the mother or child/foetus. If the outcome meets the serious criteria, this would be considered an SAE.
- Immediate reporting to the sponsor is required (within one working day of the PI/CI becoming aware of the event) using an ARCTU Pregnancy template form.
- If a subject or his/her partner becomes pregnant whilst taking part in the trial or during a stage where the foetus could have been exposed to an IMP. The CI/PI has the responsibility to ensure that the pregnancy form is completed and sent to the sponsor within the agreed timelines. The patient can continue on the study.

- The Investigator must ensure that the subject and the subject's healthcare professional are aware that follow-up information is required on the outcome of the pregnancy. If the subject leaves the area, their new healthcare professional should also be informed.
- The PI/CI also must follow up the pregnancy until delivery as well as monitoring the development of the new-born for the appropriate time after birth. Any events that occur during this time that could be considered to be a SAE must be reported to the sponsor in line with safety reporting, utilising the sponsor SAE reporting form.

9.7 Overdose

- No dose limiting toxicity has been observed during clinical trials. Patients have received my higher doses in the setting of sepsis and there was no difference in the adverse event profile.
- Dosing will be monitored as part of trial monitoring and any extra doses will be documented in the eCRF. Patients will still continue the regime as prescribed and outcome will be included in the final analysis.
- If an SAE is felt to be associated with the overdose will be fully described in the AECRF/SAE report form.

9.8 Reporting urgent safety measures

The CI may take urgent safety measures to ensure the safety and protection of the clinical trial participants from any immediate hazard to their health and safety, in accordance with Regulation 30. The measures should be taken immediately. In this instance, the approval of the Licensing Authority

For peer review only - http://bmjopen.bmj.com/site/about/guidelines.xhtml

Approval prior to implementing these safety measures is not required. However, it is the responsibility of the CI to inform the sponsor, Main Research Ethics Committee (via telephone) and the MHRA (via telephone for discussion with the medical assessor at the clinical trials unit) of this event immediately.

The CI has an obligation to inform both the MHRA and Main Ethics Committee in writing within 3 days, in the form of a substantial amendment. The sponsor must be sent a copy of the correspondence with regards to this matter.

9.9 The type and duration of the follow-up of subjects after adverse events.

All adverse events will need complete follow-up and reporting back to the ARCTU/CI/Sponsor. This will occur until the investigator feels the event or reaction has fully resolved.

8 weeks after consent and last follow-up, the Investigator should instruct each subject to report any subsequent event(s) that the subject, or the subject's general practitioner, believes might reasonably be related to participation in this study. The Investigator should notify the study sponsor of any death or adverse event occurring at any time after a subject has discontinued or terminated study participation that may be reasonably related to this study. The investigator should update the AECRF form with outcomes of all reported events.

Any SUSAR related to the IMP will need to be reported to the Sponsor irrespective of how long after IMP administration the reaction has occurred.

9.10 Development safety update reports

The Annual Safety Reports (ASR) will be sent by the CI to the sponsor, the MREC and MHRA (the date of the anniversary is the date on the "notice of acceptance letter" from the MHRA) using the ASR form. The CI will carry out a risk benefit analysis of the IMPs encompassing all events having arisen on the trial.

The CI/ARCTU will send the Annual Progress Report to the main REC using the REC template (the anniversary date is the date on the MREC "favourable opinion" letter from the MREC) and to the sponsor.

For peer review only - http://bmjopen.bmj.com/site/about/guidelines.xhtml

Health Research Authority

Anakinra vs. Steroids for the Treatment of Gout Attacks in Renal Patients (ASGARD): A Feasibility Study

EudraCT no. 2015-001787-19

10 STATISTICS AND DATA ANALYSIS

10.1 Sample size calculation

This is a feasibility study and is not statistically powered to detect a difference. We will aim to recruit at least 16 patients in each arm of the study. The sample size of 32 was obtained from a recommendation based on the feasibility of running a parallel group design. The study will aim to enrol for 15 months. Our hospital had an average of 47 patients per year over the last four years with approximately 50% of patients having chronic kidney disease (i.e. 23 patients per year).

10.2 Planned recruitment rate

Enrolment is for 15 months from 6 centres, we will aim for one patient per month from larger centres and 1 every two months from smaller centres. If we assume a recruitment rate of xxx, we should meet out accrual rates. We will only recruit patients in working hours.

10.3 Statistical analysis plan

As this is a feasibility study the sample size will be too small for estimates to have adequate precision, and some methods will not be technically possible for some patterns of missing values. However, the best use will be made of the data that arises. The primary outcome measures are the time to 50% reduction and complete resolution of pain according to the visual analogue scale score and this will be used to compare the two groups in a time-to-event ("survival") analysis. Other time-to-event outcome measures will be analysed in a similar way. Outcome measures that are continuous, or can be taken to have an underlying continuity, such as physician assessment of joint tenderness and swelling, will be used to compare the two groups in a repeated measures analysis of variance with permutation tests. Two-sample comparisons of means will be by the two-sample permutation test. The association between the intervention and categorical variables will be tested using Fisher's exact test.

For peer review only - http://bmjopen.bmj.com/site/about/guidelines.xhtml

NHS
Health Research Authority

Anakinra vs. Steroids for the Treatment of Gout Attacks in Renal Patients (ASGARD): A Feasibility Study

EudraCT no. 2015-001787-19

10.3.1 Summary of baseline data and flow of patients

CONSORT 2010 Flow Diagram for reporting with transparency

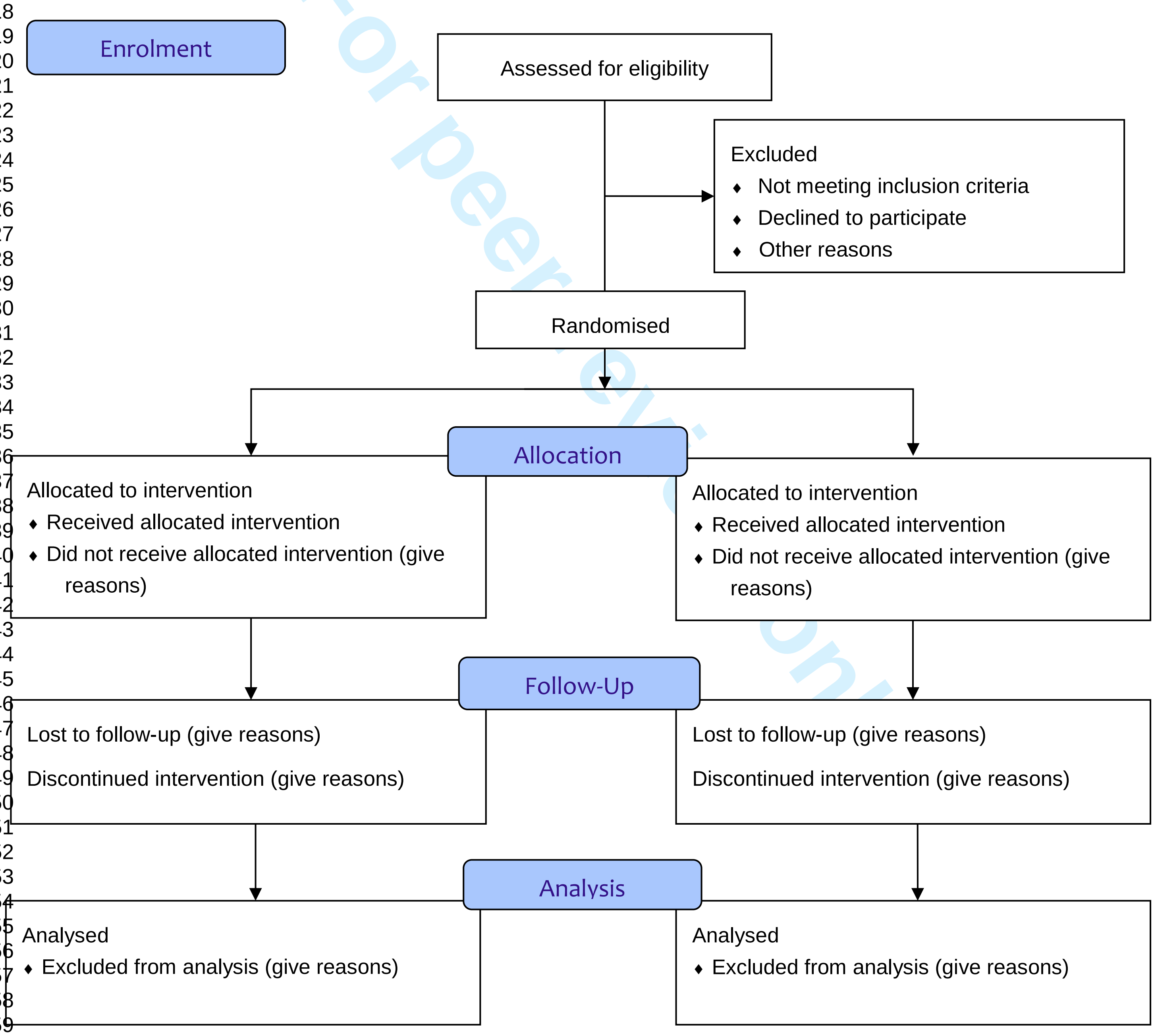

For peer review only - http://bmjopen.bmj.com/site/about/guidelines.xhtml

NHS
Health Research Authority

Anakinra vs. Steroids for the Treatment of Gout Attacks in Renal Patients (ASGARD): A Feasibility Study

EudraCT no. 2015-001787-19

For peer review only

For peer review only - http://bmjopen.bmj.com/site/about/guidelines.xhtml

10.3.2 Primary outcome analysis

- Study feasibility

  Study process will be analysed. Recruitment and retention rates, and willingness of patients to be randomised will be calculated. Proportion of patients who did not meet eligibility criteria will be looked at. Adherence and compliance rates, and qualitative feedback will be examined. Economic data on healthcare resource use and health related quality of life will be collected to inform the design of the subsequent larger study.

- Safety outcome measures with reporting as per CTIMP protocol (MHRA guidelines).

10.3.3 Secondary outcome analysis

- Proposed primary outcome measures of efficacy:
  - Time to 50% reduction and complete resolution of pain in self-assessed pain intensity in the joint most affected at baseline measure on the VAS (0-100mm) and 4-point Likert scale from baseline to 7 days post randomisation (Day 1 to Day 7)
- Patient reported outcome measure (Day 2 to Day 7)
- Physician assessment of joint tenderness and swelling (Day 1, Day 2, and Day 7)
- Time to take rescue medication
- Measurement in renal function by U+E, eGFR, CRP and urine uric acid
- Healthcare resource use.
- Patient assessment of activity limitation and quality of life: LEFS (Day 1, Day2, Day 7); EQ-5D-5L (Day 1, Day 2, Day 7 and 8 weeks); SF-36 and HAQ-DI (Day 1, Day 7 and 8 weeks).
- Changes in blood and urine inflammatory markers (Day 1, Day 2 Day 7 and 8 week).

10.4 Subgroup analyses

Not applicable

10.5 Adjusted analysis

Not applicable

10.6 Interim analysis and criteria for the premature termination of the trial

There will be no formal interim analysis. The steering committee will meet at pre-specified intervals. Patients will remain blinded, even if any adaptations are made. Analysis will be undertaken at end of study. The integrity of the trial will be protected by regular site visits and conferences (telephone, online and face-to-face meetings) with PIs.

The authority to stop or modify the trial lies with the Chief Investigator, trial steering committee, or sponsor. Although there is no formal data monitoring committee due to the small size of the study, some of the responsibilities of a DMA will be undertaken by the trial management committee as there is a pharmacovigilance aspect to this study.

10.7 Subject population

Data from all randomised population will be subjected to the study analysis, regardless of whether they received study drug and protocol adherence. This will be an intention to treat analysis.

For peer review only - http://bmjopen.bmj.com/site/about/guidelines.xhtml

10.8 Procedure(s) to account for missing or spurious data

Processes in the protocol to minimise missing or spurious data includes reviews of patients at various time points, including at end of study.

The primary analysis is a time-to-event ("survival") analysis which accommodates missing data in its treatment of loss to follow-up. The repeated measures analysis of variance will be carried out as a linear mixed model using an algorithm that does not require balance and can accommodate missing data. For example this can be using the package lme4 for the computer program R. In this way missing values will not result in patients being dropped from an analysis. The serious issue of a possible differential dropout from the two groups will be examined carefully.

10.9 Other statistical considerations.

Not applicable

10.11 Economic evaluation

As this is a feasibility study, it is not designed or powered to show differences in effectiveness between groups and hence is unlikely to show differences in cost-effectiveness. For this reason a formal economic evaluation looking at the incremental cost-effectiveness of Anakinra compared to the comparator is not planned. Instead we will collect relevant data on resource use (costs) and health-related quality of life to inform subsequent large scale study. We will record all resources required to provide the drug treatment in both groups. This will include costs of drugs and administration and any required tests and contacts with health care practitioners. We will also record health care contacts by means of a patient administered questionnaire at the 8-week follow-up. Where possible we will also record the use of health care from the patient's notes and other sources. This will be used to access the validity of patient completed data and will inform future research design. Data collected will identify key drivers of cost in this group of individuals and will allow us to access the likely cost of providing the two drug treatments.

The performance of the EQ-5D-5L will be assessed in this group of patients. We will also calculate the SF-6D from the SF-36 questionnaire and compare the performance of this measure with that of the EQ-5D-5L.

For peer review only

For peer review only - http://bmjopen.bmj.com/site/about/guidelines.xhtml

11 DATA HANDLING

11.1 Data collection tools and source document identification

Source Data

The participants' clinical notes and anywhere that data is first recorded will act as source data. This will be used to obtain information for past medical history, subsequent medical conditions, hospital admissions, data on resource use, diagnostic reports and blood and urine tests. This will also include entries from study questionnaires (LEFS, EQ-5D-5L, SF-36 and HAQ-DI) and patient diaries (PROMS, VAS, and Likert scale). Entry will be made into eCRF forms and be printed out and stored at site with patient notes to form part of source data.

Source Documents

Source documents consist of:

- Case histories in participants' clinical notes, including copies of original records and signed copies of consent form.
- Pharmacy documentation of dispensed treatment
- Original documents, data and records including charts, subject's diaries, study questionnaires, subject's recordings, pharmacy records, and records kept in clinical laboratories etc.

Case report forms

Data will be collected and retained in accordance with the Data Protection Act (1988). Electronic case report forms (eCRFs) will be used via an online system (MACRO) to collect data. A validated data collection tool appropriate to the study will be used. The investigator is responsible for ensuring the accuracy, completeness, legibility and timeliness of the data reported in the CRFs.

Forms can be remotely accessed through an internet portal and study data will be entered and captured there. Data will be anonymised and patients will only be identifiable by a study number. Any inconsistencies, validation errors or inaccuracies will be reported to the CI regularly. One data collection is complete and the data has been validated, a data lock will be performed and analysis will be initiated. Only data required by the protocol will be captured in the eCRF.

CRFs as Source Documents

Data can be entered directly onto the eCRF (MACRO) by site research team, and if this is done then this would then be considered a source document. If the CRF is then transmitted to the sponsor, it is necessary for the trial site to retain a copy to ensure that the principal investigator can provide access to the source documents to a monitor, auditor, or regulatory agency.

Copied CRFs as Source Documents

Photocopies or print-outs of the CRFs may be used as source documents. Copies will be made onto coloured paper so they are easy to find in the case history and will not be mistaken for CRFs. The photocopied case report forms will indicate who collected the data (sign/initial and date) as well as the subject to whom the data applies.

For peer review only - http://bmjopen.bmj.com/site/about/guidelines.xhtml

Health Research Authority

11.2 Data handling and record keeping

The Investigator and the study team will ensure that the Patient's identity is protected at every stage of their participation within the study. Each patient will be allocated a unique screening number by either the PI or a member of the study team before undergoing any screening procedures, this information will be kept on a screening log. Once the patient has completed screening procedures and is enrolled onto the study, the patient will be allocated a randomisation number determined by the randomisation system within the ARCTU. The data will be coded and depersonalised where the participant's identifying information is replaced by an unrelated sequence of characters. The patients initials (the first letter of their first name and the first letter of their last name) may be used as a means of pseudo-anonymising parameters. It will be securely maintained with the linking code in separate locations using encrypted digital files within password protected folders and storage media.

There will be limited access to the minimum number of individuals necessary for quality control, audit, and analysis. Identifiable data will be stored within the framework of the Caldicott principles. All stored data at the trial site, clinical trial unit and the main trial centre will be subject to review by a responsible individual from the clinical trial unit for monitoring and audit purposes. Completeness of data will be ensured by members of the research team contacting patients to ensure that allocated medication has been administered and that the main primary outcome data, patient reported pain score has been done..

The eCRF for data entry utilises MACRO. The eCRF will be developed, managed and maintained by ARCTU. Access to the eCRF will be restricted, with only authorised site-specific personnel able to make entries or amendments to their participants' data. It is the PI's/CI's responsibility to ensure completion and to review and approve all data captured in the eCRF. The PI or recruiting physician or research team will be responsible for all entries into the eCRF and will confirm (electronically) that the data are accurate and complete.

The investigator /institutions will keep records of all participating patients (sufficient information to link records e.g., CRFs, hospital records and samples), all original signed informed consent forms and copies of the CRF pages.

If any patient information needs to be sent to a third party (including correspondence/communication to central laboratories, the PI and the study team will adhere to patient pseudo-anonymous parameters. This includes the patient initials, date of birth, gender as well as the unique study ID/randomisation number. Any information that is to be collected by these third parties will utilise these coded details for any relevant documents as well as maintaining databases.

11.3 Data storage

Personal data and contact information / direct identifiers will be stored in the trial site file at the study site under the custodianship of the principle investigator for up to 15 years with participant consent. This will be available to the research / clinical team providing direct care for the patient. This will be stored with secure access in a locked cabinet. Contact information is required as the study requires daily contact with participants to ensure adherence to treatment and undertaking of assessments for data completeness. Patients will be asked for consent to keep records to contact them on a later date for update on the results and outcome of the study, and inform of further studies they could be involved with.

For peer review only - http://bmjopen.bmj.com/site/about/guidelines.xhtml

Health Research Authority

Anakinra vs. Steroids for the Treatment of Gout Attacks in Renal Patients (ASGARD): A Feasibility Study

EudraCT no. 2015-001787-19

Coded data will be stored in the pharmacy of the study centre / hospital to monitor treatment allocation. A key to coded data will be held by the local pharmacy. This information will be stored in a locked cabinet with access only available to a nominated pharmacy representative identified in the pharmacy protocol.

Linked anonymised data or pseudonymised data will be entered directly using a secure web-based portal by the trial site team and stored in the research database at the Anglia Ruskin Clinical Trials Unit. Data will be stored in a locked cabinet / room with secure access. This data will be collected until the end of the study after which analysis will be undertaken by Mike Parker, Anglia Ruskin University Clinical Trials Unit statistician, to produce a report of the findings. Health economic data that will be link anonymised will be transferred under custodianship of David Turner, Norwich Medical School, University of East Anglia.

Linked anonymised data including on samples obtained will also be stored in a database with secure access only to the chief investigator and stored in a locked cabinet at the main trial site, Southend University Hospital, where samples will be stored. This will be under the custodianship of the chief investigator, Gowrie Balasubramaniam. Patients will be consented explicitly for this. Consent will be explicitly sought to keep records for 15 years with a view to use this data for future, as yet unspecified, medical research into health. Illness and medical treatment.

There is a qualitative aspect to the study that was suggested by the funding body that will require for participants to be interviewed with regards to the experience of participation. 12 out of the 32 patients will be randomly selected for qualitative assessment of participation experience. Consent will be explicitly obtained for audio interviews to be recorded. Pseudonymised data will be used for recording purposes and data obtained will be stored in a locked cabinet with secure access only to Dr Jon Scales, University of Essex.

11.4 Access to Data

Direct access will be granted to authorised representatives from the Sponsor, host institution and the regulatory authorities to permit trial-related monitoring, audits and inspections.

11.5 Archiving

During the course of research, all records are the responsibility of the Chief Investigator and will be kept in secure conditions. When the research trial is complete, records will be kept for a further 15 years as required by the Research Governance Framework and Trust Policy that the time.

For trials involving ____ Trust patients, undertaken by

_____________ staff, the approved repository for long-term storage of local records is

______________________________________ which is based at ____________________________.

Archiving will be authorised by the Sponsor following submission of the end of study report. All essential documents will be archived for a minimum of 5 years after completion of trial. Destruction of essential documents will require authorisation from the Sponsor.

The trial database will be securely stored at Anglia Ruskin Clinical Trials Unit under the ownership of the Chief Investigator.

For peer review only - http://bmjopen.bmj.com/site/about/guidelines.xhtml

Health Research Authority

Anakinra vs. Steroids for the Treatment of Gout Attacks in Renal Patients (ASGARD): A Feasibility Study

EudraCT no. 2015-001787-19

For peer review only

For peer review only - http://bmjopen.bmj.com/site/about/guidelines.xhtml

12 MONITORING, AUDIT & INSPECTION

Trial monitoring will be co-ordinated by Anglia Ruskin Clinical Trials Unit.

The purpose of monitoring is to verify that:

- The rights and well-being of the participants are protected
- Reported trial data is accurate, complete and verifiable from source documents
- The conduct of the trial is in compliance with the currently approved protocol/amendment(s), GCP and the applicable regulatory requirements.
- Part of the quality control of clinical trial and ensures ongoing quality and rigour of the study.

A Trial Monitoring Plan will be developed and agreed by the Trial Management Group (TMG) and TSC based on the trial risk assessment which may include on site monitoring

After the first site initiation visit, subsequent visits will be planned by the TMG, this will be based on the on-going assessment by the TMG and reviewing any problems that may arise from the trial data set. Monitoring might be initially conducted across all sites, and subsequently conducted using a risk based approach that focuses, for example, on sites that have the highest enrolment rates, large numbers of withdrawals, or atypical (low or high) numbers of reported adverse events.

The monitoring procedures will be outlined in a standard operating procedure to ensure uniformity and is thorough.

The processes reviewed will examine participant enrolment, consent, eligibility, and allocation to trial groups; adherence to trial interventions and policies to protect participants, including reporting of harm and completeness, accuracy, and timeliness of data collection

A member of the study team from the site will co-ordinate site visits / assessments

The site study team member will ensure:

- All required reports, notifications, applications and submissions are available and that these documents are accurate, complete, timely, legible, dated and identify the trial.
- All documents and trial supplies needed to conduct the trial properly, and to comply with the applicable regulatory requirements, are available.
- The most current up to date reference documents are available, and that all documents are available for review.
- All personnel part of the study team have adequate training, resources and facilities, including laboratories, equipment and staff, to safely and properly conduct the trial and the these remain adequate throughout the study period.
- Trial functions are not delegated to unauthorised individuals.
- Source documents and other trial records are accurate, complete and up-to-date, check the accuracy and completeness of the eCRF entries.
- Any data required by the protocol is reported accurately in the eCRF and is consistent with the source documents
- Any treatment modification, adverse events, concomitant medications and inter-current illness is reported in accordance with the protocol in the eCRFs.
- Any visits that the participant fails to make, tests not conducted and examinations not performed are reported clearly in the eCRFs.

For peer review only - http://bmjopen.bmj.com/site/about/guidelines.xhtml

- All withdrawals and dropouts of enrolled subjects from the project are reported and explained on the eCRF
- the monitoring team are informed of any eCRF entry error, omission, or illegibility and ensure that appropriate corrections, additions or delegations are made, dated and explained (if necessary) and initialled by the Principle Investigator or an authorised individual. The monitor will not make such changes.
- Verification and collection of participant data should be performed according to data protection laws and requirements.
- All SAEs are appropriately reported within the time periods required by GCP, the protocol, the REC, clinical trial units and the applicable regulatory requirements.

With regards to the IMPs

- Ensure storage times and conditions are acceptable and that supplies are sufficient
- IMP is only supplied to subjects who are eligible, at the protocol specified dose
- Participants are provided with necessary instruction on properly using, handling, storing and returning IMPS
- The receipt, use and return of any IMP9s) at the trial sites are controlled and documented adequately.
- Disposal If unused IMP(s) complies with applicable regulatory requirement(s) and is in accordance with the sponsors of the SOP.
- Ensure that any code breaks are properly handled and documented according to the protocol and/or relevant code break SOP.
- Ensure that communication is in place in the event of any suspicion of scientific misconduct, fraud, or breach of GCP.

For peer review only - http://bmjopen.bmj.com/site/about/guidelines.xhtml

13 ETHICAL AND REGULATORY CONSIDERATIONS

13.1 Research Ethics Committee (REC) review & reports

Approval will be sought from a REC for the trial protocol, informed consent forms and other relevant documents e.g. advertisements and GP information letter. Substantial amendments that require review by REC will not be implemented until the REC grants a favourable opinion for the study. All correspondence with the REC will be retained in the Trial Master File/Investigator Site File. An annual progress report (APR) will be submitted to the REC within 30 days of the anniversary date on which the favourable opinion was given, and annually until the trial is declared ended.

It will be the Chief Investigator's responsibility to produce the annual reports as required. The Chief Investigator will notify the REC of the end of the study if the study is ended prematurely, the Chief Investigator will notify the REC, including the reasons for the premature termination.

The Chief Investigator will submit a final report with the results, including any publications/abstracts, to the REC within one year after the end of the study.

13.2 Peer review

This trial protocol was reviewed by the funder (NIHR) and internal Trust departments. It is also reviewed by the Trust Research and Development Committee.

13.3 Public and Patient Involvement

There has been public and patient involvement since the conception of this study. They have been involved in the design, and management of the research. There are members of the steering committee that are members of the patient and public group. They will help with the dissemination of the study.

13.4 Regulatory Compliance

The trial will not commence until a Clinical Trial Authorisation (CTA) is obtained from the MHRA and the protocol and trial conduct will comply with the Medicines for Human Use (Clinical Trials) Regulations 2004 and any relevant amendments.

13.5 Protocol compliance

Prospective, planned deviations or waivers to the protocol are not allowed under the UK regulations on Clinical Trials and must not be used e.g. it is not acceptable to enrol a subject if they do not meet the eligibility criteria or restrictions specified in the trial protocol. Accidental protocol deviations can happen at any time and must be adequately documented on the relevant forms and reported to the Chief Investigator and Sponsor immediately. Deviations from the protocol which are found to frequently recur are not acceptable, will require immediate action and could potentially be classified as a serious breach.

13.6 Notification of Serious Breaches to GCP and/or the protocol

A "serious breach" is a breach which is likely to effect to a significant degree –

(a) the safety or physical or mental integrity of the subjects of the trial; or
(b) the scientific value of the trial

For peer review only - http://bmjopen.bmj.com/site/about/guidelines.xhtml

Health Research Authority

Anakinra vs. Steroids for the Treatment of Gout Attacks in Renal Patients (ASGARD): A Feasibility Study

EudraCT no. 2015-001787-19

The sponsor will be notified immediately of any case where the above definition applies during the trial conduct phase. The sponsor of a clinical trial will notify the licensing authority in writing of any serious breach of:

(a) the conditions and principles of GCP in connection with that trial; or
(b) the protocol relating to that trial, as amended from time to time, within 7 days of becoming aware of that breach

13.7 Data protection and patient confidentiality

All investigators and trial site staff must comply with the requirements of the Data Protection Act 1998 with regards to the collection, storage, processing and disclosure of personal information and will uphold the Act's core principles.

The Investigator has a responsibility to ensure that patient anonymity is protected and maintained. They must also ensure that their identities are protected from any unauthorised parties. Information with regards to study patients will be kept confidential and managed in accordance with the Data Protection Act, NHS Caldicott Guardian, The Research Governance Framework for Health and Social Care and Research Ethics Committee Approval.

Data will be stored for five years before being destroyed. The Chief Investigator in the data custodian.

13.8 Financial and other competing interests for the chief investigator, PIs at each site and committee members for the overall trial management

The drug and equivalent placebo is provided by the distributor, Swedish Orphan Biovitrium (SOBI). There is a payment of £10,000 to help with additional study costs.

13.9 Indemnity

This is UK NHS-sponsored study. For NHS sponsored research HSG (96)48 reference no 2. Refers. If there is negligent harm during the clinical trial when the NHS body owes a duty of care to the person harmed, NHS indemnity covers UK NHS staff, medical academic staff with honorary contracts conducting the trial. NHS indemnity does not offer no-fault compensation and is unable to agree in advance to pay compensation for non-negligent harm. Ex-gratia payments may be considered in the case of claim. Principle investigators and their medical staff should seek indemnity against non-negligent harm for this study from their NHS trust.

13.10 Amendments

Changes made to the research after review body approval are amendments. These may be substantial or non-substantial. Amendments will be tracked in the protocol appendix and the version of the protocol will be updated.

13.10.1 Non-substantial Amendments

The sponsor may make a non-substantial amendment at any time during a trial under the Medicines for Human Use (Clinical Trials) Regulations 2004. Non-substantial amendments will be made by using the Notification of Non-Substantial/Minor Amendment(s) for NHS Studies form from the HRA website. The form will be filled by the CI ad authorised by the sponsor.

Examples of Non-substantial Amendments

For peer review only - http://bmjopen.bmj.com/site/about/guidelines.xhtml

Health Research Authority

- minor changes to the protocol or other study documentation, e.g. correcting errors, updating contact points, minor clarifications;
- updates of the investigator's brochure (unless there is a change to the risk/benefit assessment for the trial);
- changes to the chief investigator's research team changes to the research team at particular trial sites (other than appointment of a new principal investigator in a CTIMP);
- changes in funding arrangements; changes in the documentation used by the research team for recording study data;
- changes in the logistical arrangements for storing or transporting samples; inclusion of new sites and investigators in studies other than CTIMPs;
- extension of the study beyond the period specified in the application form

13.10.2 Substantial Amendments

If the sponsor wishes to make a substantial amendment to the CTA or the documents that supported the original application for the CTA, the sponsor must submit a valid notice of amendment to the licencing authority (MHRA) for consideration. If the sponsor wishes to make a substantial amendment to the REC application or the supporting documents, the sponsor must submit a valid notice of amendment to the REC for consideration. The MHRA and/or the REC will provide a response regarding the amendment within 35 days of receipt of the notice. It is the sponsor's responsibility to decide whether an amendment is substantial or non-substantial for the purposes of submission to the MHRA and/or REC.

If applicable, other specialist review bodies (e.g. CAG) need to be notified about substantial amendments in case the amendment affects their opinion of the study.

Amendments also need to be notified to NHS R&D departments of participating sites to assess whether the amendment affects the NHS permission for that site. Note that some amendments that may be considered to be non-substantial for the purposes of REC and/or MHRA may still need to be notified to NHS R&D (e.g. a change to the funding arrangements). For studies with English sites processed in NIHR CSP the amendment should be submitted in IRAS to the lead CRN, which will determine whether the amendment requires notification to English sites or may be implemented immediately (subject to REC/MHRA approval were necessary).

Examples of Substantial amendments

- changes to the design or methodology of the study, or to background information affecting its scientific value;
- changes to the procedures undertaken by participants;
- any change relating to the safety or physical or mental integrity of participants, or to the risk/benefit assessment for the study;
- significant changes to study documentation such as participant information sheets, consent forms, questionnaires, letters of invitation, letters to GPs or other clinicians, information sheets for relatives or carers;
- a change of sponsor(s) or sponsor's legal representative; appointment of a new chief investigator a change to the insurance or indemnity arrangements for the study;

For peer review only - http://bmjopen.bmj.com/site/about/guidelines.xhtml

Health Research Authority

- inclusion of a new trial site (not listed in the original application) in a CTIMP; appointment of a new principal investigator at a trial site in a CTIMP;
- temporary halt of a study to protect participants from harm, and the planned restart of a study following a temporary halt; a change to the definition of the end of the study;
- any other significant change to the protocol or the terms of the REC application

13.11 Post trial care

The treatments are for an acute flares of gout and participants will not need to be on the treatments for a long period of time. It is beyond the scope of this trial to look at the use of long-term gout prophylaxis. This is an area where evidence in people with kidney disease is lacking and we are collecting data at the 8-week point (last time point) to see if the various healthcare providers treating participants use prophylactic treatments on participants.

Use of Anakinra is not licensed for acute attacks of gout but has been used worldwide with evidence increasing building up. Continued access to this intervention need not be considered and it is available for off-label use by healthcare professionals. Intramuscular methylprednisolone is readily available and used widely.

13.12 Access to the final trial dataset

The final dataset will be available to the statistician for data analysis. Components of health economic will be made available to the health economist for economic analysis. The chief investigator and members of the steering group will have access to the full dataset. This will ensure that results are not disclosed prior to publication. Site investigators will not have access to full dataset.

For peer review only

For peer review only - http://bmjopen.bmj.com/site/about/guidelines.xhtml

14 DISSEMINIATION POLICY

14.1 Dissemination policy

The trial report will be used for publications and presentation at scientific meetings. Investigators have the right to publish orally or in writing the results of the study. On completion of the trial, the data will be analysed and tabulated and a Final Study Report prepared. Summaries of results will be made available to investigators for dissemination and feedback to participants.

Patient groups will also be sent summaries as their involvement with the subsequent larger study will be pertinent. Local primary care networks will also be informed.

The data will be owned by the trial steering committee, primarily by the chief investigator. Funding bodies will be declared in publications. The full report will be made available directly from the chief investigator or from published material. Supplemental material linked with publication will consist of the trial protocol.

14.2 Authorship eligibility guidelines and any intended use of professional writers

The author of the final report will be the chief investigator. Named authors of manuscripts for publications will be in accordance with the International Committee of Medical Journal Editors has defined authorship criteria for manuscripts submitted for publication.

For peer review only

For peer review only - http://bmjopen.bmj.com/site/about/guidelines.xhtml

15 REFERENCES

1) Fraser SD, Roderick PJ, Aitken G et al. Chronic kidney disease, albuminuria and socioeconomic status in the Health Surveys for England 2009 and 2010. J Public Health (Oxf). 2013 Nov 25.
2) Juraschek SP, Kovell LC, Miller ER 3rd, Gelber AC. Association of kidney disease with prevalent gout in the United States in 1988-1994 and 2007-2010. Semin Arthritis Rheum. 2013 Jun;42(6):551-61.
3) Annemans L, Spaepen E, Gaskin M, Bonnemaire M, Malier V, Gilbert T, Nuki G. Gout in the UK and Germany: prevalence, comorbidities and management in general practice 2000-2005. Ann Rheum Dis. 2008 Jul;67(7):960-6.
4) Keenan RT, O'Brien WR, Lee KH et al. Prevalence of contraindications and prescription of pharmacologic therapies for gout. Am J Med. 2011 Feb;124(2):155-63.
5) Curiel RV, Guzman NJ. Challenges associated with the management of gouty arthritis in patients with chronic kidney disease: a systematic review. Semin Arthritis Rheum. 2012 Oct;42(2):166-78.
6) Rothenbacher D, Primatesta P, Ferreira A, Cea-Soriano L, Rodríguez LA. Frequency and risk factors of gout flares in a large population-based cohort of incident gout. Rheumatology (Oxford). 2011 May;50(5):973-81.
7) Jordan KM, Cameron JS, Snaith M et al. British Society for Rheumatology and British Health Professionals in Rheumatology guideline for the management of gout. British Society for Rheumatology and British Health Professionals in Rheumatology Standards, Guidelines and Audit Working Group (SGAWG). Rheumatology (Oxford). 2007 Aug;46(8):1372-4.
8) Zhang W, Doherty M, Pascual E et al. EULAR evidence based recommendations for gout. Part I: Diagnosis. Report of a task force of the Standing Committee for International Clinical Studies Including Therapeutics(ESCISIT). EULAR Standing Committee for International Clinical Studies Including Therapeutics. Ann Rheum Dis. 2006 Oct;65(10):1301-11.
9) Khanna D, Khanna PP, Fitzgerald JD et al 2012. American College of Rheumatology guidelines for management of gout. Part 2: therapy and anti-inflammatory prophylaxis of acute gouty arthritis. American College of Rheumatology. Arthritis Care Res (Hoboken). 2012 Oct;64(10):1447-61.
10) Siegel LB, Alloway JA, Nashel DJ. Comparison of adrenocorticotropic hormone and triamcinolone acetonide in the treatment of acute gouty arthritis. J Rheumatol. 1994 Jul;21(7):1325-7.
11) Terkeltaub RA, Furst DE, Bennett K, Kook KA, Crockett RS, Davis MW. High versus low dosing of oral colchicine for early acute gout flare: Twenty four-hour outcome of the first multicenter, randomized, double-blind, placebocontrolled, parallel-group, dose-comparison colchicine study. Arthritis Rheum. 2010 Apr;62(4):1060-8.
12) Janssens HJ, Janssen M, van de Lisdonk EH, van Riel PL, van Weel C. Use of oral prednisolone or naproxen for the treatment of gout arthritis: a double-blind, randomised equivalence trial. Lancet. 2008 May 31;371(9627):1854-60.
13) Pyne D, Ioannou Y, Mootoo R, Bhanji A. Intra-articular steroids in knee osteoarthritis: a comparative study of triamcinolone hexacetonide and methylprednisolone acetate. Clin Rheumatol. 2004 Apr;23(2):116-20. Epub 2004 Feb 24.
14) Corkill MM, Kirkham BW, Chikanza IC, Gibson T, Panayi GS. Intramuscular depot methylprednisolone induction of chrysotherapy in rheumatoid arthritis: a 24-week randomized controlled trial. Br J Rheumatol. 1990 Aug;29(4):274-9.
15) Choy EH, Kingsley GH, Khoshaba B, Pipitone N, Scott DL. A two year randomised controlled trial of intramuscular depot steroids in patients with established rheumatoid arthritis who have shown an incomplete response to disease modifying antirheumatic drugs. Intramuscular Methylprednisolone Study Group. Ann Rheum Dis. 2005 Sep;64(9):1288-93.
16) Dinarello CA. Interleukin-1 in the pathogenesis and treatment of inflammatory diseases. Blood. 2011 Apr 7;117(14):3720-32.

For peer review only - http://bmjopen.bmj.com/site/about/guidelines.xhtml

Health Research Authority

Anakinra vs. Steroids for the Treatment of Gout Attacks in Renal Patients (ASGARD): A Feasibility Study

EudraCT no. 2015-001787-19

17) So A, De Smedt T, Revaz S, Tschopp J. A pilot study of IL-1 inhibition by anakinra in acute gout. Arthritis Res Ther. 2007;9(2):R28.
18) Chen K, Fields T, Mancuso CA, Bass AR, Vasanth L. Anakinra's efficacy is variable in refractory gout: report of ten cases. Semin Arthritis Rheum. 2010 Dec;40(3):210-4.
19) Ghosh P, Cho M, Rawat G et al. Treatment of acute gouty arthritis in complex hospitalized patients with anakinra.Arthritis Care Res (Hoboken). 2013 Aug;65(8):1381-4.
20) Ottaviani S, Moltó A, Ea HK et al. Efficacy of anakinra in gouty arthritis: a retrospective study of 40 cases. Arthritis Res Ther. 2013;15(5):R123.
21) Thueringer JT, Doll NK, Gertner E. Anakinra for the treatment of acute severe gout in critically ill patients. Semin Arthritis Rheum. 2015 Aug;45(1):81-5
22) Balasubramaniam G, Almond M, Dasgupta B. Improved renal function in diabetic patients with acute gout treated with anakinra. Kidney Int. 2015 Jul;88(1):195-6.
23) Direz G, Noël N, Guyot C, Toupance O, Salmon JH, Eschard JP. Efficacy but side effects of anakinra therapy for chronic refractory gout in a renal transplant recipient with preterminal chronic renal failure. Joint Bone Spine. 2012 Dec;79(6):631.
24) Nuki G, Bresnihan B, Bear MB et al. Long-term safety and maintenance of clinical improvement following treatment with anakinra (recombinant human interleukin-1 receptor antagonist) in patients with rheumatoid arthritis: extension phase of a randomized, double-blind, placebo controlled trial. Arthritis Rheum. 2002 Nov;46(11):2838-46.
25) Fleischmann RM, Schechtman J, Bennett R. Anakinra, a recombinant human interleukin-1 receptor antagonist (r-metHuIL-1ra), in patients with rheumatoid arthritis: A large, international, multicenter, placebo-controlled trial. Arthritis Rheum. 2003 Apr;48(4):927-34.
26) Schiff MH, DiVittorio G, Tesser J. The safety of anakinra in high-risk patients with active rheumatoid arthritis: six-month observations of patients with comorbid conditions. Arthritis Rheum. 2004 Jun;50(6):1752-60.
27) Fleischmann RM, Tesser J, Schiff MH. Safety of extended treatment with anakinra in patients with rheumatoid arthritis. J Rheumatol. 2006 Feb;33(2):234-43.
28) Larsen CM, Faulenbach M, Vaag A, Vølund A, Ehses JA, Seifert B, Mandrup-Poulsen T, Donath MY. Interleukin-1-receptor antagonist in type 2 diabetes mellitus. N Engl J Med. 2007 Apr 12;356(15):1517-26.
29) Abbate A, Kontos MC, Abouzaki NA et al. Comparative safety of interleukin-1 blockade with anakinra in patients with ST-segment elevation acute myocardial infarction (from the VCU-ART and VCU-ART2 pilot studies). Am J Cardiol. 2015 Feb 1;115(3):288-92.
30) Schlesinger N, Mysler E, Lin HY et al. Canakinumab reduces the risk of acute gouty arthritis flares during initiation of allopurinol treatment: results of a double-blind, randomised study. Ann Rheum Dis. 2011 Jul;70(7):1264-71.
31) Di Giovine FS, Malawista SE, Nuki G, Duff GW. Interleukin 1 (IL 1) as a mediator of crystal arthritis. Stimulation of T cell and synovial fibroblast mitogenesis by urate crystal-induced IL 1. J Immunol. 1987 May 15;138(10):3213-8.
32) Arthritis Advisory Committee Meeting 2011: FDA briefing committee report. http://www.fda.gov/downloads/AdvisoryCommittees/CommitteesMeetingMaterials/Drugs/Arthritis DrugsAdvisoryCommittee/UCM259596.pdf
33) Mitha E, Schumacher HR, Fouche L et al. Rilonacept for gout flare prevention during initiation of uric acid-lowering therapy: results from the PRESURGE-2 international, phase 3, randomized, placebo-controlled trial. Rheumatology (Oxford). 2013 Jul;52(7):1285-92.
34) Schumacher HR Jr, Evans RR, Saag KG et al. Rilonacept (interleukin-1 trap) for prevention of gout flares during initiation of uric acid-lowering therapy: results from a phase III randomized, double

For peer review only - http://bmjopen.bmj.com/site/about/guidelines.xhtml

Health Research Authority

Anakinra vs. Steroids for the Treatment of Gout Attacks in Renal Patients (ASGARD): A Feasibility Study

EudraCT no. 2015-001787-19

blind, placebo-controlled, confirmatory efficacy study. Arthritis Care Res (Hoboken). 2012 Oct;64(10):1462-70.
35) Terkeltaub RA, Schumacher HR, Carter JD at al. Rilonacept in the treatment of acute gouty arthritis: a randomized, controlled clinical trial using indomethacin as the active comparator. Arthritis Res Ther. 2013 Feb 1;15(1):R25.
36) So A, De Meulemeester M, Pikhlak A et al. Canakinumab for the treatment of acute flares in difficult-to-treat gouty arthritis: Results of a multicenter, phase II, dose-ranging study. Arthritis Rheum. 2010 Oct;62(10):3064-76.
37) Schlesinger N, De Meulemeester M, Pikhlak A et al. Canakinumab relieves symptoms of acute flares and improves health-related quality of life in patients with difficult-to-treat Gouty Arthritis by suppressing inflammation: results of a randomized, dose-ranging study. Arthritis Res Ther. 2011 Mar 25;13(2):R53.
38) Schlesinger N, Alten RE, Bardin T et al. Canakinumab for acute gouty arthritis in patients with limited treatment options: results from two randomised, multicentre, active-controlled, double blind trials and their initial extensions. Ann Rheum Dis. 2012 Nov;71(11):1839-48.
39) Hung AM, Ellis CD, Shintani A et al. IL-1β receptor antagonist reduces inflammation in hemodialysis patients.J Am Soc Nephrol. 2011 Mar;22(3):437-42.
40) Chakraborty A, Van LM, Skerjanec A. Pharmacokinetic and pharmacodynamic properties of canakinumab in patients with gouty arthritis. 2013 Dec;53(12):1240-51.
41) Bresnihan B, Alvaro-Gracia JM, Cobby M. Treatment of rheumatoid arthritis with recombinant human interleukin-1 receptor antagonist. Arthritis Rheum. 1998 Dec;41(12):2196-204.
42) Sicras-Mainar A, Navarro-Artieda R, Ibá˜nez-Noll J. Resource Use and Economic Impact of Patients With Gout: A Multicenter, Population-Wide Study. Reumatol Clin. 2013;9(2):94–10
43) Chandratre P, Roddy E, Clarson L, Richardson J, Hider SL, Mallen CD. Health-related quality of life in gout: a systematic review. Rheumatology (Oxford). 2013 Nov;52(11):2031-40. doi:0.1093/rheumatology/ket265.
44) Scire CA, Manara M, Cimmino MA et al. Gout impacts on function and health-related quality of life beyond associated risk factors and medical conditions: results from the KING observational study of the Italian Society for Rheumatology (SIR). Arthritis Res Ther. 2013 Aug 23;15(5):R101
45) Wu E, Patel P, Yu A et al. Disease Related and All-Cause Health Care Costs of Elderly Patients With Gout. Jour Manag Care Pharm 2008;14(2):164-75.
46) Khanna PP, Nuki G, Bardin T et al. Tophi and frequent gout flares are associated with impairments to quality of life, productivity, and increased healthcare resource use: Results from a cross-sectional survey. Health Qual Life Outcomes. 2012 Sep 22;10:117. doi: 10.1186/1477-7525-10-117.
47) Singh JA, Taylor WJ, Simon LS et al. Patient-reported outcomes in chronic gout: a report from OMERACT 10. J Rheumatol. 2011 Jul;38(7):1452-7.
48) Singh JA, Taylor WJ, Dalbeth N et al. OMERACT Endorsement of Measures of Outcome for Studies of Acute Gout. J Rheumatol. 2013 Dec 15.

For peer review only - http://bmjopen.bmj.com/site/about/guidelines.xhtml

16. APPENDICIES

16.1 Appendix 1-Risk

Risks associated with trial interventions

☐ LOW ≡ Comparable to the risk of standard medical care

× MODERATE ≡ Somewhat higher than the risk of standard medical care

☐ HIGH ≡ Markedly higher than the risk of standard medical care

Justification: Briefly justify the risk category selected and your conclusions below (where the table is completed in detail the detail need not be repeated, however a summary should be given):

There have been reports of increased risk of infections and low white cell counts in patients treated with similar agents. This may not apply with our CTIMP, Anakinra, which has good long-term safety data from use in other conditions like arthritis and septicaemia. It is a biologic and this puts it at higher risk than standard medical care.

There are also reports of injection site reactions, which would not occur with other treatments as Anakinra requires daily injections for the course of treatment.

What are the key risks related to therapeutic interventions you plan to monitor in this trial?		How will these risks be minimised?		
IMP/Intervention	Body system/Hazard	Activity	Frequency	Comments
Anakinra	Infection	Monitoring of patients and avoid recruiting patients with active infection	Throughout project, up to 8 weeks	Pharmacovigilance is an aspect of project
Anakinra	Low white cell count	Blood tests. Avoid recruiting patients with low white cell counts	Day 1, 2, 7, 8 wk	Can be reversed by stopping treatment due to its short duration of action.
Anakinra	Injection site reactions	Monitoring of injection site	Day 1, 2, 7	

For peer review only - http://bmjopen.bmj.com/site/about/guidelines.xhtml

Outline any other processes that have been put in place to mitigate risks to participant safety (e.g. DMC, independent data review, etc.)<br><br>There is a pharmacovigilance aspect to this trial which will monitor all adverse events. There is no formal DMC due to the small size of study but some of this responsibility will be undertaken by the TSC.

16.2 Appendix 2 - Study management / responsibilities

The CI will manage the Trial Management file with the Trail manager who is based at the Anglia Ruskin Clinical Trials Unit (ARCTU). The CI will also manage the investigator site file (ISF) for Southend University hospital. The CI and trial manager will set up ISF for all sites participating in this trial, this will be managed by the PIs at each of the participating sites. The ISF will contain the same sections as the TMF.

The ISF will be actively maintained by the site PI from its establishment until the trial is formally closed. The ISF and source documentation (printed eCRF) will be stored in a locked cabinet or a room in a secure area with access only authorised by study personnel.

16.2.1 Patient registration/randomisation procedure

Randomisation will be carried out by the Clinical Trials Data Manager within ARCTU. ARCTU uses the TENALEA randomisation service provided by the Trans European Network for Clinical Trial Services. This is an internet based randomisation system, which will be set up for the study by ARCTU in accordance with protocol. This will be a 1:1 block randomisation, which will only be possible if the patient meets inclusion and exclusion criteria and has consented to take part in the trial. The system stores the pre-determined sequence of randomisation; this list is visible to neither the investigator nor ARCTU. The research nurse or investigator will log onto the web browser application and enter the patient's eligibility and stratification factors into the system; the treatment allocation is then returned to the investigator and selected members of ARCTU and study team.

16.2.2 Data management

Project data collection will be managed by the Clinical Trials Unit Data Manager who will oversee recruitment and collection of data. The responsibility for data collection and entry rests with the research nurse at each respective site. ARCTU uses an online data management system called MACRO to design and manage electronic case report forms (eCRFs). ARCTU will work with Southend University Hospital study team to design and validate the data collection tools. Once a patient is enrolled, the research team can access these forms remotely through the Internet portal. All data will be in anonymised form; patients will be identifiable only by study number. Data will be remotely monitored by ARCTU and discussed at data monitoring committee meetings. Any inconsistencies, validation errors or inaccuracies will be reported to the lead investigator regularly. Data queries will be sent regularly to each study site to ensure data is accurate and being entered in a timely manner. Once data collection is complete and the data has been validated, a data lock will be performed and analysis can begin.

16.2.3 Preparation and submission of amendments

For peer review only - http://bmjopen.bmj.com/site/about/guidelines.xhtml

Health Research Authority

Anakinra vs. Steroids for the Treatment of Gout Attacks in Renal Patients (ASGARD): A Feasibility Study

EudraCT no. 2015-001787-19

The CI along with the TSC will prepare and submit amendments. The overall process will be under the governance of the sponsor.

16.2.4 Preparation and submission of Annual Safety Report/Annual

The recruitment period is scheduled to last 15 months. The CI will prepare and submit an annual progress report to the main REC and to the sponsor as appropriate until the close of the study.

16.2.5 Data protection/confidentiality

All investigators and trial site staff must comply with the requirements of the Data Protection Act 1998 with regards to the collection, storage, processing and disclosure of personal information and will uphold the Act's core principles.

The Investigator has a responsibility to ensure that patient anonymity is protected and maintained. They must also ensure that their identities are protected from any unauthorised parties. Information with regards to study patients will be kept confidential and managed in accordance with the Data Protection Act, NHS Caldicott Guardian, The Research Governance Framework for Health and Social Care and Research Ethics Committee Approval.

Data will be stored for twenty years before being destroyed. The Chief Investigator in the data custodian.

16.2.6 Trial documentation and archiving

During the course of the research, all records are the responsibility of the Chief Investigator and will be kept in secure conditions at each designated site. When the research trial is complete, it is a requirement of the Research Governance Framework and Trust Policy that the records are kept for a further 20 years.

16.3 Appendix 3 – Authorisation of participating sites

16.3.1 Required documentation

- CVs of CI and site staff
- Protocol and any subsequent amendments
- Approved Participant Information Sheets
- Approved Consent Forms
- Indemnity documentation from sponsor
- Conditions of sponsorship from sponsor
- Final R&D Approval
- Signed site agreement
- Ethics submission and approval
- Laboratory accreditation letter, certification and normal ranges
- Delegation log
- Staff training log
- Site signature log
- Investigator's brochure
- Investigator site file

For peer review only - http://bmjopen.bmj.com/site/about/guidelines.xhtml

- Study drug accountability

16.3.2 Procedure for initiating/opening a new site

Site initiation will be managed by the trial coordinator at ARCTU and must be completed prior to any trial procedures taking place. The trial coordinator will arrange a site initiation visit, where the following information will be discussed:

- Investigator and study staff responsibilities
- Protocol review
- Investigator's brochure, including adverse events and serious adverse events
- Site recruitment action plan and recruitment expectations
- Informed consent procedures
- Source documentation and eCRF completion review
- Investigational product
- Lab procedure review
- Pharmacy procedure review
- Safety reporting
- Monitoring requirements
- Audits and Inspections
- Investigator site file

Once the trial coordinator has reviewed the necessary requirements related to the study and has received all required documentation, the site can begin trail procedures and the release of drug will be available for the participating site.

16.3.3 Principal Investigator responsibilities

The PI is responsible for managing the study and trial procedures carried out at the participating site. Prior to any study procedures taking place at the site, the PI is required to review the protocol and study documents and ensure that the site is set up and capable to handle trial procedures. The PI must attend the site initiation visit and training and participate in meetings and teleconferences related to the trial. The PI must confirm completion of training for site research staff, ensure that the ISF is accurately maintained, disseminate important safety or trial related information to the trial oversight committee and CI, and provide adequate safety reporting.

16.2 Appendix 2 - Study management / responsibilities

The CI will manage the Trial Management file with the Trail manager who is based at the Anglia Ruskin Clinical Trials Unit. The CI will also manage the investigator site file for Southend University hospital. The CI will set up ISF for all sites participating in this trial, this will be managed by the PIs at each of the participating sites. The ISF will contain the same sections as the TMF.

The ISF will be actively maintained by the site PI from its establishment until the trial is formally closed. The ISF and source documentation (printed eCRF) will be stored in a locked cabinet or a room in a secure are with access only authorised by study personnel.

For peer review only

For peer review only - http://bmjopen.bmj.com/site/about/guidelines.xhtml

Health Research Authority

16.4 Appendix 4 – Schedule of Procedures

SCHEDULE OF PROCEDURES

Procedures	Screening	Baseline (pre-treatment) Day 1	Day 2	Day 3	Day 4	Day 5	Day 6	Day 7	8 weeks
		Treatment phase							
			Follow up						
Informed Consent	X								
Demographics	X								
Medical History	X								
Physical examination	X		X						
Vital signs	X		X						
Blood and urine tests	X	X	X					X	X
Concomitant medications	X		X	X	X	X	X	X	
Eligibility assessment	X								
Randomisation		X							
Dispensing of trial drug		X							
Drug administration		X	X	X	X	X			
Patient reported pain and outcome assessments (diary entry)		X	X	X	X	X	X	X	
Physician assessment of joint		X	X					X	

For peer review only

For peer review only - http://bmjopen.bmj.com/site/about/guidelines.xhtml

Patient assessment of activity limitation and quality of life using:									
• LEFS		X	X					X	
• EQ-5D-5L		X	X					X	X
• HAQ-DI and SF-36		X						X	X
Compliance Assessments			X	X	X	X	X	X	
Checking patient diary entry for time of injection and pain assessment (by phone or face to face)			X	X	X	X	X	X	
Diary return								X	
Check injection sites		X	X					X	
Check returned syringes								X	
Questionnaire return (face to face or by mail)									X
Adverse events assessments (face to face or by phone)		X	X	X	X	X	X	X	X
Physician's withdrawal checklist									
Interview (12 patients)									X
Resource use data									X

For peer review only

Health Research Authority

Anakinra vs. Steroids for the Treatment of Gout Attacks in Renal Patients (ASGARD): A Feasibility Study

EudraCT no. 2015-001787-19

For peer review only

For peer review only - http://bmjopen.bmj.com/site/about/guidelines.xhtml

NHS
Health Research Authority

Anakinra vs. Steroids for the Treatment of Gout Attacks in Renal Patients (ASGARD): A Feasibility Study

EudraCT no. 2015-001787-19

16.5 Appendix 5 – Safety Reporting Flow Chart

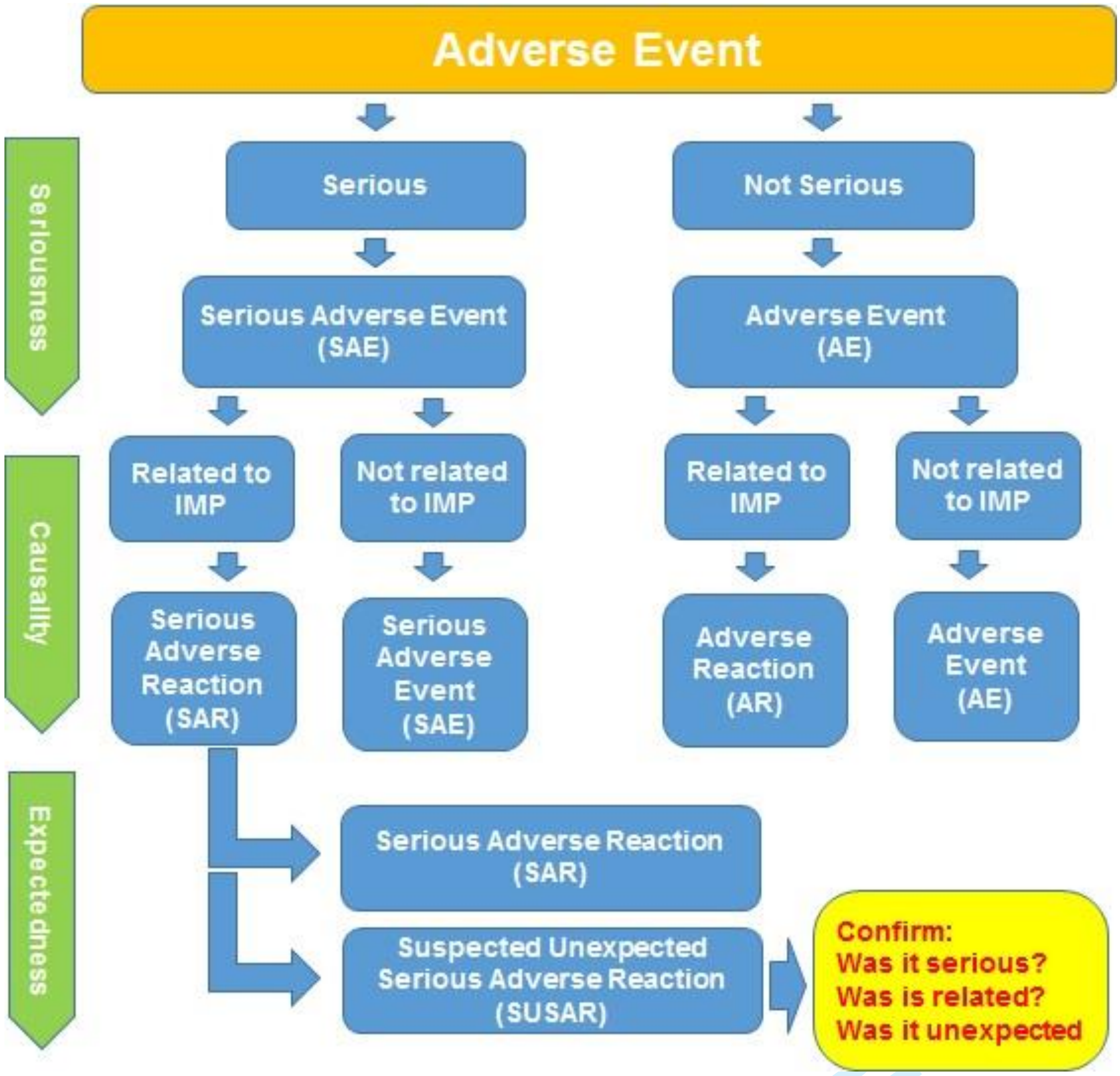

**Adverse Event (AE)**	Any untoward medical occurrence in a participant to whom a medicinal product has been administered, including occurrences which are not necessarily caused by or related to that product.
**Adverse Reaction (AR)**	An untoward and unintended response in a participant to an investigational medicinal product which is related to any dose administered to that participant.<br>The phrase "response to an investigational medicinal product" means that a causal relationship between a trial medication and an AE is at least a reasonable possibility, i.e. the relationship cannot be ruled out.<br>All cases judged by either the reporting medically qualified professional or the Sponsor as having a reasonable suspected causal relationship to the trial medication qualify as adverse reactions.
**Serious Adverse Event (SAE)**	A serious adverse event is any untoward medical occurrence that results in death; is life-threatening, requires inpatient; hospitalisation or prolongation of existing hospitalisation; results in persistent or significant disability/incapacity; or consists of a congenital anomaly or birth defect.<br>Other 'important medical events' may also be considered serious if they jeopardise the participant or require an intervention to prevent one of the above consequences.<br>NOTE: The term "life-threatening" in the definition of "serious" refers to an event in which the participant was at risk of death at the time of the event; it does not refer to an event which hypothetically might have caused death if it were more severe.
**Serious Adverse Reaction (SAR)**	An adverse event that is both serious and, in the opinion of the reporting Investigator, believed with reasonable probability to be due to one of the trial treatments, based on the information provided.

For peer review only - http://bmjopen.bmj.com/site/about/guidelines.xhtml

**Suspected Unexpected Serious Adverse Reaction (SUSAR)**	A serious adverse reaction, the nature and severity of which is not consistent with the information about the medicinal product in question set out in the case of a product with a marketing authorisation, in the summary of product characteristics (SmPC) for that product or in the case of any other investigational medicinal product, in the investigator's brochure (IB) relating to the trial in question.

16.6 Appendix 6 – Amendment History

Amendment No.	Protocol version no.	Date issued	Author(s) of changes	Details of changes made
1	1.1	17/11/15	G Balasubramaniam	Removal of day 4 assessment due to feedback from recruiting centres and outcome of TMG discussion. Week 8 visit for all patients to record health resource use and blood tests.
2	1.2	01/01/16	G Balasubramaniam	Exclusion criteria amended to be clear to exclude patients requiring intra-venous anti-biotic administration, or within two weeks of such treatment.<br><br>Abstinence from sex clarified as true abstinence i.e. non-periodic (calendar, ovulation, sympthothermal, post-ovulation methods)<br><br>Included a rationale for dose and dose duration for Anakinra (Kineret)
3	1.3	13/05/16	G Balasubramaniam	Trial pharmacist changed from Stuart Chandler to trial pharmacists: Amiirah Ropun, Inisa Idress and Laura Wilson.<br><br>Trial statistician changed from Michael Parker to Jufen Zhang.
4	1.3	01/07/16	G Balasubramaniam	Information in baseline assessment pertaining to new diagnostic gout criteria in 2015 (ACR and EULAR) taken in baseline assessment.

For peer review only - http://bmjopen.bmj.com/site/about/guidelines.xhtml

NHS
Health Research Authority

Anakinra vs. Steroids for the Treatment of Gout Attacks in Renal Patients (ASGARD): A Feasibility Study

EudraCT no. 2015-001787-19

5	1.3	01/07/16	G Balasubramaniam	Use of CKD EPI or MDRD (as per local guidelines) to calculate eGFR
6	1.3	01/07/16	G Balasubramaniam	Men of child bearing age not willing to undertake highly effective contraception during treatment period excluded
7	1.3	01/07/16	G Balasubramaniam	One further centre added Basildon and Thurrock University Hospital NHS Trust.
8	1.3	01/07/16	G Balasubramaniam	PI changed at Mid Essex NHS Trust
9	1.3	01/07/16	G Balasubramaniam	Participants can be contacted on Friday and Monday as a reminder for weekend or compliance check respectively.
10	1.3	01/07/16	G Balasubramaniam	Baseline assessments can be made after four hours as opposed to six hours if permitted form of analgesia used.
11	1.3	01/07/16	G Balasubramaniam	ARCTU email added in SAE / SUSAR section to help with monitoring.
12	1.4	01/02/17	G Balasubramaniam	Depo-Medrone changed to generic term of intramuscular methylprednisolone throughout protocol.
13	1.4	01/02/17	G Balasubramaniam	Exclusion criteria changed to specific criteria for each treatment i.e. Colchicine, Steroids and NSAIDS
14	1.4	01/02/17	G Balasubramaniam	Line in exclusion criteria 5, (7 in new version 1.4) "Con-current medication for RA like methotrexate and anti-TNF treatment has been associated with increased risk of neutropenia and infection" removed as repeats

For peer review only - http://bmjopen.bmj.com/site/about/guidelines.xhtml

				exclusion criteria 4 (6 in version 1.4)
15	1.4	01/02/17	G Balasubramaniam	Exclusion criteria 13; potential participants who were given empirical treatment with antibiotics but infection has been excluded can be recruited.
16	1.4	01/02/17	G Balasubramaniam	Exclusion criteria 14; potential participants uncontrolled concomitant disease. The term “serious” has been removed and potential participation in the trial should be in agreement with the direct medical team.
17	1.4	01/02/17	G Balasubramaniam	Pre-screening for potential participants.
18	1.4	01/02/17	G Balasubramaniam	Use of present renal blood tests that may show an acute kidney injury will not be used to calculate baseline renal function.
19	1.5	13/03/2017	G Balasubramaniam	Trial Manager changed from Trisha Parker to Arit Udoh.
20	1.5	13/03/2017	G Balasubramaniam	Section 7.13 Rescue medication added. Rescue treatment is Prednisolone 35mg po orally, with additional low dose colchicine if needed.
21	1.5	13/03/2017	G Balasubramaniam	Typographical error with blood collection in page 41.

List details of all protocol amendments here whenever a new version of the protocol is produced.

Protocol amendments must be submitted to the Sponsor for approval prior to submission to the REC committee or MHRA.

For peer review only - http://bmjopen.bmj.com/site/about/guidelines.xhtml

Health Research Authority

Anakinra vs. Steroids for the Treatment of Gout Attacks in Renal Patients (ASGARD): A Feasibility Study

EudraCT no. 2015-001787-19

For peer review only

For peer review only - http://bmjopen.bmj.com/site/about/guidelines.xhtml

PARTICIPANT INFORMATION SHEET (VERSION 1.1)

Centre Number: Study Number:

ASGARD: THE STUDY OF ANAKINRA VERSUS STEROIDS FOR THE TREATMENT OF ACUTE GOUT ATTACKS IN PEOPLE WITH KIDNEY DISEASE

Invitation to participate in the above project

We would like to invite you to take part in our research project. Before you decide, we would like you to understand why the research is being done and what it would involve for you. One of our team will go through the information sheet with you and answer any questions you have. We'd suggest this should take about 30 minutes. Talk to others about the study if you wish and ask us if there is anything that is not clear.

Part 1 tells you the purpose of this study and what will happen to you if you take part. Part 2 gives you more details information about the conduct of the study.

Part 1

What is the purpose of the project?

Gout attacks affect approximately 1.3 million people with kidney disease in the UK. We do not know the safest and best way to treat an attack of gout in these patients. We want to compare the safest treatment currently available, steroids, with a new treatment that is being increasingly used called Anakinra. Anakinra stops the action of a chemical called interleukin-1, which plays an important role in the acute gout attack, and we think it might be a better alternative to steroid treatment. This project is looking at a small number of people and will provide information to plan a larger study. We hope the larger study will then go on to tell us which treatment may be better, safer and more cost-effective.

Why have I been invited?

You are being asked to participate in this clinical research project because you have acute gout and kidney disease.

Do I have to take part?

No, it is up to you to decide whether or not to join the project. We will describe the project and go through this information sheet. If you agree to take part, we will ask you to sign a consent form. You are free to withdraw at any time, without giving a reason. This would not affect the standard of care you receive.

What will happen to me if I take part?

If you agree to take part in the project, a member of the project team may undertake some additional blood tests (about two teaspoonful) to make sure it is suitable for you to be involved with the project. If this is ok, then you will be asked to fill a consent form to proceed. This project lasts for 8 weeks but most of your involvement will be for the first 7 days.

For peer review only - http://bmjopen.bmj.com/site/about/guidelines.xhtml

ASGARD

PARTICIPANT INFORMATION SHEET (VERSION 1.1)

Centre Number: **Study Number:**

This project compares two different treatments. We put people into two groups and give each group a different treatment. The results are then compared to see if one is better. To try and make sure the groups are the same to start with, each participant is put into a group by chance (randomly). The chance of being on a particular type of treatment in 50:50. This is a “double-blind trial,” so neither you nor your doctor will know in which treatment group you are in (although, if your doctor needs to find out he/she can do so).

We will undertake an assessment on the first day (Day 1) before you receive the treatment. This will take about an hour and consists of clinical questions and examination, blood tests and you will be asked to complete some questionnaires. You will also be issued a diary for you to assess your pain and you will be asked to complete some questionnaires to assess how the pain is affecting your ability to go about your day.

You will then be given the two injections, one in the buttock, and one under the skin in your tummy or thigh. The injection into your buttock would have been part of your usual care anyway.

You will also be issued a further 4 injections to give under the skin daily over the next 4 days. We will teach you or someone who lives with you to give these injections. If we cannot do this, we can organise a nurse to visit your home to do this. You will be given a small bin to dispose of your empty syringes safely and return after 7 days.

An outline of your visits and assessments following your enrolment is shown in the figure below.

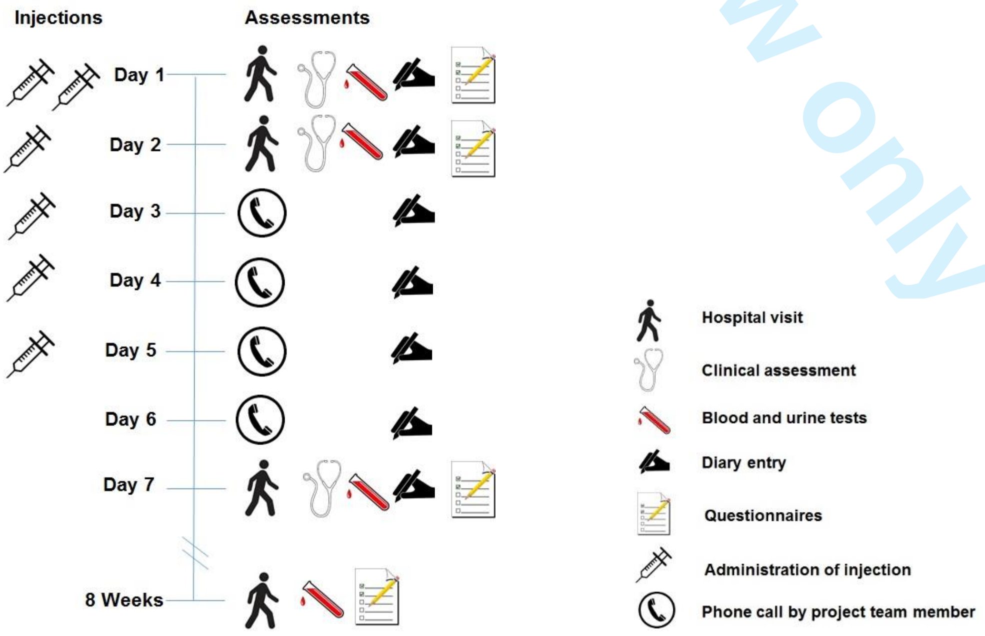

You will then have to attend hospital on the second and seventh day for about an hour. We will call you on the days you don’t attend hospital to check that you have had the treatment

For peer review only - http://bmjopen.bmj.com/site/about/guidelines.xhtml

ASGARD

PARTICIPANT INFORMATION SHEET (VERSION 1.1)

Centre Number: **Study Number:**

and filled your diary. There is a final visit and assessment after 8 weeks for blood tests and questionnaires, one additional questionnaire will look at all the NHS services you have used over the last eight weeks. You may also be asked to have an interview to give your feelings on being part of this project, you do not need to do this if you don't want to.

Your contact information will be stored at your local hospital so that we can contact you about the study, any results of the project and any future studies that we may plan.

You will have blood tests (about two tablespoons) and urine samples (a small cupful) on the second day, the seventh day and after eight weeks. Some of these samples will be tested straight away in the local NHS laboratory, while some will be processed, stored and analysed at the end of the study at Southend Hospital or specialised laboratories linked to the project. With your consent, a tiny sample of your blood plasma, serum, urine and DNA will be stored in a clinical laboratory at Southend University Hospital under the custodianship of Dr Gowrie Balasubramaniam (Chief Investigator).

Expenses and payments

You will not be paid for taking part in the study. We can pay for travel expenses on the days you need to attend hospital.

What will I have to do?

You will have to receive additional injections for five days and make assessments for the week. You will be issued with a diary to make the assessments, this should take less than five minutes a day. We will call you to make sure you have done this. You will also have to attend hospital for a clinical assessment and to fill some questionnaires on the second day, the seventh day and at eight weeks. This visit will probably take approximately forty-five minutes, with forty minutes for the questionnaires and five minutes for the assessment and blood tests.

A small number of participants (12) will be asked to undertake an interview session when you attend at eight weeks. This can take up to an additional hour. We will ask you about your experience of participating in the project and your answers will be recorded using an audio recording device. We will specifically choose participants to give a broad representation of people based on age and gender. The questions you will be asked will be related to your feelings on what you had to do as part of this project, we will also ask if you feel we could do things differently. This will help improve our future research. You can decide not to undertake this particular aspect of the project if you wish.

What are the alternatives for treatment?

Two other alternative treatments are available. NSAIDS are the best treatment for gout but they can be harmful in people with kidney disease. Colchicine is also a good treatment but can cause severe diarrhoea and other serious complications, as colchicine can build up in the body with kidney failure.

For peer review only - http://bmjopen.bmj.com/site/about/guidelines.xhtml

PARTICIPANT INFORMATION SHEET (VERSION 1.1)

Centre Number: **Study Number:**

What are the possible disadvantages, risks and side-effects?

Anakinra is a new treatment that works by reducing your immune reaction and there can be complications associated with this. In one study where Anakinra was used to help treat people with Rheumatoid Arthritis, there was a risk (<2%) of picking up an infection (like a chest infection) and a lowering of the white blood cell count. These participants were on other treatments that weakened their immune reaction and treatment with Anakinra lasted for many months. We do not anticipate these problems as we are not involving people on other treatments that affect the immune system and treatment is only for 5 days.

We will make sure that you don't have a serious infection and that your blood counts are normal before you are involved with the project. You will be monitored to make sure everything is going well and we will also check your blood counts.

You will be receiving injections under the skin for five days. Some people have had reactions where they inject their skin for many months but we do not feel this will be a problem for the five days you will receive the injection. We will have a look at the injection site when you visit the hospital.

We will ask that you refrain from using an additional or rescue pain killer for four hours prior to a pain assessment, this is to obtain a more accurate and fair comparison of the two treatments. You would normally have to wait for four hours in between doses of pain killers anyway, so you can minimise any discomfort by doing the assessment just before a dose. You do not have to do the assessments at the exact time every day but at roughly the same sort of time i.e. late morning or afternoon.

What are the possible benefits of taking part?

You will not be given a treatment that we already know may be harmful for you. You will be looked after by a specialist and you will be given contact details to use if there are any problems. If this study is successful then it would help develop clear guidelines to help doctors treat people with kidney disease and gout, something we know is currently not being done well.

There is also some suggestion that using this novel treatment may be beneficial for other conditions.

Will taking part in the study affect your usual care?

The only alteration to your treatment is that you will be asked to refrain from taking additional or rescue painkillers for four hours prior to doing an assessment of your joint. You would normally wait for four hours in between doses of painkillers and we would suggest that you do the assessment just before your next dose. We will not alter any of your other medications or interfere with your other treatments in any way. You will continue to be seen by your GP and by any hospital clinics that you usually attend.

For peer review only - http://bmjopen.bmj.com/site/about/guidelines.xhtml

PARTICIPANT INFORMATION SHEET (VERSION 1.1)

Centre Number: **Study Number:**

What happens when the research study stops?

The medication is only used as part of the study. Any future gout attacks you have will be treated as before. You will not know what exact treatment you have had as part of the study.

What if there is a problem?

Any complaint about the way you have been dealt with during the study or any possible harm you might suffer will be addressed. The detailed information on this is given in Part 2.

Will my taking part in this study be kept confidential?

Yes, we will follow ethical and legal practice and all the information about you will be handled in confidence. The details are included in part 2.

This completes Part 1 of the information sheet.

For peer review only - http://bmjopen.bmj.com/site/about/guidelines.xhtml

ASGARD

PARTICIPANT INFORMATION SHEET (VERSION 1.1)

Centre Number: **Study Number:**

Part 2 of the information sheet

If the information in Part 1 has interested you and you are considering participation please read the additional information in Part 2 before making any decision.

What if relevant new information becomes available?

Sometimes we get new information about the treatment being studied. If this happens your research doctor will tell you and discuss whether you should continue in the study. If you decide not to carry on, your research doctor will make arrangements for your care to continue. If you decide to continue in the study, he/she may ask you to sign an agreement outlining the discussion.

What will happen if I don't want to carry on with the study?

You can withdraw from treatment at any time but keep in contact with us to let us know your progress. Information collected may still be used. Any stored blood samples that can still be identified as yours can be destroyed if you so wish.

What if there is a problem?

If you have a concern about any aspect of this study, you should ask to speak to the researchers who will do their best to answer your questions. If you remain unhappy and wish

to complain formally, you can do this using the Complaints Officer for ___________________

hospital on: _______________. Details can be obtained from__________________________.

In the event something goes wrong and you are harmed during the project, and this is due to someone's negligence then you may have grounds for a legal action for compensation

against _____________________ hospital but you may have to pay your legal costs. The normal National Health Service complaints mechanisms will still be available to you.

Will my taking part in this study be kept confidential?

If you join the study, some parts of your medical records and the data collected for the study will be looked at by authorised persons from the organisers of the research. They may also be looked at by authorised people to check that the study is being carried out correctly. All will have a duty of confidentiality to you as the research participant and we will do our best to meet this duty.

Your research data will be stored using a unique study code which is non-identifiable, but can be linked back to your original records. All written information will be kept in a locked filing cabinet in a locked room. Any web-based data will be stored in a secure password protected central database at the Anglia Ruskin Clinical Trials Unit. Some of the data will be

For peer review only - http://bmjopen.bmj.com/site/about/guidelines.xhtml

PARTICIPANT INFORMATION SHEET (VERSION 1.1)

Centre Number: **Study Number:**

analysed at the University of East Anglia and the University of Essex. Only individuals directly involved with the study will have access to this information. All data will be under the custodianship of Dr Gowrie Balasubramaniam. Information may be retained for future studies but this will be after the appropriate ethical approvals. It will be retained for fifteen years and be disposed of securely after this period.

Will my GP know about this project?

We will inform your GP of your participation with your permission and we will also inform him/her of any clinical results, and of any new medical problems we might find as a result of your participation in the project.

What will happen to any samples I give?

Blood and urine samples taken for this study will be processed at the local centre and transferred for storage to Southend University Hospital. The samples with be labelled with a unique study code which will not identify you, but can be linked back to your original records. The samples can be stored for up to fifteen years and some will be sent to other laboratories both in the UK and within the European Union for further analysis. They may be used for future research that cannot yet be specified but this will be done after the appropriate ethical approvals. Samples will be under the custodianship of Dr Gowrie Balasubramaniam.

Will any genetic tests be done?

We will test for changes in the structure of the DNA. There will be **no** DNA genetic testing per se that may reveal significant results such as a family genetic condition etc.

What will happen to the results of the research study?

The results will be examined by the researcher at the end of the study and a report will be produced. We will share our results with the funding body, the National Institute for Health Research. We may publish the study in a scientific journal and present the results at scientific meetings. You will not be identified in any of the reports, presentations or publications. We hope to use the results to undertake a larger, more definitive study.

A summary of the findings of the study can be made available to you if you wish from the research team or the principle investigator.

Who is organising and funding the research?

The research is being organised by Southend University Hospital. It is being funded by the National Institute of Health Research.

Who has reviewed the study?

For peer review only - http://bmjopen.bmj.com/site/about/guidelines.xhtml

PARTICIPANT INFORMATION SHEET (VERSION 1.1)

Centre Number: **Study Number:**

All research in the NHS is looked at by independent group of people, called a Research Ethics Committee, to protect your interests. This study has been reviewed and given a favourable opinion by The London Research Ethics Committee.

What if I become unwell during the study?

If during the study you become unwell or are concerned, as well as the usual services provided by the NHS, you can also contact a member of the study team on this number during normal working hours on <PI/Research member contact number>.

You can also contact <a research member of the team or the principle investigator out-of-hours> via the hospital switch board on this number <centre switch board number>.

If you are unwell, and need urgent advice or assistance do not delay in seeking further advice or treatment as usual through the NHS services.

Thank you for taking the time to read this information sheet and considering to take part in this study. If you would like more information or want to ask questions about the study please contact the study team.

For further information contact:

Research Nurse

Principal Investigator

<Contact details>

For peer review only

For peer review only - http://bmjopen.bmj.com/site/about/guidelines.xhtml

CONSENT FORM (VERSION 1.2)

Centre Number: **Study Number:**

Participation Id. no. for this trial:

ASGARD STUDY: How does a new treatment called Anakinra, an Interleukin-1 inhibitor, compare with the currently available best treatment, Steroids, for the treatment of gout attacks in people with kidney disease

Name of Researcher: Study reference:

This form must be completed and signed by the research participant in the presence of Principle Investigator or someone from the research team designated by the Principle Investigator

For peer review only

Please initial box

1. I confirm that I have read and understood the information sheet dated …………………. (Version……….). I have had the opportunity to consider the information, ask questions and have had these answered satisfactorily. ☐

2. I understand that my participation is entirely voluntary and that I am free to withdraw at any time without giving any reason, without my medical or legal rights being affected. ☐

3. I understand that relevant sections of my medical notes and data collected will be looked at by individuals who are part of the study team (including staff from Southend University Hospital, Anglia Ruskin University, University of East Anglia and University of Essex), from regulatory authorities or from the NHS Trust, where it is relevant to my taking part in this research. I give permission for these individuals to have access to my records. ☐

4. I understand, and I agree that my identifiable routine blood tests will be stored within the NHS clinical system and will be available to doctors looking after me in the future. ☐

5. I understand that my participation in the study is voluntary and that I am free to leave the study at any time without having to give a reason and that this will not affect my medical care in any way. ☐

6. I understand that I will be asked to refrain from using additional / rescue pain killers for four hours (normal time in-between doses) prior to an assessment, this may mean that I make a slight adjustment to perform an assessment before I take the next dose. ☐

7. I agree that if I withdraw or I am withdrawn from the study that data already collected can be retained and included in the data analysis. ☐

8. I understand that I will be required to give blood samples as part of this study. ☐

For peer review only - http://bmjopen.bmj.com/site/about/guidelines.xhtml

ASGARD

CONSENT FORM (VERSION 1.2)

Centre Number: **Study Number:**

Participation Id. no. for this trial:

9. I understand and I agree that the samples I provide will be gifted by myself for use in future research. They will be transferred to specialist laboratories and analysed by members of the study team or stored (link-anonymised) for up to fifteen years and can be used for future, as yet unspecified, medical research into health, illness and medical treatment. This research will be subject to proper scientific and ethical review. (Optional) ☐

10. I agree, if required, to undertake an interview and give permission for audio recordings that will be stored for a small qualitative aspect of this study. (Optional) ☐

11. I agree that my contact information can be made available to the research team as part of the study assessments, for any new findings that may become relevant during the course of the study and for future studies that I might be interested in. ☐

12. I give consent for storage and studies on my DNA. I understand that this is **not** genetic testing per se that may reveal significant results such as a family genetic condition. (Optional) ☐

13. I agree that samples can be exported to countries in the European Union for further analysis. The collaborators / recipient country will ensure that the material has been handled properly and that the required standards of that collaborating institution / country have been met. (Optional) ☐

14. I agree to my GP being informed of my participation in the study. ☐

15. I agree to take part in the above study. ☐

____________________	____________	____________
Name of Patient	Date	Signature

____________________	____________	____________
Name of Person taking consent	Date	Signature

When completed: 1 for participant 1 for researcher site file; 1 (original) to be kept in medical notes.

For peer review only

For peer review only - http://bmjopen.bmj.com/site/about/guidelines.xhtml

SPIRIT — Standard Protocol Items: Recommendations for Interventional Trials

SPIRIT 2013 Checklist: Recommended items to address in a clinical trial protocol and related documents*

Section/item	Item No	Description	Addressed on page number
**Administrative information**			
Title	1	Descriptive title identifying the study design, population, interventions, and, if applicable, trial acronym	1
Trial registration	2a	Trial identifier and registry name. If not yet registered, name of intended registry	2
	2b	All items from the World Health Organization Trial Registration Data Set	2
Protocol version	3	Date and version identifier	1____
Funding	4	Sources and types of financial, material, and other support	2_____________
Roles and responsibilities	5a	Names, affiliations, and roles of protocol contributors	1 and submission details__
	5b	Name and contact information for the trial sponsor	main protocol (supplementary material)___
	5c	Role of study sponsor and funders, if any, in study design; collection, management, analysis, and interpretation of data; writing of the report; and the decision to submit the report for publication, including whether they will have ultimate authority over any of these activities	main protocol (supplementary material)___

For peer review only - http://bmjopen.bmj.com/site/about/guidelines.xhtml

	5d	Composition, roles, and responsibilities of the coordinating centre, steering committee, endpoint adjudication committee, data management team, and other individuals or groups overseeing the trial, if applicable (see Item 21a for data monitoring committee)	main protocol (supplementary material)___
**Introduction**			
Background and rationale	6a	Description of research question and justification for undertaking the trial, including summary of relevant studies (published and unpublished) examining benefits and harms for each intervention	_3____________
	6b	Explanation for choice of comparators	_5____________
Objectives	7	Specific objectives or hypotheses	_6____________
Trial design	8	Description of trial design including type of trial (eg, parallel group, crossover, factorial, single group), allocation ratio, and framework (eg, superiority, equivalence, noninferiority, exploratory)	_6____________
**Methods: Participants, interventions, and outcomes**			
Study setting	9	Description of study settings (eg, community clinic, academic hospital) and list of countries where data will be collected. Reference to where list of study sites can be obtained	_7____________
Eligibility criteria	10	Inclusion and exclusion criteria for participants. If applicable, eligibility criteria for study centres and individuals who will perform the interventions (eg, surgeons, psychotherapists)	_7____________
Interventions	11a	Interventions for each group with sufficient detail to allow replication, including how and when they will be administered	_7____________
	11b	Criteria for discontinuing or modifying allocated interventions for a given trial participant (eg, drug dose change in response to harms, participant request, or improving/worsening disease)	_8____________
	11c	Strategies to improve adherence to intervention protocols, and any procedures for monitoring adherence (eg, drug tablet return, laboratory tests)	_8___________
	11d	Relevant concomitant care and interventions that are permitted or prohibited during the trial	_8____________

For peer review only - http://bmjopen.bmj.com/site/about/guidelines.xhtml

Outcomes	12	Primary, secondary, and other outcomes, including the specific measurement variable (eg, systolic blood pressure), analysis metric (eg, change from baseline, final value, time to event), method of aggregation (eg, median, proportion), and time point for each outcome. Explanation of the clinical relevance of chosen efficacy and harm outcomes is strongly recommended	_8___________
Participant timeline	13	Time schedule of enrolment, interventions (including any run-ins and washouts), assessments, and visits for participants. A schematic diagram is highly recommended (see Figure)	_9___________
Sample size	14	Estimated number of participants needed to achieve study objectives and how it was determined, including clinical and statistical assumptions supporting any sample size calculations	_9___________
Recruitment	15	Strategies for achieving adequate participant enrolment to reach target sample size	_9__________

Methods: Assignment of interventions (for controlled trials)

Allocation:			
Sequence generation	16a	Method of generating the allocation sequence (eg, computer-generated random numbers), and list of any factors for stratification. To reduce predictability of a random sequence, details of any planned restriction (eg, blocking) should be provided in a separate document that is unavailable to those who enrol participants or assign interventions	_10____________
Allocation concealment mechanism	16b	Mechanism of implementing the allocation sequence (eg, central telephone; sequentially numbered, opaque, sealed envelopes), describing any steps to conceal the sequence until interventions are assigned	_10___________
Implementation	16c	Who will generate the allocation sequence, who will enrol participants, and who will assign participants to interventions	_11____________
Blinding (masking)	17a	Who will be blinded after assignment to interventions (eg, trial participants, care providers, outcome assessors, data analysts), and how	_11____________
	17b	If blinded, circumstances under which unblinding is permissible, and procedure for revealing a participant's allocated intervention during the trial	_11____________

Methods: Data collection, management, and analysis

Data collection methods	18a	Plans for assessment and collection of outcome, baseline, and other trial data, including any related processes to promote data quality (eg, duplicate measurements, training of assessors) and a description of study instruments (eg, questionnaires, laboratory tests) along with their reliability and validity, if known. Reference to where data collection forms can be found, if not in the protocol	_11____________
	18b	Plans to promote participant retention and complete follow-up, including list of any outcome data to be collected for participants who discontinue or deviate from intervention protocols	_12____________
Data management	19	Plans for data entry, coding, security, and storage, including any related processes to promote data quality (eg, double data entry; range checks for data values). Reference to where details of data management procedures can be found, if not in the protocol	_12___________
Statistical methods	20a	Statistical methods for analysing primary and secondary outcomes. Reference to where other details of the statistical analysis plan can be found, if not in the protocol	_12___________
	20b	Methods for any additional analyses (eg, subgroup and adjusted analyses)	_13____________
	20c	Definition of analysis population relating to protocol non-adherence (eg, as randomised analysis), and any statistical methods to handle missing data (eg, multiple imputation)	_13____________
**Methods: Monitoring**			
Data monitoring	21a	Composition of data monitoring committee (DMC); summary of its role and reporting structure; statement of whether it is independent from the sponsor and competing interests; and reference to where further details about its charter can be found, if not in the protocol. Alternatively, an explanation of why a DMC is not needed	_13____________
	21b	Description of any interim analyses and stopping guidelines, including who will have access to these interim results and make the final decision to terminate the trial	_13____________
Harms	22	Plans for collecting, assessing, reporting, and managing solicited and spontaneously reported adverse events and other unintended effects of trial interventions or trial conduct	__14___________
Auditing	23	Frequency and procedures for auditing trial conduct, if any, and whether the process will be independent from investigators and the sponsor	__15___________
**Ethics and dissemination**			

For peer review only - http://bmjopen.bmj.com/site/about/guidelines.xhtml

Research ethics approval	24	Plans for seeking research ethics committee/institutional review board (REC/IRB) approval	__15____________
Protocol amendments	25	Plans for communicating important protocol modifications (eg, changes to eligibility criteria, outcomes, analyses) to relevant parties (eg, investigators, REC/IRBs, trial participants, trial registries, journals, regulators)	___15___________
Consent or assent	26a	Who will obtain informed consent or assent from potential trial participants or authorised surrogates, and how (see Item 32)	__15____________
	26b	Additional consent provisions for collection and use of participant data and biological specimens in ancillary studies, if applicable	__16____________
Confidentiality	27	How personal information about potential and enrolled participants will be collected, shared, and maintained in order to protect confidentiality before, during, and after the trial	__16____________
Declaration of interests	28	Financial and other competing interests for principal investigators for the overall trial and each study site	___16___________
Access to data	29	Statement of who will have access to the final trial dataset, and disclosure of contractual agreements that limit such access for investigators	___17___________
Ancillary and post-trial care	30	Provisions, if any, for ancillary and post-trial care, and for compensation to those who suffer harm from trial participation	___17___________
Dissemination policy	31a	Plans for investigators and sponsor to communicate trial results to participants, healthcare professionals, the public, and other relevant groups (eg, via publication, reporting in results databases, or other data sharing arrangements), including any publication restrictions	___17___________
	31b	Authorship eligibility guidelines and any intended use of professional writers	__17____________
	31c	Plans, if any, for granting public access to the full protocol, participant-level dataset, and statistical code	__17____________
**Appendices**			
Informed consent materials	32	Model consent form and other related documentation given to participants and authorised surrogates	Supplementary material_________ ____

For peer review only - http://bmjopen.bmj.com/site/about/guidelines.xhtml

Biological specimens	33	Plans for collection, laboratory evaluation, and storage of biological specimens for genetic or molecular analysis in the current trial and for future use in ancillary studies, if applicable	_16____________

*It is strongly recommended that this checklist be read in conjunction with the SPIRIT 2013 Explanation & Elaboration for important clarification on the items. Amendments to the protocol should be tracked and dated. The SPIRIT checklist is copyrighted by the SPIRIT Group under the Creative Commons "Attribution-NonCommercial-NoDerivs 3.0 Unported" license.

For peer review only
